# A Revised Molecular Model of Ovarian Cancer Biomarker CA125 (MUC16) Enabled by Long-read Sequencing

**DOI:** 10.1158/2767-9764.CRC-23-0327

**Published:** 2024-01-31

**Authors:** Chien-Wei Wang, Simon D. Weaver, Nicha Boonpattrawong, Naviya Schuster-Little, Manish Patankar, Rebecca J. Whelan

**Affiliations:** 1 Department of Chemistry, University of Kansas, Lawrence, Kansas.; 2 Department of Chemistry and Biochemistry, University of Notre Dame, Notre Dame, Indiana.; 3 Integrated Biomedical Sciences Graduate Program, University of Notre Dame, Notre Dame, Indiana.; 4 Department of Obstetrics and Gynecology, University of Wisconsin–Madison, Madison, Wisconsin.

## Abstract

The biomarker CA125, a peptide epitope located in several tandem repeats of the mucin MUC16, is the gold standard for monitoring regression and recurrence of high-grade serous ovarian cancer in response to therapy. However, the CA125 epitope along with several structural features of the MUC16 molecule are ill defined. One central aspect still unresolved is the number of tandem repeats in MUC16 and how many of these repeats contain the CA125 epitope. Studies from the early 2000s assembled short DNA reads to estimate that MUC16 contained 63 repeats.

Here, we conduct Nanopore long-read sequencing of MUC16 transcripts from three primary ovarian tumors and established cell lines (OVCAR3, OVCAR5, and Kuramochi) for a more exhaustive and accurate estimation and sequencing of the MUC16 tandem repeats.

The consensus sequence derived from these six sources was confirmed by proteomics validation and agrees with recent additions to the NCBI database. We propose a model of MUC16 containing 19—not 63—tandem repeats. In addition, we predict the structure of the tandem repeat domain using the deep learning algorithm, AlphaFold.

The predicted structure displays an SEA domain and unstructured linker region rich in proline, serine, and threonine residues in all 19 tandem repeats. These studies now pave the way for a detailed characterization of the CA125 epitope. Sequencing and modeling of the MUC16 tandem repeats along with their glycoproteomic characterization, currently underway in our laboratories, will help identify novel epitopes in the MUC16 molecule that improve on the sensitivity and clinical utility of the current CA125 assay.

**Significance:**

Despite its crucial role in clinical management of ovarian cancer, the exact molecular sequence and structure of the biomarker, CA125, are not defined. Here, we combine long-read sequencing, mass spectrometry, and *in silico* modeling to provide the foundational dataset for a more complete characterization of the CA125 epitope.

## Introduction

There is an unmet need for innovative molecular tools that can support the clinical management of high-grade serous ovarian cancer, given that more than 220,000 women in the United States are living with ovarian cancer and at risk of recurrence (1). Improving the performance of validated biomarkers by considering not just the known epitope but the entire molecule and glycoproteoform variants is a compelling and underused approach to meet this need. CA125 is a crucial biomarker in the clinical management of ovarian cancer (2, 3) whose molecular structure remains incompletely characterized (4–6). Efforts to determine the molecular nature of the CA125 epitope(s) have revealed mechanisms of both false positive and false negative recognition of CA125 in patient samples (7, 8), and the exact location of the epitope(s) remains unknown (9, 10).

Current understanding of the molecular structure of CA125 derives from studies conducted in the early 2000s that located the epitopes within the repetitive tandem repeat domain of the highly glycosylated mucin, MUC16 (11–14). The prevailing model describes MUC16 as containing a large glycosylated N-terminal domain, a tandem repeat domain containing approximately 60 epitope-presenting subunits, and a short C-terminal region. This model was derived using short-read genome assembly (11). The short reads of first- and second-generation DNA sequencing technology were limited in their scope and accuracy especially for sequencing near-identical repeat arrays such as the tandem repeat domains of MUC16. In part because of this technical limitation and the highly repetitive nature of its tandem repeat region, the three-dimensional (3D) structure of the complete MUC16 protein has not been determined. Newer long-read DNA sequencing platforms enable more accurate characterization of repetitive domains of genes such as *MUC16* (15, 16). Long-read DNA sequencing methods—commercialized by PacBio and Oxford Nanopore—were recently used to generate a gap-free human reference genome using a complete hydatiform mole as the source material (17). The goal of that effort was the completion of a high-quality reference genome, not the characterization of genomes or transcriptomes associated with particular disease states. To support ongoing efforts to identify the CA125 epitope, and as one of the steps necessary to identify source-specific glycoproteoforms of MUC16, we chose to leverage third-generation DNA sequencing technologies to clarify the molecular model of CA125 (MUC16).

Here, we report a revised model of the tandem repeat region of CA125 (MUC16) that contains 19 tandem repeats. The MUC16 tandem repeat domain and C-terminus in three human cancer cell lines (OVCAR3, OVCAR5, and Kuramochi) and three patient-derived tumors were sequenced on an Oxford Nanopore platform. The sequences were verified by bottom-up proteomics analyses. The 3D structures of the 19 tandem repeats were predicted by AlphaFold. The model proposed here—comprising 19 units in the tandem repeat domain—should become the standard description of CA125 (MUC16) in the future, replacing the approximately 60 tandem repeat domain model that continues to be cited in the ovarian cancer literature to describe CA125 (MUC16; refs. 10, 18–21). Improved understanding of the structure of CA125 (MUC16) may enable the development of novel therapeutic and diagnostic tools by helping to identify the CA125 epitopes.

## Materials and Methods

### Patient Recruitment

All experiments were approved by the Institutional Review Board of the University of Wisconsin–Madison and conducted in accordance with the U.S. Health and Human Services Basic Policy for Protection of Human Research Subjects. Patients suspected of ovarian cancer were recruited, and written informed consents were obtained from participants. This study investigates tumor tissue collected from 3 patients: OV1 (endometrioid adenocarcinoma), OV2 (high-grade serous ovarian cancer), and OV3 (mixed low-grade serous and endometrioid adenocarcinoma).

### Cytoplasmic RNA Isolation

The identity of three cell lines (OVCAR3, RRID: CVCL_0465; OVCAR5, RRID: CVCL_1628; Kuramochi, RRID: CVCL_1345) was validated by human cell short tandem repeat profiling (ATCC), and cell lines were confirmed to be free of *Mycoplasma* contamination using a PCR kit from Genlantis. Cell lines were cultured in RPMI1640 supplemented with 10% FBS and 2 mmol/L l-glutamine until they reached 100% confluency. Cytoplasmic RNA was isolated from cultured cells using the Invitrogen PARIS kit (Thermo Fisher Scientific) following manufacturer's instructions. Briefly, 10^7^ cells were harvested and lysed by addition of 500 µL Cell Fractionation Buffer. Cell debris and nuclear material were pelleted (500 × *g*) and the cytoplasmic fraction was mixed with an equal volume of 2 × Lysis/Binding Solution and 100% ethanol. The mixture was filtered and RNA was eluted into 50 µL elution buffer and immediately used for RT-PCR.

### Tumor RNA Isolation

Tumors from patients with ovarian cancer (serous and endometrioid adenocarcinoma) were collected and stored at –80°C for later use. Tumors weighing 30–40 mg were placed in 750 µL Invitrogen TRIzol (Thermo Fisher Scientific) and cut into smaller pieces with scissors. To each sample 150 µL of chloroform was added, and samples were mixed for 30 seconds and centrifuged at 20,854 ×  *g* for 15 minutes at 4°C to separate RNA into the aqueous phase. The aqueous phase was mixed with approximately 0.53 × volume of 100% ethanol and transferred to an RNeasy spin column (Qiagen). Total RNA was purified with RNeasy Mini kit (Qiagen) as per manufacturer's protocol with RNAse free DNase (Qiagen) on-column treatment. RNA concentration and integrity were measured on the 2100 Bioanalyzer (Agilent Technologies) using the RNA NanoChip Kit (Agilent).

### cDNA Preparation

A reverse primer targeting the 3′ end of *MUC16* and a forward primer targeting a binding site 600 bps upstream of the tandem repeat region were used for cDNA generation. Primer sequences are found in Table 1. First-strand cDNA generation and cDNA amplification were done in one reaction using the SuperScript IV RT-PCR system (Thermo Fisher Scientific). A total of 1 µg of cytoplasmic RNA was used as template for each reaction. All other reagents were prepared according to the manufacturer's instructions. The reverse transcription reaction was done at 60°C for 10 minutes followed by heating to 98°C for 2 minutes to inactivate RNase. cDNA was amplified immediately after reverse transcription using the following temperature cycling steps: 35 cycles of 98°C for 10 seconds, 64.8°C for 15 seconds, and 72°C for 330 seconds. A final extension was done at 72°C for 5 minutes. The cDNA was separated by 0.5% agarose gel electrophoresis and purified using a gel cleanup kit (Qiagen).

**TABLE 1:** Primer sequences used for RT-PCR

Primer name	Sequence
Forward	TTGGTTTACTAGAGACTACAGGCTTAC
Reverse	TTGCAGATCCTCCAGGTCTAGG

### Sample Preparation for Nanopore Sequencing

cDNA (40 fmol) amplified from five independent reactions of each cell line or tumor sample were combined in equal proportions for Nanopore sequencing. The combined cDNA was prepared by Q20+ ligation sequencing kit (Oxford Nanopore). In brief, 48 µL cDNA was incubated with 3.5 µL NEBNext formalin-fixed paraffin-embedded (FFPE) DNA Repair Buffer, 2 µL NEBNext FFPE DNA Repair Mix, 3.5 µL Ultra II End-prep reaction buffer, and 3 µL Ultra II End-prep enzyme mix (NEB) at 20°C for 5 minutes and 65°C for 5 minutes followed by AMPure XP beads purification (Beckman Coulter). cDNA (60 µL) eluted from AMPure XP bead purification was ligated with sequencing adapters by incubation with 5 µL Adapter Mix H, 10 µL NEBNext Quick T4 DNA Ligase (NEB), and 25 µL Ligation Buffer (Oxford Nanopore) at room temperature for 10 minutes. The cDNA was then purified one additional time by AMPure XP beads with L fragment buffer.

### Nanopore Sequencing and Consensus Sequence Generation

The R10.4 flow cell with K12 chemistry was assembled with MinION sequencer and primed with priming mix following manufacturer's instructions. cDNA (12 µL) was mixed with 37.5 µL sequencing buffer II and 25.5 µL loading solution and immediately loaded into the sample port. The sequencing was run by MinKNOW (RRID: SCR_003756) version 22.03.6 for 5 hours with minimal read length 1,000 bp. Raw signal was base called by guppy base caller version 6.1.2 under superaccuracy mode with read splitting and adapter trimming. Raw reads with average quality score above 20 and read length between 10 and 10.5 kbp were selected as high-quality reads. The read with highest quality score was used as a template and all other high-quality reads were used as the input for consensus generation by Medaka version 1.6.0.

### MUC16 Immunoprecipitation

OVCAR3 cells were grown on a Petri dish, harvested by scraping, and lysed by sonication in RIPA buffer (Thermo Fisher Scientific) with protease inhibitor (Roche Diagnostics). Cell lysate was centrifuged at 16.1 relative centrifugal force (RCF) at 4°C for 30 minutes. Total protein concentration of the supernatant was measured by bicinchoninic acid protein assay (Thermo Fisher Scientific). A total of 1 mg of total protein was mixed with 5 µg anti-CA125 antibody (M11-like, Fitzgerald, catalog no. 10-C02G, clone M61703) and incubated at 4°C overnight. After overnight incubation, 25 µL protein A magnetic beads (Thermo Fisher Scientific) were pre-equilibrated with RIPA buffer and mixed with the reaction. The reaction was incubated at room temperature for 1 hour and the magnetic beads were collected and washed three times with TBS-T buffer (TBS with 0.05% Tween 20) and once with water. Purified MUC16 was eluted by 0.1 mol/L glycine at pH 2.0 and precipitated by acetone for proteomics analysis. MUC16 from commercially available pooled ascites (Fitzgerald) was repurified by the same immunoprecipitation method described above.

### Proteomics Sample Preparation and Mass Spectrometry

A total of 10 µg of total protein eluted from immunoprecipitation (IP) was precipitated in 70% acetone (VWR) at –20°C overnight and resuspended in 100 mmol/L triethyl ammonium bicarbonate (TEAB, Sigma) buffer with 0.2% deoxycholic acid, 6% SDS, and 10 mmol/L tris(2-carboxyethyl)phosphine (all from VWR). Protein was denatured and reduced by incubation at 95°C for 10 minutes. Reduced protein was alkylated with 10 mmol/L iodoacetamide (Sigma) for 30 minutes at room temperature in the dark. The alkylation reaction was quenched by 1.2% phosphoric acid (VWR). The protein solution was spun onto an S-Trap device (Protifi) and digested with 0.75 µg Trypsin Gold (Promega) in 100 mmol/L TEAB buffer at 37°C overnight. Digested peptides were eluted by three elution buffers: 100 mmol/L TEAB, 0.1% formic acid (FA) in water, and 50% acetonitrile with 0.1% FA. Eluted peptides were combined and desalted using C18 ZipTips (Thermo Fisher Scientific). Desalted peptides were reconstituted in water with 4% acetonitrile and 0.5% FA (both from Thermo Fisher Scientific) and analyzed by a Waters NanoAcquity liquid chromatography (LC) system coupled to a Q-Exactive mass spectrometer (Thermo Fisher Scientific). The LC/MS-MS used conditions previously optimized by our group for MUC16 identification (19). Each sample was run in technical triplicate with the following linear gradient, where solvent A was water with 0.1% FA (Burdick & Jackson, VWR) and solvent B was acetonitrile with 0.1% FA: 4% B for 0–10 minutes, 4%–7% B from 10–12 minutes, 7%–31% B from 12–70 minutes, 31%–90% B from 70–74 minutes, 90% B until 78 minutes, 90%–94% B for 1 minutes, and re-equilibration at 4% B from 79–90 minutes. The mass spectrometer settings were identical to those described previously (22).

### Database Searching

Triplicate injections for each IP sample were searched using PEAKS proteomics software *de novo* assisted database search (23) using the following parameters: Precursor Mass Error Tolerance: 10 ppm, Fragment Mass Error Tolerance: 0.02 Da, Enzyme: Trypsin, Fixed Modifications (using one-letter amino acid codes): Carbamidomethylation of C, Variable Modifications: Deamidation of N&Q; Oxidation of M; Pyroglutamic acid formation from E&Q; Sodium Adduct; maximum of 3 missed cleavages; PSM FDR of 1%; Protein −10log*P* of 20 (equivalent to a FDR of 1%). Peptides 6 to 45 amino acids in length were considered. For OVCAR3 IP samples, the database used was the Uniprot Human Protein Database (downloaded July 7, 2022; ref. 24) with the MUC16 entry replaced with the OVCAR3 amino acid sequence predicted by Nanopore sequencing of OVCAR3 RNA as described above. For pooled ascites MUC16 IP samples, the database used was the same as above, but with the MUC16 entry replaced with the consensus sequence created by combining all Nanopore sequencing results as described above. Peptide results files were exported for further data analysis. In addition, data previously collected (19) from a bottom-up proteomics analysis of MUC16 enriched from ascites using an affinity-free method were reanalyzed using the searching method described above for pooled ascites MUC16 IP samples.

### Proteomic Data Analysis

Peptides from the database searches were analyzed using R (version 4.1.3; ref. 25) and the following packages: stringr (26), readxl (27), seqinr (28), dplyr (29). Plots were created with ggplot2 (30). Peptides mapping to MUC16 were extracted, and only peptides unique to MUC16 (not mapping to other proteins in the database) were considered. Peptides that mapped to repeats 8–12 were checked to see whether they mapped to other repeats in MUC16, and if they did not, they were flagged as unique to those five repeats. Coverage maps of the MUC16 peptides were created by plotting the amino acid sequence versus repeat number, and peptides were shown as boxes where they mapped to the sequence, colored by whether they were unique to repeats 8–12. The R script used to perform proteomics data analysis is found as Supplementary Document S1.

### AlphaFold Protein Structure Prediction

AlphaFold structure prediction was performed on each tandem repeat individually and on the entire MUC16 tandem repeat region plus C-terminal domain. The prediction was run using AlphaFold (version 2.3.1) with A100 GPU on the Bigjay cluster at the University of Kansas (Lawrence, Kansas). “--model_preset = monomer”, “--db_preset = full_dbs”, “--max_template_date = 2023-01-01”, and “--use_gpu_relax = True” were set to use monomer model with full database for structure prediction. The top rank relaxed model was colored with pLDDT score and visualized by ChimeraX (version 1.5).

### Data Availability

Nanopore sequencing raw reads are available in the NCBI sequence read archive (BioProject: PRJNA986150). All consensus sequences are available in NCBI Genbank (OV1: OR339648, OV2: OR339649, OV3: OR339650, Kuramochi: OR339651, OVCAR3: OR339652, OVCAR5: OR339653). The mass spectrometry proteomics data have been deposited to the ProteomeXchange Consortium via the PRIDE partner repository with the dataset identifier PXD044117.

## Results

### MUC16 cDNA Preparation

**FIGURE 1:**
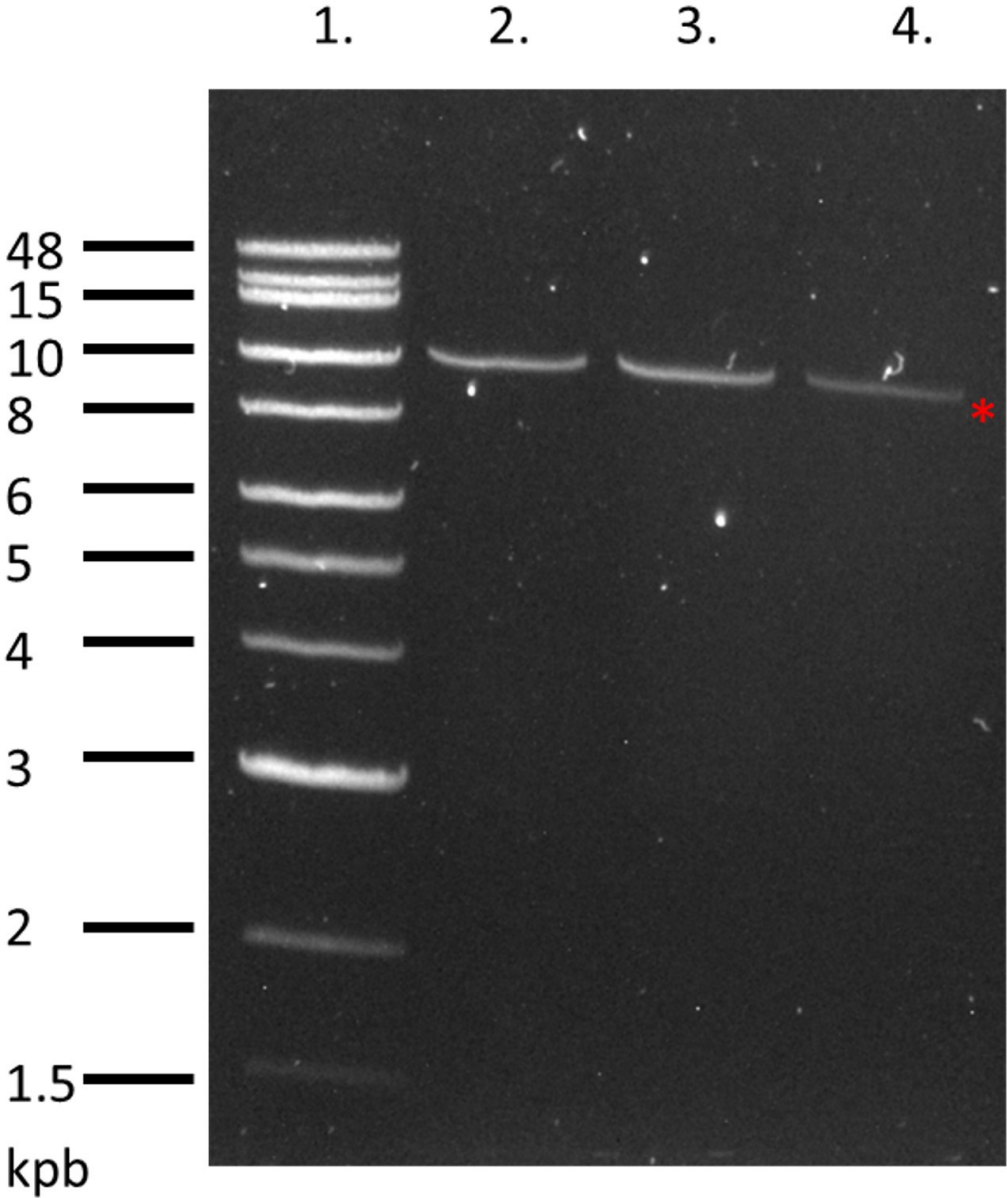
Gel image of cDNA products from RT-PCR. Lane 1: NEB 1 kb Extend DNA Ladder. Lanes 2–4: RT-PCR product from Kuramochi, OVCAR3 and OVCAR5 cells, respectively. The asterisk indicates the location of the approximate 10 kbp cDNA product.

Three cell lines—Kuramochi, OVCAR3, and OVCAR5—were chosen for mRNA sequencing of MUC16. To avoid pre-mRNA contamination, cytoplasmic RNA was isolated from cell culture and used as the template for cDNA generation. Our initial sequencing experiments were designed on the basis of the 63 tandem repeat model (GenBank: AF41442.2) and used three *MUC16* gene-specific primer sets to amplify the tandem repeat region, as shown in Supplementary Fig. S1. Surprisingly, RT-PCR using these primer sets resulted in cDNA that was shorter than expected on the basis of the 63 tandem repeat model. Moreover, the sequence containing a portion of the N-terminus (produced by primer set 1) and the sequence containing a portion of the C-terminus (produced by primer set 3) share a 3 kbp overlap region with 100% agreement. Taken together, these results suggested that the tandem repeat region of *MUC16* is less than 9 kbp and encodes a protein containing only 19 tandem repeats. Therefore, we decided to use only one set of *MUC16* gene-specific primers (Table 1) to amplify the complete tandem repeat region and C-terminus. The reverse primer targeted the 3′ end of MUC16 mRNA, and the forward primer targeted the N-terminal domain, 600 bp upstream of the tandem repeat domain. Primer sequences were designed on the basis of specificity for the target gene and an annealing temperature near 60°C. The amplified cDNA was predicted to contain a short fragment of the N-terminal region, the complete tandem repeat region, and the C-terminal region. RT-PCR products at the expected size, slightly larger than 10 kbp, were observed and purified from all three cell lines. A representative gel image for the three cell lines is shown in Fig. 1. RNA isolated from three patient tumors was reverse-transcribed and amplified using identical RT-PCR conditions. A representative gel image for the three patient samples is shown in Supplementary Fig. S2. Although the DNA polymerase used for the RT-PCR reaction has a low error rate, with fidelity 300 × greater than Taq, the reverse transcriptase does not have proofreading function. To prevent potential errors from RT-PCR from dominating the cDNA library, we combined equal volumes of cDNA from five independent reactions for sequencing.

### Nanopore Sequencing

To achieve high consensus accuracy for our sequence of interest, the K12 chemistry released in 2021 with R10.4 flow cell and Q20+ ligation sequencing was used. The cDNA library from RT-PCR was prepared and sequenced. The raw signals were base called by guppy base caller with superaccuracy mode. More than 100K reads were generated from each cell line. Nanopore sequencing dot plots for the three cell lines and three tumor samples are found in Supplementary Fig. S3. Sequencing raw reads are available in the NCBI sequence read archive (BioProject: PRJNA986150). Raw reads with average quality score greater than 20 and read length between 10 and 10.5 kbps were selected as high-quality reads for consensus sequence generation. We chose the read with highest quality score as a template for consensus generation. The template was then polished by Medaka with more than 10K high-quality reads to generate a consensus sequence with 99.999% minimum accuracy (>Q50).

### Consensus Sequences of MUC16 from Cell Lines and Primary Tumors

The consensus sequences of three cell lines and three primary tumors all contain 10,322 nucleotides that encode 3,440 amino acids containing a small section of the N-terminal domain, the complete tandem repeat domain and the entire C-terminal domain. All consensus sequences are available in NCBI Genbank (OV1: OR339648, OV2: OR339649, OV3: OR339650, Kuramochi: OR339651, OVCAR3: OR339652, OVCAR5: OR339653). These consensus sequences were aligned with the MUC16 isoform 3 mRNA script (NM_001414687.1) that was previously deposited in NCBI to generate an overall consensus sequence. The DNA alignment of the seven input sequences with the overall consensus sequence (labeled “Consensus” on the top line) is shown in Supplementary Document S2. All six consensus sequences (from three cell lines and three primary tumors) have the same structure as the isoform 3 mRNA script, with no insertions, deletions, or frame shifts. Each consensus sequence differs slightly in nucleotide sequence from the overall consensus sequence: 8 nt differences for Kuramochi; 8 nt differences for OVCAR3; 9 nt differences for OVCAR5; 3 nt differences for OV1; 10 nt differences for OV2; and 12 nt differences for OV3. MUC16 isoform 3 differs from the overall consensus sequence at 15 nt. These nucleotide differences result in an alteration of the encoded amino acid as follows: 8 aa differences for MUC16 isoform 3; 3 aa differences for Kuramochi; 4 aa differences for OVCAR3; 5 aa differences for OVCAR5; no aa differences for OV1; 8 aa differences for OV2; and 9 aa differences for OV3. The protein alignment of the seven input sequences with the overall consensus sequence is found in Supplementary Document S3.

### Proteomic Confirmation

**FIGURE 2:**
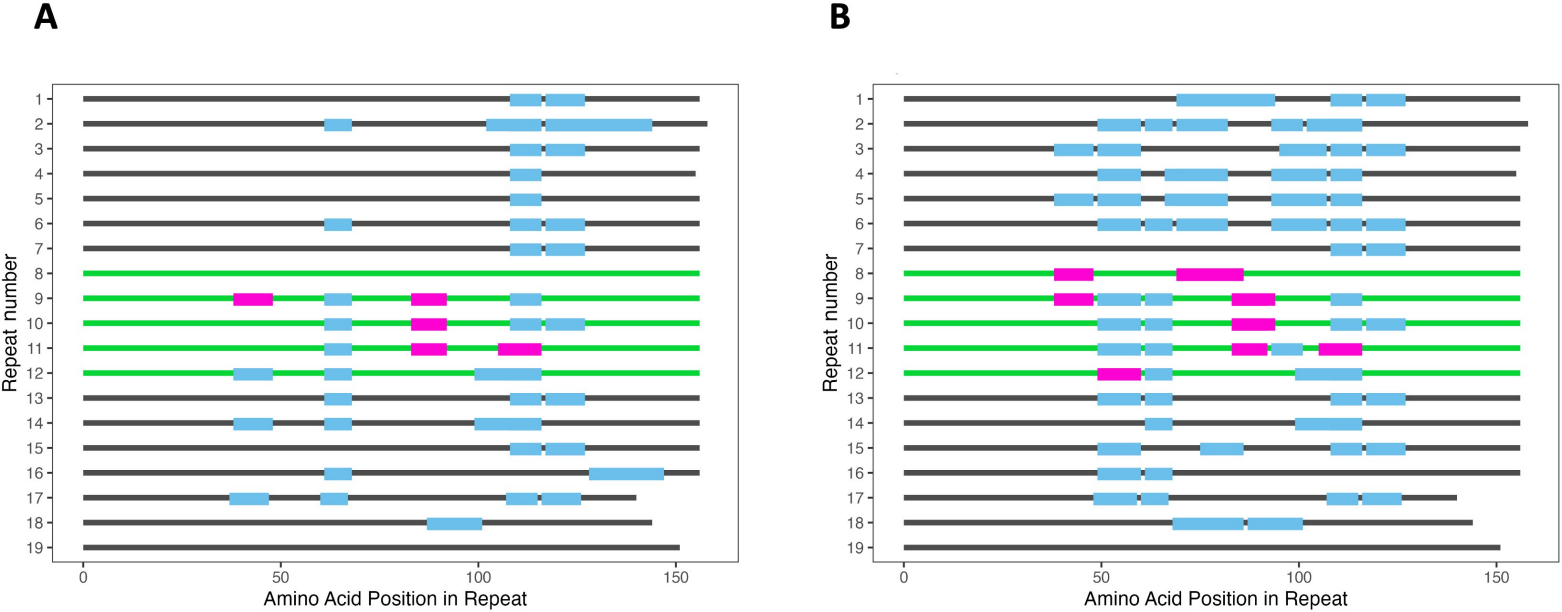
Proteomic analysis of immunoprecipitated MUC16. Identified MUC16 peptides from immunoprecipitated (**A**) OVCAR3 cell lysate and (**B**) pooled ascites mapped onto the proposed repeat domains of MUC16. The repeat domains are each represented by a horizontal line, and the amino acid position within the repeat is on the *x*-axis. Repeats shown in green (8--12) are the five repeats not included in the NM_024690.2 sequence (MUC16 isoform 4). Rectangles represent peptides, where the pink peptides are unique to repeats 8–12 (map to nowhere else in MUC16) and blue peptides are not unique to repeats 8–12. All peptides shown are unique to MUC16 (map to nowhere else in the proteome).

The other MUC16 mRNA script deposited in NCBI as of November 2023 is MUC16 isoform 4 mRNA (NM_024690.2). Isoform 4 contains 2,340 fewer nucleotides than isoform 3 and all six consensus sequences that we obtained through long-read sequencing. This difference of 2,340 nucleotides corresponds to five fewer tandem repeats in isoform 4. Because cytoplasmic RNA was used as the input for our RT-PCR and sequencing, it is not likely that these 2,340 nucleotides are contained in an intron or pre-mRNA. To confirm that the 2,340 nucleotides, coding for five tandem repeats, that appear in our consensus sequence and isoform 3 (but not isoform 4) are an exon that is actively translated, we performed bottom-up proteomics analysis. MUC16 protein from OVCAR3 cells and from pooled patient ascites was purified by immunoprecipitation. MUC16 was pulled down by anti-CA125 antibody and protein A beads, followed by trypsin digestion and LC/MS-MS analysis. For the OVCAR3 sample, we searched the tandem mass spectrometry results against the newly generated OVCAR3 consensus sequence. For the pooled ascites sample, we searched the tandem mass spectrometry results against the overall consensus sequence. In the OVCAR3 sample, we identified 10 peptides from the five tandem repeats, resulting in 18% coverage. Five of the identified peptides were unique to this region (Fig. 2A). In the pooled ascites sample, we identified 17 peptides from the five tandem repeats, resulting in 28% coverage. Ten of the identified peptides were unique to this region (Fig. 2B). These unique peptides do not appear elsewhere in MUC16, or the rest of the proteome database, leading us to conclude that they originate from this region of MUC16. In addition, data collected on MUC16 enriched from the ascites of three individual patients using an immunoaffinity-free method in our previous study (19) were reanalyzed using the same method as the pooled ascites sample. In three patient-derived samples, MUC16 peptides from the five tandem repeats were detected. Percent coverage of the five tandem repeats ranged from a minimum of 31% from 15 peptides to a maximum of 45% from 25 peptides. Seven to 16 of the identified peptides were unique to the five tandem repeats. These data support the conclusion that the 2,340 nucleotides are both transcribed and translated. A coverage map of immunoaffinity-free enriched MUC16 is found in Supplementary Fig. S4.

**FIGURE 3:**
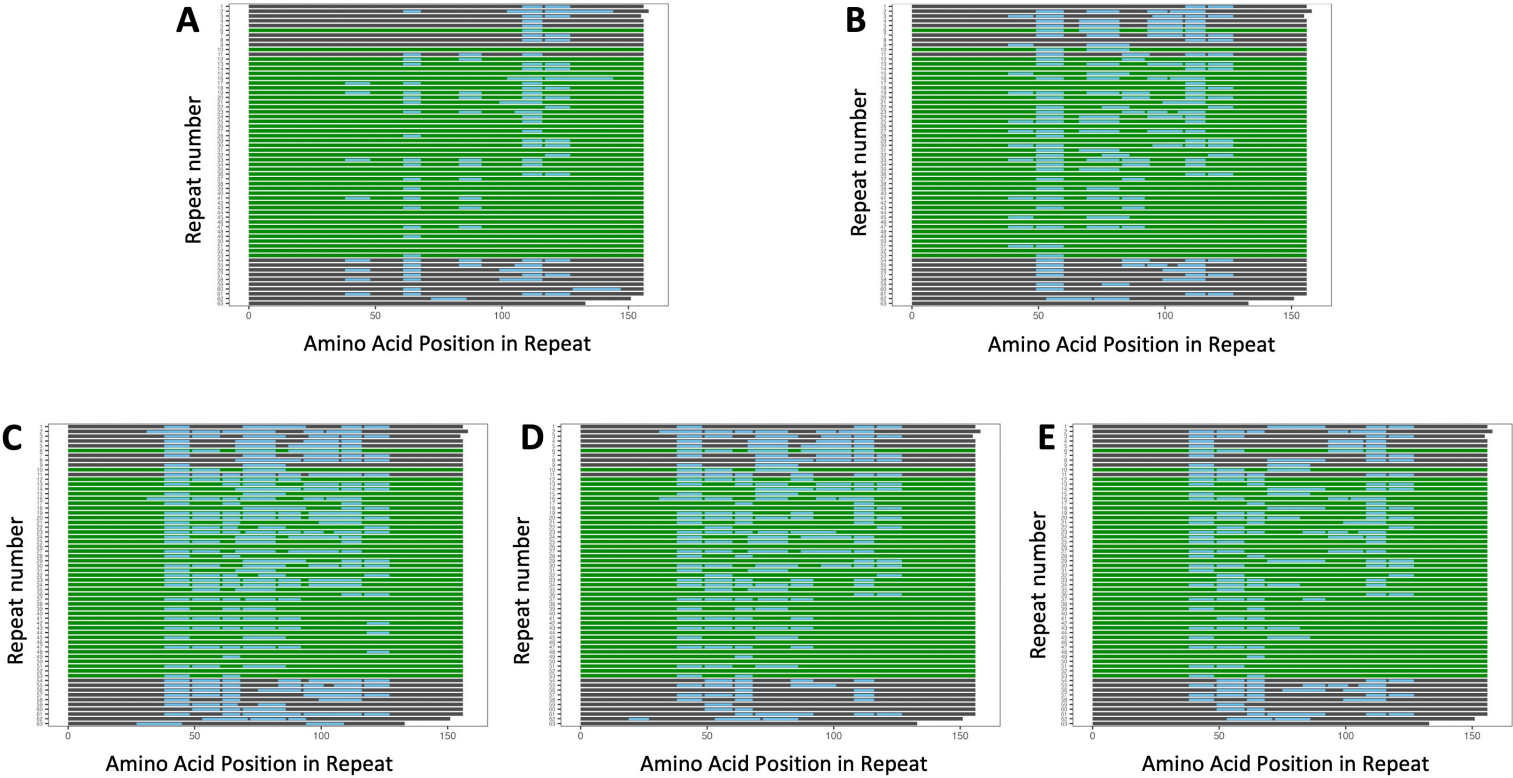
Proteomic analysis of immunoprecipitated and patient-derived samples. Identified MUC16 peptides from immunoprecipitated (**A**) OVCAR3 cell lysate and (**B**) pooled ascites mapped onto the 63 tandem repeat model. Identified MUC16 peptides from patient-derived ascites (**C**) Patient 1, (**D**) Patient 2, and (**E**) Patient 3 mapped onto the 63 tandem repeat model. The repeat domains are each represented by a horizontal line, and the amino acid position within the repeat is on the *x*-axis. Repeats shown in green (6, 10, 12--53) are the 44 repeats in the 63 tandem repeat model, but not our 19 tandem repeat model. No peptides are identified to be unique to repeats 6, 10, 12–53. All peptides shown are unique to MUC16 (map to nowhere else in the proteome).

Mass spectrometry data from five proteomics samples were also searched against the MUC16 model with 63 tandem repeats. In the analyses of all five samples, no peptides were identified that map uniquely to the 44 repeats found in the 63-repeat model but not in our model (Fig. 3). The observation that the extra tandem repeats found in the 63-repeat model are not detected in protein digests of MUC16 from IP pulldown is not conclusive evidence but supports our long-read sequence and resulting molecular model.

### New Proposed Molecular Model of MUC16

An amino acid consensus sequence from all seven sources of MUC16 was generated using the MUSCLE algorithm (Supplementary Document S3). The consensus sequence contains 19 tandem repeats. An alignment of these 19 repeats is shown in Supplementary Fig. S5. Repeats 1–17 have high sequence similarity, while repeats 18 and 19 are more variable. Each tandem repeat contains two cysteine residues (Cys59 and Cys79) that in oxidizing conditions could engage in intramolecular disulfide bonding to form a “C-loop” with 19 intervening amino acids (31). These Cys residues are completely conserved. We propose a new numbering system for these 19 repeats (Table 2).

**TABLE 2:** The amino acid consensus sequence derived from long-read cDNA sequencing of six sources of MUC16 mRNA

Repeat	Amino Acid Sequence
R1	ATVPFMVPFTLNFTITNLQYEEDMRHPGSRKFNATERELQGLLKPLFRNSSLEYLYSGCRLASLRPEKDSSATAVDAICTHRPDPEDLGLDRERLYWE LSNLTNGIQELGPYTLDRNSLYVNGFTHRSSMPTTSTPGTSTVDVGTSGTPSSSPSPT
R2	TAGPLLMPFTLNFTITNLQYEEDMRRTGSRKFNTMESVLQGLLKPLFKNTSVGPLYSGCRLTLLRPEKDGAATGVDAICTHRLDPKSPGLNREQLY WELSKLTNDIEELGPYTLDRNSLYVNGFTHQSSVSTTSTPGTSTVDLRTSGTPSSLSSPTIM
R3	AAGPLLVPFTLNFTITNLQYGEDMGHPGSRKFNTTERVLQGLLGPIFKNTSVGPLYSGCRLTSLRSEKDGAATGVDAICIHHLDPKSPGLNRERLYW ELSQLTNGIKELGPYTLDRNSLYVNGFTHRTSVPTTSTPGTSTVDLGTSGTPFSLPSPA
R4	TAGPLLVLFTLNFTITNLKYEEDMHRPGSRKFNTTERVLQTLLGPMFKNTSVGLLYSGCRLTLLRSEKDGAATGVDAICTHRLDPKSPGVDREQLY WELSQLTNGIKELGPYTLDRNSLYVNGFTHWIPVPTSSTPGTSTVDLGSGTPSSLPSPT
R5	TAGPLLVPFTLNFTITNLKYEEDMHCPGSRKFNTTERVLQSLLGPMFKNTSVGPLYSGCRLTLLRSEKDGAATGVDAICTHRLDPKSPGVDREQLY WELSQLTNGIKELGPYTLDRNSLYVNGFTHQTSAPNTSTPGTSTVDLGTSGTPSSLPSPT
R6	SAGPLLVPFTLNFTITNLQYEEDMHHPGSRKFNTTERVLQGLLGPMFKNTSVGLLYSGCRLTLLRPEKNGAATGMDAICSHRLDPKSPGLNREQLY WELSQLTHGIKELGPYTLDRNSLYVNGFTHRSSVAPTSTPGTSTVDLGTSGTPSSLPSPT
R7	TAVPLLVPFTLNFTITNLQYGEDMRHPGSRKFNTTERVLQGLLGPLFKNSSVGPLYSGCRLISLRSEKDGAATGVDAICTHHLNPQSPGLDREQLY WQLSQMTNGIKELGPYTLDRNSLYVNGFTHRSSGLTTSTPWTSTVDLGTSGTPSPVPSPT
R8	TAGPLLVPFTLNFTITNLQYEEDMHRPGSRKFNTTERVLQGLLSPIFKNSSVGPLYSGCRLTSLRPEKDGAATGMDAVCLYHPNPKRPGLDREQLY WELSQLTHNITELGPYSLDRDSLYVNGFTHQNSVPTTSTPGTSTVYWATTGTPSSFPGHT
R9	EPGPLLIPFTFNFTITNLHYEENMQHPGSRKFNTTERVLQGLLTPLFKNTSVGPLYSGCRLTLLRPEKHEAATGVDTICTHRVDPIGPGLDRERLY WELSQLTNSITELGPYTLDRDSLYVNGFNPWSSVPTTSTPGTSTVHLATSGTPSSLPGHT
R10	APVPLLIPFTLNFTITNLHYEENMQHPGSRKFNTTERVLQGLLKPLFKSTSVGPLYSGCRLTLLRPEKHGAATGVDAICTLRLDPTGPGLDRERLY WELSQLTNSVTELGPYTLDRDSLYVNGFTHRSSVPTTSIPGTSAVHLETSGTPASLPGHT
R11	APGPLLVPFTLNFTITNLQYEEDMRHPGSRKFNTTERVLQGLLKPLFKSTSVGPLYSGCRLTLLRPEKRGAATGVDTICTHRLDPLNPGLDREQLY WELSKLTRGIIELGPYLLDRGSLYVNGFTHRNFVPITSTPGTSTVHLGTSETPSSLPRPI
R12	VPGPLLVPFTLNFTITNLQYEEAMRHPGSRKFNTTERVLQGLLRPLFKNTSIGPLYSSCRLTLLRPEKDKAATRVDAICTHHPDPQSPGLNREQLY WELSQLTHGITELGPYTLDRDSLYVDGFTHWSPIPTTSTPGTSIVNLGTSGIPPSLPETT
R13	ATGPLLVPFTLNFTITNLQYEENMGHPGSRKFNITESVLQGLLKPLFKSTSVGPLYSGCRLTLLRPEKDGVATRVDAICTHRPDPKIPGLDRQQLY WELSQLTHSITELGPYTLDRDSLYVNGFTQRSSVPTTSTPGTFTVQPETSETPSSLPGPT
R14	ATGPVLLPFTLNFTIINLQYEEDMHRPGSRKFNTTERVLQGLLMPLFKNTSVSSLYSGCRLTLLRPEKDGAATRVDAVCTHRPDPKSPGLDRERLY WKLSQLTHGITELGPYTLDRHSLYVNGFTHQSSMTTTRTPDTSTMHLATSRTPASLSGPT
R15	TASPLLVLFTINFTITNLRYEENMHHPGSRKFNTTERVLQGLLRPVFKNTSVGPLYSGCRLTLLRPKKDGAATKVDAICTYRPDPKSPGLDREQLY WELSQLTHSITELGPYTLDRDSLYVNGFTQRSSVPTTSIPGTPTVDLGTSGTPVSKPGPS
R16	AASPLLVLFTLNFTITNLRYEENMQHPGSRKFNTTERVLQGLLRSLFKSTSVGPLYSGCRLTLLRPEKDGTATGVDAICTHHPDPKSPRLDREQLY WELSQLTHNITELGPYALDNDSLFVNGFTHRSSVSTTSTPGTPTVYLGASKTPASIFGPS
R17	AASHLLILFTLNFTITNLRYEENMWPGSRKFNTTERVLQGLLRPLFKNTSVGPLYSGCRLTLLRPEKDGEATGVDAICTHRPDPTGPGLDREQLY LELSQLTHSITELGPYTLDRDSLYVNGFTHRSSVPTTSTGVVSEE
R18	PFTLNFTINNLRYMADMGQPGSLKFNITDNVMQHLLSPLFQRSSLGARYTGCRVIALRSVKNGAETRVDLLCTYLQPLSGPGLPIKQVFHELSQQTH GITRLGPYSLDKDSLYLNGYNEPGPDEPPTTPKPATTFLPPLSEATT
R19	AMGYHLKTLTLNFTISNLQYSPDMGKGSATFNSTEGVLQHLLRPLFQKSSMGPFYLGCQLISLRPEKDGAATGVDTTCTYHPDPVGPGLDIQQLY WELSQLTHGVTQLGFYVLDRDSLFINGYAPQNLSIRGEYQINFHIVNWNLSNPDPT

**FIGURE 4:**
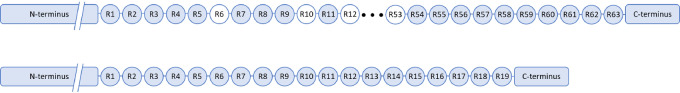
Schematic models of MUC16. Top, The model derived from the sequence determined by O'Brien and colleagues (2001) containing 63 units in the tandem repeat domain. Bottom, The model proposed in this study, containing 19 units in the tandem repeat domain. The repeats that are not included in the proposed model (6, 10, and 12 through 53) are shown as white circles. Ellipses represent repeats that are not shown for clarity. The N-terminal domain is much larger than shown here and is also compressed for clarity.

**FIGURE 5:**
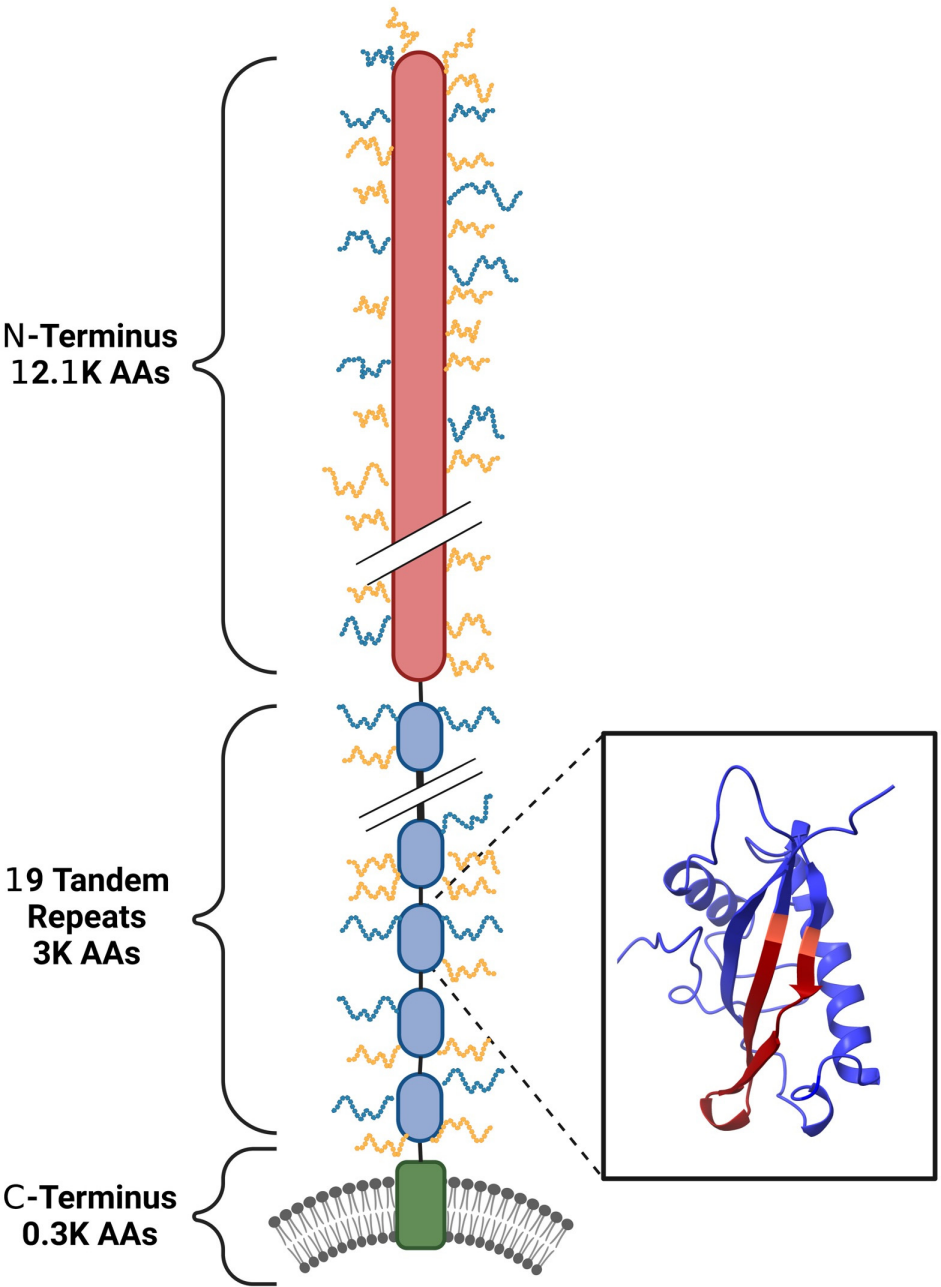
MUC16 model proposed in this study. The model includes: a highly glycosylated N-terminal domain, a 19-unit tandem repeat domain that contains the CA125 epitopes, and a short C-terminal domain that includes a membrane-spanning region. The inset shows a representative 3D structure of one tandem repeat domain, predicted by AlphaFold. The C-loop (residues 59–79) is shown in red, with the cysteine residues shown in salmon. The unstructured proline/serine/threonine-rich region is omitted for clarity. White slashes within the N-terminal domain and tandem repeat domain show locations where amino acids are not explicitly shown for clarity. Figure created with https://Biorender.com.

Comparing our MUC16 sequence with the 63-repeat version (AF414442.2), we note that repeats 6, 10, and 12 through 53 in the 63-repeat version do not appear in our consensus sequence. Figure 4 shows a simple schematic comparison of the 63-repeat MUC16 model with the model proposed here. There are 8 repeats in our version that are identical to the 63-repeat version, while the others differ by fewer than two amino acids. Supplementary Figure S6 compares the model proposed in this study to the model predicted on the basis of the sequence reported by O'Brien and co-workers. We attribute the difference in the number of tandem repeat domains to the use of primer walking and assembly of short reads used to make the 63-repeat model. Figure 5 presents a more detailed model of MUC16 that is consistent with the new consensus sequence.

### AlphaFold Protein Structure Prediction

**FIGURE 6:**
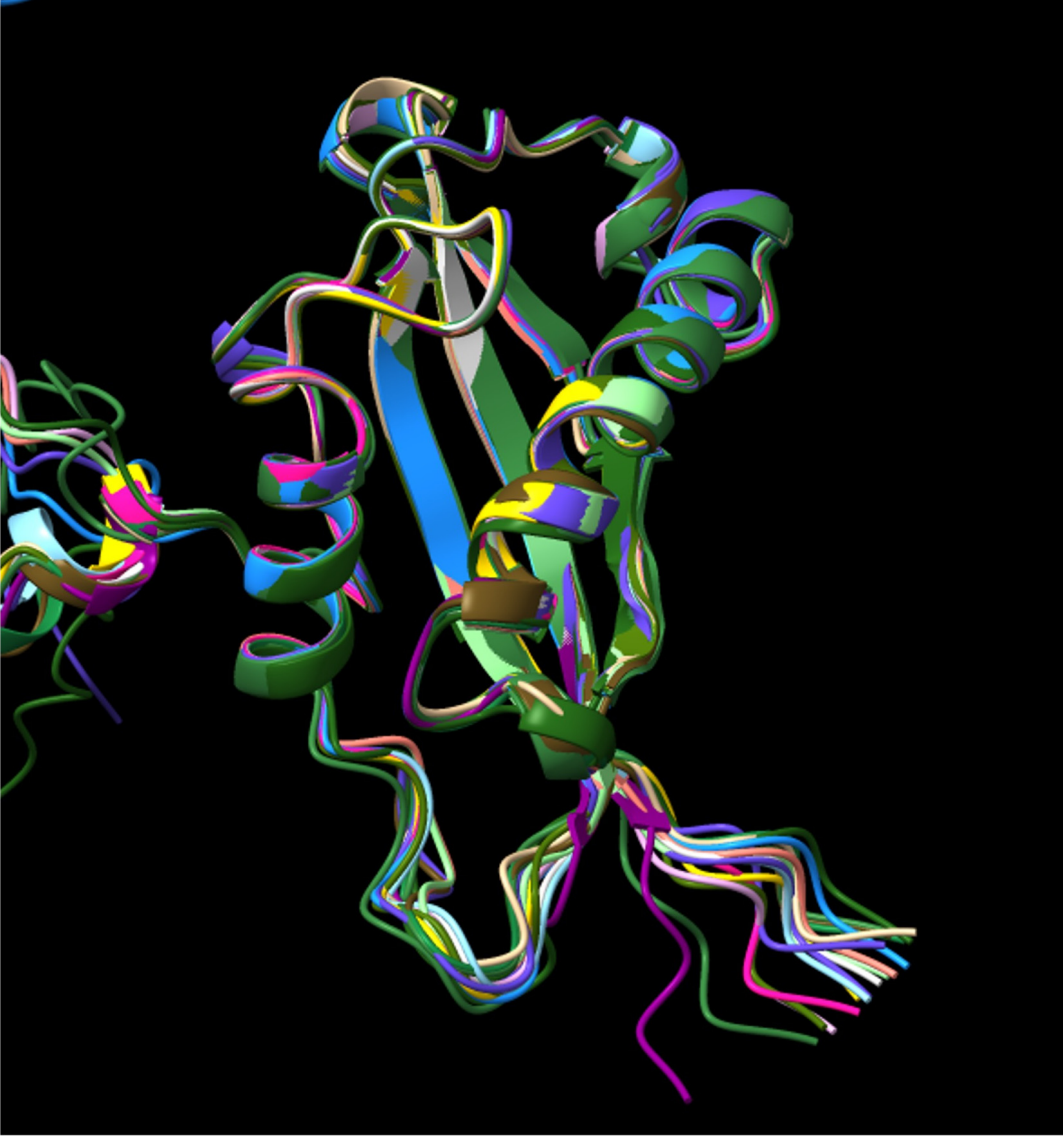
AlphaFold predicted MUC16 tandem repeat model. The 19 tandem repeat structures were predicted individually and overlaid. All repeats contain a similar structure including two alpha helices, one beta sheet, and a proline/serine/threonine-rich disordered region (this region is not predicted with high confidence and is omitted for clarity).

To date, there is no experimentally determined structure of intact MUC16. A major advance in MUC16 structural characterization was achieved in 2022, with the publication of a crystal structure of the SEA domain of one tandem repeat (21). To better understand the structure of the MUC16 tandem repeat domain, we used AlphaFold to predict the structure of each tandem repeat. AlphaFold is a high-accuracy protein structure prediction software developed by the DeepMind group. (32) We used its monomer model with full database to create five predicted models. The five predicted models of all repeats have highly similar structure. Top-ranked models of individual tandem repeats predicted by AlphaFold are shown in an overlaid plot in Fig. 6 and as individual images in Supplementary Fig. S7A–S7S. Each tandem repeat contains two alpha helices and one beta sheet that match the MUC16 SEA5 domain structure solved by diffraction methods. This structural similarity is notable because AlphaFold does not require templates for generating predicted structures. Use of a templating protein structure prediction tool [i-Tasser (33–35)] gives results indistinguishable from those predicted by AlphaFold (Supplementary Fig. S8). The SEA domain predicted by AlphaFold is also in good agreement with the nuclear magnetic resonance solution structure of a murine SEA domain (ref. 36; Supplementary Fig. S9).

## Discussion

The characterization of the molecule bearing the CA125 epitopes has been an area of active interest in the ovarian cancer field since CA125 was first reported as a biomarker by Bast and co-workers in the early 1980s (37, 38). In 2001 and 2002, publications from two independent research teams advanced the field considerably. Yin and Lloyd reported the isolation of a long, but partial, cDNA corresponding to the CA125 antigen which enabled the identification of MUC16 as a new mucin that carried the CA125 epitope. The deduced amino acid sequence reported in 2001 by Yin and Lloyd contains nine partially conserved tandem repeats of 156 amino acids each. The assignment was confirmed in a follow-up study, by transfecting a partial MUC16 cDNA into two CA125-negative cell lines and observing that CA125 expression resulted (14).

Also in 2001 and 2002, two articles were published by O'Brien and co-workers, the first describing their work on cloning the CA125 gene and the second extending the size of the glycosylated N-terminal domain (11, 13). In the 2001 report, repeat sequences were placed in order using regions of overlap from cloned 400 bp PCR products. The authors remark that “there is some potential redundancy and we have evidence that some repeats exist in more than one location in the sequence giving a total of 60+ repeats in the CA125 molecule” (11). This statement was the origin of the 63 tandem repeat model that has been reported in the ovarian cancer literature for two decades. Examination of the complete list of repeat sequences (Table 1 in ref. 11) reveals that 28 out of 61 repeats have uncalled bases, represented by “X” in the sequences. These include R12, R17, R18, R24, R26, R28, R29, R31–R44, R46–R53, and R57. There is high correlation between the set of repeats in the 2001 O'Brien publication with uncalled bases and the set of repeats excluded from the 19 tandem repeat model proposed here. We suggest that the high number of unassigned bases, along with the relatively short lengths (400 bp) of the PCR products used to assemble the 60+ tandem repeat sequence, are responsible for the difference between that model and the shorter molecular model reported here. It is crucial to emphasize that the technical innovation of long-read sequencing enabled cDNA transcripts from MUC16 mRNA of approximately 10,000 bp to be sequenced in a single pass through the Nanopore, and many long transcripts were sequenced in parallel through the array of pores. These considerations provide confidence in the validity of the 19 tandem repeat model that we propose.

**FIGURE 7:**
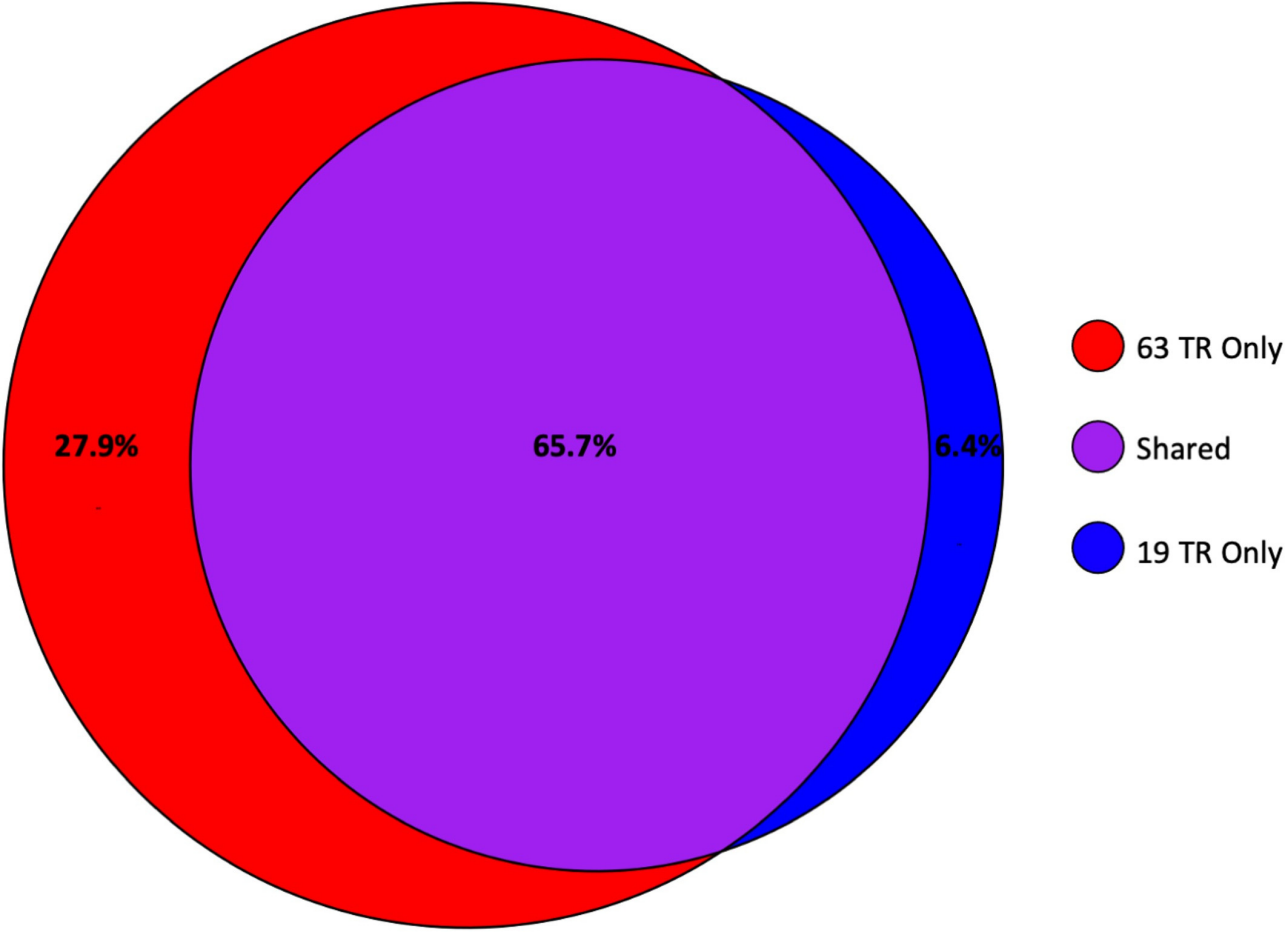
Venn diagram comparing peptides from two MUC16 models. An *in silico* tryptic digest was done of the 63 and 19 tandem repeat models of MUC16. The Venn diagram compares the peptides from the two models that would be detectable by mass spectrometry.

The major finding in this study is that MUC16 from six sources (three cancer cell lines and three ovarian tumors) contains 19 tandem repeats, rather than 63 as was previously reported. Evidence supporting this structural model comes from transcriptomics and from proteomics, which are independent methods of molecular characterization. Regarding transcriptomics, three primer sets (shown in Supplementary Fig. S1) that were designed to cover the 63 tandem repeat region were found to amplify a transcript shorter than expected, with perfect overlap between the products of two primer sets that were expected to yield the extrema of the tandem repeat region. This observation prompted the use of a single primer set (Table 1) that yielded a transcript of similar size (∼10 kbp) from all cell lines and patient samples analyzed. Further corroborating evidence comes from proteomics. First, we detect no peptides that map uniquely to the 44 tandem repeats that appear in the 63 tandem repeat model but not in the 19 tandem repeat model. Although the 19 tandem repeat model is supported by this observation, it is difficult to reach a conclusion based on the absence of an observation. To address this concern, we performed an *in silico* digest of the 63 tandem repeat sequence and of the 19 tandem repeat sequence and made Venn diagrams comparing the detected peptides (Fig. 7). Among the peptides, 65.7% were common to both the 63-repeat and 19-repeat sequence; 27.9% were unique to the 63-repeat sequence; and 6.4% were unique to the 19-repeat sequence. We searched for peptides unique to the 63-repeat sequence in eight datasets collected from patient-derived ascites (a biofluid with high concentrations of MUC16). In none of the eight datasets did a peptide “unique to the 63” appear. Further corroborating evidence for our model comes from consideration of the coverage typically attained in bottom-up proteomics of MUC16 tandem repeats. Among the 19 tandem repeats from MUC16 isolated from pooled ascites, the average peptide coverage is 55% when peptides of size amenable to detection with our mass spectrometer are considered. It is therefore more likely than not that mass spectrometry would have detected peptides derived from the 44 “missing” repeats. While no one piece of data is conclusive, taken together, they provide additional support for our claim that the 19 tandem repeat structure is correct.

None of the input samples yielded sequences that were identical (Supplementary Fig. S5). Each sequence also differs from a deposited MUC16 sequence (isoform 3, NM_001414687.1) at a small number of amino acids. Supplementary Document S4 compares the sequences from three cell lines and three ovarian tumors to MUC16 isoform 3. Kuramochi differs from isoform 3 at 6 amino acids; OVCAR3 differs from isoform 3 at 7 amino acids; OVCAR5 differs from isoform 3 at 12 amino acids; and the three patient tumor samples differ from isoform 3 at 7, 12, and 12 amino acids, respectively. We note that the amino acid differences are not clustered as might be expected in mutations, but are distributed among repeat numbers from 1 to 18 and among the positions within the repeats from 7 to 156. Amino acid differences are found in both the SEA domain proposed to contain the CA125 epitopes and in the unstructured P/S/T rich region. In no case does the amino acid difference add or remove a possible site for N-linked glycosylation. An equal number of additions (A13216T, M14274T, P14668T) and deletions (T14134I, T12162M, T13382K) of possible O-linked glycosylation sites are observed in multiple samples. Among the amino acid differences reported in Supplementary Document S4, some are unique to one cell line (L12828P in Kuramochi; R13142W and V14786M in OVCAR3; R12216Q and E12963K in OVCAR5). Some are unique to one patient sample (T12162M, E12312K, and P14668T in OV2; R12588W, R13754H; V13969M, and Q14759K in OV3). Seven amino acid differences (A13216T, Q13407H, T14134I, M14274T, W13279C, T13382K, W13465R) are found in multiple cell lines and/or patient samples. Three amino acid differences from isoform 3 are shared among all six samples (S12536T, V13444I, R14143H). We note that among the cell lines studied, the largest number of amino acid differences from deposited MUC16 isoform 3 is found in OVCAR5, which was originally identified as ovarian in nature, having been derived from an untreated patient with ovarian cancer (39) but later identified through gene expression compositional analysis to be gastrointestinal in origin (40). MUC16 isoform 3 is a reference sequence, curated by NCBI staff, derived from two complete mappings of *Homo sapiens* chromosome 19 (May 2002 and November 2002) and two whole-genome shotgun sequencing datasets (July 2013 and August 2018). MUC16 isoform 4 has more sources, including a sequence deposited by Yin and Lloyd (21,112 pb, 7,037 aa), a sequence deposited by O'Brien (55,765 bp mRNA, 22,255 aa), and deposits from uterus tissue (3613 bp mRNA), lung tumor (245 bp mRNA), trachea (5,459 bp mRNA), and cervical cancer cell line (578 bp mRNA). This wide range of mRNA transcript size, which may derive from differences in sample preparation, has contributed to the confusion over the molecular nature of MUC16 as expressed by different tissue types, including ovarian tumors.

The three patient samples used for long-read sequencing and analysis derive from patients with MUC16-expressing tumors. The serum CA125 levels from these patients were 474, 1,135, and 652 U/mL, respectively, at the time of surgery. In healthy women, by contrast, CA125 serum levels are below 35 U/mL. The high amount of CA125 detected in the serum indicates that tumors from these patients express MUC16 and are therefore appropriate to be included for MUC16 transcript analysis. We note that MUC16 is not only expressed by ovarian cancer cells. CA125 is also a proven or potential biomarker in endometriosis (41), urothelial carcinoma of the bladder (42), lung cancer (43), gastric cancer (44), and colorectal cancer (45, 46). MUC16 from different sources may differ from the overall consensus sequence reported here. Additional investigation should be undertaken to compare the proteoforms of MUC16 relevant to these diseases with the model derived here. The characterization tools used in this study (long-read mRNA sequencing and bottom-up proteomics) will enable us and others to identify structural features of MUC16 that are most relevant for differential diagnosis and will aid in the development of multiplexed assays that may be amenable for clinical screening.

Efforts to identify the epitopes of CA125 have been ongoing since shortly after the biomarker was first reported but have not yet reached a successful conclusion. Most epitope characterization efforts have taken an (understandably) reductionistic approach, testing the antibody-binding behavior of individual tandem repeats (8, 47), portions of two subdomains and the intervening linkers (9) or conserved subdomains of individual tandem repeats (31). As demonstrated by our group and others (8, 10), different tandem repeats of MUC16 are recognized to different extents by CA125 antibodies. The model that we propose here—derived from high-quality long-read Nanopore sequencing and supported by proteomics—should be referred to as the source of sequence and numbering/naming in future epitope mapping and identification efforts. It is probable that with only 19 tandem repeat domains—rather than 63 as was previously thought—as possible binding locations, the epitopes of CA125 will soon be defined. Efforts in our group remain directed toward this objective. An additional benefit of the revised model reported here is its use in improving and informing studies of the biological function of MUC16 in health and disease. The interaction of MUC16 with mesothelin has been shown to mediate cell adhesion (48) and facilitate peritoneal metastasis of ovarian tumors (49). Agents targeting MUC16/mesothelin interactions—such as TRAIL ligands, single-chain mAbs, nanobodies, or immunoadhesins—may be useful tools in the efforts to prevent intraperitoneal metastasis (50). Drug design and molecular docking studies directed toward the development of MUC16/mesothelin targeting agents should use the corrected molecular model of MUC16 reported here. Design of novel affinity reagents such as aptamers (51, 52) and antibodies (18, 53) for diagnostic and therapeutic applications will also benefit from the use of the revised and corrected MUC16 structural model reported here.

## Conclusions

In this study, we report the results of long-read sequencing of mRNA from three cancer cell lines and three ovarian tumors which enabled us to propose a revised molecular model of MUC16. While sequencing mRNA from ovarian tumors, we noted shorter cDNA transcripts in the samples from one patient (OV1). These shorter transcripts coded for versions of MUC16 with fewer than 19 tandem repeats. The observation of MUC16 proteforms that differ by the deletion of entire tandem repeats is supported by the existence of a similarly truncated sequence (MUC16 isoform 4) deposited in the NCBI database. These shorter sequences may result from alternative splicing, but more patient samples will need to be sequenced to support or refute the hypothesis that alternative splicing of MUC16 occurs in ovarian tumors. In addition to more thoroughly investigating the possibility of tumor-specific splice variant formation, our ongoing efforts include identifying the amino acid sequences that form the CA125 epitopes, developing CA125-specific affinity agents that complement the antibodies used in the clinical assay, and elucidating the cellular processes behind MUC16’s role in immune evasion and metastasis. An accurate molecular model of CA125 (MUC16) will support these efforts and is hoped to bring the realization of improved molecular tools for ovarian cancer management into being.

## Supplementary Material

Figure S1 Set of three primer sequences originally developed for RT-PCR. The three primer sets were found to be redundant, which suggested that the tandem repeat region is shorter than that reported in AF414442.2.

Supplementary Document 1 R script used for proteomics data analysis

Figure S2 Gel image of cDNA products from RT-PCR from patient tumors. (A) Lanes 1-3: NEB 1 kb Extend DNA Ladder, RT-PCR product of OV2 and OV3. Asterisk indicates the location of the approximate 10 kbp cDNA product. (B) Lane 1: NEB 1 kb DNA Ladder. Lane 2: RT-PCR product of OV1. Asterisk indicates the location of the approximate 10 kbp cDNA product.

Supplementary Document 2 DNA alignment

Figure S3 Nanopore sequencing dot plots. The sequencing dot plots show the read length and average read quality. Each dot represents a single read. (A) Kuramochi, (B) OVCAR3, (C) OVCAR5, (D) OV1, (E) OV2, (F) OV3. Note that the axes for each plot are optimized for that plot’s data.

Supplementary Document 3 Protein alignment

Figure S4 Proteomic analysis of immunoaffinity-free enriched MUC16. Identified MUC16 peptides from ascites-derived MUC16 from three patients (A) Patient 1, (B) Patient 2, (C) Patient 3. The repeat domains are each represented by a horizontal line, and the amino acid position within the repeat is on the x-axis. Repeats shown in green (8-12) are the five repeats not included in the NM_024690.2 sequence (MUC16 isoform 4). Rectangles represent peptides, where the pink peptides are unique to repeats 8-12 (map to nowhere else in MUC16) and blue peptides are not unique to repeats 8-12. All peptides shown are unique to MUC16 (map to nowhere else in the proteome).

Supplementary Document 4 Mutation table

Figure S5 Clustal Omega alignment of the 19 tandem repeats. The tandem repeats were aligned using Clustal Omega and visualized using Jalview with the Clustal Omega standard color scheme. The consensus sequence shows abundance of each amino acid at each position.

Figure S6 Comparison of the sequence reported in this study to the sequence reported by O’Brien and co-workers (AF414442.2). Tandem repeats were aligned using Clustal Omega and visualized using Jalview with the Clustal Omega standard color scheme. Tandem repeats are shown in pairs, with AF414442.2 above and our reported sequence below. The consensus sequence shows abundance of each amino acid at each position.

Figure S7 AlphaFold predicted models of individual tandem repeats. (A)-(S) Repeats 1-19.

Figure S8 Comparison of AlphaFold and I-TASSER predicted MUC16 tandem repeat model. The repeat 6 structure predicted by AlphaFold (brown) and I-TASSER (blue) are overlayed. The SEA domains predicted by the two algorithms show high similarity.

Figure S9 Comparison of AlphaFold predicted MUC16 tandem repeat and murine SEA domain (PDB 1IVZ, reference 36). The repeat 6 structure predicted by AlphaFold (brown) and NMR solution structure of a murine SEA domain (gray) are overlayed. The SEA domain predicted by AlphaFold shows high similarity with the murine SEA domain.
