# Supplementary figures and images for: A Revised Molecular Model of Ovarian Cancer Biomarker CA125 (MUC16) Enabled by Long-read Sequencing
Source: Cancer Res Commun. 2024 Jan 31;4(1):253–63. doi: 10.1158/2767-9764.CRC-23-0327 (PMC10829539; doi:10.1158/2767-9764.CRC-23-0327)

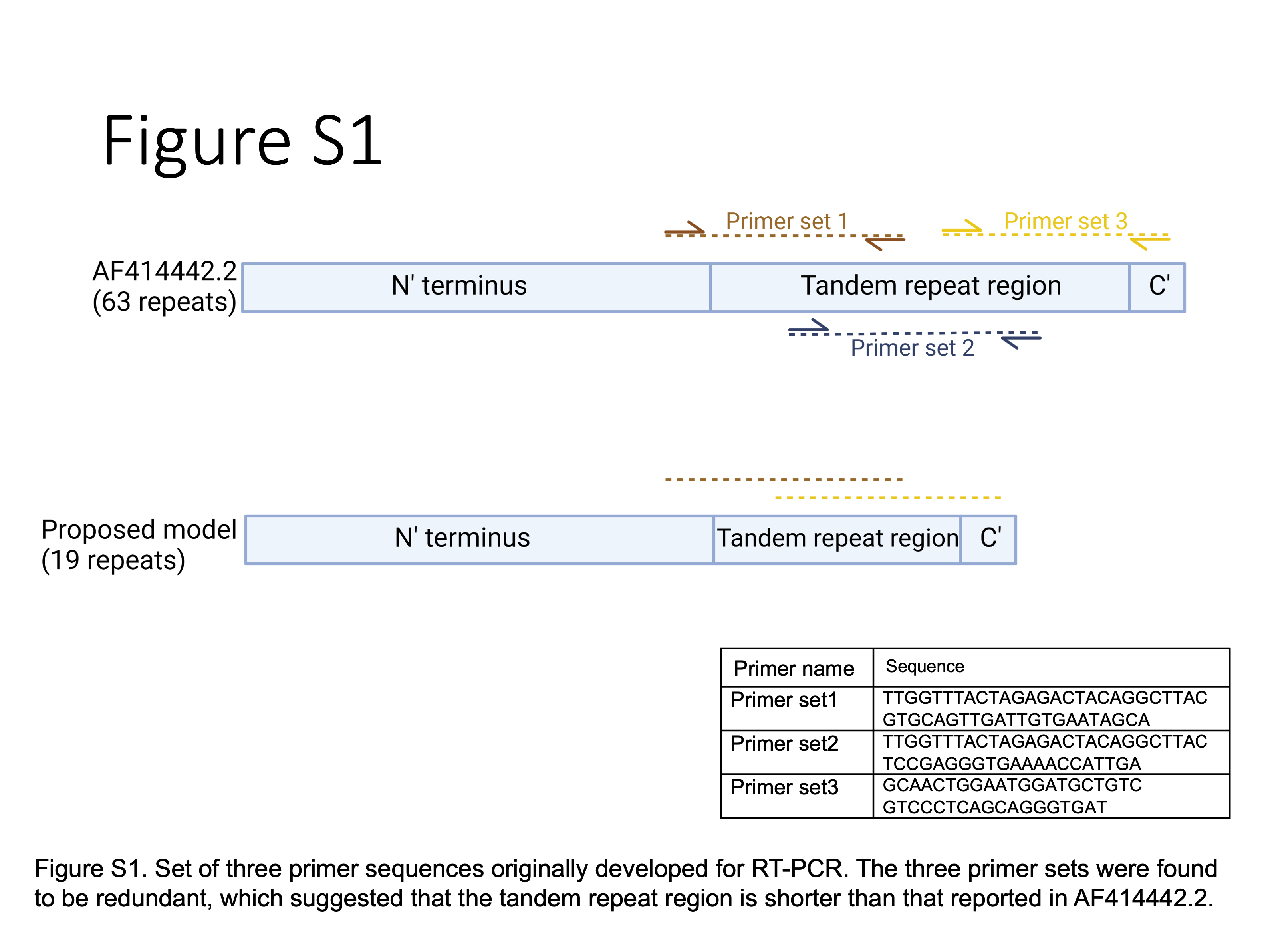

Supplement: Figure S1 — Set of three primer sequences originally developed for RT-PCR. The three primer sets were found to be redundant, which suggested that the tandem repeat region is shorter than that reported in AF414442.2. [file crc-23-0327-s01.png]

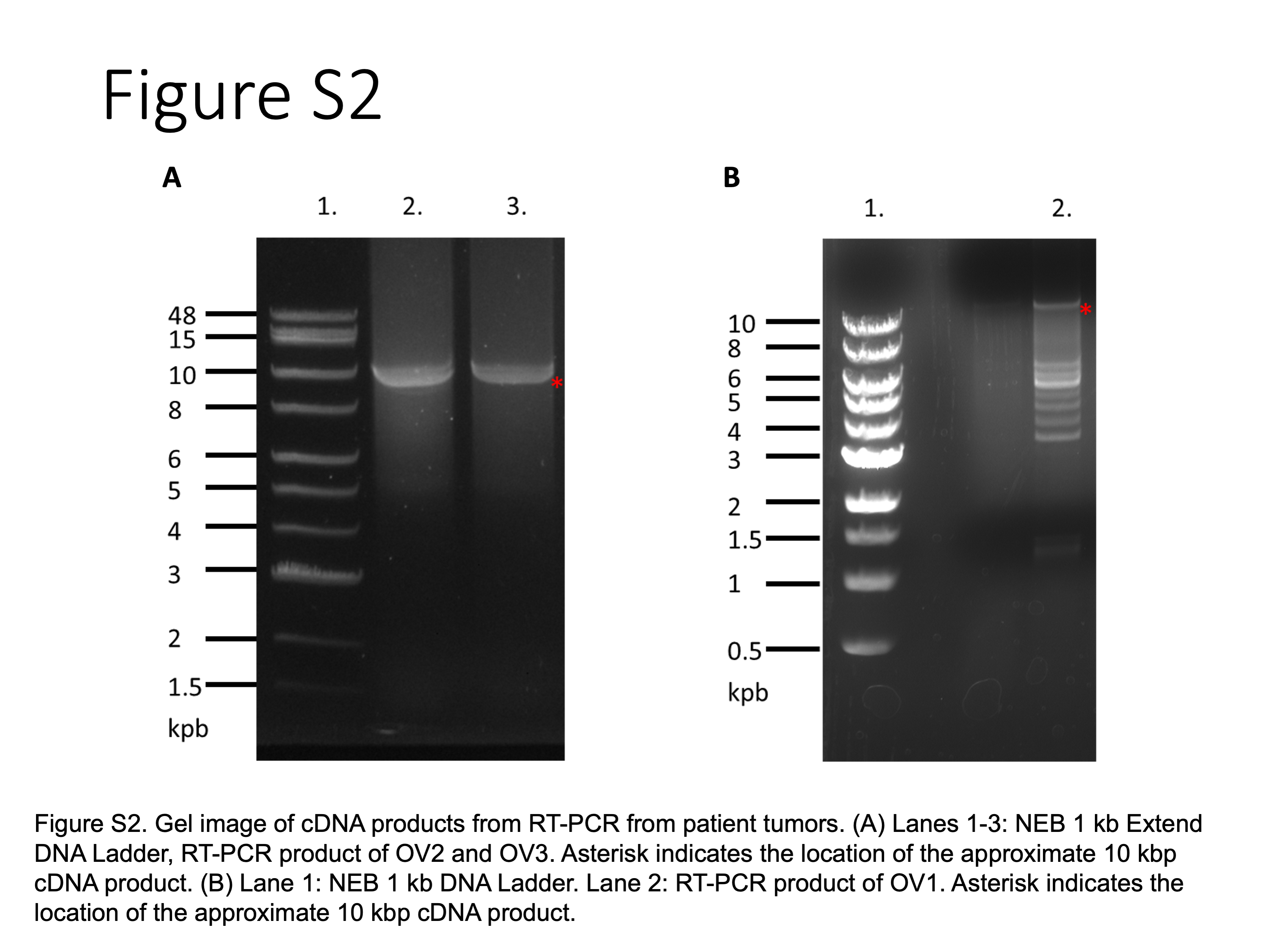

Supplement: Figure S2 — Gel image of cDNA products from RT-PCR from patient tumors. (A) Lanes 1-3: NEB 1 kb Extend DNA Ladder, RT-PCR product of OV2 and OV3. Asterisk indicates the location of the approximate 10 kbp cDNA product. (B) Lane 1: NEB 1 kb DNA Ladder. Lane 2: RT-PCR product of OV1. Asterisk indicates the location of the approximate 10 kbp cDNA product. [file crc-23-0327-s03.png]

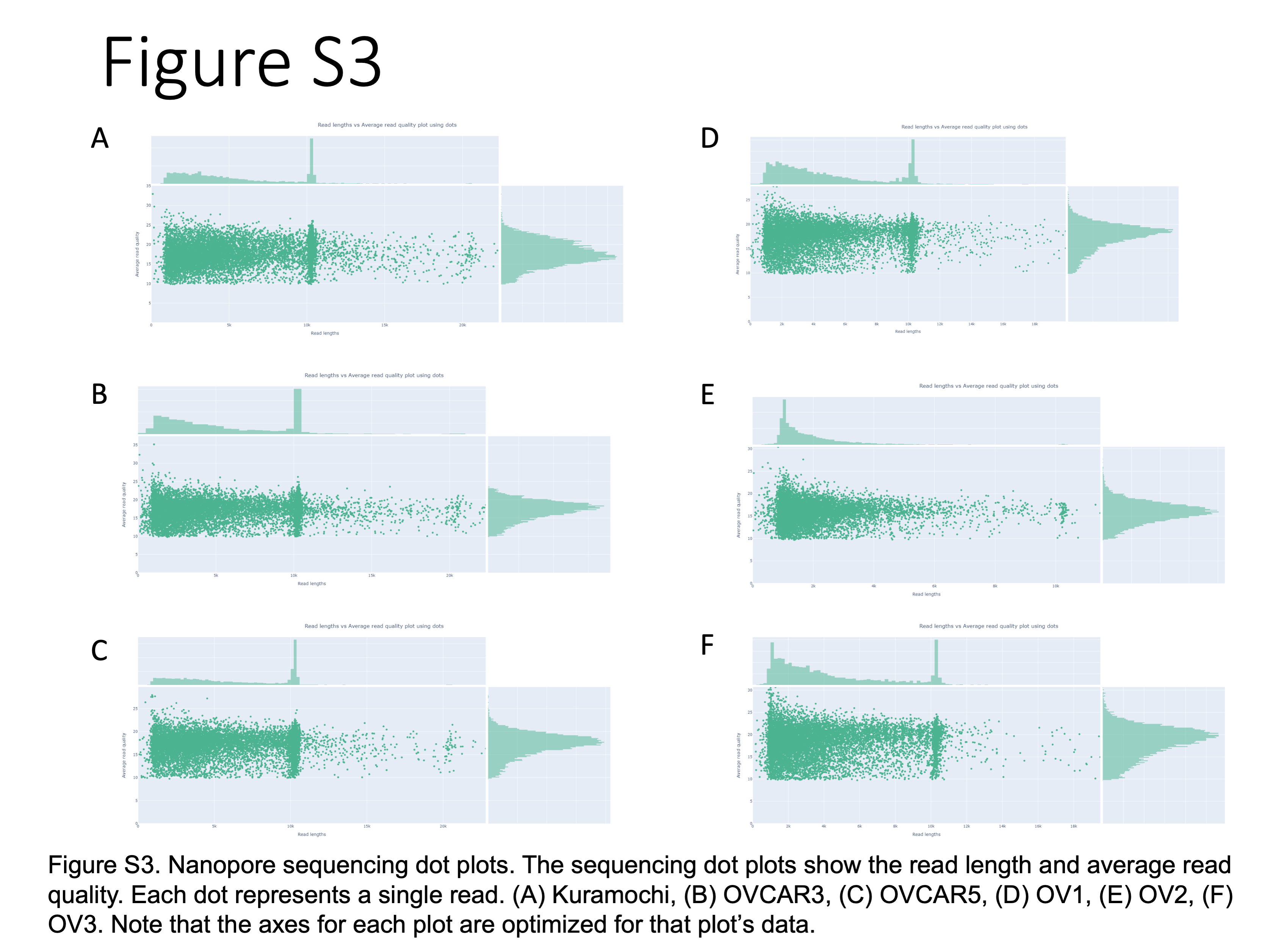

Supplement: Figure S3 — Nanopore sequencing dot plots. The sequencing dot plots show the read length and average read quality. Each dot represents a single read. (A) Kuramochi, (B) OVCAR3, (C) OVCAR5, (D) OV1, (E) OV2, (F) OV3. Note that the axes for each plot are optimized for that plot’s data. [file crc-23-0327-s05.png]

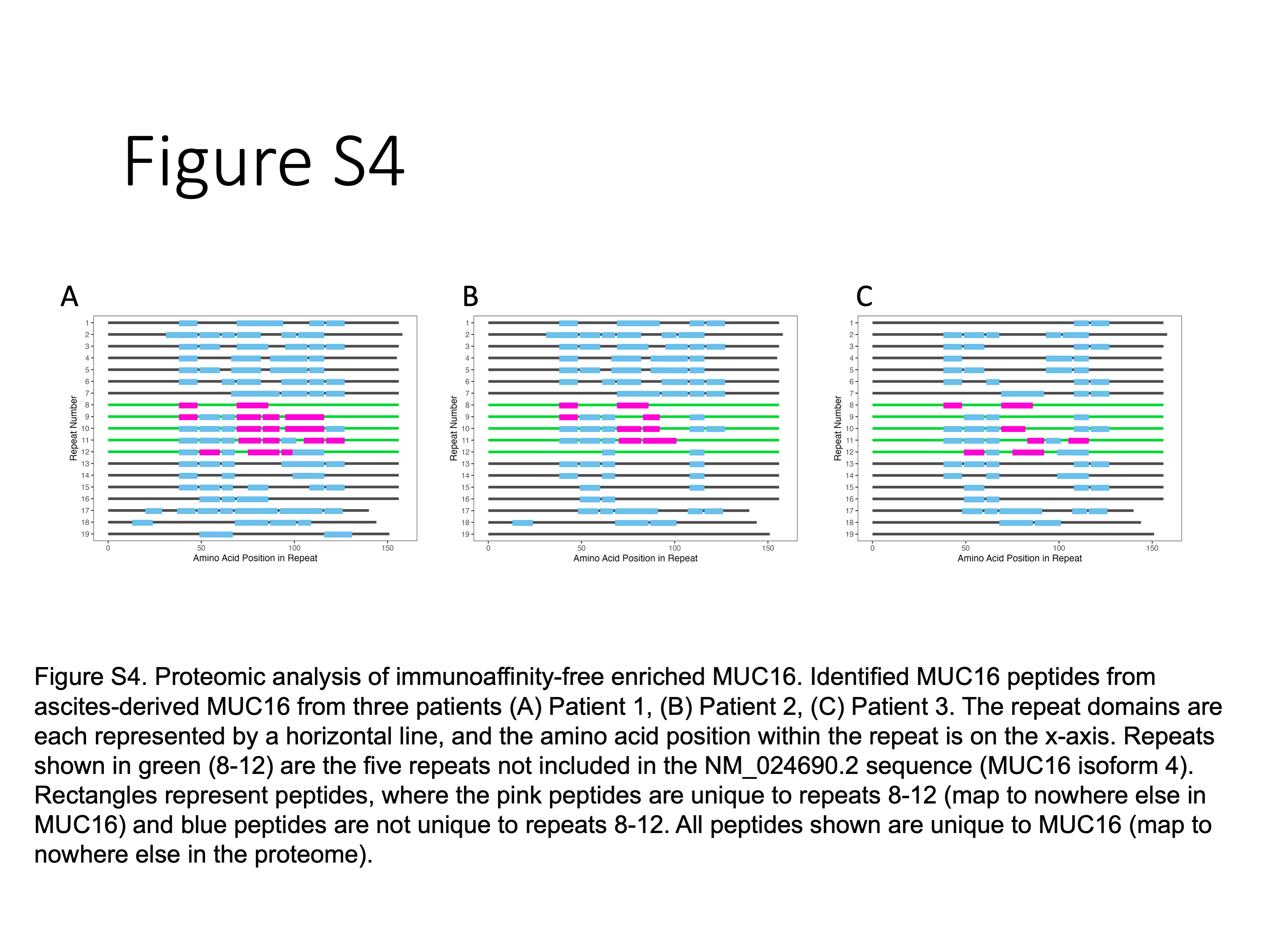

Supplement: Figure S4 — Proteomic analysis of immunoaffinity-free enriched MUC16. Identified MUC16 peptides from ascites-derived MUC16 from three patients (A) Patient 1, (B) Patient 2, (C) Patient 3. The repeat domains are each represented by a horizontal line, and the amino acid position within the repeat is on the x-axis. Repeats shown in green (8-12) are the five repeats not included in the NM_024690.2 sequence (MUC16 isoform 4). Rectangles represent peptides, where the pink peptides are unique to repeats 8-12 (map to nowhere else in MUC16) and blue peptides are not unique to repeats 8-12. All peptides shown are unique to MUC16 (map to nowhere else in the proteome). [file crc-23-0327-s07.png]

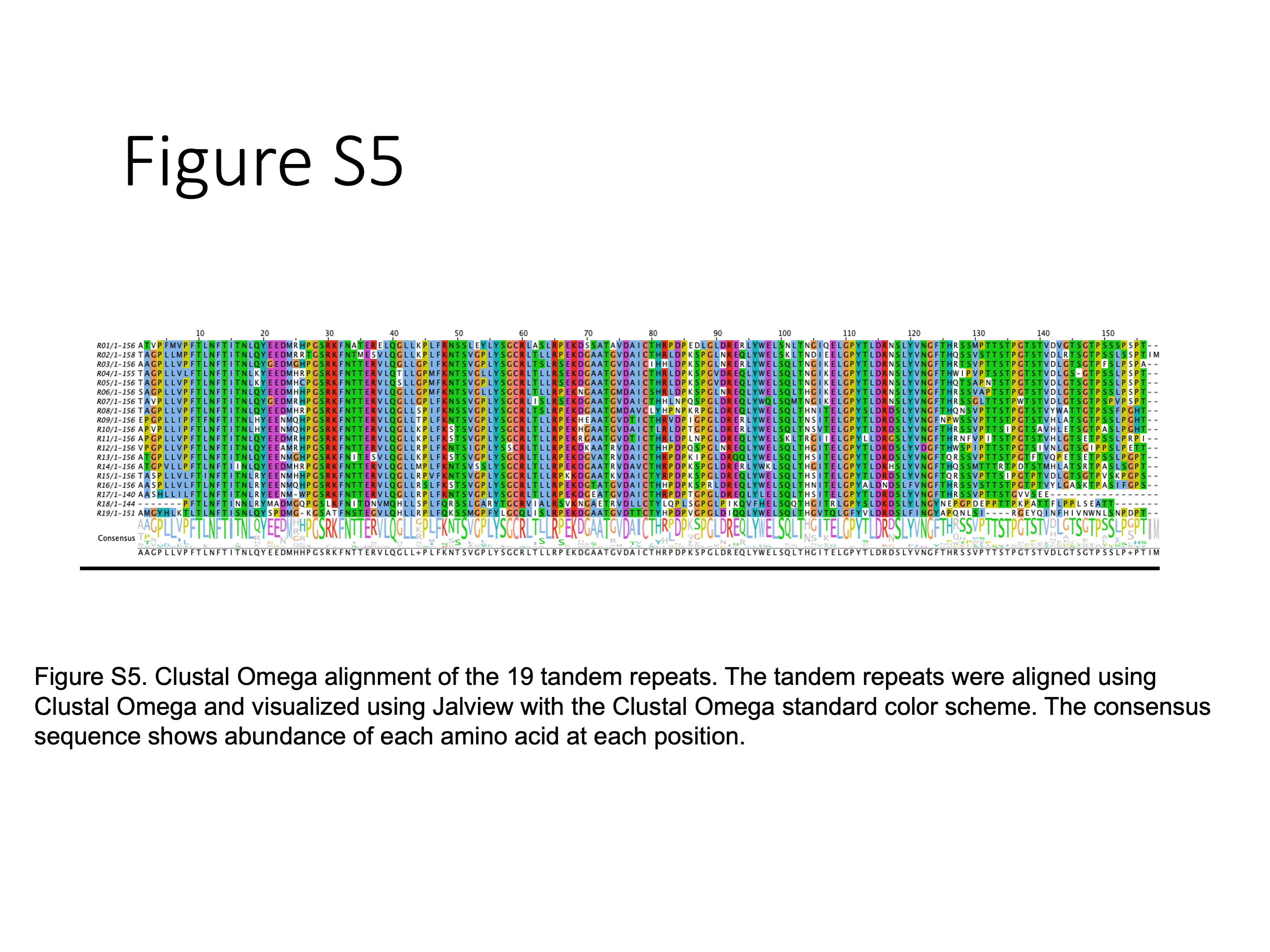

Supplement: Figure S5 — Clustal Omega alignment of the 19 tandem repeats. The tandem repeats were aligned using Clustal Omega and visualized using Jalview with the Clustal Omega standard color scheme. The consensus sequence shows abundance of each amino acid at each position. [file crc-23-0327-s09.png]

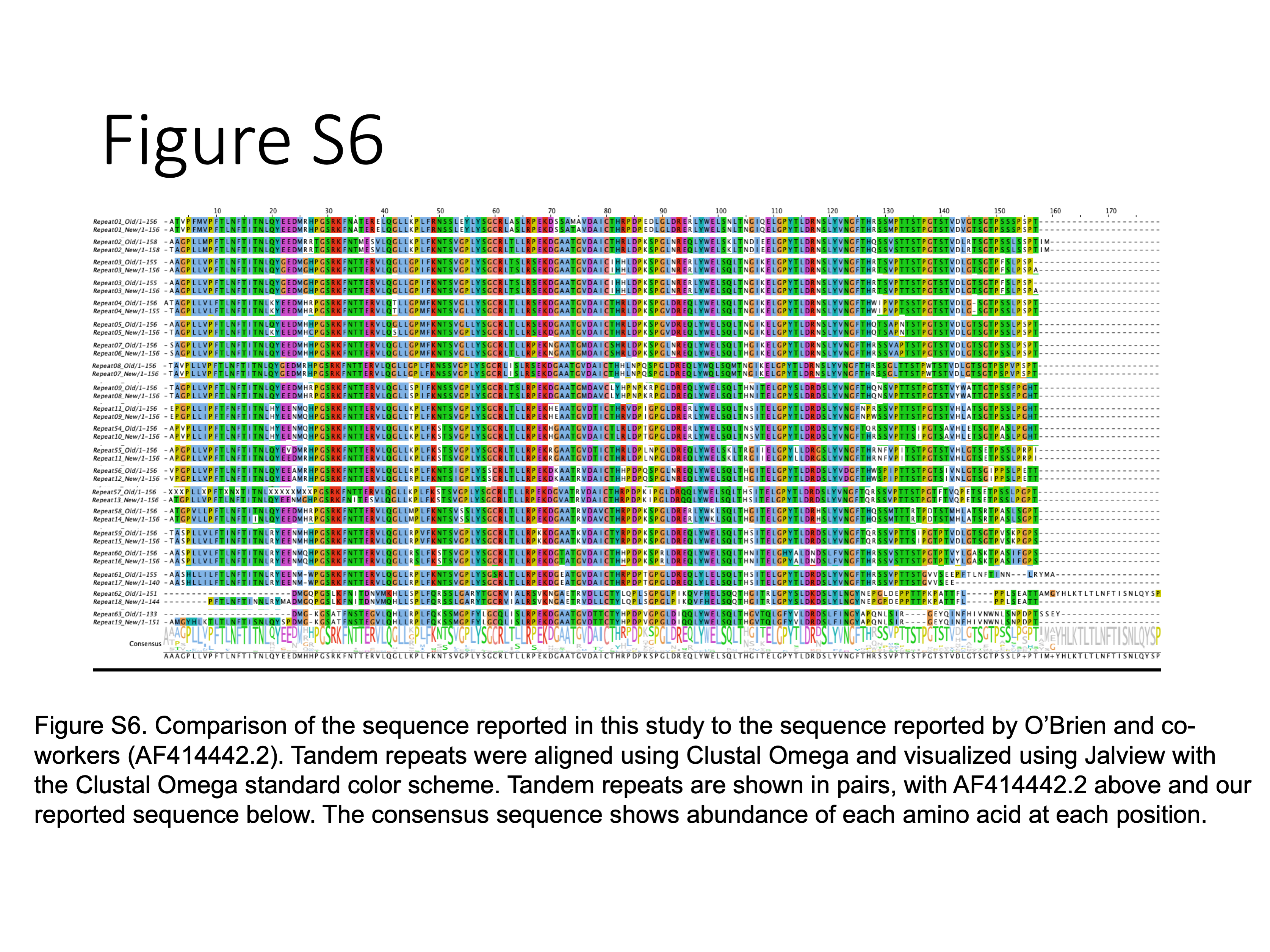

Supplement: Figure S6 — Comparison of the sequence reported in this study to the sequence reported by O’Brien and co-workers (AF414442.2). Tandem repeats were aligned using Clustal Omega and visualized using Jalview with the Clustal Omega standard color scheme. Tandem repeats are shown in pairs, with AF414442.2 above and our reported sequence below. The consensus sequence shows abundance of each amino acid at each position. [file crc-23-0327-s10.png]

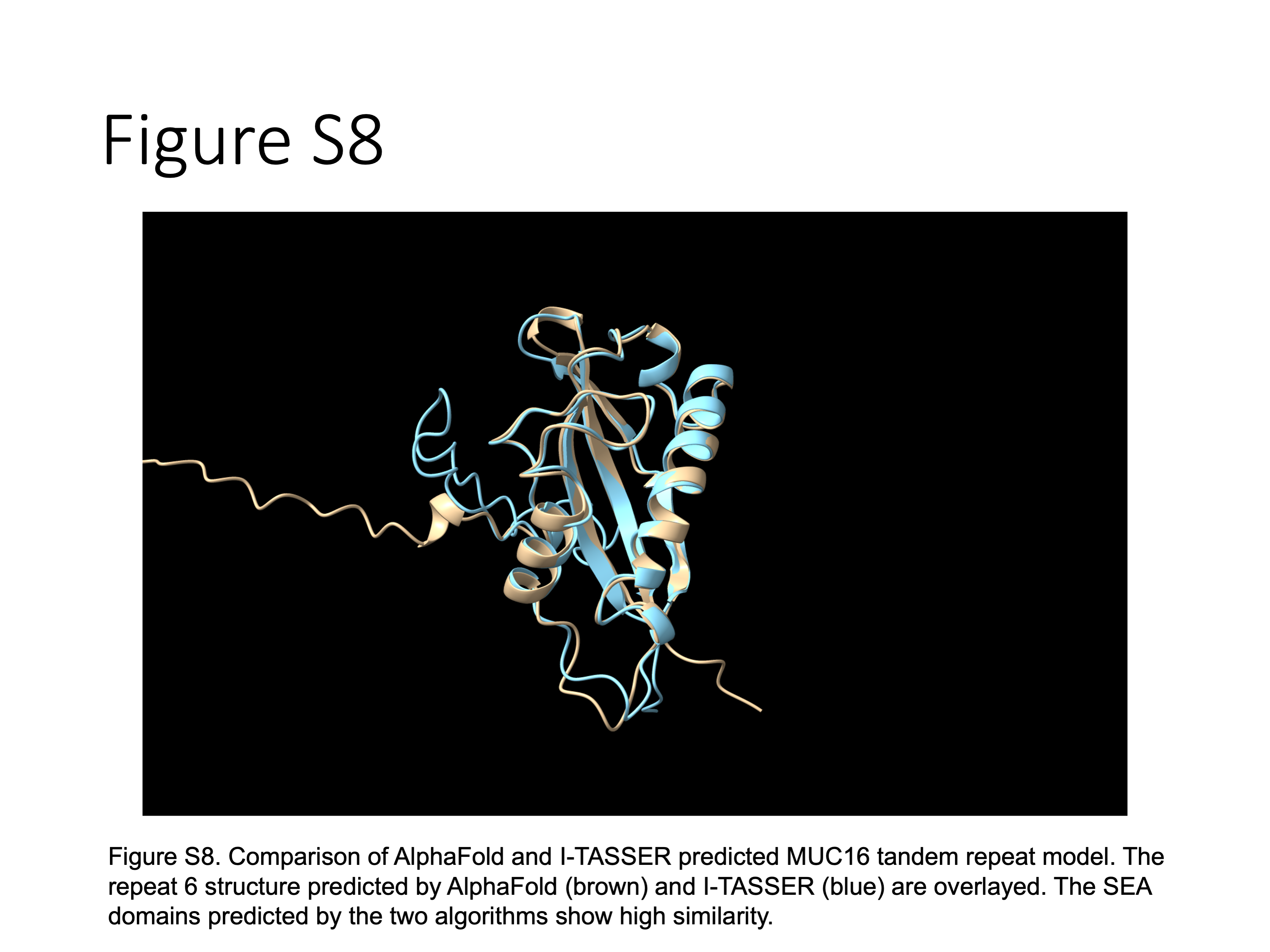

Supplement: Figure S8 — Comparison of AlphaFold and I-TASSER predicted MUC16 tandem repeat model. The repeat 6 structure predicted by AlphaFold (brown) and I-TASSER (blue) are overlayed. The SEA domains predicted by the two algorithms show high similarity. [file crc-23-0327-s12.png]

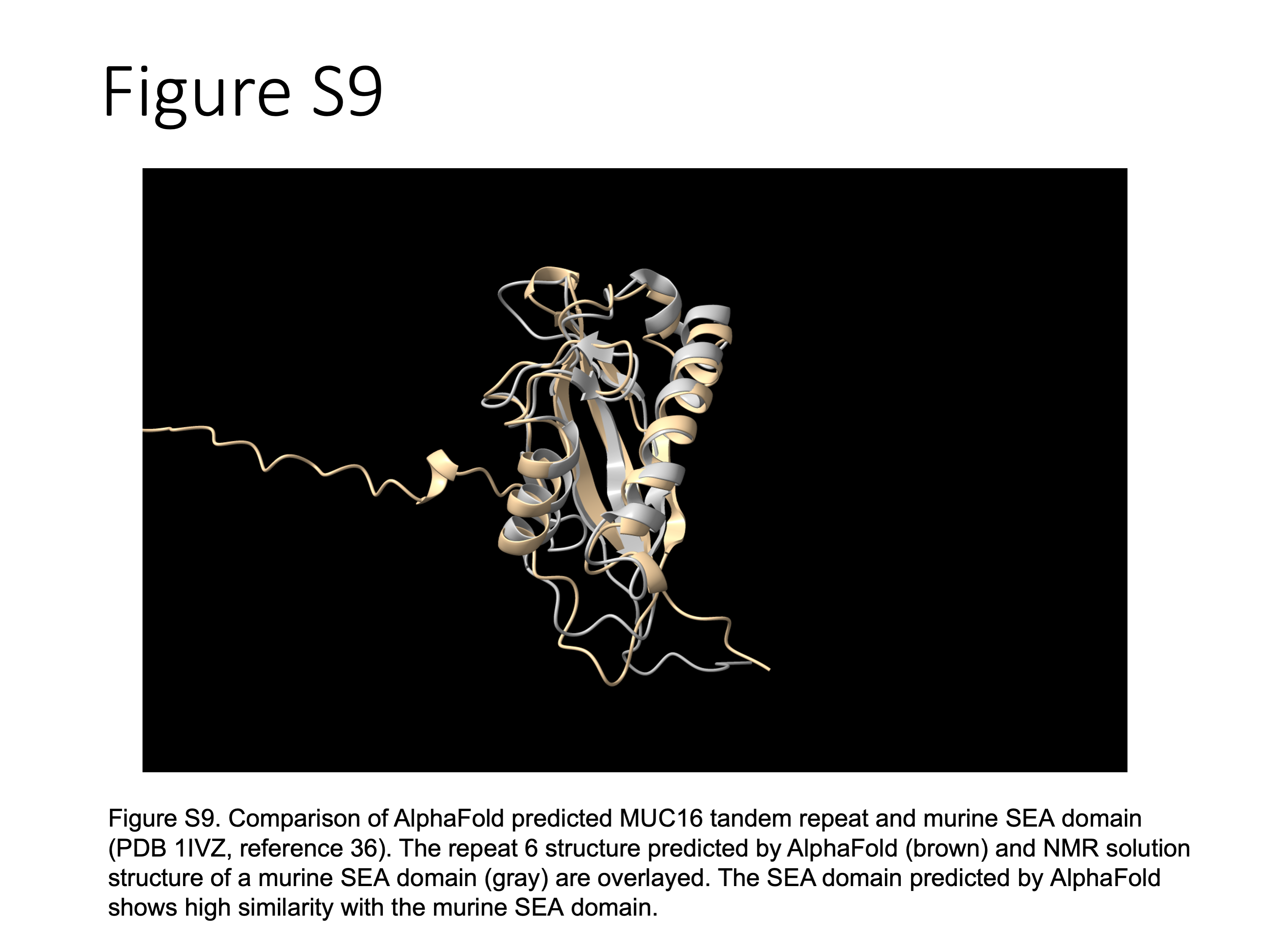

Supplement: Figure S9 — Comparison of AlphaFold predicted MUC16 tandem repeat and murine SEA domain (PDB 1IVZ, reference 36). The repeat 6 structure predicted by AlphaFold (brown) and NMR solution structure of a murine SEA domain (gray) are overlayed. The SEA domain predicted by AlphaFold shows high similarity with the murine SEA domain. [file crc-23-0327-s13.png]
