# Supplementary material for: A Revised Molecular Model of Ovarian Cancer Biomarker CA125 (MUC16) Enabled by Long-read Sequencing
Source: Cancer Res Commun. 2024 Jan 31;4(1):253–63. doi: 10.1158/2767-9764.CRC-23-0327 (PMC10829539; doi:10.1158/2767-9764.CRC-23-0327)
Supplement: Supplementary Document 1 — R script used for proteomics data analysis [file crc-23-0327-s02.pdf]

Proteomic_DataAnalysis_Muc16_nanopore.R

simon

2023-04-03

```r
#import packages
suppressPackageStartupMessages({
  library(stringr)
  library(readxl)
  library(seqinr)
  library(dplyr)
  library(ggplot2)
})
```

```
## Warning: package 'ggplot2' was built under R version 4.2.3
```

```r
####import sequences####
#repeats 8-12 for ovcar3 and 6sample consensus
novel_repeat_seq_ovcar3 <- "TAGPLLVPFTLNFTITNLQYEEDMHRPGSRKFNATERVLQGLLSPIFKNSSVGPLYSGCRLTSLRPEKDGAATGMDAVCLYHPNP
KRPGLDREQLYWELSQLTHNITELGPYSLDRDSLYVNGFTHQNSVPTTSTPGTSTVYWATTGTPSSFPGHTEPGPLLIPFTFNFTITNLHYEENMQHPGSRKFNTTERVLQGL
LTPLFKNTSVGPLYSGCRLTLLRPEKQEAATGVDTICTHRVDPIGPGLDRERLYWELSQLTNSITELGPYTLDRDSLYVNGFNPWSSVPTTSTPGTSTVHLATSGTPSSLPGH
TAPVPLLIPFTLNFTITNLHYEENMQHPGSRKFNTTERVLQGLLKPLFKSTSVGPLYSGCRLTLLRPEKHGAATGVDAICTLRLDPTGPGLDRERLYWELSQLTNSVTELGPY
TLDRDSLYVNGFTHRSSVPTTSIPGTSAVHLETSGTPASLPGHTAPGPLLVPFTLNFTITNLQYEEDMRHPGSRKFNTTERVLQGLLKPLFKSTSVGPLYSGCRLTLLRPEKR
GAATGVDTICTHRLDPLNPGLDREQLYWELSKLTRGIIELGPYLLDRGSLYVNGFTHRNFVPITSTPGTSTVHLGTSETPSSLPRPIVPGPLLVPFTLNFTITNLQYEEAMRH
PGSRKFNTTERVLQGLLRPLFKNTSIGPLYSSCRLTLLRPEKDKAATRVDAICTHHPDPQSPGLNREQLYWELSQLTHGITELGPYTLDRDSLYVDGFTHWSPIPTTSTPGTS
IVNLGTSGIPPSLPETT"
novel_repeat_seq_6samp_concensus <- "TAGPLLVPFTLNFTITNLQYEEDMHRPGSRKFNTTERVLQGLLSPIFKNSSVGPLYSGCRLTSLRPEKDGAATGMD
AVCLYHPNPKRPGLDREQLYWELSQLTHNITELGPYSLDRDSLYVNGFTHQNSVPTTSTPGTSTVYWATTGTPSSFPGHTEPGPLLIPFTFNFTITNLHYEENMQHPGSRKFN
TTERVLQGLLTPLFKNTSVGPLYSGCRLTLLRPEKHEAATGVDTICTHRVDPIGPGLDRERLYWELSQLTNSITELGPYTLDRDSLYVNGFNPWSSVPTTSTPGTSTVHLATS
GTPSSLPGHTAPVPLLIPFTLNFTITNLHYEENMQHPGSRKFNTTERVLQGLLKPLFKSTSVGPLYSGCRLTLLRPEKHGAATGVDAICTLRLDPTGPGLDRERLYWELSQLT
NSVTELGPYTLDRDSLYVNGFTHRSSVPTTSIPGTSAVHLETSGTPASLPGHTAPGPLLVPFTLNFTITNLQYEEDMRHPGSRKFNTTERVLQGLLKPLFKSTSVGPLYSGCR
LTLLRPEKRGAATGVDTICTHRLDPLNPGLDREQLYWELSKLTRGIIELGPYLLDRGSLYVNGFTHRNFVPITSTPGTSTVHLGTSETPSSLPRPIVPGPLLVPFTLNFTITN
LQYEEAMRHPGSRKFNTTERVLQGLLRPLFKNTSIGPLYSSCRLTLLRPEKDKAATRVDAICTHHPDPQSPGLNREQLYWELSQLTHGITELGPYTLDRDSLYVDGFTHWSPI
PTTSTPGTSIVNLGTSGIPPSLPETT"
#import entire MUC16 sequence, first as dataframe and then convert to string
muc16_file_ovcar3 <- read.csv("MUC16_OVCAR3_v1.txt", header = F)
muc16_seq_ovcar3 <- muc16_file_ovcar3[1,1]
muc16_file_6samp_concensus <- read.csv("MUC16_consensus_from 6_sequences.txt", header = F)
muc16_seq_6samp_concensus <- muc16_file_ovcar3[1,1]
#import proteome. This is the human proteome w/o MUC16 entry (every other protein)
proteome <- read.fasta(file = "Homo Sapiens UP000005640 no MUC16.fasta", seqtype = "AA", as.string = T)

####import peptide files####
####For each sample, create new column that is peptide sequence w/o mods.
####For each sample, create a dataframe with only MUC16 peptides.
#Fitzgerald sample searched against ovcar3 seq cont. database
Fitz_ov3 <- read.csv("IP_nanopore_SDW.OVCAR3_db_search_ovcar3_and_fitz_IPs/db.Fitzgerald_IP.peptides.csv")
Fitz_ov3$sequence <- str_remove_all(Fitz_ov3$Peptide, "[()\\.0-9\\-+]")
Fitz_ov3_MUC16 <- Fitz_ov3[str_detect(Fitz_ov3$Accession, "MUC16") ,]
Fitz_ov3_MUC16_peps <- Fitz_ov3_MUC16$sequence
#Ovcar3 sample searched against ovcar3 seq cont. database
Ovcar_ov3 <- read.csv("IP_nanopore_SDW.OVCAR3_db_search_ovcar3_and_fitz_IPs/db.OVCAR3_IP.peptides.csv")
Ovcar_ov3$sequence <- str_remove_all(Ovcar_ov3$Peptide, "[()\\.0-9\\-+]")
Ovcar_ov3_MUC16 <- Ovcar_ov3[str_detect(Ovcar_ov3$Accession, "MUC16") ,]
Ovcar_ov3_MUC16_peps <- Ovcar_ov3_MUC16$sequence
#Fitzgerald sample searched against 6sample consensus seq cont. database
Fitz_6s <- read.csv("IP_nanopore_SDW.6sampConcensus_db_Search_ovcar3_and_Fitz_IPs/db.Fitzgerald_IP.peptides.csv")
Fitz_6s$sequence <- str_remove_all(Fitz_6s$Peptide, "[()\\.0-9\\-+]")
Fitz_6s_MUC16 <- Fitz_6s[str_detect(Fitz_6s$Accession, "MUC16") ,]
Fitz_6s_MUC16_peps <- Fitz_6s_MUC16$sequence
#Ovcar3 sample searched against 6sample consensus seq cont. database
Ovcar_6s <- read.csv("IP_nanopore_SDW.6sampConcensus_db_Search_ovcar3_and_Fitz_IPs/db.OVCAR3_IP.peptides.csv")
Ovcar_6s$sequence <- str_remove_all(Ovcar_6s$Peptide, "[()\\.0-9\\-+]")
Ovcar_6s_MUC16 <- Ovcar_6s[str_detect(Ovcar_6s$Accession, "MUC16") ,]
Ovcar_6s_MUC16_peps <- Ovcar_6s_MUC16$sequence

####convert proteome to a string####
#create empty list, fill it with each entry in the proteome object
proteome_2 <- list()
for (i in 1:length(proteome)) {
  proteome_2[[i]] <- proteome[[i]][1]
}

#turn the list into a vector, and collapse it into one long string
proteome_vector <- unlist(proteome_2)
proteome_string <- paste(proteome_vector, sep = '', collapse = '')
proteome_string2 <- paste(proteome, sep = '', collapse = '')

####ANALYSIS FOR FITZGERALD####
#ovcar3 db analysis
#Create columns to store variable for whether sample is in R8-12,
#how many times it appears in MUC16, how many times it is in the proteome and how
#many times it is in the repeat
Fitz_ov3_MUC16$in_repeat <- F
Fitz_ov3_MUC16$count <- NA
Fitz_ov3_MUC16$count_proteome <- NA
Fitz_ov3_MUC16$count_in_repeat <- NA
#Fill these columns with string matching/counting for each peptide in the MUC16 dataframe
for (i in 1:nrow(Fitz_ov3_MUC16)) {
  Fitz_ov3_MUC16$in_repeat[i] <- grepl(Fitz_ov3_MUC16$sequence[i], novel_repeat_seq_ovcar3)
  Fitz_ov3_MUC16$count[i] <- str_count(muc16_seq_ovcar3, Fitz_ov3_MUC16$sequence[i])
  Fitz_ov3_MUC16$count_proteome[i] <- str_count(proteome_string2, Fitz_ov3_MUC16$sequence[i])
  Fitz_ov3_MUC16$count_in_repeat[i] <- str_count(novel_repeat_seq_ovcar3, Fitz_ov3_MUC16$sequence[i])
}

#create a column evaluating if the peptide is unique to R8-12
Fitz_ov3_MUC16$unique_to_repeat <- F

#Fill this column based on the boolean values from the columns above
for (i in 1:nrow(Fitz_ov3_MUC16)) {
  if (Fitz_ov3_MUC16$in_repeat[i] == T && Fitz_ov3_MUC16$count[i] == Fitz_ov3_MUC16$count_in_repeat[i] && Fitz_ov
3_MUC16$count_proteome[i] == 0) {
    Fitz_ov3_MUC16$unique_to_repeat[i] <- T
  }
}

#Pull out the peptides that map to MUC16, are unique to MUC16, and are unique to R8-12
peptides_of_interest_Fitz_ov3 <- Fitz_ov3_MUC16[Fitz_ov3_MUC16$unique_to_repeat == T,]
peptide_list_Fitz_ov3 <- peptides_of_interest_Fitz_ov3$sequence
peptide_list_final_Fitz_ov3 <- unique(peptide_list_Fitz_ov3)

#Same analysis as above, but with the 6sample consensus sequence
#6 sample concensus analysis
Fitz_6s_MUC16$in_repeat <- F
Fitz_6s_MUC16$count <- NA
Fitz_6s_MUC16$count_proteome <- NA
Fitz_6s_MUC16$count_in_repeat <- NA
for (i in 1:nrow(Fitz_6s_MUC16)) {
  Fitz_6s_MUC16$in_repeat[i] <- grepl(Fitz_6s_MUC16$sequence[i], novel_repeat_seq_6samp_concensus)
  Fitz_6s_MUC16$count[i] <- str_count(muc16_seq_6samp_concensus, Fitz_6s_MUC16$sequence[i])
  Fitz_6s_MUC16$count_proteome[i] <- str_count(proteome_string2, Fitz_6s_MUC16$sequence[i])
  Fitz_6s_MUC16$count_in_repeat[i] <- str_count(novel_repeat_seq_6samp_concensus, Fitz_6s_MUC16$sequence[i])
}
Fitz_6s_MUC16$unique_to_repeat <- F
for (i in 1:nrow(Fitz_6s_MUC16)) {
  if (Fitz_6s_MUC16$in_repeat[i] == T && Fitz_6s_MUC16$count[i] == Fitz_6s_MUC16$count_in_repeat[i] && Fitz_6s_MU
C16$count_proteome[i] == 0) {
    Fitz_6s_MUC16$unique_to_repeat[i] <- T
  }
}
peptides_of_interest_Fitz_6s <- Fitz_6s_MUC16[Fitz_6s_MUC16$unique_to_repeat == T,]
peptide_list_Fitz_6s <- peptides_of_interest_Fitz_6s$sequence
peptide_list_final_Fitz_6s <- unique(peptide_list_Fitz_6s)

#calculate percent coverage of repeats 8-12
#pull out peptides that mapped to R8-12
R8_12 <- Fitz_6s_MUC16[Fitz_6s_MUC16$in_repeat == T,]
AA_vec <- str_split(novel_repeat_seq_6samp_concensus, pattern = "")[[1]]
AA_df <- data.frame(AA = AA_vec,
                    covered = F)
for (i in 1:nrow(R8_12)) {
  matches <- data.frame(str_locate_all(novel_repeat_seq_6samp_concensus, R8_12$sequence[i]))
  for (j in 1:nrow(matches)) {
    start <- matches$start[j]
    end <- matches$end[j]
    sequence <- start:end
    for (k in sequence) {
      AA_df$covered[k] <- T
    }
  }
}

percent_coverage_8_12 <- (sum(AA_df$covered) / nrow(AA_df)) * 100
#print results
cat(paste0("Results for Fitzgerald Sample mapped to 6sample consensus:", "\n",
           "MUC16 peptides: ", nrow(Fitz_6s_MUC16), "\n",
           "MUC16 sequences: ", length(unique(Fitz_6s_MUC16$sequence)), "\n",
           "R8 - R12 peptides: ", nrow(R8_12), "\n",
           "R8 - R12 sequences: ", length(unique(R8_12$sequence)), "\n",
           "R8 - R12 percent coverage: ", round(percent_coverage_8_12, 2),"%", "\n",
           "sequences unique to R8-12: ", length(peptide_list_final_Fitz_6s), "\n"))
```

```
## Results for Fitzgerald Sample mapped to 6sample consensus:
## MUC16 peptides: 47
## MUC16 sequences: 38
## R8 - R12 peptides: 21
## R8 - R12 sequences: 17
## R8 - R12 percent coverage: 28.46%
## sequences unique to R8-12: 10
```

```r
peptide_list_final_Fitz_6s
```

```
##  [1] "DGAATGMDAVCLYHPNPK" "GIIELGPYLLDR"       "LDPLNPGLDR"
##  [4] "LDPTGPGLDR"         "LDPTGPGLDRER"       "NTSIGPLYSSCR"
##  [7] "VDPIGPGLDR"         "VDPIGPGLDRER"       "VLQGLLSPIFK"
## [10] "VLQGLLTPLFK"
```

```r
#save peptides to .txt file
write.table(peptide_list_final_Fitz_ov3, "unique_repeat_peptides_Fitz_IP_ov3_db.txt",
            row.names = F, col.names = F, quote = F)
write.table(peptide_list_final_Fitz_6s, "unique_repeat_peptides_Fitz_IP_6s_db.txt",
            row.names = F, col.names = F, quote = F)

####ANALYSIS FOR OVCAR3####
#same analysis as above, but for the OVCAR3 IP sample
#ovcar3 db
Ovcar_ov3_MUC16$in_repeat <- F
Ovcar_ov3_MUC16$count <- NA
Ovcar_ov3_MUC16$count_proteome <- NA
Ovcar_ov3_MUC16$count_in_repeat <- NA
for (i in 1:nrow(Ovcar_ov3_MUC16)) {
  Ovcar_ov3_MUC16$in_repeat[i] <- grepl(Ovcar_ov3_MUC16$sequence[i], novel_repeat_seq_ovcar3)
  Ovcar_ov3_MUC16$count[i] <- str_count(muc16_seq_ovcar3, Ovcar_ov3_MUC16$sequence[i])
  Ovcar_ov3_MUC16$count_proteome[i] <- str_count(proteome_string2, Ovcar_ov3_MUC16$sequence[i])
  Ovcar_ov3_MUC16$count_in_repeat[i] <- str_count(novel_repeat_seq_ovcar3, Ovcar_ov3_MUC16$sequence[i])
}
Ovcar_ov3_MUC16$unique_to_repeat <- F
for (i in 1:nrow(Ovcar_ov3_MUC16)) {
  if (Ovcar_ov3_MUC16$in_repeat[i] == T && Ovcar_ov3_MUC16$count[i] == Ovcar_ov3_MUC16$count_in_repeat[i] && Ovca
r_ov3_MUC16$count_proteome[i] == 0) {
    Ovcar_ov3_MUC16$unique_to_repeat[i] <- T
  }
}
peptides_of_interest_Ovcar3_ov3 <- Ovcar_ov3_MUC16[Ovcar_ov3_MUC16$unique_to_repeat == T,]
peptide_list_Ovcar3_ov3 <- peptides_of_interest_Ovcar3_ov3$sequence
peptide_list_final_Ovcar3_ov3 <- unique(peptide_list_Ovcar3_ov3)

#calculate percent coverage of repeats 8-12
#pull out peptides that mapped to R8-12
R8_12 <- Ovcar_ov3_MUC16[Ovcar_ov3_MUC16$in_repeat == T,]
AA_vec <- str_split(novel_repeat_seq_ovcar3, pattern = "")[[1]]
AA_df <- data.frame(AA = AA_vec,
                    covered = F)
for (i in 1:nrow(R8_12)) {
  matches <- data.frame(str_locate_all(novel_repeat_seq_ovcar3, R8_12$sequence[i]))
  for (j in 1:nrow(matches)) {
    start <- matches$start[j]
    end <- matches$end[j]
    sequence <- start:end
    for (k in sequence) {
      AA_df$covered[k] <- T
    }
  }
}

percent_coverage_8_12 <- (sum(AA_df$covered) / nrow(AA_df)) * 100
#print results
cat(paste0("Results for OVCAR3 Sample mapped to OVCAR3 consensus:", "\n",
           "MUC16 peptides: ", nrow(Ovcar_ov3_MUC16), "\n",
           "MUC16 sequences: ", length(unique(Ovcar_ov3_MUC16$sequence)), "\n",
           "R8 - R12 peptides: ", nrow(R8_12), "\n",
           "R8 - R12 sequences: ", length(unique(R8_12$sequence)), "\n",
           "R8 - R12 percent coverage: ", round(percent_coverage_8_12, 2),"%", "\n",
           "sequences unique to R8-12: ", length(peptide_list_final_Ovcar3_ov3), "\n"))
```

```
## Results for OVCAR3 Sample mapped to OVCAR3 consensus:
## MUC16 peptides: 24
## MUC16 sequences: 21
## R8 - R12 peptides: 10
## R8 - R12 sequences: 10
## R8 - R12 percent coverage: 18.33%
## sequences unique to R8-12: 5
```

```r
peptide_list_final_Ovcar3_ov3
```

```
## [1] "GIIELGPYLLDR" "LDPLNPGLDR"   "LDPTGPGLDR"   "VDPIGPGLDR"   "VLQGLLTPLFK"
```

```r
#6 sample concensus analysis
Ovcar_6s_MUC16$in_repeat <- F
Ovcar_6s_MUC16$count <- NA
Ovcar_6s_MUC16$count_proteome <- NA
Ovcar_6s_MUC16$count_in_repeat <- NA
for (i in 1:nrow(Ovcar_6s_MUC16)) {
  Ovcar_6s_MUC16$in_repeat[i] <- grepl(Ovcar_6s_MUC16$sequence[i], novel_repeat_seq_6samp_concensus)
  Ovcar_6s_MUC16$count[i] <- str_count(muc16_seq_6samp_concensus, Ovcar_6s_MUC16$sequence[i])
  Ovcar_6s_MUC16$count_proteome[i] <- str_count(proteome_string2, Ovcar_6s_MUC16$sequence[i])
  Ovcar_6s_MUC16$count_in_repeat[i] <- str_count(novel_repeat_seq_6samp_concensus, Ovcar_6s_MUC16$sequence[i])
}
Ovcar_6s_MUC16$unique_to_repeat <- F
for (i in 1:nrow(Ovcar_6s_MUC16)) {
  if (Ovcar_6s_MUC16$in_repeat[i] == T && Ovcar_6s_MUC16$count[i] == Ovcar_6s_MUC16$count_in_repeat[i] && Ovcar_6
s_MUC16$count_proteome[i] == 0) {
    Ovcar_6s_MUC16$unique_to_repeat[i] <- T
  }
}
peptides_of_interest_Ovcar3_6s <- Ovcar_6s_MUC16[Ovcar_6s_MUC16$unique_to_repeat == T,]
peptide_list_Ovcar3_6s <- peptides_of_interest_Ovcar3_6s$sequence
peptide_list_final_Ovcar3_6s <- unique(peptide_list_Ovcar3_6s)
write.table(peptide_list_final_Ovcar3_ov3, "unique_repeat_peptides_Ovcar3_IP_ov3_db.txt",
            row.names = F, col.names = F, quote = F)
write.table(peptide_list_final_Ovcar3_6s, "unique_repeat_peptides_Ovcar3_IP_6s_db.txt",
            row.names = F, col.names = F, quote = F)

####plot creation####
####Creates the plots to map peptides onto MUC16 repeats. Color R8-12 a different
####color, and color peptides unique to R8-12 a different color
#fitz mapped to 6s concensus
repeats_6s <- read.csv("6samp_concensus_repeats_byline.txt", header = F)
repeats_6s <- repeats_6s$V1
Fitz_6s_MUC16 <- Fitz_6s_MUC16[Fitz_6s_MUC16$count_proteome[i] == 0,]

sequence <- list()
repeat_n <- list()
start <- list()
end <- list()
unique_8to12 <- list()

for (i in 1:nrow(Fitz_6s_MUC16)) {
  for (j in 1:length(repeats_6s)) {
    matches <- data.frame(str_locate_all(repeats_6s[j], Fitz_6s_MUC16$sequence[i]))
    if (nrow(matches > 0)) {
      sequence[[length(sequence) + 1]] <- Fitz_6s_MUC16$sequence[i]
      repeat_n[[length(repeat_n) + 1]] <- j
      start[[length(start) + 1]] <- matches$start[1]
      end[[length(end) + 1]] <- matches$end[1]
      unique_8to12[[length(unique_8to12) + 1]] <- Fitz_6s_MUC16$unique_to_repeat[i]
    }
  }
}

matches_df <- data.frame(sequence = unlist(sequence),
                         repeat_n = unlist(repeat_n),
                         start = unlist(start),
                         end = unlist(end),
                         unique = unlist(unique_8to12))
matches_df$inv_repeat <- (19 - matches_df$repeat_n) + 1

aa_count <- rep(NA, 19)
for (i in 1:length(repeats_6s)) {
  aa_count[i] <- nchar(repeats_6s[i])
}

repeat_category <- c(rep("old", 7), rep("new", 5), rep("old", 7))

repeat_df <- data.frame(repeat_number = 1:19,
                        aa_count = aa_count,
                        repeat_category = repeat_category)
repeat_df_0 <- repeat_df
repeat_df_0$aa_count <- 0

repeat_df <- bind_rows(repeat_df, repeat_df_0)
repeat_df$repeat_number <- factor(repeat_df$repeat_number, levels = 19:1)

fitz_6s_plot <- ggplot() +
  geom_line(data = repeat_df,
            aes(x = aa_count, y = repeat_number, color = repeat_category, group = repeat_number),
            lwd = 2) +
  theme_bw(base_size = 15) +
  theme(panel.grid = element_blank(), legend.position = "none",
        plot.title = element_text(size = 10)) +
  labs(y = "Repeat Number", x = "Amino Acid Position in Repeat",
       title = "Fitzgerald sample peptides mapped to consensus sequence") +
  scale_color_manual(values = c("#04d635", "grey30")) +
  geom_rect(data = matches_df,
            aes(xmin = start, xmax = end, ymin = inv_repeat - 0.3, ymax = inv_repeat + 0.2,
                fill = unique)) +
  scale_fill_manual(values = c("#6abfeb", "#fc03d3"))
fitz_6s_plot
```

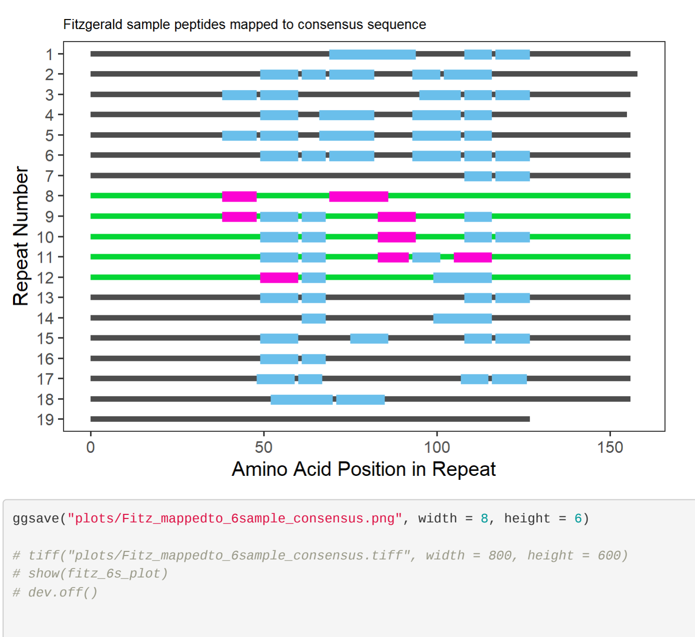


```r
ggsave("plots/Fitz_mappedto_6sample_consensus.png", width = 8, height = 6)

# tiff("plots/Fitz_mappedto_6sample_consensus.tiff", width = 800, height = 600)
# show(fitz_6s_plot)
# dev.off()


#ovcar3 mapped to 6s concensus
repeats_ov3 <- read.csv("Ovcar3_concensus_repeats_byline.txt", header = F)
repeats_ov3 <- repeats_ov3$V1
Ovcar_ov3_MUC16 <- Ovcar_ov3_MUC16[Ovcar_ov3_MUC16$count_proteome[i] == 0,]

sequence <- list()
repeat_n <- list()
start <- list()
end <- list()
unique_8to12 <- list()

for (i in 1:nrow(Ovcar_ov3_MUC16)) {
  for (j in 1:length(repeats_ov3)) {
    matches <- data.frame(str_locate_all(repeats_ov3[j], Ovcar_ov3_MUC16$sequence[i]))
    if (nrow(matches > 0)) {
      sequence[[length(sequence) + 1]] <- Ovcar_ov3_MUC16$sequence[i]
      repeat_n[[length(repeat_n) + 1]] <- j
      start[[length(start) + 1]] <- matches$start[1]
      end[[length(end) + 1]] <- matches$end[1]
      unique_8to12[[length(unique_8to12) + 1]] <- Ovcar_ov3_MUC16$unique_to_repeat[i]
    }
  }
}

matches_df <- data.frame(sequence = unlist(sequence),
                         repeat_n = unlist(repeat_n),
                         start = unlist(start),
                         end = unlist(end),
                         unique = unlist(unique_8to12))
matches_df$inv_repeat <- (19 - matches_df$repeat_n) + 1

aa_count <- rep(NA, 19)
for (i in 1:length(repeats_ov3)) {
  aa_count[i] <- nchar(repeats_ov3[i])
}

repeat_category <- c(rep("old", 7), rep("new", 5), rep("old", 7))

repeat_df <- data.frame(repeat_number = 1:19,
                         aa_count = aa_count,
                         repeat_category = repeat_category)
repeat_df_0 <- repeat_df
repeat_df_0$aa_count <- 0

repeat_df <- bind_rows(repeat_df, repeat_df_0)
repeat_df$repeat_number <- factor(repeat_df$repeat_number, levels = 19:1)

Ovcar_ov3_plot <- ggplot() +
  geom_line(data = repeat_df,
            aes(x = aa_count, y = repeat_number, color = repeat_category, group = repeat_number),
            lwd = 2) +
  theme_bw(base_size = 15) +
  theme(panel.grid = element_blank(), legend.position = "none",
        plot.title = element_text(size = 10)) +
  labs(y = "Repeat Number", x = "Amino Acid Position in Repeat",
       title = "Ovcar3 sample peptides mapped to ov3 consensus sequence") +
  scale_color_manual(values = c("#04d635", "grey30")) +
  geom_rect(data = matches_df,
            aes(xmin = start, xmax = end, ymin = inv_repeat - 0.3, ymax = inv_repeat + 0.2,
                fill = unique)) +
  scale_fill_manual(values = c("#6abfeb", "#fc03d3"))
Ovcar_ov3_plot
```

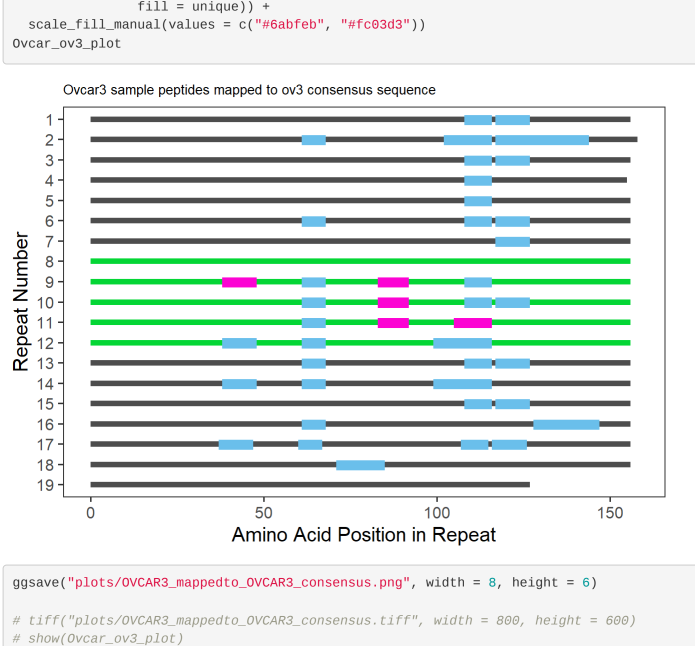


```r
ggsave("plots/OVCAR3_mappedto_OVCAR3_consensus.png", width = 8, height = 6)

# tiff("plots/OVCAR3_mappedto_OVCAR3_consensus.tiff", width = 800, height = 600)
# show(Ovcar_ov3_plot)
# dev.off()


sessionInfo()
```

```
## R version 4.2.2 (2022-10-31 ucrt)
## Platform: x86_64-w64-mingw32/x64 (64-bit)
## Running under: Windows 10 x64 (build 22621)
##
## Matrix products: default
##
## locale:
## [1] LC_COLLATE=English_United States.utf8
## [2] LC_CTYPE=English_United States.utf8
## [3] LC_MONETARY=English_United States.utf8
## [4] LC_NUMERIC=C
## [5] LC_TIME=English_United States.utf8
##
## attached base packages:
## [1] stats     graphics  grDevices utils     datasets  methods   base
##
## other attached packages:
## [1] ggplot2_3.4.1 dplyr_1.1.0   seqinr_4.2-23 readxl_1.4.2  stringr_1.5.0
##
## loaded via a namespace (and not attached):
##  [1] cellranger_1.1.0  bslib_0.4.2       compiler_4.2.2    pillar_1.8.1
##  [5] jquerylib_0.1.4   tools_4.2.2       digest_0.6.31     jsonlite_1.8.4
##  [9] evaluate_0.20     lifecycle_1.0.3   tibble_3.1.8      gtable_0.3.1
## [13] pkgconfig_2.0.3   rlang_1.0.6       cli_3.6.0         rstudioapi_0.14
## [17] yaml_2.3.7        xfun_0.36         fastmap_1.1.0     withr_2.5.0
## [21] knitr_1.42        systemfonts_1.0.4 generics_0.1.3    sass_0.4.5
## [25] vctrs_0.5.2       ade4_1.7-20       grid_4.2.2        tidyselect_1.2.0
## [29] glue_1.6.2        R6_2.5.1          textshaping_0.3.6 fansi_1.0.4
## [33] rmarkdown_2.20    farver_2.1.1      magrittr_2.0.3    scales_1.2.1
## [37] htmltools_0.5.4   MASS_7.3-58.1     colorspace_2.1-0  ragg_1.2.5
## [41] labeling_0.4.2    utf8_1.2.2        stringi_1.7.12    munsell_0.5.0
## [45] cachem_1.0.6
```
