# Supplementary material for: A Revised Molecular Model of Ovarian Cancer Biomarker CA125 (MUC16) Enabled by Long-read Sequencing
Source: Cancer Res Commun. 2024 Jan 31;4(1):253–63. doi: 10.1158/2767-9764.CRC-23-0327 (PMC10829539; doi:10.1158/2767-9764.CRC-23-0327)
Supplement: Supplementary Document 2 — DNA alignment [file crc-23-0327-s04.pdf]

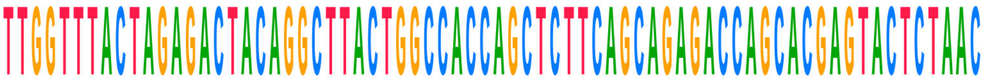

| | | |
|---|---|---|
| **Consensus** | **TTGGTTTACTAGAGACTACAGGCTTACTGGCCACCAGCTCTTCAGCAGAGACCAGCACGAGTACTCTAAC** | |
| NM_001414687.1 (35,819 .. 46,231) | TTGGTTTACTAGAGACTACAGGCTTACTGGCCACCAGCTCTTCAGCAGAGACCAGCACGAGTACTCTAAC | 70 |
| Kuramochi | TTGGTTTACTAGAGACTACAGGCTTACTGGCCACCAGCTCTTCAGCAGAGACCAGCACGAGTACTCTAAC | 70 |
| OVCAR3 | TTGGTTTACTAGAGACTACAGGCTTACTGGCCACCAGCTCTTCAGCAGAGACCAGCACGAGTACTCTAAC | 70 |
| OVCAR5 | TTGGTTTACTAGAGACTACAGGCTTACTGGCCACCAGCTCTTCAGCAGAGACCAGCACGAGTACTCTAAC | 70 |
| OV1 | TTGGTTTACTAGAGACTACAGGCTTACTGGCCACCAGCTCTTCAGCAGAGACCAGCACGAGTACTCTAAC | 70 |
| OV2 | TTGGTTTACTAGAGACTACAGGCTTACTGGCCACCAGCTCTTCAGCAGAGACCAGCACGAGTACTCTAAC | 70 |
| OV3 | TTGGTTTACTAGAGACTACAGGCTTACTGGCCACCAGCTCTTCAGCAGAGACCAGCACGAGTACTCTAAC | 70 |

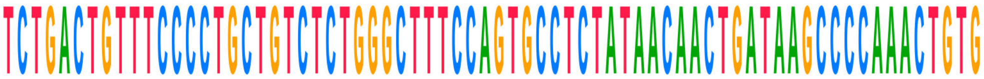

| | | |
|---|---|---|
| **Consensus** | **TCTGACTGTTTCCCCTGCTGTCTCTGGGCTTTCCAGTGCCTCTATAACAACTGATAAGCCCCAAACTGTG** | |
| NM_001414687.1 (35,819 .. 46,231) | TCTGACTGTTTCCCCTGCTGTCTCTGGGCTTTCCAGTGCCTCTATAACAACTGATAAGCCCCAAACTGTG | 140 |
| Kuramochi | TCTGACTGTTTCCCCTGCTGTCTCTGGGCTTTCCAGTGCCTCTATAACAACTGATAAGCCCCAAACTGTG | 140 |
| OVCAR3 | TCTGACTGTTTCCCCTGCTGTCTCTGGGCTTTCCAGTGCCTCTATAACAACTGATAAGCCCCAAACTGTG | 140 |
| OVCAR5 | TCTGACTGTTTCCCCTGCTGTCTCTGGGCTTTCCAGTGCCTCTATAACAACTGATAAGCCCCAAACTGTG | 140 |
| OV1 | TCTGACTGTTTCCCCTGCTGTCTCTGGGCTTTCCAGTGCCTCTATAACAACTGATAAGCCCCAAACTGTG | 140 |
| OV2 | TCTGACTGTTTCCCCTGCTGTCTCTGGGCTTTCCAGTGCCTCTATAACAACTGATAAGCCCCAAACTGTG | 140 |
| OV3 | TCTGACTGTTTCCCCTGCTGTCTCTGGGCTTTCCAGTGCCTCTATAACAACTGATAAGCCCCAAACTGTG | 140 |

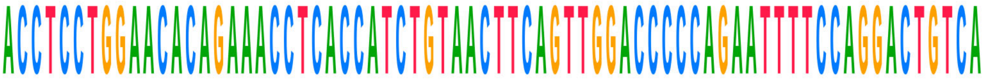

| | | |
|---|---|---|
| **Consensus** | **ACCTCCTGGAACACAGAAACCTCACCATCTGTAACTTCAGTTGGACCCCCAGAATTTTCCAGGACTGTCA** | |
| NM_001414687.1 (35,819 .. 46,231) | ACCTCCTGGAACACAGAAACCTCACCATCTGTAACTTCAGTTGGACCCCCAGAATTTTCCAGGACTGTCA | 210 |
| Kuramochi | ACCTCCTGGAACACAGAAACCTCACCATCTGTAACTTCAGTTGGACCCCCAGAATTTTCCAGGACTGTCA | 210 |
| OVCAR3 | ACCTCCTGGAACACAGAAACCTCACCATCTGTAACTTCAGTTGGACCCCCAGAATTTTCCAGGACTGTCA | 210 |
| OVCAR5 | ACCTCCTGGAACACAGAAACCTCACCATCTGTAACTTCAGTTGGACCCCCAGAATTTTCCAGGACTGTCA | 210 |
| OV1 | ACCTCCTGGAACACAGAAACCTCACCATCTGTAACTTCAGTTGGACCCCCAGAATTTTCCAGGACTGTCA | 210 |
| OV2 | ACCTCCTGGAACACAGAAACCTCACCATCTGTAACTTCAGTTGGACCCCCAGAATTTTCCAGGACTGTCA | 210 |
| OV3 | ACCTCCTGGAACACAGAAACCTCACCATCTGTAACTTCAGTTGGACCCCCAGAATTTTCCAGGACTGTCA | 210 |

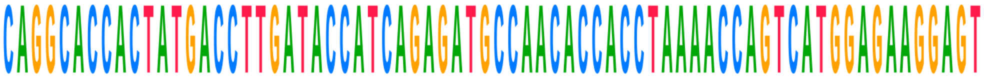

| | | |
|---|---|---|
| **Consensus** | **CAGGCACCACTATGACCTTGATACCATCAGAGATGCCAACACCACCTAAAACCAGTCATGGAGAAGGAGT** | |
| NM_001414687.1 (35,819 .. 46,231) | CAGGCACCACTATGACCTTGATACCATCAGAGATGCCAACACCACCTAAAACCAGTCATGGAGAAGGAGT | 280 |
| Kuramochi | CAGGCACCACTATGACCTTGATACCATCAGAGATGCCAACACCACCTAAAACCAGTCATGGAGAAGGAGT | 280 |
| OVCAR3 | CAGGCACCACTATGACCTTGATACCATCAGAGATGCCAACACCACCTAAAACCAGTCATGGAGAAGGAGT | 280 |
| OVCAR5 | CAGGCACCACTATGACCTTGATACCATCAGAGATGCCAACACCACCTAAAACCAGTCATGGAGAAGGAGT | 280 |
| OV1 | CAGGCACCACTATGACCTTGATACCATCAGAGATGCCAACACCACCTAAAACCAGTCATGGAGAAGGAGT | 280 |
| OV2 | CAGGCACCACTATGACCTTGATACCATCAGAGATGCCAACACCACCTAAAACCAGTCATGGAGAAGGAGT | 280 |
| OV3 | CAGGCACCACTATGACCTTGATACCATCAGAGATGCCAACACCACCTAAAACCAGTCATGGAGAAGGAGT | 280 |

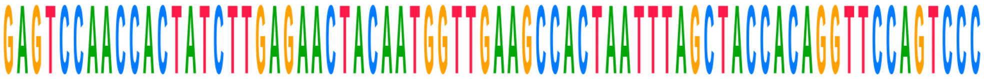

| | | |
|---|---|---|
| **Consensus** | **GAGTCCAACCACTATCTTGAGAACTACAATGGTTGAAGCCACTAATTTAGCTACCACAGGTTCCAGTCCC** | |
| NM_001414687.1 (35,819 .. 46,231) | GAGTCCAACCACTATCTTGAGAACTACAATGGTTGAAGCCACTAATTTAGCTACCACAGGTTCCAGTCCC | 350 |
| Kuramochi | GAGTCCAACCACTATCTTGAGAACTACAATGGTTGAAGCCACTAATTTAGCTACCACAGGTTCCAGTCCC | 350 |
| OVCAR3 | GAGTCCAACCACTATCTTGAGAACTACAATGGTTGAAGCCACTAATTTAGCTACCACAGGTTCCAGTCCC | 350 |
| OVCAR5 | GAGTCCAACCACTATCTTGAGAACTACAATGGTTGAAGCCACTAATTTAGCTACCACAGGTTCCAGTCCC | 350 |
| OV1 | GAGTCCAACCACTATCTTGAGAACTACAATGGTTGAAGCCACTAATTTAGCTACCACAGGTTCCAGTCCC | 350 |
| OV2 | GAGTCCAACCACTATCTTGAGAACTACAATGGTTGAAGCCACTAATTTAGCTACCACAGGTTCCAGTCCC | 350 |
| OV3 | GAGTCCAACCACTATCTTGAGAACTACAATGGTTGAAGCCACTAATTTAGCTACCACAGGTTCCAGTCCC | 350 |

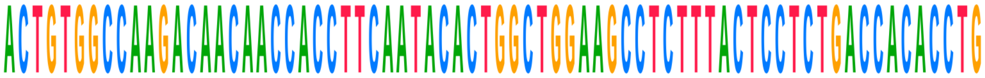

| | | |
|---|---|---|
| **Consensus** | **ACTGTGGCCAAGACAACAACCACCTTCAATACACTGGCTGGAAGCCTCTTTACTCCTCTGACCACACCTG** | |
| NM_001414687.1 (35,819 .. 46,231) | ACTGTGGCCAAGACAACAACCACCTTCAATACACTGGCTGGAAGCCTCTTTACTCCTCTGACCACACCTG | 420 |
| Kuramochi | ACTGTGGCCAAGACAACAACCACCTTCAATACACTGGCTGGAAGCCTCTTTACTCCTCTGACCACACCTG | 420 |
| OVCAR3 | ACTGTGGCCAAGACAACAACCACCTTCAATACACTGGCTGGAAGCCTCTTTACTCCTCTGACCACACCTG | 420 |
| OVCAR5 | ACTGTGGCCAAGACAACAACCACCTTCAATACACTGGCTGGAAGCCTCTTTACTCCTCTGACCACACCTG | 420 |
| OV1 | ACTGTGGCCAAGACAACAACCACCTTCAATACACTGGCTGGAAGCCTCTTTACTCCTCTGACCACACCTG | 420 |
| OV2 | ACTGTGGCCAAGACAACAACCACCTTCAATACACTGGCTGGAAGCCTCTTTACTCCTCTGACCACACCTG | 420 |
| OV3 | ACTGTGGCCAAGACAACAACCACCTTCAATACACTGGCTGGAAGCCTCTTTACTCCTCTGACCACACCTG | 420 |

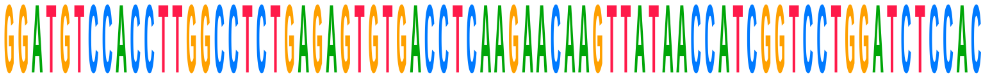

| | | |
|---|---|---|
| **Consensus** | **GGATGTCCACCTTGGCCTCTGAGAGTGTGACCTCAAGAACAAGTTATAACCATCGGTCCTGGATCTCCAC** | |
| NM_001414687.1 (35,819 .. 46,231) | GGATGTCCACCTTGGCCTCTGAGAGTGTGACCTCAAGAACAAGTTATAACCATCGGTCCTGGATCTCCAC | 490 |
| Kuramochi | GGATGTCCACCTTGGCCTCTGAGAGTGTGACCTCAAGAACAAGTTATAACCATCGGTCCTGGATCTCCAC | 490 |
| OVCAR3 | GGATGTCCACCTTGGCCTCTGAGAGTGTGACCTCAAGAACAAGTTATAACCATCGGTCCTGGATCTCCAC | 490 |
| OVCAR5 | GGATGTCCACCTTGGCCTCTGAGAGTGTGACCTCAAGAACAAGTTATAACCATCGGTCCTGGATCTCCAC | 490 |
| OV1 | GGATGTCCACCTTGGCCTCTGAGAGTGTGACCTCAAGAACAAGTTATAACCATCGGTCCTGGATCTCCAC | 490 |
| OV2 | GGATGTCCACCTTGGCCTCTGAGAGTGTGACCTCAAGAACAAGTTATAACCATCGGTCCTGGATCTCCAC | 490 |
| OV3 | GGATGTCCACCTTGGCCTCTGAGAGTGTGACCTCAAGAACAAGTTATAACCATCGGTCCTGGATCTCCAC | 490 |

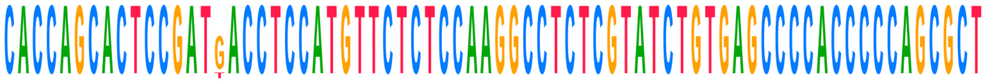

| | | |
|---|---|---|
| **Consensus** | **CACCAGCACTCCGATGACCTCCATGTTCTCTCCAAGGCCTCTCGTATCTGTGAGCCCCACCCCCAGCGCT** | |
| NM_001414687.1 (35,819 .. 46,231) | CACCAGCACTCCGATGACCTCCATGTTCTCTCCAAGGCCTCTCGTATCTGTGAGCCCCACCCCCAGCGCT | 560 |
| Kuramochi | CACCAGCACTCCGATGACCTCCATGTTCTCTCCAAGGCCTCTCGTATCTGTGAGCCCCACCCCCAGCGCT | 560 |
| OVCAR3 | CACCAGCACTCCGATGACCTCCATGTTCTCTCCAAGGCCTCTCGTATCTGTGAGCCCCACCCCCAGCGCT | 560 |
| OVCAR5 | CACCAGCACTCCGATGACCTCCATGTTCTCTCCAAGGCCTCTCGTATCTGTGAGCCCCACCCCCAGCGCT | 560 |
| OV1 | CACCAGCACTCCGATGACCTCCATGTTCTCTCCAAGGCCTCTCGTATCTGTGAGCCCCACCCCCAGCGCT | 560 |
| OV2 | CACCAGCACTCCGATTACCTCCATGTTCTCTCCAAGGCCTCTCGTATCTGTGAGCCCCACCCCCAGCGCT | 560 |
| OV3 | CACCAGCACTCCGATGACCTCCATGTTCTCTCCAAGGCCTCTCGTATCTGTGAGCCCCACCCCCAGCGCT | 560 |

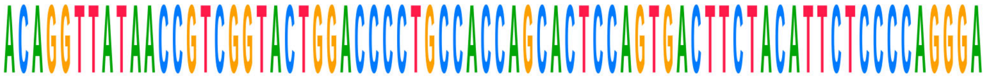

| | | |
|---|---|---|
| **Consensus** | **ACAGGTTATAACCGTCGGTACTGGACCCCTGCCACCAGCACTCCAGTGACTTCTACATTCTCCCCAGGGA** | |
| NM_001414687.1 (35,819 .. 46,231) | ACAGGTTATAACCGTCGGTACTGGACCCCTGCCACCAGCACTCCAGTGACTTCTACATTCTCCCCAGGGA | 630 |
| Kuramochi | ACAGGTTATAACCGTCGGTACTGGACCCCTGCCACCAGCACTCCAGTGACTTCTACATTCTCCCCAGGGA | 630 |
| OVCAR3 | ACAGGTTATAACCGTCGGTACTGGACCCCTGCCACCAGCACTCCAGTGACTTCTACATTCTCCCCAGGGA | 630 |
| OVCAR5 | ACAGGTTATAACCGTCGGTACTGGACCCCTGCCACCAGCACTCCAGTGACTTCTACATTCTCCCCAGGGA | 630 |
| OV1 | ACAGGTTATAACCGTCGGTACTGGACCCCTGCCACCAGCACTCCAGTGACTTCTACATTCTCCCCAGGGA | 630 |
| OV2 | ACAGGTTATAACCGTCGGTACTGGACCCCTGCCACCAGCACTCCAGTGACTTCTACATTCTCCCCAGGGA | 630 |
| OV3 | ACAGGTTATAACCGTCGGTACTGGACCCCTGCCACCAGCACTCCAGTGACTTCTACATTCTCCCCAGGGA | 630 |

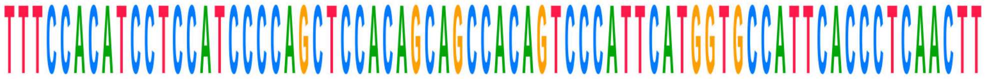

| | | |
|---|---|---|
| **Consensus** | **TTTCCACATCCTCCATCCCCAGCTCCACAGCAGCCACAGTCCCATTCATGGTGCCATTCACCCTCAACTT** | |
| NM_001414687.1 (35,819 .. 46,231) | TTTCCACATCCTCCATCCCCAGCTCCACAGCAGCCACAGTCCCATTCATGGTGCCATTCACCCTCAACTT | 700 |
| Kuramochi | TTTCCACATCCTCCATCCCCAGCTCCACAGCAGCCACAGTCCCATTCATGGTGCCATTCACCCTCAACTT | 700 |
| OVCAR3 | TTTCCACATCCTCCATCCCCAGCTCCACAGCAGCCACAGTCCCATTCATGGTGCCATTCACCCTCAACTT | 700 |
| OVCAR5 | TTTCCACATCCTCCATCCCCAGCTCCACAGCAGCCACAGTCCCATTCATGGTGCCATTCACCCTCAACTT | 700 |
| OV1 | TTTCCACATCCTCCATCCCCAGCTCCACAGCAGCCACAGTCCCATTCATGGTGCCATTCACCCTCAACTT | 700 |
| OV2 | TTTCCACATCCTCCATCCCCAGCTCCACAGCAGCCACAGTCCCATTCATGGTGCCATTCACCCTCAACTT | 700 |
| OV3 | TTTCCACATCCTCCATCCCCAGCTCCACAGCAGCCACAGTCCCATTCATGGTGCCATTCACCCTCAACTT | 700 |

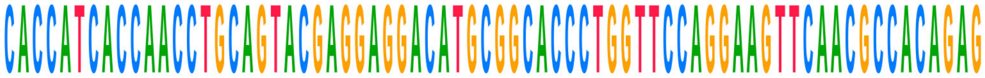

| | | |
|---|---|---|
| **Consensus** | **CACCATCACCAACCTGCAGTACGAGGAGGACATGCGGCACCCTGGTTCCAGGAAGTTCAACGCCACAGAG** | |
| NM_001414687.1 (35,819 .. 46,231) | CACCATCACCAACCTGCAGTACGAGGAGGACATGCGGCACCCTGGTTCCAGGAAGTTCAACGCCACAGAG | 770 |
| Kuramochi | CACCATCACCAACCTGCAGTACGAGGAGGACATGCGGCACCCTGGTTCCAGGAAGTTCAACGCCACAGAG | 770 |
| OVCAR3 | CACCATCACCAACCTGCAGTACGAGGAGGACATGCGGCACCCTGGTTCCAGGAAGTTCAACGCCACAGAG | 770 |
| OVCAR5 | CACCATCACCAACCTGCAGTACGAGGAGGACATGCGGCACCCTGGTTCCAGGAAGTTCAACGCCACAGAG | 770 |
| OV1 | CACCATCACCAACCTGCAGTACGAGGAGGACATGCGGCACCCTGGTTCCAGGAAGTTCAACGCCACAGAG | 770 |
| OV2 | CACCATCACCAACCTGCAGTACGAGGAGGACATGCGGCACCCTGGTTCCAGGAAGTTCAACGCCACAGAG | 770 |
| OV3 | CACCATCACCAACCTGCAGTACGAGGAGGACATGCGGCACCCTGGTTCCAGGAAGTTCAACGCCACAGAG | 770 |

| | | |
|---|---|---|
| **Consensus** | **AGAGAACTGCAGGGTCTGCTCAAACCCTTGTTCAGGAATAGCAGTCTGGAATACCTCTATTCAGGCTGCA** | |
| NM_001414687.1 (35,819 .. 46,231) | AGAGAACTGCAGGGTCTGCTCAAACCCTTGTTCAGGAATAGCAGTCTGGAATACCTCTATTCAGGCTGCA | 840 |
| Kuramochi | AGAGAACTGCAGGGTCTGCTCAAACCCTTGTTCAGGAATAGCAGTCTGGAATACCTCTATTCAGGCTGCA | 840 |
| OVCAR3 | AGAGAACTGCAGGGTCTGCTCAAACCCTTGTTCAGGAATAGCAGTCTGGAATACCTCTATTCAGGCTGCA | 840 |
| OVCAR5 | AGAGAACTGCAGGGTCTGCTCAAACCCTTGTTCAGGAATAGCAGTCTGGAATACCTCTATTCAGGCTGCA | 840 |
| OV1 | AGAGAACTGCAGGGTCTGCTCAAACCCTTGTTCAGGAATAGCAGTCTGGAATACCTCTATTCAGGCTGCA | 840 |
| OV2 | AGAGAACTGCAGGGTCTGCTCAAACCCTTGTTCAGGAATAGCAGTCTGGAATACCTCTATTCAGGCTGCA | 840 |
| OV3 | AGAGAACTGCAGGGTCTGCTCAAACCCTTGTTCAGGAATAGCAGTCTGGAATACCTCTATTCAGGCTGCA | 840 |

| | | |
|---|---|---|
| **Consensus** | **GACTAGCCTCACTCAGGCCAGAGAAGGATAGCTCAGCCACGGCAGTGGATGCCATCTGCACACATCGCCC** | |
| NM_001414687.1 (35,819 .. 46,231) | GACTAGCCTCACTCAGGCCAGAGAAGGATAGCTCAGCCACGGCAGTGGATGCCATCTGCACACATCGCCC | 910 |
| Kuramochi | GACTAGCCTCACTCAGGCCAGAGAAGGATAGCTCAGCCACGGCAGTGGATGCCATCTGCACACATCGCCC | 910 |
| OVCAR3 | GACTAGCCTCACTCAGGCCAGAGAAGGATAGCTCAGCCACGGCAGTGGATGCCATCTGCACACATCGCCC | 910 |
| OVCAR5 | GACTAGCCTCACTCAGGCCAGAGAAGGATAGCTCAGCCACGGCAGTGGATGCCATCTGCACACATCGCCC | 910 |
| OV1 | GACTAGCCTCACTCAGGCCAGAGAAGGATAGCTCAGCCACGGCAGTGGATGCCATCTGCACACATCGCCC | 910 |
| OV2 | GACTAGCCTCACTCAGGCCAGAGAAGGATAGCTCAGCCATGGCAGTGGATGCCATCTGCACACATCGCCC | 910 |
| OV3 | GACTAGCCTCACTCAGGCCAGAGAAGGATAGCTCAGCCACGGCAGTGGATGCCATCTGCACACATCGCCC | 910 |

| | | |
|---|---|---|
| **Consensus** | **TGACCCTGAAGACCTCGGACTGGACAGAGAGCGACTGTACTGGGAGCTGAGCAATCTGACAAATGGCATC** | |
| NM_001414687.1 (35,819 .. 46,231) | TGACCCTGAAGACCTCGGACTGGACAGAGAGCGACTGTACTGGGAGCTGAGCAATCTGACAAATGGCATC | 980 |
| Kuramochi | TGACCCTGAAGACCTCGGACTGGACAGAGAGCGACTGTACTGGGAGCTGAGCAATCTGACAAATGGCATC | 980 |
| OVCAR3 | TGACCCTGAAGACCTCGGACTGGACAGAGAGCGACTGTACTGGGAGCTGAGCAATCTGACAAATGGCATC | 980 |
| OVCAR5 | TGACCCTGAAGACCTCGGACTGGACAGAGAGCGACTCTACTGGGAGCTGAGCAATCTGACAAATGGCATC | 980 |
| OV1 | TGACCCTGAAGACCTCGGACTGGACAGAGAGCGACTGTACTGGGAGCTGAGCAATCTGACAAATGGCATC | 980 |
| OV2 | TGACCCTGAAGACCTCGGACTGGACAGAGAGCGACTGTACTGGGAGCTGAGCAATCTGACAAATGGCATC | 980 |
| OV3 | TGACCCTGAAGACCTCGGACTGGACAGAGAGCGACTGTACTGGGAGCTGAGCAATCTGACAAATGGCATC | 980 |

| | | |
|---|---|---|
| **Consensus** | **CAGGAGCTGGGCCCCTACACCCTGGACCGGAACAGTCTCTATGTCAATGGTTTCACCCATCGAAGCTCTA** | |
| NM_001414687.1 (35,819 .. 46,231) | CAGGAGCTGGGCCCCTACACCCTGGACCGGAACAGTCTCTATGTCAATGGTTTCACCCATCGAAGCTCTA | 1050 |
| Kuramochi | CAGGAGCTGGGCCCCTACACCCTGGACCGGAACAGTCTCTATGTCAATGGTTTCACCCATCGAAGCTCTA | 1050 |
| OVCAR3 | CAGGAGCTGGGCCCCTACACCCTGGACCGGAACAGTCTCTATGTCAATGGTTTCACCCATCGAAGCTCTA | 1050 |
| OVCAR5 | CAGGAGCTGGGCCCCTACACCCTGGACCGGAACAGTCTCTATGTCAATGGTTTCACCCATCAAAGCTCTA | 1050 |
| OV1 | CAGGAGCTGGGCCCCTACACCCTGGACCGGAACAGTCTCTATGTCAATGGTTTCACCCATCGAAGCTCTA | 1050 |
| OV2 | CAGGAGCTGGGCCCCTACACCCTGGACCGGAACAGTCTCTATGTCAATGGTTTCACCCATCGAAGCTCTA | 1050 |
| OV3 | CAGGAGCTGGGCCCCTACACCCTGGACCGGAACAGTCTCTATGTCAATGGTTTCACCCATCGAAGCTCTA | 1050 |

| | | |
|---|---|---|
| **Consensus** | **TGCCCACCACCAGCACTCCTGGGACCTCCACAGTGGATGTGGGAACCTCAGGGACTCCATCCTCCAGCCC** | |
| NM_001414687.1 (35,819 .. 46,231) | TGCCCACCACCAGCACTCCTGGGACCTCCACAGTGGATGTGGGAACCTCAGGGACTCCATCCTCCAGCCC | 1120 |
| Kuramochi | TGCCCACCACCAGCACTCCTGGGACCTCCACAGTGGATGTGGGAACCTCAGGGACTCCATCCTCCAGCCC | 1120 |
| OVCAR3 | TGCCCACCACCAGCACTCCTGGGACCTCCACAGTGGATGTGGGAACCTCAGGGACTCCATCCTCCAGCCC | 1120 |
| OVCAR5 | TGCCCACCACCAGCACTCCTGGGACCTCCACAGTGGATGTGGGAACCTCAGGGACTCCATCCTCCAGCCC | 1120 |
| OV1 | TGCCCACCACCAGCACTCCTGGGACCTCCACAGTGGATGTGGGAACCTCAGGGACTCCATCCTCCAGCCC | 1120 |
| OV2 | TGCCCACCACCAGCACTCCTGGGACCTCCACAGTGGATGTGGGAACCTCAGGGACTCCATCCTCCAGCCC | 1120 |
| OV3 | TGCCCACCACCAGCACTCCTGGGACCTCCACAGTGGATGTGGGAACCTCAGGGACTCCATCCTCCAGCCC | 1120 |

| | | |
|---|---|---|
| **Consensus** | **CAGCCCCACGACTGCTGGCCCTCTCCTGATGCCGTTCACCCTCAACTTCACCATCACCAACCTGCAGTAC** | |
| NM_001414687.1 (35,819 .. 46,231) | CAGCCCCACGACTGCTGGCCCTCTCCTGATGCCGTTCACCCTCAACTTCACCATCACCAACCTGCAGTAC | 1190 |
| Kuramochi | CAGCCCCACGACTGCTGGCCCTCTCCTGATGCCGTTCACCCTCAACTTCACCATCACCAACCTGCAGTAC | 1190 |
| OVCAR3 | CAGCCCCACGACTGCTGGCCCTCTCCTGATGCCGTTCACCCTCAACTTCACCATCACCAACCTGCAGTAC | 1190 |
| OVCAR5 | CAGCCCCACGACTGCTGGCCCTCTCCTGATGCCGTTCACCCTCAACTTCACCATCACCAACCTGCAGTAC | 1190 |
| OV1 | CAGCCCCACGACTGCTGGCCCTCTCCTGATGCCGTTCACCCTCAACTTCACCATCACCAACCTGCAGTAC | 1190 |
| OV2 | CAGCCCCACGACTGCTGGCCCTCTCCTGATGCCGTTCACCCTCAACTTCACCATCACCAACCTGCAGTAC | 1190 |
| OV3 | CAGCCCCACGACTGCTGGCCCTCTCCTGATGCCGTTCACCCTCAACTTCACCATCACCAACCTGCAGTAC | 1190 |

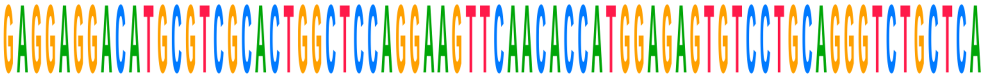

| | | |
|---|---|---|
| **Consensus** | **GAGGAGGACATGCGTCGCACTGGCTCCAGGAAGTTCAACACCATGGAGAGTGTCCTGCAGGGTCTGCTCA** | |
| NM_001414687.1 (35,819 .. 46,231) | GAGGAGGACATGCGTCGCACTGGCTCCAGGAAGTTCAACACCATGGAGAGTGTCCTGCAGGGTCTGCTCA | 1260 |
| Kuramochi | GAGGAGGACATGCGTCGCACTGGCTCCAGGAAGTTCAACACCATGGAGAGTGTCCTGCAGGGTCTGCTCA | 1260 |
| OVCAR3 | GAGGAGGACATGCGTCGCACTGGCTCCAGGAAGTTCAACACCATGGAGAGTGTCCTGCAGGGTCTGCTCA | 1260 |
| OVCAR5 | GAGGAGGACATGCGTCGCACTGGCTCCAGGAAGTTCAACACCATGGAGAGTGTCCTGCAGGGTCTGCTCA | 1260 |
| OV1 | GAGGAGGACATGCGTCGCACTGGCTCCAGGAAGTTCAACACCATGGAGAGTGTCCTGCAGGGTCTGCTCA | 1260 |
| OV2 | GAGGAGGACATGCGTCGCACTGGCTCCAGGAAGTTCAACACCATGGAGAGTGTCCTGCAGGGTCTGCTCA | 1260 |
| OV3 | GAGGAGGACATGCGTCGCACTGGCTCCAGGAAGTTCAACACCATGGAGAGTGTCCTGCAGGGTCTGCTCA | 1260 |

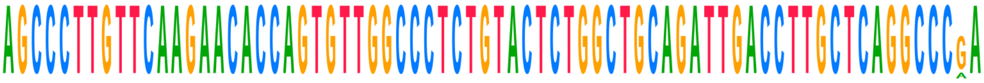

| | | |
|---|---|---|
| **Consensus** | **AGCCCTTGTTCAAGAACACCAGTGTTGGCCCTCTGTACTCTGGCTGCAGATTGACCTTGCTCAGGCCCGA** | |
| NM_001414687.1 (35,819 .. 46,231) | AGCCCTTGTTCAAGAACACCAGTGTTGGCCCTCTGTACTCTGGCTGCAGATTGACCTTGCTCAGGCCCGA | 1330 |
| Kuramochi | AGCCCTTGTTCAAGAACACCAGTGTTGGCCCTCTGTACTCTGGCTGCAGATTGACCTTGCTCAGGCCCGA | 1330 |
| OVCAR3 | AGCCCTTGTTCAAGAACACCAGTGTTGGCCCTCTGTACTCTGGCTGCAGATTGACCTTGCTCAGGCCCGA | 1330 |
| OVCAR5 | AGCCCTTGTTCAAGAACACCAGTGTTGGCCCTCTGTACTCTGGCTGCAGATTGACCTTGCTCAGGCCCGA | 1330 |
| OV1 | AGCCCTTGTTCAAGAACACCAGTGTTGGCCCTCTGTACTCTGGCTGCAGATTGACCTTGCTCAGGCCCGA | 1330 |
| OV2 | AGCCCTTGTTCAAGAACACCAGTGTTGGCCCTCTGTACTCTGGCTGCAGATTGACCTTGCTCAGGCCCAA | 1330 |
| OV3 | AGCCCTTGTTCAAGAACACCAGTGTTGGCCCTCTGTACTCTGGCTGCAGATTGACCTTGCTCAGGCCCGA | 1330 |

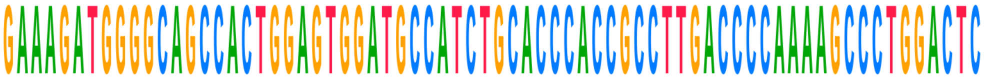

| | | |
|---|---|---|
| **Consensus** | **GAAAGATGGGGCAGCCACTGGAGTGGATGCCATCTGCACCCACCGCCTTGACCCCAAAGCCCTGGACTC** | |
| NM_001414687.1 (35,819 .. 46,231) | GAAAGATGGGGCAGCCACTGGAGTGGATGCCATCTGCACCCACCGCCTTGACCCCAAAGCCCTGGACTC | 1400 |
| Kuramochi | GAAAGATGGGGCAGCCACTGGAGTGGATGCCATCTGCACCCACCGCCTTGACCCCAAAGCCCTGGACTC | 1400 |
| OVCAR3 | GAAAGATGGGGCAGCCACTGGAGTGGATGCCATCTGCACCCACCGCCTTGACCCCAAAGCCCTGGACTC | 1400 |
| OVCAR5 | GAAAGATGGGGCAGCCACTGGAGTGGATGCCATCTGCACCCACCGCCTTGACCCCAAAGCCCTGGACTC | 1400 |
| OV1 | GAAAGATGGGGCAGCCACTGGAGTGGATGCCATCTGCACCCACCGCCTTGACCCCAAAGCCCTGGACTC | 1400 |
| OV2 | GAAAGATGGGGCAGCCACTGGAGTGGATGCCATCTGCACCCACCGCCTTGACCCCAAAGCCCTGGACTC | 1400 |
| OV3 | GAAAGATGGGGCAGCCACTGGAGTGGATGCCATCTGCACCCACCGCCTTGACCCCAAAGCCCTGGACTC | 1400 |

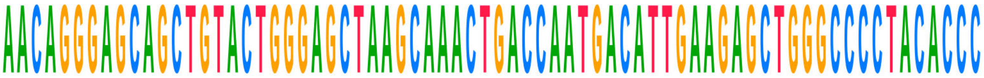

| | | |
|---|---|---|
| **Consensus** | **AACAGGGAGCAGCTGTACTGGGAGCTAAGCAAACTGACCAATGACATTGAAGAGCTGGGCCCCTACACCC** | |
| NM_001414687.1 (35,819 .. 46,231) | AACAGGGAGCAGCTGTACTGGGAGCTAAGCAAACTGACCAATGACATTGAAGAGCTGGGCCCCTACACCC | 1470 |
| Kuramochi | AACAGGGAGCAGCTGTACTGGGAGCTAAGCAAACTGACCAATGACATTGAAGAGCTGGGCCCCTACACCC | 1470 |
| OVCAR3 | AACAGGGAGCAGCTGTACTGGGAGCTAAGCAAACTGACCAATGACATTGAAGAGCTGGGCCCCTACACCC | 1470 |
| OVCAR5 | AACAGGGAGCAGCTGTACTGGGAGCTAAGCAAACTGACCAATGACATTGAAGAGCTGGGCCCCTACACCC | 1470 |
| OV1 | AACAGGGAGCAGCTGTACTGGGAGCTAAGCAAACTGACCAATGACATTGAAGAGCTGGGCCCCTACACCC | 1470 |
| OV2 | AACAGGGAGCAGCTGTACTGGGAGCTAAGCAAACTGACCAATGACATTGAAGAGCTGGGCCCCTACACCC | 1470 |
| OV3 | AACAGGGAGCAGCTGTACTGGGAGCTAAGCAAACTGACCAATGACATTGAAGAGCTGGGCCCCTACACCC | 1470 |

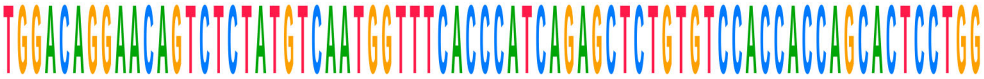

| | | |
|---|---|---|
| **Consensus** | **TGGACAGGAACAGTCTCTATGTCAATGGTTTCACCCATCAGAGCTCTGTGTCCACCACCAGCACTCCTGG** | |
| NM_001414687.1 (35,819 .. 46,231) | TGGACAGGAACAGTCTCTATGTCAATGGTTTCACCCATCAGAGCTCTGTGTCCACCACCAGCACTCCTGG | 1540 |
| Kuramochi | TGGACAGGAACAGTCTCTATGTCAATGGTTTCACCCATCAGAGCTCTGTGTCCACCACCAGCACTCCTGG | 1540 |
| OVCAR3 | TGGACAGGAACAGTCTCTATGTCAATGGTTTCACCCATCAGAGCTCTGTGTCCACCACCAGCACTCCTGG | 1540 |
| OVCAR5 | TGGACAGGAACAGTCTCTATGTCAATGGTTTCACCCATCAGAGCTCTGTGTCCACCACCAGCACTCCTGG | 1540 |
| OV1 | TGGACAGGAACAGTCTCTATGTCAATGGTTTCACCCATCAGAGCTCTGTGTCCACCACCAGCACTCCTGG | 1540 |
| OV2 | TGGACAGGAACAGTCTCTATGTCAATGGTTTCACCCATCAGAGCTCTGTGTCCACCACCAGCACTCCTGG | 1540 |
| OV3 | TGGACAGGAACAGTCTCTATGTCAATGGTTTCACCCATCAGAGCTCTGTGTCCACCACCAGCACTCCTGG | 1540 |

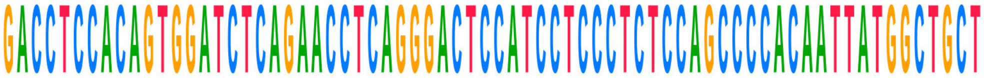

| | | |
|---|---|---|
| **Consensus** | **GACCTCCACAGTGGATCTCAGAACCTCAGGGACTCCATCCTCCCTCTCCAGCCCCACAATTATGGCTGCT** | |
| NM_001414687.1 (35,819 .. 46,231) | GACCTCCACAGTGGATCTCAGAACCTCAGGGACTCCATCCTCCCTCTCCAGCCCCACAATTATGGCTGCT | 1610 |
| Kuramochi | GACCTCCACAGTGGATCTCAGAACCTCAGGGACTCCATCCTCCCTCTCCAGCCCCACAATTATGGCTGCT | 1610 |
| OVCAR3 | GACCTCCACAGTGGATCTCAGAACCTCAGGGACTCCATCCTCCCTCTCCAGCCCCACAATTATGGCTGCT | 1610 |
| OVCAR5 | GACCTCCACAGTGGATCTCAGAACCTCAGGGACTCCATCCTCCCTCTCCAGCCCCACAATTATGGCTGCT | 1610 |
| OV1 | GACCTCCACAGTGGATCTCAGAACCTCAGGGACTCCATCCTCCCTCTCCAGCCCCACAATTATGGCTGCT | 1610 |
| OV2 | GACCTCCACAGTGGATCTCAGAACCTCAGGGACTCCATCCTCCCTCTCCAGCCCCACAATTATGGCTGCT | 1610 |
| OV3 | GACCTCCACAGTGGATCTCAGAACCTCAGGGACTCCATCCTCCCTCTCCAGCCCCACAATTATGGCTGCT | 1610 |

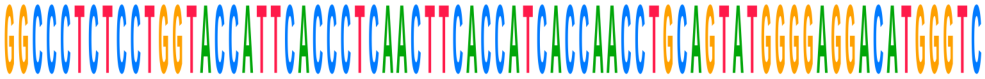

| | | |
|---|---|---|
| **Consensus** | **GGCCCTCTCCTGGTACCATTCACCCTCAACTTCACCATCACCAACCTGCAGTATGGGGAGGACATGGGTC** | |
| NM_001414687.1 (35,819 .. 46,231) | GGCCCTCTCCTGGTACCATTCACCCTCAACTTCACCATCACCAACCTGCAGTATGGGGAGGACATGGGTC | 1680 |
| Kuramochi | GGCCCTCTCCTGGTACCATTCACCCTCAACTTCACCATCACCAACCTGCAGTATGGGGAGGACATGGGTC | 1680 |
| OVCAR3 | GGCCCTCTCCTGGTACCATTCACCCTCAACTTCACCATCACCAACCTGCAGTATGGGGAGGACATGGGTC | 1680 |
| OVCAR5 | GGCCCTCTCCTGGTACCATTCACCCTCAACTTCACCATCACCAACCTGCAGTATGGGGAGGACATGGGTC | 1680 |
| OV1 | GGCCCTCTCCTGGTACCATTCACCCTCAACTTCACCATCACCAACCTGCAGTATGGGGAGGACATGGGTC | 1680 |
| OV2 | GGCCCTCTCCTGGTACCATTCACCCTCAACTTCACCATCACCAACCTGCAGTATGGGGAGGACATGGGTC | 1680 |
| OV3 | GGCCCTCTCCTGGTACCATTCACCCTCAACTTCACCATCACCAACCTGCAGTATGGGGAGGACATGGGTC | 1680 |

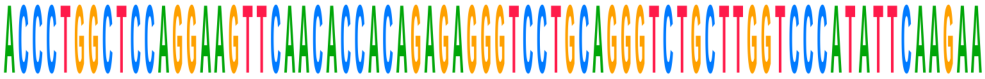

| | | |
|---|---|---|
| **Consensus** | **ACCCTGGCTCCAGGAAGTTCAACACCACAGAGAGGGTCCTGCAGGGTCTGCTTGGTCCCATATTCAAGAA** | |
| NM_001414687.1 (35,819 .. 46,231) | ACCCTGGCTCCAGGAAGTTCAACACCACAGAGAGGGTCCTGCAGGGTCTGCTTGGTCCCATATTCAAGAA | 1750 |
| Kuramochi | ACCCTGGCTCCAGGAAGTTCAACACCACAGAGAGGGTCCTGCAGGGTCTGCTTGGTCCCATATTCAAGAA | 1750 |
| OVCAR3 | ACCCTGGCTCCAGGAAGTTCAACACCACAGAGAGGGTCCTGCAGGGTCTGCTTGGTCCCATATTCAAGAA | 1750 |
| OVCAR5 | ACCCTGGCTCCAGGAAGTTCAACACCACAGAGAGGGTCCTGCAGGGTCTGCTTGGTCCCATATTCAAGAA | 1750 |
| OV1 | ACCCTGGCTCCAGGAAGTTCAACACCACAGAGAGGGTCCTGCAGGGTCTGCTTGGTCCCATATTCAAGAA | 1750 |
| OV2 | ACCCTGGCTCCAGGAAGTTCAACACCACAGAGAGGGTCCTGCAGGGTCTGCTTGGTCCCATATTCAAGAA | 1750 |
| OV3 | ACCCTGGCTCCAGGAAGTTCAACACCACAGAGAGGGTCCTGCAGGGTCTGCTTGGTCCCATATTCAAGAA | 1750 |

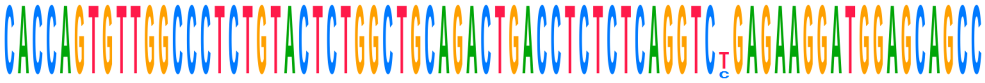

| | | |
|---|---|---|
| **Consensus** | **CACCAGTGTTGGCCCTCTGTACTCTGGCTGCAGACTGACCTCTCTCAGGTCTGAGAAGGATGGAGCAGCC** | |
| NM_001414687.1 (35,819 .. 46,231) | CACCAGTGTTGGCCCTCTGTACTCTGGCTGCAGACTGACCTCTCTCAGGTCTGAGAAGGATGGAGCAGCC | 1820 |
| Kuramochi | CACCAGTGTTGGCCCTCTGTACTCTGGCTGCAGACTGACCTCTCTCAGGTCTGAGAAGGATGGAGCAGCC | 1820 |
| OVCAR3 | CACCAGTGTTGGCCCTCTGTACTCTGGCTGCAGACTGACCTCTCTCAGGTCCGAGAAGGATGGAGCAGCC | 1820 |
| OVCAR5 | CACCAGTGTTGGCCCTCTGTACTCTGGCTGCAGACTGACCTCTCTCAGGTCTGAGAAGGATGGAGCAGCC | 1820 |
| OV1 | CACCAGTGTTGGCCCTCTGTACTCTGGCTGCAGACTGACCTCTCTCAGGTCCGAGAAGGATGGAGCAGCC | 1820 |
| OV2 | CACCAGTGTTGGCCCTCTGTACTCTGGCTGCAGACTGACCTCTCTCAGGTCTGAGAAGGATGGAGCAGCC | 1820 |
| OV3 | CACCAGTGTTGGCCCTCTGTACTCTGGCTGCAGACTGACCTCTCTCAGGTCTGAGAAGGATGGAGCAGCC | 1820 |

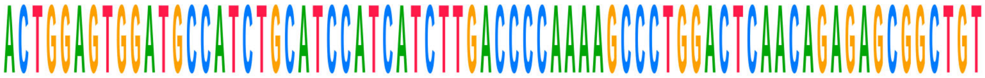

| | | |
|---|---|---|
| **Consensus** | **ACTGGAGTGGATGCCATCTGCATCCATCATCTTGACCCCAAAAGCCCTGGACTCAACAGAGAGCGGCTGT** | |
| NM_001414687.1 (35,819 .. 46,231) | ACTGGAGTGGATGCCATCTGCATCCATCATCTTGACCCCAAAAGCCCTGGACTCAACAGAGAGCGGCTGT | 1890 |
| Kuramochi | ACTGGAGTGGATGCCATCTGCATCCATCATCTTGACCCCAAAAGCCCTGGACTCAACAGAGAGCGGCTGT | 1890 |
| OVCAR3 | ACTGGAGTGGATGCCATCTGCATCCATCATCTTGACCCCAAAAGCCCTGGACTCAACAGAGAGCGGCTGT | 1890 |
| OVCAR5 | ACTGGAGTGGATGCCATCTGCATCCATCATCTTGACCCCAAAAGCCCTGGACTCAACAGAGAGCGGCTGT | 1890 |
| OV1 | ACTGGAGTGGATGCCATCTGCATCCATCATCTTGACCCCAAAAGCCCTGGACTCAACAGAGAGCGGCTGT | 1890 |
| OV2 | ACTGGAGTGGATGCCATCTGCATCCATCATCTTGACCCCAAAAGCCCTGGACTCAACAGAGAGCGGCTGT | 1890 |
| OV3 | ACTGGAGTGGATGCCATCTGCATCCATCATCTTGACCCCAAAAGCCCTGGACTCAACAGAGAGCGGCTGT | 1890 |

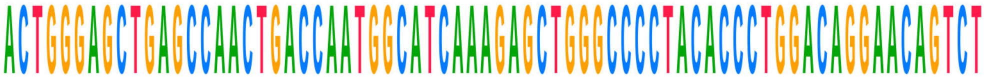

| | | |
|---|---|---|
| **Consensus** | **ACTGGGAGCTGAGCCAACTGACCAATGGCATCAAAGAGCTGGGCCCCTACACCCTGGACAGGAACAGTCT** | |
| NM_001414687.1 (35,819 .. 46,231) | ACTGGGAGCTGAGCCAACTGACCAATGGCATCAAAGAGCTGGGCCCCTACACCCTGGACAGGAACAGTCT | 1960 |
| Kuramochi | ACTGGGAGCTGAGCCAACTGACCAATGGCATCAAAGAGCTGGGCCCCTACACCCTGGACAGGAACAGTCT | 1960 |
| OVCAR3 | ACTGGGAGCTGAGCCAACTGACCAATGGCATCAAAGAGCTGGGCCCCTACACCCTGGACAGGAACAGTCT | 1960 |
| OVCAR5 | ACTGGGAGCTGAGCCAACTGACCAATGGCATCAAAGAGCTGGGCCCCTACACCCTGGACAGGAACAGTCT | 1960 |
| OV1 | ACTGGGAGCTGAGCCAACTGACCAATGGCATCAAAGAGCTGGGCCCCTACACCCTGGACAGGAACAGTCT | 1960 |
| OV2 | ACTGGGAGCTGAGCCAACTGACCAATGGCATCAAAGAGCTGGGCCCCTACACCCTGGACAGGAACAGTCT | 1960 |
| OV3 | ACTGGGAGCTGAGCCAACTGACCAATGGCATCAAAGAGCTGGGCCCCTACACCCTGGACAGGAACAGTCT | 1960 |

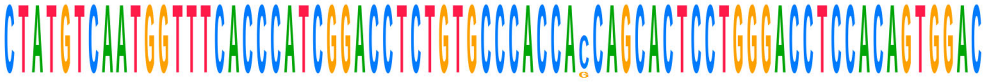

| | | |
|---|---|---|
| **Consensus** | **CTATGTCAATGGTTTCACCCATCGGACCTCTGTGCCCACCACCAGCACTCCTGGGACCTCCACAGTGGAC** | |
| NM_001414687.1 (35,819 .. 46,231) | CTATGTCAATGGTTTCACCCATCGGACCTCTGTGCCCACCAGCAGCACTCCTGGGACCTCCACAGTGGAC | 2030 |
| Kuramochi | CTATGTCAATGGTTTCACCCATCGGACCTCTGTGCCCACCACCAGCACTCCTGGGACCTCCACAGTGGAC | 2030 |
| OVCAR3 | CTATGTCAATGGTTTCACCCATCGGACCTCTGTGCCCACCACCAGCACTCCTGGGACCTCCACAGTGGAC | 2030 |
| OVCAR5 | CTATGTCAATGGTTTCACCCATCGGACCTCTGTGCCCACCACCAGCACTCCTGGGACCTCCACAGTGGAC | 2030 |
| OV1 | CTATGTCAATGGTTTCACCCATCGGACCTCTGTGCCCACCACCAGCACTCCTGGGACCTCCACAGTGGAC | 2030 |
| OV2 | CTATGTCAATGGTTTCACCCATCGGACCTCTGTGCCCACCACCAGCACTCCTGGGACCTCCACAGTGGAC | 2030 |
| OV3 | CTATGTCAATGGTTTCACCCATCGGACCTCTGTGCCCACCACCAGCACTCCTGGGACCTCCACAGTGGAC | 2030 |

| | | |
|---|---|---|
| **Consensus** | **CTTGGAACCTCAGGGACTCCATTCTCCCTCCCAAGCCCCGCAACTGCTGGCCCTCTCCTGGTGCTGTTCA** | |
| NM_001414687.1 (35,819 .. 46,231) | CTTGGAACCTCAGGGACTCCATTCTCCCTCCCAAGCCCCGCAACTGCTGGCCCTCTCCTGGTGCTGTTCA | 2100 |
| Kuramochi | CTTGGAACCTCAGGGACTCCATTCTCCCTCCCAAGCCCCGCAACTGCTGGCCCTCTCCTGGTGCTGTTCA | 2100 |
| OVCAR3 | CTTGGAACCTCAGGGACTCCATTCTCCCTCCCAAGCCCCGCAACTGCTGGCCCTCTCCTGGTGCTGTTCA | 2100 |
| OVCAR5 | CTTGGAACCTCAGGGACTCCATTCTCCCTCCCAAGCCCCGCAACTGCTGGCCCTCTCCTGGTGCTGTTCA | 2100 |
| OV1 | CTTGGAACCTCAGGGACTCCATTCTCCCTCCCAAGCCCCGCAACTGCTGGCCCTCTCCTGGTGCTGTTCA | 2100 |
| OV2 | CTTGGAACCTCAGGGACTCCATTCTCCCTCCCAAGCCCCGCAACTGCTGGCCCTCTCCTGGTGCTGTTCA | 2100 |
| OV3 | CTTGGAACCTCAGGGACTCCATTCTCCCTCCCAAGCCCCGCAACTGCTGGCCCTCTCCTGGTGCTGTTCA | 2100 |

| | | |
|---|---|---|
| **Consensus** | **CCCTCAACTTCACCATCACCAACCTGAAGTATGAGGAGGACATGCATCGCCCTGGCTCCAGGAAGTTCAA** | |
| NM_001414687.1 (35,819 .. 46,231) | CCCTCAACTTCACCATCACCAACCTGAAGTATGAGGAGGACATGCATCGCCCTGGCTCCAGGAAGTTCAA | 2170 |
| Kuramochi | CCCTCAACTTCACCATCACCAACCTGAAGTATGAGGAGGACATGCATCGCCCTGGCTCCAGGAAGTTCAA | 2170 |
| OVCAR3 | CCCTCAACTTCACCATCACCAACCTGAAGTATGAGGAGGACATGCATCGCCCTGGCTCCAGGAAGTTCAA | 2170 |
| OVCAR5 | CCCTCAACTTCACCATCACCAACCTGAAGTATGAGGAGGACATGCATCGCCCTGGCTCCAGGAAGTTCAA | 2170 |
| OV1 | CCCTCAACTTCACCATCACCAACCTGAAGTATGAGGAGGACATGCATCGCCCTGGCTCCAGGAAGTTCAA | 2170 |
| OV2 | CCCTCAACTTCACCATCACCAACCTGAAGTATGAGGAGGACATGCATCGCCCTGGCTCCAGGAAGTTCAA | 2170 |
| OV3 | CCCTCAACTTCACCATCACCAACCTGAAGTATGAGGAGGACATGCATCGCCCTGGCTCCAGGAAGTTCAA | 2170 |

| | | |
|---|---|---|
| **Consensus** | **CACCACTGAGAGGGTCCTGCAGACTCTGCTTGGTCCTATGTTCAAGAACACCAGTGTTGGCCTTCTGTAC** | |
| NM_001414687.1 (35,819 .. 46,231) | CACCACTGAGAGGGTCCTGCAGACTCTGCTTGGTCCTATGTTCAAGAACACCAGTGTTGGCCTTCTGTAC | 2240 |
| Kuramochi | CACCACTGAGAGGGTCCTGCAGACTCTGCTTGGTCCTATGTTCAAGAACACCAGTGTTGGCCTTCTGTAC | 2240 |
| OVCAR3 | CACCACTGAGAGGGTCCTGCAGACTCTGCTTGGTCCTATGTTCAAGAACACCAGTGTTGGCCTTCTGTAC | 2240 |
| OVCAR5 | CACCACTGAGAGGGTCCTGCAGACTCTGCTTGGTCCTATGTTCAAGAACACCAGTGTTGGCCTTCTGTAC | 2240 |
| OV1 | CACCACTGAGAGGGTCCTGCAGACTCTGCTTGGTCCTATGTTCAAGAACACCAGTGTTGGCCTTCTGTAC | 2240 |
| OV2 | CACCACTGAGAGGGTCCTGCAGACTCTGCTTGGTCCTATGTTCAAGAACACCAGTGTTGGCCTTCTGTAC | 2240 |
| OV3 | CACCACTGAGAGGGTCCTGCAGACTCTGCTTGGTCCTATGTTCAAGAACACCAGTGTTGGCCTTCTGTAC | 2240 |

| | | |
|---|---|---|
| **Consensus** | **TCTGGCTGCAGACTGACCTTGCTCAGGTCCGAGAAGGATGGAGCAGCCACTGGAGTGGATGCCATCTGCA** | |
| NM_001414687.1 (35,819 .. 46,231) | TCTGGCTGCAGACTGACCTTGCTCAGGTCCGAGAAGGATGGAGCAGCCACTGGAGTGGATGCCATCTGCA | 2310 |
| Kuramochi | TCTGGCTGCAGACTGACCTTGCTCAGGTCCGAGAAGGATGGAGCAGCCACTGGAGTGGATGCCATCTGCA | 2310 |
| OVCAR3 | TCTGGCTGCAGACTGACCTTGCTCAGGTCCGAGAAGGATGGAGCAGCCACTGGAGTGGATGCCATCTGCA | 2310 |
| OVCAR5 | TCTGGCTGCAGACTGACCTTGCTCAGGTCCGAGAAGGATGGAGCAGCCACTGGAGTGGATGCCATCTGCA | 2310 |
| OV1 | TCTGGCTGCAGACTGACCTTGCTCAGGTCCGAGAAGGATGGAGCAGCCACTGGAGTGGATGCCATCTGCA | 2310 |
| OV2 | TCTGGCTGCAGACTGACCTTGCTCAGGTCCGAGAAGGATGGAGCAGCCACTGGAGTGGATGCCATCTGCA | 2310 |
| OV3 | TCTGGCTGCAGACTGACCTTGCTCAGGTCCGAGAAGGATGGAGCAGCCACTGGAGTGGATGCCATCTGCA | 2310 |

| | | |
|---|---|---|
| **Consensus** | **CCCACCGTCTTGACCCCAAAAGCCCTGGAGTGGACAGGGAGCAGCTATACTGGGAGCTGAGCCAGCTGAC** | |
| NM_001414687.1 (35,819 .. 46,231) | CCCACCGTCTTGACCCCAAAAGCCCTGGAGTGGACAGGGAGCAGCTATACTGGGAGCTGAGCCAGCTGAC | 2380 |
| Kuramochi | CCCACCGTCTTGACCCCAAAAGCCCTGGAGTGGACAGGGAGCAGCTATACTGGGAGCTGAGCCAGCTGAC | 2380 |
| OVCAR3 | CCCACCGTCTTGACCCCAAAAGCCCTGGAGTGGACAGGGAGCAGCTATACTGGGAGCTGAGCCAGCTGAC | 2380 |
| OVCAR5 | CCCACCGTCTTGACCCCAAAAGCCCTGGAGTGGACAGGGAGCAGCTATACTGGGAGCTGAGCCAGCTGAC | 2380 |
| OV1 | CCCACCGTCTTGACCCCAAAAGCCCTGGAGTGGACAGGGAGCAGCTATACTGGGAGCTGAGCCAGCTGAC | 2380 |
| OV2 | CCCACCGTCTTGACCCCAAAAGCCCTGGAGTGGACAGGGAGCAGCTATACTGGGAGCTGAGCCAGCTGAC | 2380 |
| OV3 | CCCACCGTCTTGACCCCAAAAGCCCTGGAGTGGACAGGGAGCAGCTATACTGGGAGCTGAGCCAGCTGAC | 2380 |

| | | |
|---|---|---|
| **Consensus** | **CAATGGCATCAAAGAGCTGGGCCCCTACACCCTGGACAGGAACAGTCTCTATGTCAATGGTTTCACCCAT** | |
| NM_001414687.1 (35,819 .. 46,231) | CAATGGCATCAAAGAGCTGGGCCCCTACACCCTGGACAGGAACAGTCTCTATGTCAATGGTTTCACCCAT | 2450 |
| Kuramochi | CAATGGCATCAAAGAGCTGGGCCCCTACACCCTGGACAGGAACAGTCTCTATGTCAATGGTTTCACCCAT | 2450 |
| OVCAR3 | CAATGGCATCAAAGAGCTGGGCCCCTACACCCTGGACAGGAACAGTCTCTATGTCAATGGTTTCACCCAT | 2450 |
| OVCAR5 | CAATGGCATCAAAGAGCTGGGCCCCTACACCCTGGACAGGAACAGTCTCTATGTCAATGGTTTCACCCAT | 2450 |
| OV1 | CAATGGCATCAAAGAGCTGGGCCCCTACACCCTGGACAGGAACAGTCTCTATGTCAATGGTTTCACCCAT | 2450 |
| OV2 | CAATGGCATCAAAGAGCTGGGCCCCTACACCCTGGACAGGAACAGTCTCTATGTCAATGGTTTCACCCAT | 2450 |
| OV3 | CAATGGCATCAAAGAGCTGGGCCCCTACACCCTGGACAGGAACAGTCTCTATGTCAATGGTTTCACCCAT | 2450 |

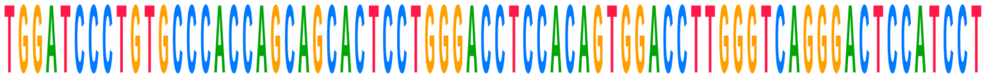

| | | |
|---|---|---|
| **Consensus** | **TGGATCCCTGTGCCCACCAGCAGCACTCCTGGGACCTCCACAGTGGACCTTGGGTCAGGGACTCCATCCT** | |
| NM_001414687.1 (35,819 .. 46,231) | TGGATCCCTGTGCCCACCAGCAGCACTCCTGGGACCTCCACAGTGGACCTTGGGTCAGGGACTCCATCCT | 2520 |
| Kuramochi | TGGATCCCTGTGCCCACCAGCAGCACTCCTGGGACCTCCACAGTGGACCTTGGGTCAGGGACTCCATCCT | 2520 |
| OVCAR3 | TGGATCCCTGTGCCCACCAGCAGCACTCCTGGGACCTCCACAGTGGACCTTGGGTCAGGGACTCCATCCT | 2520 |
| OVCAR5 | TGGATCCCTGTGCCCACCAGCAGCACTCCTGGGACCTCCACAGTGGACCTTGGGTCAGGGACTCCATCCT | 2520 |
| OV1 | TGGATCCCTGTGCCCACCAGCAGCACTCCTGGGACCTCCACAGTGGACCTTGGGTCAGGGACTCCATCCT | 2520 |
| OV2 | TGGATCCCTGTGCCCACCAGCAGCACTCCTGGGACCTCCACAGTGGACCTTGGGTCAGGGACTCCATCCT | 2520 |
| OV3 | TGGATCCCTGTGCCCACCAGCAGCACTCCTGGGACCTCCACAGTGGACCTTGGGTCAGGGACTCCATCCT | 2520 |

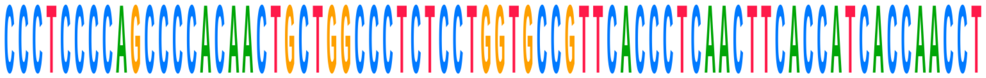

| | | |
|---|---|---|
| **Consensus** | **CCCTCCCCAGCCCCACAACTGCTGGCCCTCTCCTGGTGCCGTTCACCCTCAACTTCACCATCACCAACCT** | |
| NM_001414687.1 (35,819 .. 46,231) | CCCTCCCCAGCCCCACAACTGCTGGCCCTCTCCTGGTGCCGTTCACCCTCAACTTCACCATCACCAACCT | 2590 |
| Kuramochi | CCCTCCCCAGCCCCACAACTGCTGGCCCTCTCCTGGTGCCGTTCACCCTCAACTTCACCATCACCAACCT | 2590 |
| OVCAR3 | CCCTCCCCAGCCCCACAACTGCTGGCCCTCTCCTGGTGCCGTTCACCCTCAACTTCACCATCACCAACCT | 2590 |
| OVCAR5 | CCCTCCCCAGCCCCACAACTGCTGGCCCTCTCCTGGTGCCGTTCACCCTCAACTTCACCATCACCAACCT | 2590 |
| OV1 | CCCTCCCCAGCCCCACAACTGCTGGCCCTCTCCTGGTGCCGTTCACCCTCAACTTCACCATCACCAACCT | 2590 |
| OV2 | CCCTCCCCAGCCCCACAACTGCTGGCCCTCTCCTGGTGCCGTTCACCCTCAACTTCACCATCACCAACCT | 2590 |
| OV3 | CCCTCCCCAGCCCCACAACTGCTGGCCCTCTCCTGGTGCCGTTCACCCTCAACTTCACCATCACCAACCT | 2590 |

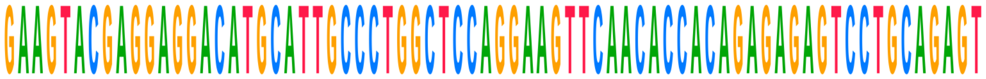

| | | |
|---|---|---|
| **Consensus** | **GAAGTACGAGGAGGACATGCATTGCCCTGGCTCCAGGAAGTTCAACACCACAGAGAGAGTCCTGCAGAGT** | |
| NM_001414687.1 (35,819 .. 46,231) | GAAGTACGAGGAGGACATGCATTGCCCTGGCTCCAGGAAGTTCAACACCACAGAGAGAGTCCTGCAGAGT | 2660 |
| Kuramochi | GAAGTACGAGGAGGACATGCATTGCCCTGGCTCCAGGAAGTTCAACACCACAGAGAGAGTCCTGCAGAGT | 2660 |
| OVCAR3 | GAAGTACGAGGAGGACATGCATTGCCCTGGCTCCAGGAAGTTCAACACCACAGAGAGAGTCCTGCAGAGT | 2660 |
| OVCAR5 | GAAGTACGAGGAGGACATGCATTGCCCTGGCTCCAGGAAGTTCAACACCACAGAGAGAGTCCTGCAGAGT | 2660 |
| OV1 | GAAGTACGAGGAGGACATGCATTGCCCTGGCTCCAGGAAGTTCAACACCACAGAGAGAGTCCTGCAGAGT | 2660 |
| OV2 | GAAGTACGAGGAGGACATGCATTGCCCTGGCTCCAGGAAGTTCAACACCACAGAGAGAGTCCTGCAGAGT | 2660 |
| OV3 | GAAGTACGAGGAGGACATGCATTGCCCTGGCTCCAGGAAGTTCAACACCACAGAGAGAGTCCTGCAGAGT | 2660 |

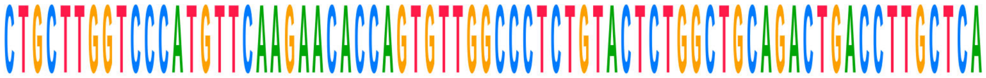

| | | |
|---|---|---|
| **Consensus** | **CTGCTTGGTCCCATGTTCAAGAACACCAGTGTTGGCCCTCTGTACTCTGGCTGCAGACTGACCTTGCTCA** | |
| NM_001414687.1 (35,819 .. 46,231) | CTGCTTGGTCCCATGTTCAAGAACACCAGTGTTGGCCCTCTGTACTCTGGCTGCAGACTGACCTTGCTCA | 2730 |
| Kuramochi | CTGCTTGGTCCCATGTTCAAGAACACCAGTGTTGGCCCTCTGTACTCTGGCTGCAGACTGACCTTGCTCA | 2730 |
| OVCAR3 | CTGCTTGGTCCCATGTTCAAGAACACCAGTGTTGGCCCTCTGTACTCTGGCTGCAGACTGACCTTGCTCA | 2730 |
| OVCAR5 | CTGCTTGGTCCCATGTTCAAGAACACCAGTGTTGGCCCTCTGTACTCTGGCTGCAGACTGACCTTGCTCA | 2730 |
| OV1 | CTGCTTGGTCCCATGTTCAAGAACACCAGTGTTGGCCCTCTGTACTCTGGCTGCAGACTGACCTTGCTCA | 2730 |
| OV2 | CTGCTTGGTCCCATGTTCAAGAACACCAGTGTTGGCCCTCTGTACTCTGGCTGCAGACTGACCTTGCTCA | 2730 |
| OV3 | CTGCTTGGTCCCATGTTCAAGAACACCAGTGTTGGCCCTCTGTACTCTGGCTGCAGACTGACCTTGCTCA | 2730 |

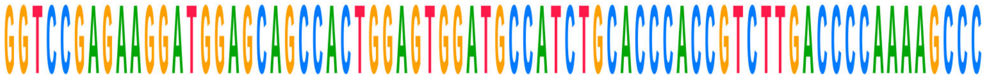

| | | |
|---|---|---|
| **Consensus** | **GGTCCGAGAAGGATGGAGCAGCCACTGGAGTGGATGCCATCTGCACCCACCGTCTTGACCCCAAAAGCCC** | |
| NM_001414687.1 (35,819 .. 46,231) | GGTCCGAGAAGGATGGAGCAGCCACTGGAGTGGATGCCATCTGCACCCACCGTCTTGACCCCAAAAGCCC | 2800 |
| Kuramochi | GGTCCGAGAAGGATGGAGCAGCCACTGGAGTGGATGCCATCTGCACCCACCGTCTTGACCCCAAAAGCCC | 2800 |
| OVCAR3 | GGTCCGAGAAGGATGGAGCAGCCACTGGAGTGGATGCCATCTGCACCCACCGTCTTGACCCCAAAAGCCC | 2800 |
| OVCAR5 | GGTCCGAGAAGGATGGAGCAGCCACTGGAGTGGATGCCATCTGCACCCACCGTCTTGACCCCAAAAGCCC | 2800 |
| OV1 | GGTCCGAGAAGGATGGAGCAGCCACTGGAGTGGATGCCATCTGCACCCACCGTCTTGACCCCAAAAGCCC | 2800 |
| OV2 | GGTCCGAGAAGGATGGAGCAGCCACTGGAGTGGATGCCATCTGCACCCACCGTCTTGACCCCAAAAGCCC | 2800 |
| OV3 | GGTCCGAGAAGGATGGAGCAGCCACTGGAGTGGATGCCATCTGCACCCACCGTCTTGACCCCAAAAGCCC | 2800 |

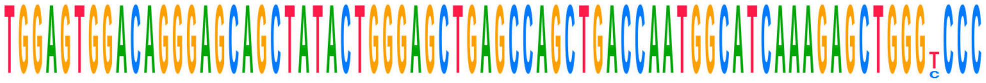

| | | |
|---|---|---|
| **Consensus** | **TGGAGTGGACAGGGAGCAGCTATACTGGGAGCTGAGCCAGCTGACCAATGGCATCAAAGAGCTGGGTCCC** | |
| NM_001414687.1 (35,819 .. 46,231) | TGGAGTGGACAGGGAGCAGCTATACTGGGAGCTGAGCCAGCTGACCAATGGCATCAAAGAGCTGGGTCCC | 2870 |
| Kuramochi | TGGAGTGGACAGGGAGCAGCTATACTGGGAGCTGAGCCAGCTGACCAATGGCATCAAAGAGCTGGGCCCC | 2870 |
| OVCAR3 | TGGAGTGGACAGGGAGCAGCTATACTGGGAGCTGAGCCAGCTGACCAATGGCATCAAAGAGCTGGGTCCC | 2870 |
| OVCAR5 | TGGAGTGGACAGGGAGCAGCTATACTGGGAGCTGAGCCAGCTGACCAATGGCATCAAAGAGCTGGGCCCC | 2870 |
| OV1 | TGGAGTGGACAGGGAGCAGCTATACTGGGAGCTGAGCCAGCTGACCAATGGCATCAAAGAGCTGGGTCCC | 2870 |
| OV2 | TGGAGTGGACAGGGAGCAGCTATACTGGGAGCTGAGCCAGCTGACCAATGGCATCAAAGAGCTGGGTCCC | 2870 |
| OV3 | TGGAGTGGACAGGGAGCAGCTATACTGGGAGCTGAGCCAGCTGACCAATGGCATCAAAGAGCTGGGTCCC | 2870 |

| | | |
|---|---|---|
| **Consensus** | **TACACCCTGGACAGAAACAGTCTCTATGTCAATGGTTTCACCCATCAGACCTCTGCGCCCAACACCAGCA** | |
| NM_001414687.1 (35,819 .. 46,231) | TACACCCTGGACAGAAACAGTCTCTATGTCAATGGTTTCACCCATCAGACCTCTGCGCCCAACACCAGCA | 2940 |
| Kuramochi | TACACCCCGGACAGGAACAGTCTCTATGTCAATGGTTTCACCCATCAGACCTCTGCGCCCAACACCAGCA | 2940 |
| OVCAR3 | TACACCCTGGACAGAAACAGTCTCTATGTCAATGGTTTCACCCATCAGACCTCTGCGCCCAACACCAGCA | 2940 |
| OVCAR5 | TACACCCTGGACAGGAACAGTCTCTATGTCAATGGTTTCACCCATCAGACCTCTGCGCCCAACACCAGCA | 2940 |
| OV1 | TACACCCTGGACAGAAACAGTCTCTATGTCAATGGTTTCACCCATCAGACCTCTGCGCCCAACACCAGCA | 2940 |
| OV2 | TACACCCTGGACAGAAACAGTCTCTATGTCAATGGTTTCACCCATCAGACCTCTGCGCCCAACACCAGCA | 2940 |
| OV3 | TACACCCTGGACAGAAACAGTCTCTATGTCAATGGTTTCACCCATCAGACCTCTGCGCCCAACACCAGCA | 2940 |

| | | |
|---|---|---|
| **Consensus** | **CTCCTGGGACCTCCACAGTGGACCTTGGGACCTCAGGGACTCCATCCTCCCTCCCCAGCCCTACATCTGC** | |
| NM_001414687.1 (35,819 .. 46,231) | CTCCTGGGACCTCCACAGTGGACCTTGGGACCTCAGGGACTCCATCCTCCCTCCCCAGCCCTACATCTGC | 3010 |
| Kuramochi | CTCCTGGGACCTCCACAGTGGACCTTGGGACCTCAGGGACTCCATCCTCCCTCCCCAGCCCTACATCTGC | 3010 |
| OVCAR3 | CTCCTGGGACCTCCACAGTGGACCTTGGGACCTCAGGGACTCCATCCTCCCTCCCCAGCCCTACATCTGC | 3010 |
| OVCAR5 | CTCCTGGGACCTCCACAGTGGACCTTGGGACCTCAGGGACTCCATCCTCCCTCCCCAGCCCTACATCTGC | 3010 |
| OV1 | CTCCTGGGACCTCCACAGTGGACCTTGGGACCTCAGGGACTCCATCCTCCCTCCCCAGCCCTACATCTGC | 3010 |
| OV2 | CTCCTGGGACCTCCACAGTGGACCTTGGGACCTCAGGGACTCCATCCTCCCTCCCCAGCCCTACATCTGC | 3010 |
| OV3 | CTCCTGGGACCTCCACAGTGGACCTTGGGACCTCAGGGACTCCATCCTCCCTCCCCAGCCCTACATCTGC | 3010 |

| | | |
|---|---|---|
| **Consensus** | **TGGCCCTCTCCTGGTGCCATTCACCCTCAACTTCACCATCACCAACCTGCAGTACGAGGAGGACATGCAT** | |
| NM_001414687.1 (35,819 .. 46,231) | TGGCCCTCTCCTGGTGCCATTCACCCTCAACTTCACCATCACCAACCTGCAGTACGAGGAGGACATGCAT | 3080 |
| Kuramochi | TGGCCCTCTCCTGGTGCCATTCACCCTCAACTTCACCATCACCAACCTGCAGTACGAGGAGGACATGCAT | 3080 |
| OVCAR3 | TGGCCCTCTCCTGGTGCCATTCACCCTCAACTTCACCATCACCAACCTGCAGTACGAGGAGGACATGCAT | 3080 |
| OVCAR5 | TGGCCCTCTCCTGGTGCCATTCACCCTCAACTTCACCATCACCAACCTGCAGTACGAGGAGGACATGCAT | 3080 |
| OV1 | TGGCCCTCTCCTGGTGCCATTCACCCTCAACTTCACCATCACCAACCTGCAGTACGAGGAGGACATGCAT | 3080 |
| OV2 | TGGCCCTCTCCTGGTGCCATTCACCCTCAACTTCACCATCACCAACCTGCAGTACGAGGAGGACATGCAT | 3080 |
| OV3 | TGGCCCTCTCCTGGTGCCATTCACCCTCAACTTCACCATCACCAACCTGCAGTACGAGGAGGACATGCAT | 3080 |

| | | |
|---|---|---|
| **Consensus** | **CACCCAGGCTCCAGGAAGTTCAACACCACGGAGCGGGTCCTGCAGGGTCTGCTTGGTCCCATGTTCAAGA** | |
| NM_001414687.1 (35,819 .. 46,231) | CACCCAGGCTCCAGGAAGTTCAACACCACGGAGCGGGTCCTGCAGGGTCTGCTTGGTCCCATGTTCAAGA | 3150 |
| Kuramochi | CACCCAGGCTCCAGGAAGTTCAACACCACGGAGCGGGTCCTGCAGGGTCTGCTTGGTCCCATGTTCAAGA | 3150 |
| OVCAR3 | CACCCAGGCTCCAGGAAGTTCAACACCACGGAGCGGGTCCTGCAGGGTCTGCTTGGTCCCATGTTCAAGA | 3150 |
| OVCAR5 | CACCCAGGCTCCAGGAAGTTCAACACCACGGAGCGGGTCCTGCAGGGTCTGCTTGGTCCCATGTTCAAGA | 3150 |
| OV1 | CACCCAGGCTCCAGGAAGTTCAACACCACGGAGCGGGTCCTGCAGGGTCTGCTTGGTCCCATGTTCAAGA | 3150 |
| OV2 | CACCCAGGCTCCAGGAAGTTCAACACCACGGAGCGGGTCCTGCAGGGTCTGCTTGGTCCCATGTTCAAGA | 3150 |
| OV3 | CACCCAGGCTCCAGGAAGTTCAACACCACGGAGCGGGTCCTGCAGGGTCTGCTTGGTCCCATGTTCAAGA | 3150 |

| | | |
|---|---|---|
| **Consensus** | **ACACCAGTGTCGGCCTTCTGTACTCTGGCTGCAGACTGACCTTGCTCAGGCCTGAGAAGAATGGGGCAGC** | |
| NM_001414687.1 (35,819 .. 46,231) | ACACCAGTGTCGGCCTTCTGTACTCTGGCTGCAGACTGACCTTGCTCAGGCCTGAGAAGAATGGGGCAGC | 3220 |
| Kuramochi | ACACCAGTGTCGGCCTTCTGTACTCTGGCTGCAGACTGACCTTGCTCAGGCCTGAGAAGAATGGGGCAGC | 3220 |
| OVCAR3 | ACACCAGTGTCGGCCTTCTGTACTCTGGCTGCAGACTGACCTTGCTCAGGCCTGAGAAGAATGGGGCAGC | 3220 |
| OVCAR5 | ACACCAGTGTCGGCCTTCTGTACTCTGGCTGCAGACTGACCTTGCTCAGGCCTGAGAAGAATGGGGCAGC | 3220 |
| OV1 | ACACCAGTGTCGGCCTTCTGTACTCTGGCTGCAGACTGACCTTGCTCAGGCCTGAGAAGAATGGGGCAGC | 3220 |
| OV2 | ACACCAGTGTCGGCCTTCTGTACTCTGGCTGCAGACTGACCTTGCTCAGGCCTGAGAAGAATGGGGCAGC | 3220 |
| OV3 | ACACCAGTGTCGGCCTTCTGTACTCTGGCTGCAGACTGACCTTGCTCAGGCCTGAGAAGAATGGGGCAGC | 3220 |

| | | |
|---|---|---|
| **Consensus** | **CACTGGAATGGATGCCATCTGCAGCCACCGTCTTGACCCCAAAAGCCCTGGACTCAACAGAGAGCAGCTG** | |
| NM_001414687.1 (35,819 .. 46,231) | CACTGGAATGGATGCCATCTGCAGCCACCGTCTTGACCCCAAAAGCCCTGGACTCAACAGAGAGCAGCTG | 3290 |
| Kuramochi | CACTGGAATGGATGCCATCTGCAGCCACCGTCTTGACCCCAAAAGCCCTGGACTCAACAGAGAGCAGCTG | 3290 |
| OVCAR3 | CACTGGAATGGATGCCATCTGCAGCCACCGTCTTGACCCCAAAAGCCCTGGACTCAACAGAGAGCAGCTG | 3290 |
| OVCAR5 | CACTGGAATGGATGCCATCTGCAGCCACCGTCTTGACCCCAAAAGCCCTGGACTCAACAGAAAGCAGCTG | 3290 |
| OV1 | CACTGGAATGGATGCCATCTGCAGCCACCGTCTTGACCCCAAAAGCCCTGGACTCAACAGAGAGCAGCTG | 3290 |
| OV2 | CACTGGAATGGATGCCATCTGCAGCCACCGTCTTGACCCCAAAAGCCCTGGACTCAACAGAGAGCAGCTG | 3290 |
| OV3 | CACTGGAATGGATGCCATCTGCAGCCACCGTCTTGACCCCAAAAGCCCTGGACTCAACAGAGAGCAGCTG | 3290 |

| | | |
|---|---|---|
| **Consensus** | **TACTGGGAGCTGAGCCAGCTGACCCATGGCATCAAAGAGCTGGGCCCCTACACCCTGGACAGGAACAGTC** | |
| NM_001414687.1 (35,819 .. 46,231) | TACTGGGAGCTGAGCCAGCTGACCCATGGCATCAAAGAGCTGGGCCCCTACACCCTGGACAGGAACAGTC | 3360 |
| Kuramochi | TACTGGGAGCTGAGCCAGCTGACCCATGGCATCAAAGAGCTGGGCCCCTACACCCTGGACAGGAACAGTC | 3360 |
| OVCAR3 | TACTGGGAGCTGAGCCAGCTGACCCATGGCATCAAAGAGCTGGGCCCCTACACCCTGGACAGGAACAGTC | 3360 |
| OVCAR5 | TACTGGGAGCTGAGCCAGCTGACCCATGGCATCAAAGAGCTGGGCCCCTACACCCTGGACAGGAACAGTC | 3360 |
| OV1 | TACTGGGAGCTGAGCCAGCTGACCCATGGCATCAAAGAGCTGGGCCCCTACACCCTGGACAGGAACAGTC | 3360 |
| OV2 | TACTGGGAGCTGAGCCAGCTGACCCATGGCATCAAAGAGCTGGGCCCCTACACCCTGGACAGGAACAGTC | 3360 |
| OV3 | TACTGGGAGCTGAGCCAGCTGACCCATGGCATCAAAGAGCTGGGCCCCTACACCCTGGACAGGAACAGTC | 3360 |

| | | |
|---|---|---|
| **Consensus** | **TCTATGTCAATGGTTTCACCCATCGGAGCTCTGTGGCCCCCACCAGCACTCCTGGGACCTCCACAGTGGA** | |
| NM_001414687.1 (35,819 .. 46,231) | TCTATGTCAATGGTTTCACCCATCGGAGCTCTGTGGCCCCCACCAGCACTCCTGGGACCTCCACAGTGGA | 3430 |
| Kuramochi | TCTATGTCAATGGTTTCACCCATCGGAGCTCTGTGGCCCCCACCAGCACTCCTGGGACCTCCACAGTGGA | 3430 |
| OVCAR3 | TCTATGTCAATGGTTTCACCCATCGGAGCTCTGTGGCCCCCACCAGCACTCCTGGGACCTCCACAGTGGA | 3430 |
| OVCAR5 | TCTATGTCAATGGTTTCACCCATCGGAGCTCTGTGGCCCCCACCAGCACTCCTGGGACCTCCACAGTGGA | 3430 |
| OV1 | TCTATGTCAATGGTTTCACCCATCGGAGCTCTGTGGCCCCCACCAGCACTCCTGGGACCTCCACAGTGGA | 3430 |
| OV2 | TCTATGTCAATGGTTTCACCCATCGGAGCTCTGTGGCCCCCACCAGCACTCCTGGGACCTCCACAGTGGA | 3430 |
| OV3 | TCTATGTCAATGGTTTCACCCATCGGAGCTCTGTGGCCCCCACCAGCACTCCTGGGACCTCCACAGTGGA | 3430 |

| | | |
|---|---|---|
| **Consensus** | **CCTTGGGACCTCAGGGACTCCATCCTCCCTCCCCAGCCCCACAACAGCTGTTCCTCTCCTGGTGCCGTTC** | |
| NM_001414687.1 (35,819 .. 46,231) | CCTTGGGACCTCAGGGACTCCATCCTCCCTCCCCAGCCCCACAACAGCTGTTCCTCTCCTGGTGCCGTTC | 3500 |
| Kuramochi | CCTTGGGACCTCAGGGACTCCATCCTCCCTCCCCAGCCCCACAACAGCTGTTCCTCTCCTGGTGCCGTTC | 3500 |
| OVCAR3 | CCTTGGGACCTCAGGGACTCCATCCTCCCTCCCCAGCCCCACAACAGCTGTTCCTCTCCTGGTGCCGTTC | 3500 |
| OVCAR5 | CCTTGGGACCTCAGGGACTCCATCCTCCCTCCCCAGCCCCACAACAGCTGTTCCTCTCCTGGTGCCGTTC | 3500 |
| OV1 | CCTTGGGACCTCAGGGACTCCATCCTCCCTCCCCAGCCCCACAACAGCTGTTCCTCTCCTGGTGCCGTTC | 3500 |
| OV2 | CCTTGGGACCTCAGGGACTCCATCCTCCCTCCCCAGCCCCACAACAGCTGTTCCTCTCCTGGTGCCGTTC | 3500 |
| OV3 | CCTTGGGACCTCAGGGACTCCATCCTCCCTCCCCAGCCCCACAACAGCTGTTCCTCTCCTGGTGCCGTTC | 3500 |

| | | |
|---|---|---|
| **Consensus** | **ACCCTCAACTTTACCATCACCAATCTGCAGTATGGGGAGGACATGCGTCACCCTGGCTCCAGGAAGTTCA** | |
| NM_001414687.1 (35,819 .. 46,231) | ACCCTCAACTTTACCATCACCAATCTGCAGTATGGGGAGGACATGCGTCACCCTGGCTCCAGGAAGTTCA | 3570 |
| Kuramochi | ACCCTCAACTTTACCATCACCAATCTGCAGTATGGGGAGGACATGCGTCACCCTGGCTCCAGGAAGTTCA | 3570 |
| OVCAR3 | ACCCTCAACTTTACCATCACCAATCTGCAGTATGGGGAGGACATGCGTCACCCTGGCTCCAGGAAGTTCA | 3570 |
| OVCAR5 | ACCCTCAACTTTACCATCACCAATCTGCAGTATGGGGAGGACATGCGTCACCCTGGCTCCAGGAAGTTCA | 3570 |
| OV1 | ACCCTCAACTTTACCATCACCAATCTGCAGTATGGGGAGGACATGCGTCACCCTGGCTCCAGGAAGTTCA | 3570 |
| OV2 | ACCCTCAACTTTACCATCACCAATCTGCAGTATGGGGAGGACATGCGTCACCCTGGCTCCAGGAAGTTCA | 3570 |
| OV3 | ACCCTCAACTTTACCATCACCAATCTGCAGTATGGGGAGGACATGCGTCACCCTGGCTCCAGGAAGTTCA | 3570 |

| | | |
|---|---|---|
| **Consensus** | **ACACCACAGAGAGGGTCCTGCAGGGTCTGCTTGGTCCCTTGTTCAAGAACTCCAGTGTCGGCCCTCTGTA** | |
| NM_001414687.1 (35,819 .. 46,231) | ACACCACAGAGAGGGTCCTGCAGGGTCTGCTTGGTCCCTTGTTCAAGAACTCCAGTGTCGGCCCTCTGTA | 3640 |
| Kuramochi | ACACCACAGAGAGGGTCCTGCAGGGTCTGCTTGGTCCCTTGTTCAAGAACTCCAGTGTCGGCCCTCTGTA | 3640 |
| OVCAR3 | ACACCACAGAGAGGGTCCTGCAGGGTCTGCTTGGTCCCTTGTTCAAGAACTCCAGTGTCGGCCCTCTGTA | 3640 |
| OVCAR5 | ACACCACAGAGAGGGTCCTGCAGGGTCTGCTTGGTCCCTTGTTCAAGAACTCCAGTGTCGGCCCTCTGTA | 3640 |
| OV1 | ACACCACAGAGAGGGTCCTGCAGGGTCTGCTTGGTCCCTTGTTCAAGAACTCCAGTGTCGGCCCTCTGTA | 3640 |
| OV2 | ACACCACAGAGAGGGTCCTGCAGGGTCTGCTTGGTCCCTTGTTCAAGAACTCCAGTGTCGGCCCTCTGTA | 3640 |
| OV3 | ACACCACAGAGAGGGTCCTGCAGGGTCTGCTTGGTCCCTTGTTCAAGAACTCCAGTGTCGGCCCTCTGTA | 3640 |

| | | |
|---|---|---|
| **Consensus** | **CTCTGGCTGCAGACTGATCTCTCTCAGGTCTGAGAAGGATGGGGCAGCCACTGGAGTGGATGCCATCTGC** | |
| NM_001414687.1 (35,819 .. 46,231) | CTCTGGCTGCAGACTGATCTCTCTCAGGTCTGAGAAGGATGGGGCAGCCACTGGAGTGGATGCCATCTGC | 3710 |
| Kuramochi | CTCTGGCTGCAGACTGATCTCTCTCAGGTCTGAGAAGGATGGGGCAGCCACTGGAGTGGATGCCATCTGC | 3710 |
| OVCAR3 | CTCTGGCTGCAGACTGATCTCTCTCAGGTCTGAGAAGGATGGGGCAGCCACTGGAGTGGATGCCATCTGC | 3710 |
| OVCAR5 | CTCTGGCTGCAGACTGATCTCTCTCAGGTCTGAGAAGGATGGGGCAGCCACTGGAGTGGATGCCATCTGC | 3710 |
| OV1 | CTCTGGCTGCAGACTGATCTCTCTCAGGTCTGAGAAGGATGGGGCAGCCACTGGAGTGGATGCCATCTGC | 3710 |
| OV2 | CTCTGGCTGCAGACTGATCTCTCTCAGGTCTGAGAAGGATGGGGCAGCCACTGGAGTGGATGCCATCTGC | 3710 |
| OV3 | CTCTGGCTGCAGACTGATCTCTCTCAGGTCTGAGAAGGATGGGGCAGCCACTGGAGTGGATGCCATCTGC | 3710 |

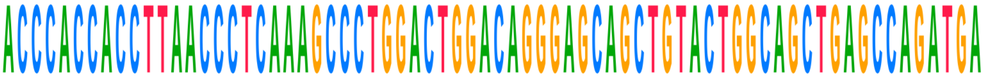

| | | |
|---|---|---|
| **Consensus** | **ACCCACCACCTTAACCCTCAAAGCCCTGGACTGGACAGGGAGCAGCTGTACTGGCAGCTGAGCCAGATGA** | |
| NM_001414687.1 (35,819 .. 46,231) | ACCCACCACCTTAACCCTCAAAGCCCTGGACTGGACAGGGAGCAGCTGTACTGGCAGCTGAGCCAGATGA | 3780 |
| Kuramochi | ACCCACCACCTTAACCCTCAAAGCCCTGGACTGGACAGGGAGCAGCTGTACTGGCAGCTGAGCCAGATGA | 3780 |
| OVCAR3 | ACCCACCACCTTAACCCTCAAAGCCCTGGACTGGACAGGGAGCAGCTGTACTGGCAGCTGAGCCAGATGA | 3780 |
| OVCAR5 | ACCCACCACCTTAACCCTCAAAGCCCTGGACTGGACAGGGAGCAGCTGTACTGGCAGCTGAGCCAGATGA | 3780 |
| OV1 | ACCCACCACCTTAACCCTCAAAGCCCTGGACTGGACAGGGAGCAGCTGTACTGGCAGCTGAGCCAGATGA | 3780 |
| OV2 | ACCCACCACCTTAACCCTCAAAGCCCTGGACTGGACAGGGAGCAGCTGTACTGGCAGCTGAGCCAGATGA | 3780 |
| OV3 | ACCCACCACCTTAACCCTCAAAGCCCTGGACTGGACAGGGAGCAGCTGTACTGGCAGCTGAGCCAGATGA | 3780 |

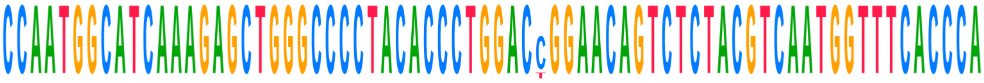

| | | |
|---|---|---|
| **Consensus** | **CCAATGGCATCAAAGAGCTGGGCCCCTACACCCTGGACCGGAACAGTCTCTACGTCAATGGTTTCACCCA** | |
| NM_001414687.1 (35,819 .. 46,231) | CCAATGGCATCAAAGAGCTGGGCCCCTACACCCTGGACCGGAACAGTCTCTACGTCAATGGTTTCACCCA | 3850 |
| Kuramochi | CCAATGGCATCAAAGAGCTGGGCCCCTACACCCTGGACCGGAACAGTCTCTACGTCAATGGTTTCACCCA | 3850 |
| OVCAR3 | CCAATGGCATCAAAGAGCTGGGCCCCTACACCCTGGACTGGAACAGTCTCTACGTCAATGGTTTCACCCA | 3850 |
| OVCAR5 | CCAATGGCATCAAAGAGCTGGGCCCCTACACCCTGGACCGGAACAGTCTCTACGTCAATGGTTTCACCCA | 3850 |
| OV1 | CCAATGGCATCAAAGAGCTGGGCCCCTACACCCTGGACCGGAACAGTCTCTACGTCAATGGTTTCACCCA | 3850 |
| OV2 | CCAATGGCATCAAAGAGCTGGGCCCCTACACCCTGGACCGGAACAGTCTCTACGTCAATGGTTTCACCCA | 3850 |
| OV3 | CCAATGGCATCAAAGAGCTGGGCCCCTACACCCTGGACCGGAACAGTCTCTACGTCAATGGTTTCACCCA | 3850 |

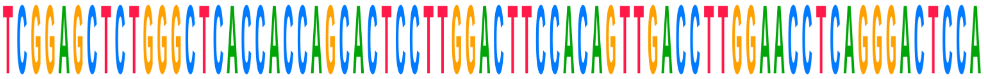

| | | |
|---|---|---|
| **Consensus** | **TCGGAGCTCTGGGCTCACCACCAGCACTCCTTGGACTTCCACAGTTGACCTTGGAACCTCAGGGACTCCA** | |
| NM_001414687.1 (35,819 .. 46,231) | TCGGAGCTCTGGGCTCACCACCAGCACTCCTTGGACTTCCACAGTTGACCTTGGAACCTCAGGGACTCCA | 3920 |
| Kuramochi | TCGGAGCTCTGGGCTCACCACCAGCACTCCTTGGACTTCCACAGTTGACCTTGGAACCTCAGGGACTCCA | 3920 |
| OVCAR3 | TCGGAGCTCTGGGCTCACCACCAGCACTCCTTGGACTTCCACAGTTGACCTTGGAACCTCAGGGACTCCA | 3920 |
| OVCAR5 | TCGGAGCTCTGGGCTCACCACCAGCACTCCTTGGACTTCCACAGTTGACCTTGGAACCTCAGGGACTCCA | 3920 |
| OV1 | TCGGAGCTCTGGGCTCACCACCAGCACTCCTTGGACTTCCACAGTTGACCTTGGAACCTCAGGGACTCCA | 3920 |
| OV2 | TCGGAGCTCTGGGCTCACCACCAGCACTCCTTGGACTTCCACAGTTGACCTTGGAACCTCAGGGACTCCA | 3920 |
| OV3 | TCGGAGCTCTGGGCTCACCACCAGCACTCCTTGGACTTCCACAGTTGACCTTGGAACCTCAGGGACTCCA | 3920 |

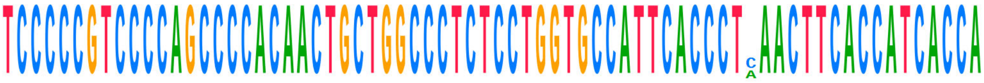

| | | |
|---|---|---|
| **Consensus** | **TCCCCGTCCCCAGCCCCACAACTGCTGGCCCTCTCCTGGTGCCATTCACCCTCAACTTCACCATCACCA** | |
| NM_001414687.1 (35,819 .. 46,231) | TCCCCGTCCCCAGCCCCACAACTGCTGGCCCTCTCCTGGTGCCATTCACCCTAAACTTCACCATCACCA | 3990 |
| Kuramochi | TCCCCGTCCCCAGCCCCACAACTGCTGGCCCTCTCCTGGTGCCATTCACCCTAAACTTCACCATCACCA | 3990 |
| OVCAR3 | TCCCCGTCCCCAGCCCCACAACTGCTGGCCCTCTCCTGGTGCCATTCACCCTAAACTTCACCATCACCA | 3990 |
| OVCAR5 | TCCCCGTCCCCAGCCCCACAACTGCTGGCCCTCTCCTGGTGCCATTCACCCTCAACTTCACCATCACCA | 3990 |
| OV1 | TCCCCGTCCCCAGCCCCACAACTGCTGGCCCTCTCCTGGTGCCATTCACCCTCAACTTCACCATCACCA | 3990 |
| OV2 | TCCCCGTCCCCAGCCCCACAACTGCTGGCCCTCTCCTGGTGCCATTCACCCTCAACTTCACCATCACCA | 3990 |
| OV3 | TCCCCGTCCCCAGCCCCACAACTGCTGGCCCTCTCCTGGTGCCATTCACCCTCAACTTCACCATCACCA | 3990 |

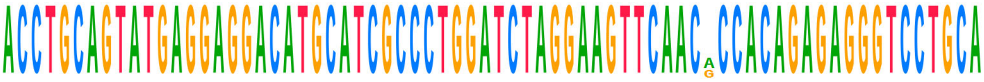

| | | |
|---|---|---|
| **Consensus** | **ACCTGCAGTATGAGGAGGACATGCATCGCCCTGGATCTAGGAAGTTCAACACCACAGAGAGGGTCCTGCA** | |
| NM_001414687.1 (35,819 .. 46,231) | ACCTGCAGTATGAGGAGGACATGCATCGCCCTGGATCTAGGAAGTTCAACGCCACAGAGAGGGTCCTGCA | 4060 |
| Kuramochi | ACCTGCAGTATGAGGAGGACATGCATCGCCCTGGATCTAGGAAGTTCAACGCCACAGAGAGGGTCCTGCA | 4060 |
| OVCAR3 | ACCTGCAGTATGAGGAGGACATGCATCGCCCTGGATCTAGGAAGTTCAACGCCACAGAGAGGGTCCTGCA | 4060 |
| OVCAR5 | ACCTGCAGTATGAGGAGGACATGCATCGCCCTGGATCTAGGAAGTTCAACACCACAGAGAGGGTCCTGCA | 4060 |
| OV1 | ACCTGCAGTATGAGGAGGACATGCATCGCCCTGGATCTAGGAAGTTCAACACCACAGAGAGGGTCCTGCA | 4060 |
| OV2 | ACCTGCAGTATGAGGAGGACATGCATCGCCCTGGATCTAGGAAGTTCAACACCACAGAGAGGGTCCTGCA | 4060 |
| OV3 | ACCTGCAGTATGAGGAGGACATGCATCGCCCTGGATCTAGGAAGTTCAACACCACAGAGAGGGTCCTGCA | 4060 |

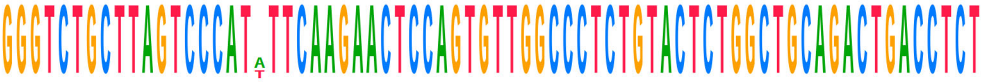

| | | |
|---|---|---|
| **Consensus** | **GGGTCTGCTTAGTCCCATATTCAAGAACTCCAGTGTTGGCCCTCTGTACTCTGGCTGCAGACTGACCTCT** | |
| NM_001414687.1 (35,819 .. 46,231) | GGGTCTGCTTAGTCCCATATTCAAGAACTCCAGTGTTGGCCCTCTGTACTCTGGCTGCAGACTGACCTCT | 4130 |
| Kuramochi | GGGTCTGCTTAGTCCCATATTCAAGAACTCCAGTGTTGGCCCTCTGTACTCTGGCTGCAGACTGACCTCT | 4130 |
| OVCAR3 | GGGTCTGCTTAGTCCCATATTCAAGAACTCCAGTGTTGGCCCTCTGTACTCTGGCTGCAGACTGACCTCT | 4130 |
| OVCAR5 | GGGTCTGCTTAGTCCCATTTTCAAGAACTCCAGTGTTGGCCCTCTGTACTCTGGCTGCAGACTGACCTCT | 4130 |
| OV1 | GGGTCTGCTTAGTCCCATATTCAAGAACTCCAGTGTTGGCCCTCTGTACTCTGGCTGCAGACTGACCTCT | 4130 |
| OV2 | GGGTCTGCTTAGTCCCATTTTCAAGAACTCCAGTGTTGGCCCTCTGTACTCTGGCTGCAGACTGACCTCT | 4130 |
| OV3 | GGGTCTGCTTAGTCCCATTTTCAAGAACTCCAGTGTTGGCCCTCTGTACTCTGGCTGCAGACTGACCTCT | 4130 |

```
Consensus                        CTCAGGCCCGAGAAGGATGGGGCAGCAACTGGAATGGATGCTGTCTGCCTCTACCACCCTAATCCCAAAA
NM_001414687.1 (35,819 .. 46,231) CTCAGGCCCGAGAAGGATGGGGCAGCAACTGGAATGGATGCTGTCTGCCTCTACCACCCTAATCCCAAAA 4200
Kuramochi                        CTCAGGCCCGAGAAGGATGGGGCAGCAACTGGAATGGATGCTGTCTGCCTCTACCACCCTAATCCCAAAA 4200
OVCAR3                           CTCAGGCCCGAGAAGGATGGGGCAGCAACTGGAATGGATGCTGTCTGCCTCTACCACCCTAATCCCAAAA 4200
OVCAR5                           CTCAGGCCCGAGAAGGATGGGGCAGCAACTGGAATGGATGCTGTCTGCCTCTACCACCCTAATCCCAAAA 4200
OV1                              CTCAGGCCCGAGAAGGATGGGGCAGCAACTGGAATGGATGCTGTCTGCCTCTACCACCCTAATCCCAAAA 4200
OV2                              CTCAGGCCCGAGAAGGATGGGGCAGCAACTGGAATGGATGCTGTCTGCCTCTACCACCCTAATCCCAAAA 4200
OV3                              CTCAGGCCCGAGAAGGATGGGGCAGCAACTGGAATGGATGCTGTCTGCCTCTACCACCCTAATCCCAAAA 4200
```

```
Consensus                        GACCTGGGCTGGACAGAGAGCAGCTGTACTGGGAGCTAAGCCAGCTGACCCACAACATCACTGAGCTGGG
NM_001414687.1 (35,819 .. 46,231) GACCTGGGCTGGACAGAGAGCAGCTGTACTGGGAGCTAAGCCAGCTGACCCACAACATCACTGAGCTGGG 4270
Kuramochi                        GACCTGGGCTGGACAGAGAGCAGCTGTACTGGGAGCTAAGCCAGCTGACCCACAACATCACTGAGCTGGG 4270
OVCAR3                           GACCTGGGCTGGACAGAGAGCAGCTGTACTGGGAGCTAAGCCAGCTGACCCACAACATCACTGAGCTGGG 4270
OVCAR5                           GACCTGGGCTGGACAGAGAGCAGCTGTACTGCGAGCTAAGCCAGCTGACCCACAACATCACTGAGCTGGG 4270
OV1                              GACCTGGGCTGGACAGAGAGCAGCTGTACTGGGAGCTAAGCCAGCTGACCCACAACATCACTGAGCTGGG 4270
OV2                              GACCTGGGCTGGACAGAGAGCAGCTGTACTGCGAGCTAAGCCAGCTGACCCACAACATCACTGAGCTGGG 4270
OV3                              GACCTGGGCTGGACAGAGAGCAGCTGTACTGCGAGCTAAGCCAGCTGACCCACAACATCACTGAGCTGGG 4270
```

```
Consensus                        CCCCTACAGCCTGGACAGGGACAGTCTCTATGTCAATGGTTTCACCCATCAGAACTCTGTGCCCACCACC
NM_001414687.1 (35,819 .. 46,231) CCCCTACAGCCTGGACAGGGACAGTCTCTATGTCAATGGTTTCACCCATCAGAACTCTGTGCCCACCACC 4340
Kuramochi                        CCCCTACAGCCTGGACAGGGACAGTCTCTATGTCAATGGTTTCACCCATCAGAACTCTGTGCCCACCACC 4340
OVCAR3                           CCCCTACAGCCTGGACAGGGACAGTCTCTATGTCAATGGTTTCACCCATCAGAACTCTGTGCCCACCACC 4340
OVCAR5                           CCCCTACAGCCTGGACAGGGACAGTCTCTATGTCAATGGTTTCACCCATCAGAACTCTGTGCCCACCACC 4340
OV1                              CCCCTACAGCCTGGACAGGGACAGTCTCTATGTCAATGGTTTCACCCATCAGAACTCTGTGCCCACCACC 4340
OV2                              CCCCTACAGCCTGGACAGGGACAGTCTCTATGTCAATGGTTTCACCCATCAGAACTCTGTGCCCACCACC 4340
OV3                              CCCCTACAGCCTGGACAGGGACAGTCTCTATGTCAATGGTTTCACCCATCAGAACTCTGTGCCCACCACC 4340
```

```
Consensus                        AGTACTCCTGGGACCTCCACAGTGTACTGGGCAACCACTGGGACTCCATCCTCCTTCCCCGGCCACACAG
NM_001414687.1 (35,819 .. 46,231) AGTACTCCTGGGACCTCCACAGTGTACTGGGCAACCACTGGGACTCCATCCTCCTTCCCCGGCCACACAG 4410
Kuramochi                        AGTACTCCTGGGACCTCCACAGTGTACTGGGCAACCACTGGGACTCCATCCTCCTTCCCCGGCCACACAG 4410
OVCAR3                           AGTACTCCTGGGACCTCCACAGTGTACTGGGCAACCACTGGGACTCCATCCTCCTTCCCCGGCCACACAG 4410
OVCAR5                           AGTACTCCTGGGACCTCCACAGTGTACTGGGCAACCACTGGGACTCCATCCTCCTTCCCCGGCCACACAG 4410
OV1                              AGTACTCCTGGGACCTCCACAGTGTACTGGGCAACCACTGGGACTCCATCCTCCTTCCCCGGCCACACAG 4410
OV2                              AGTACTCCTGGGACCTCCACAGTGTACTGGGCAACCACTGGGACTCCATCCTCCTTCCCCGGCCACACAG 4410
OV3                              AGTACTCCTGGGACCTCCACAGTGTACTGGGCAACCACTGGGACTCCATCCTCCTTCCCCGGCCACACAG 4410
```

```
Consensus                        AGCCTGGCCCTCTCCTGATACCATTCACTTTCAACTTTACCATCACCAACCTGCATTATGAGGAAAACAT
NM_001414687.1 (35,819 .. 46,231) AGCCTGGCCCTCTCCTGATACCATTCACTTTCAACTTTACCATCACCAACCTGCATTATGAGGAAAACAT 4480
Kuramochi                        AGCCTGGCCCTCTCCTGATACCATTCACTTTCAACTTTACCATCACCAACCTGCATTATGAGGAAAACAT 4480
OVCAR3                           AGCCTGGCCCTCTCCTGATACCATTCACTTTCAACTTTACCATCACCAACCTGCATTATGAGGAAAACAT 4480
OVCAR5                           AGCCTGGCCCTCTCCTGATACCATTCACTTTCAACTTTACCATCACCAACCTGCATTATGAGGAAAACAT 4480
OV1                              AGCCTGGCCCTCTCCTGATACCATTCACTTTCAACTTTACCATCACCAACCTGCATTATGAGGAAAACAT 4480
OV2                              AGCCTGGCCCTCTCCTGATACCATTCACTTTCAACTTTACCATCACCAACCTGCATTATGAGGAAAACAT 4480
OV3                              AGCCTGGCCCTCTCCTGATACCATTCACTTTCAACTTTACCATCACCAACCTGCATTATGAGGAAAACAT 4480
```

```
Consensus                        GCAACACCCTGGTTCCAGGAAGTTCAACACCACGGAGAGGGTTCTGCAGGGTCTGCTCACGCCCTTGTTC
NM_001414687.1 (35,819 .. 46,231) GCAACACCCTGGTTCCAGGAAGTTCAACACCACGGAGAGGGTTCTGCAGGGTCTGCTCACGCCCTTGTTC 4550
Kuramochi                        GCAACACCCTGGTTCCAGGAAGTTCAACACCACGGAGAGGGTTCTGCAGGGTCTGCTCACGCCCTTGTTC 4550
OVCAR3                           GCAACACCCTGGTTCCAGGAAGTTCAACACCACGGAGAGGGTTCTGCAGGGTCTGCTCACGCCCTTGTTC 4550
OVCAR5                           GCAACACCCTGGTTCCAGGAAGTTCAACACCACGGAGAGGGTTCTGCAGGGTCTGCTCAAGCCCTTGTTC 4550
OV1                              GCAACACCCTGGTTCCAGGAAGTTCAACACCACGGAGAGGGTTCTGCAGGGTCTGCTCACGCCCTTGTTC 4550
OV2                              GCAACACCCTGGTTCCAGGAAGTTCAACACCACGGAGAGGGTTCTGCAGGGTCTGCTCAAGCCCTTGTTC 4550
OV3                              GCAACACCCTGGTTCCAGGAAGTTCAACACCACGGAGAGGGTTCTGCAGGGTCTGCTCAAGCCCTTGTTC 4550
```

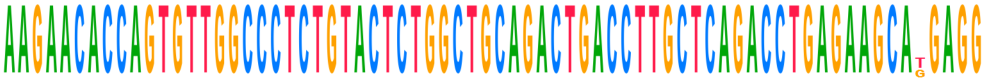

| | | |
|---|---|---|
| **Consensus** | **AAGAACACCAGTGTTGGCCCTCTGTACTCTGGCTGCAGACTGACCTTGCTCAGACCTGAGAAGCATGAGG** | |
| NM_001414687.1 (35,819 .. 46,231) | AAGAACACCAGTGTTGGCCCTCTGTACTCTGGCTGCAGACTGACCTTGCTCAGACCTGAGAAGCAGGAGG | 4620 |
| Kuramochi | AAGAACACCAGTGTTGGCCCTCTGTACTCTGGCTGCAGACTGACCTTGCTCAGACCTGAGAAGCAGGAGG | 4620 |
| OVCAR3 | AAGAACACCAGTGTTGGCCCTCTGTACTCTGGCTGCAGACTGACCTTGCTCAGACCTGAGAAGCAGGAGG | 4620 |
| OVCAR5 | AAGAACACCAGTGTTGGCCCTCTGTACTCTGGCTGCAGACTGACCTTGCTCAGACCTGAGAAGCATGAGG | 4620 |
| OV1 | AAGAACACCAGTGTTGGCCCTCTGTACTCTGGCTGCAGACTGACCTTGCTCAGACCTGAGAAGCATGAGG | 4620 |
| OV2 | AAGAACACCAGTGTTGGCCCTCTGTACTCTGGCTGCAGACTGACCTTGCTCAGACCTGAGAAGCATGAGG | 4620 |
| OV3 | AAGAACACCAGTGTTGGCCCTCTGTACTCTGGCTGCAGACTGACCTTGCTCAGACCTGAGAAGCATGAGG | 4620 |

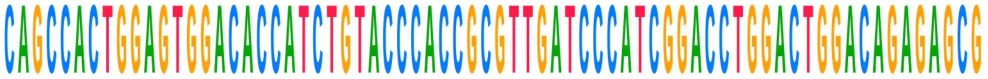

| | | |
|---|---|---|
| **Consensus** | **CAGCCACTGGAGTGGACACCATCTGTACCCACCGCGTTGATCCCATCGGACCTGGACTGGACAGAGAGCG** | |
| NM_001414687.1 (35,819 .. 46,231) | CAGCCACTGGAGTGGACACCATCTGTACCCACCGCGTTGATCCCATCGGACCTGGACTGGACAGAGAGCG | 4690 |
| Kuramochi | CAGCCACTGGAGTGGACACCATCTGTACCCACCGCGTTGATCCCATCGGACCTGGACTGGACAGAGAGCG | 4690 |
| OVCAR3 | CAGCCACTGGAGTGGACACCATCTGTACCCACCGCGTTGATCCCATCGGACCTGGACTGGACAGAGAGCG | 4690 |
| OVCAR5 | CAGCCACTGGAGTGGACACCATCTGTACCCACCGCGTTGATCCCATCGGACCTGGACTGGACAGAGAGCG | 4690 |
| OV1 | CAGCCACTGGAGTGGACACCATCTGTACCCACCGCGTTGATCCCATCGGACCTGGACTGGACAGAGAGCG | 4690 |
| OV2 | CAGCCACTGGAGTGGACACCATCTGTACCCACCGCGTTGATCCCATCGGACCTGGACTGGACAGAGAGCG | 4690 |
| OV3 | CAGCCACTGGAGTGGACACCATCTGTACCCACCGCGTTGATCCCATCGGACCTGGACTGGACAGAGAGCG | 4690 |

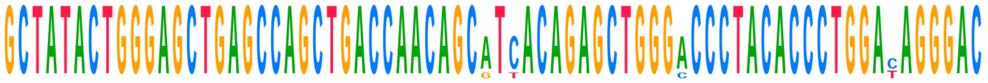

| | | |
|---|---|---|
| **Consensus** | **GCTATACTGGGAGCTGAGCCAGCTGACCAACAGCATCACAGAGCTGGGACCCTACACCCTGGACAGGGAC** | |
| NM_001414687.1 (35,819 .. 46,231) | GCTATACTGGGAGCTGAGCCAGCTGACCAACAGCGTTACAGAGCTGGGCCCCTACACCCTGGACAGGGAC | 4760 |
| Kuramochi | GCTATACTGGGAGCTGAGCCAGCTGACCAACAGCATCACAGAGCTGGGACCCTACACCCTGGATAGGGAC | 4760 |
| OVCAR3 | GCTATACTGGGAGCTGAGCCAGCTGACCAACAGCATCACAGAGCTGGGACCCTACACCCTGGATAGGGAC | 4760 |
| OVCAR5 | GCTATACTGGGAGCTGAGCCAGCTGACCAACAGCATCACAGAGCTGGGACCCTACACCCTGGACAGGGAC | 4760 |
| OV1 | GCTATACTGGGAGCTGAGCCAGCTGACCAACAGCATCACAGAGCTGGGACCCTACACCCTGGATAGGGAC | 4760 |
| OV2 | GCTATACTGGGAGCTGAGCCAGCTGACCAACAGCATCACAGAGCTGGGACCCTACACCCTGGACAGGGAC | 4760 |
| OV3 | GCTATACTGGGAGCTGAGCCAGCTGACCAACAGCATCACAGAGCTGGGACCCTACACCCTGGACAGGGAC | 4760 |

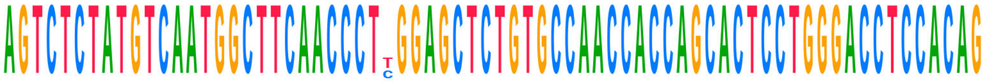

| | | |
|---|---|---|
| **Consensus** | **AGTCTCTATGTCAATGGCTTCAACCCTTGGAGCTCTGTGCCAACCACCAGCACTCCTGGGACCTCCACAG** | |
| NM_001414687.1 (35,819 .. 46,231) | AGTCTCTATGTCAATGGCTTCAACCCTTGGAGCTCTGTGCCAACCACCAGCACTCCTGGGACCTCCACAG | 4830 |
| Kuramochi | AGTCTCTATGTCAATGGCTTCAACCCTTGGAGCTCTGTGCCAACCACCAGCACTCCTGGGACCTCCACAG | 4830 |
| OVCAR3 | AGTCTCTATGTCAATGGCTTCAACCCTTGGAGCTCTGTGCCAACCACCAGCACTCCTGGGACCTCCACAG | 4830 |
| OVCAR5 | AGTCTCTATGTCAATGGCTTCAACCCTCGGAGCTCTGTGCCAACCACCAGCACTCCTGGGACCTCCACAG | 4830 |
| OV1 | AGTCTCTATGTCAATGGCTTCAACCCTTGGAGCTCTGTGCCAACCACCAGCACTCCTGGGACCTCCACAG | 4830 |
| OV2 | AGTCTCTATGTCAATGGCTTCAACCCTCGGAGCTCTGTGCCAACCACCAGCACTCCTGGGACCTCCACAG | 4830 |
| OV3 | AGTCTCTATGTCAATGGCTTCAACCCTCGGAGCTCTGTGCCAACCACCAGCACTCCTGGGACCTCCACAG | 4830 |

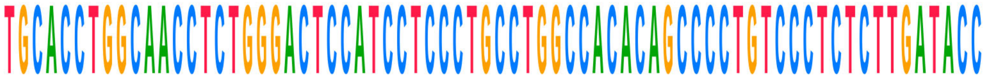

| | | |
|---|---|---|
| **Consensus** | **TGCACCTGGCAACCTCTGGGACTCCATCCTCCCTGCCTGGCCACACAGCCCCTGTCCCTCTCTTGATACC** | |
| NM_001414687.1 (35,819 .. 46,231) | TGCACCTGGCAACCTCTGGGACTCCATCCTCCCTGCCTGGCCACACAGCCCCTGTCCCTCTCTTGATACC | 4900 |
| Kuramochi | TGCACCTGGCAACCTCTGGGACTCCATCCTCCCTGCCTGGCCACACAGCCCCTGTCCCTCTCTTGATACC | 4900 |
| OVCAR3 | TGCACCTGGCAACCTCTGGGACTCCATCCTCCCTGCCTGGCCACACAGCCCCTGTCCCTCTCTTGATACC | 4900 |
| OVCAR5 | TGCACCTGGCAACCTCTGGGACTCCATCCTCCCTGCCTGGCCACACAGCCCCTGTCCCTCTCTTGATACC | 4900 |
| OV1 | TGCACCTGGCAACCTCTGGGACTCCATCCTCCCTGCCTGGCCACACAGCCCCTGTCCCTCTCTTGATACC | 4900 |
| OV2 | TGCACCTGGCAACCTCTGGGACTCCATCCTCCCTGCCTGGCCACACAGCCCCTGTCCCTCTCTTGATACC | 4900 |
| OV3 | TGCACCTGGCAACCTCTGGGACTCCATCCTCCCTGCCTGGCCACACAGCCCCTGTCCCTCTCTTGATACC | 4900 |

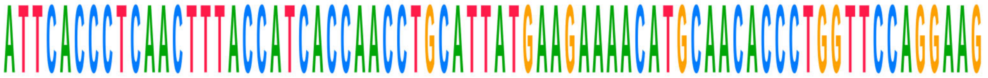

| | | |
|---|---|---|
| **Consensus** | **ATTCACCCTCAACTTTACCATCACCAACCTGCATTATGAAGAAAACATGCAACACCCTGGTTCCAGGAAG** | |
| NM_001414687.1 (35,819 .. 46,231) | ATTCACCCTCAACTTTACCATCACCAACCTGCATTATGAAGAAAACATGCAACACCCTGGTTCCAGGAAG | 4970 |
| Kuramochi | ATTCACCCTCAACTTTACCATCACCAACCTGCATTATGAAGAAAACATGCAACACCCTGGTTCCAGGAAG | 4970 |
| OVCAR3 | ATTCACCCTCAACTTTACCATCACCAACCTGCATTATGAAGAAAACATGCAACACCCTGGTTCCAGGAAG | 4970 |
| OVCAR5 | ATTCACCCTCAACTTTACCATCACCAACCTGCATTATGAAGAAAACATGCAACACCCTGGTTCCAGGAAG | 4970 |
| OV1 | ATTCACCCTCAACTTTACCATCACCAACCTGCATTATGAAGAAAACATGCAACACCCTGGTTCCAGGAAG | 4970 |
| OV2 | ATTCACCCTCAACTTTACCATCACCAACCTGCATTATGAAGAAAACATGCAACACCCTGGTTCCAGGAAG | 4970 |
| OV3 | ATTCACCCTCAACTTTACCATCACCAACCTGCATTATGAAGAAAACATGCAACACCCTGGTTCCAGGAAG | 4970 |

| | | |
|---|---|---|
| **Consensus** | **TTCAACACCACGGAGAGGGTTCTGCAGGGTCTGCTCAAGCCCTTGTTCAAGAGCACCAGTGTTGGCCCTC** | |
| NM_001414687.1 (35,819 .. 46,231) | TTCAACACCACGGAGAGGGTTCTGCAGGGTCTGCTCAAGCCCTTGTTCAAGAGCACCAGCGTTGGCCCTC | 5040 |
| Kuramochi | TTCAACACCACGGAGAGGGTTCTGCAGGGTCTGCTCAAGCCCTTGTTCAAGAGCACCAGCGTTGGCCCTC | 5040 |
| OVCAR3 | TTCAACACCACGGAGAGGGTTCTGCAGGGTCTGCTCAAGCCCTTGTTCAAGAGCACCAGCGTTGGCCCTC | 5040 |
| OVCAR5 | TTCAACACCACGGAGAGGGTTCTGCAGGGTCTGCTCAAGCCCTTGTTCAAGAGCACCAGTGTTGGCCCTC | 5040 |
| OV1 | TTCAACACCACGGAGAGGGTTCTGCAGGGTCTGCTCAAGCCCTTGTTCAAGAGCACCAGTGTTGGCCCTC | 5040 |
| OV2 | TTCAACACCACGGAGAGGGTTCTGCAGGGTCTGCTCAAGCCCTTGTTCAAGAGCACCAGTGTTGGCCCTC | 5040 |
| OV3 | TTCAACACCACGGAGAGGGTTCTGCAGGGTCTGCTCAAGCCCTTGTTCAAGAGCACCAGTGTTGGCCCTC | 5040 |

| | | |
|---|---|---|
| **Consensus** | **TGTACTCTGGCTGCAGACTGACCTTGCTCAGACCTGAGAAACATGGGGCAGCCACTGGAGTGGACGCCAT** | |
| NM_001414687.1 (35,819 .. 46,231) | TGTACTCTGGCTGCAGACTGACCTTGCTCAGACCTGAGAAACATGGGGCAGCCACTGGAGTGGACGCCAT | 5110 |
| Kuramochi | TGTACTCTGGCTGCAGACTGACCTTGCTCAGACCTGAGAAACATGGGGCAGCCACTGGAGTGGACGCCAT | 5110 |
| OVCAR3 | TGTACTCTGGCTGCAGACTGACCTTGCTCAGACCTGAGAAACATGGGGCAGCCACTGGAGTGGACGCCAT | 5110 |
| OVCAR5 | TGTACTCTGGCTGCAGACTGACCTTGCTCAGACCTGAGAAACATGGGGCAGCCACTGGAGTGGACGCCAT | 5110 |
| OV1 | TGTACTCTGGCTGCAGACTGACCTTGCTCAGACCTGAGAAACATGGGGCAGCCACTGGAGTGGACGCCAT | 5110 |
| OV2 | TGTACTCTGGCTGCAGACTGACCTTGCTCAGACCTGAGAAACATGGGGCAGCCACTGGAGTGGACGCCAT | 5110 |
| OV3 | TGTACTCTGGCTGCAGACTGACCTTGCTCAGACCTGAGAAACATGGGGCAGCCACTGGAGTGGACGCCAT | 5110 |

| | | |
|---|---|---|
| **Consensus** | **CTGCACCCTCCGCCTTGATCCCACTGGTCCTGGACTGGACAGAGAGCGGCTATACTGGGAGCTGAGCCAG** | |
| NM_001414687.1 (35,819 .. 46,231) | CTGCACCCTCCGCCTTGATCCCACTGGTCCTGGACTGGACAGAGAGCGGCTATACTGGGAGCTGAGCCAG | 5180 |
| Kuramochi | CTGCACCCTCCGCCTTGATCCCACTGGTCCTGGACTGGACAGAGAGCGGCTATACTGGGAGCTGAGCCAG | 5180 |
| OVCAR3 | CTGCACCCTCCGCCTTGATCCCACTGGTCCTGGACTGGACAGAGAGCGGCTATACTGGGAGCTGAGCCAG | 5180 |
| OVCAR5 | CTGCACCCTCCGCCTTGATCCCACTGGTCCTGGACTGGACAGAGAGCGGCTATACTGGGAGCTGAGCCAG | 5180 |
| OV1 | CTGCACCCTCCGCCTTGATCCCACTGGTCCTGGACTGGACAGAGAGCGGCTATACTGGGAGCTGAGCCAG | 5180 |
| OV2 | CTGCACCCTCCGCCTTGATCCCACTGGTCCTGGACTGGACAGAGAGCGGCTATACTGGGAGCTGAGCCAG | 5180 |
| OV3 | CTGCACCCTCCGCCTTGATCCCACTGGTCCTGGACTGGACAGAGAGTGGCTATACTGGGAGCTGAGCCAG | 5180 |

| | | |
|---|---|---|
| **Consensus** | **CTGACCAACAGCGTTACAGAGCTGGGCCCCTACACCCTGGACAGGGACAGTCTCTATGTCAATGGCTTCA** | |
| NM_001414687.1 (35,819 .. 46,231) | CTGACCAACAGCGTTACAGAGCTGGGCCCCTACACCCTGGACAGGGACAGTCTCTATGTCAATGGCTTCA | 5250 |
| Kuramochi | CTGACCAACAGCGTTACAGAGCTGGGCCCCTACACCCTGGACAGGGACAGTCTCTATGTCAATGGCTTCA | 5250 |
| OVCAR3 | CTGACCAACAGCGTTACAGAGCTGGGCCCCTACACCCTGGACAGGGACAGTCTCTATGTCAATGGCTTCA | 5250 |
| OVCAR5 | CTGACCAACAGCGTTACAGAGCTGGGCCCCTACACCCTGGACAGGGACAGTCTCTATGTCAATGGCTTCA | 5250 |
| OV1 | CTGACCAACAGCGTTACAGAGCTGGGCCCCTACACCCTGGACAGGGACAGTCTCTATGTCAATGGCTTCA | 5250 |
| OV2 | CTGACCAACAGCGTTACAGAGCTGGGCCCCTACACCCTGGACAGGGACAGTCTCTATGTCAATGGCTTCA | 5250 |
| OV3 | CTGACCAACAGCGTTACAGAGCTGGGCCCCTACACCCTGGACAGGGACAGTCTCTATGTCAATGGCTTCA | 5250 |

| | | |
|---|---|---|
| **Consensus** | **CCCATCGGAGCTCTGTGCCAACCACCAGTATTCCTGGGACCTCTGCAGTGCACCTGGAAACCTCTGGGAC** | |
| NM_001414687.1 (35,819 .. 46,231) | CCCATCGGAGCTCTGTGCCAACCACCAGTATTCCTGGGACCTCTGCAGTGCACCTGGAAACCTCTGGGAC | 5320 |
| Kuramochi | CCCATCGGAGCTCTGTGCCAACCACCAGTATTCCTGGGACCTCTGCAGTGCACCTGGAAACCTCTGGGAC | 5320 |
| OVCAR3 | CCCATCGGAGCTCTGTGCCAACCACCAGTATTCCTGGGACCTCTGCAGTGCACCTGGAAACCTCTGGGAC | 5320 |
| OVCAR5 | CCCATCGGAGCTCTGTGCCAACCACCAGTATTCCTGGGACCTCTGCAGTGCACCTGGAAACCTCTGGGAC | 5320 |
| OV1 | CCCATCGGAGCTCTGTGCCAACCACCAGTATTCCTGGGACCTCTGCAGTGCACCTGGAAACCTCTGGGAC | 5320 |
| OV2 | CCCATCGGAGCTCTGTGCCAACCACCAGTATTCCTGGGACCTCTGCAGTGCACCTGGAAACCTCTGGGAC | 5320 |
| OV3 | CCCATCGGAGCTCTGTGCCAACCACCAGTATTCCTGGGACCTCTGCAGTGCACCTGGAAACCTCTGGGAC | 5320 |

| | | |
|---|---|---|
| **Consensus** | **TCCAGCCTCCCTCCCTGGCCACACAGCCCCTGGCCCTCTCCTGGTGCCATTCACCCTCAACTTCACTATC** | |
| NM_001414687.1 (35,819 .. 46,231) | TCCAGCCTCCCTCCCTGGCCACACAGCCCCTGGCCCTCTCCTGGTGCCATTCACCCTCAACTTCACTATC | 5390 |
| Kuramochi | TCCAGCCTCCCTCCCTGGCCACACAGCCCCTGGCCCTCTCCTGGTGCCATTCACCCTCAACTTCACTATC | 5390 |
| OVCAR3 | TCCAGCCTCCCTCCCTGGCCACACAGCCCCTGGCCCTCTCCTGGTGCCATTCACCCTCAACTTCACTATC | 5390 |
| OVCAR5 | TCCAGCCTCCCTCCCTGGCCACACAGCCCCTGGCCCTCTCCTGGTGCCATTCACCCTCAACTTCACTATC | 5390 |
| OV1 | TCCAGCCTCCCTCCCTGGCCACACAGCCCCTGGCCCTCTCCTGGTGCCATTCACCCTCAACTTCACTATC | 5390 |
| OV2 | TCCAGCCTCCCTCCCTGGCCACACAGCCCCTGGCCCTCTCCTGGTGCCATTCACCCTCAACTTCACTATC | 5390 |
| OV3 | TCCAGCCTCCCTCCCTGGCCACACAGCCCCTGGCCCTCTCCTGGTGCCATTCACCCTCAACTTCACTATC | 5390 |

| | | |
|---|---|---|
| **Consensus** | **ACCAACCTGCAGTATGAGGAGGACATGCGTCACCCTGGTTCCAGGAAGTTCAACACCACGGAGAGAGTCC** | |
| NM_001414687.1 (35,819 .. 46,231) | ACCAACCTGCAGTATGAGGAGGACATGCGTCACCCTGGTTCCAGGAAGTTCAACACCACGGAGAGAGTCC | 5460 |
| Kuramochi | ACCAACCTGCAGTATGAGGAGGACATGCGTCACCCTGGTTCCAGGAAGTTCAACACCACGGAGAGAGTCC | 5460 |
| OVCAR3 | ACCAACCTGCAGTATGAGGAGGACATGCGTCACCCTGGTTCCAGGAAGTTCAACACCACGGAGAGAGTCC | 5460 |
| OVCAR5 | ACCAACCTGCAGTATGAGGAGGACATGCGTCACCCTGGTTCCAGGAAGTTCAACACCACGGAGAGAGTCC | 5460 |
| OV1 | ACCAACCTGCAGTATGAGGAGGACATGCGTCACCCTGGTTCCAGGAAGTTCAACACCACGGAGAGAGTCC | 5460 |
| OV2 | ACCAACCTGCAGTATGAGGAGGACATGCGTCACCCTGGTTCCAGGAAGTTCAACACCACGGAGAGAGTCC | 5460 |
| OV3 | ACCAACCTGCAGTATGAGGAGGACATGCGTCACCCTGGTTCCAGGAAGTTCAACACCACGGAGAGAGTCC | 5460 |

| | | |
|---|---|---|
| **Consensus** | **TGCAGGGTCTGCTCAAGCCCTTGTTCAAGAGCACCAGTGTTGGCCCTCTGTACTCTGGCTGCAGACTGAC** | |
| NM_001414687.1 (35,819 .. 46,231) | TGCAGGGTCTGCTCAAGCCCTTGTTCAAGAGCACCAGTGTTGGCCCTCTGTACTCTGGCTGCAGACTGAC | 5530 |
| Kuramochi | TGCAGGGTCTGCTCAAGCCCTTGTTCAAGAGCACCAGTGTTGGCCCTCTGTACTCTGGCTGCAGACTGAC | 5530 |
| OVCAR3 | TGCAGGGTCTGCTCAAGCCCTTGTTCAAGAGCACCAGTGTTGGCCCTCTGTACTCTGGCTGCAGACTGAC | 5530 |
| OVCAR5 | TGCAGGGTCTGCTCAAGCCCTTGTTCAAGAGCACCAGTGTTGGCCCTCTGTACTCTGGCTGCAGACTGAC | 5530 |
| OV1 | TGCAGGGTCTGCTCAAGCCCTTGTTCAAGAGCACCAGTGTTGGCCCTCTGTACTCTGGCTGCAGACTGAC | 5530 |
| OV2 | TGCAGGGTCTGCTCAAGCCCTTGTTCAAGAGCACCAGTGTTGGCCCTCTGTACTCTGGCTGCAGACTGAC | 5530 |
| OV3 | TGCAGGGTCTGCTCAAGCCCTTGTTCAAGAGCACCAGTGTTGGCCCTCTGTACTCTGGCTGCAGACTGAC | 5530 |

| | | |
|---|---|---|
| **Consensus** | **CTTGCTCAGGCCTGAAAAACGTGGGGCAGCCACCGGCGTGGACACCATCTGCACTCACCGCCTTGACCCT** | |
| NM_001414687.1 (35,819 .. 46,231) | CTTGCTCAGGCCTGAAAAACGTGGGGCAGCCACCGGCGTGGACACCATCTGCACTCACCGCCTTGACCCT | 5600 |
| Kuramochi | CTTGCTCAGGCCTGAAAAACGTGGGGCAGCCACCGGCGTGGACACCATCTGCACTCACCGCCTTGACCCT | 5600 |
| OVCAR3 | CTTGCTCAGGCCTGAAAAACGTGGGGCAGCCACCGGCGTGGACACCATCTGCACTCACCGCCTTGACCCT | 5600 |
| OVCAR5 | CTTGCTCAGGCCTGAAAAACGTGGGGCAGCCACCGGCGTGGACACCATCTGCACTCACCGCCTTGACCCT | 5600 |
| OV1 | CTTGCTCAGGCCTGAAAAACGTGGGGCAGCCACCGGCGTGGACACCATCTGCACTCACCGCCTTGACCCT | 5600 |
| OV2 | CTTGCTCAGGCCTGAAAAACGTGGGGCAGCCACCGGCGTGGACACCATCTGCACTCACCGCCTTGACCCT | 5600 |
| OV3 | CTTGCTCAGGCCTGAAAAACGTGGGGCAGCCACCGGCGTGGACACCATCTGCACTCACCGCCTTGACCCT | 5600 |

| | | |
|---|---|---|
| **Consensus** | **CTAAACCCTGGACTGGACAGAGAGCAGCTATACTGGGAGCTGAGCAAACTGACCCGTGGCATCATCGAGC** | |
| NM_001414687.1 (35,819 .. 46,231) | CTAAACCCTGGACTGGACAGAGAGCAGCTATACTGGGAGCTGAGCAAACTGACCCGTGGCATCATCGAGC | 5670 |
| Kuramochi | CTAAACCCTGGACTGGACAGAGAGCAGCTATACTGGGAGCTGAGCAAACTGACCCGTGGCATCATCGAGC | 5670 |
| OVCAR3 | CTAAACCCTGGACTGGACAGAGAGCAGCTATACTGGGAGCTGAGCAAACTGACCCGTGGCATCATCGAGC | 5670 |
| OVCAR5 | CTAAACCCTGGACTGGACAGAGAGCAGCTATACTGGGAGCTGAGCAAACTGACCCGTGGCATCATCGAGC | 5670 |
| OV1 | CTAAACCCTGGACTGGACAGAGAGCAGCTATACTGGGAGCTGAGCAAACTGACCCGTGGCATCATCGAGC | 5670 |
| OV2 | CTAAACCCTGGACTGGACAGAGAGCAGCTATACTGGGAGCTGAGCAAACTGACCCGTGGCATCATCGAGC | 5670 |
| OV3 | CTAAACCCTGGACTGGACAGAGAGCAGCTATACTGGGAGCTGAGCAAACTGACCCATGGCATCATCGAGC | 5670 |

| | | |
|---|---|---|
| **Consensus** | **TGGGCCCCTACCTCCTGGACAGAGGCAGTCTCTATGTCAATGGTTTCACCCATCGGAACTTTGTGCCCAT** | |
| NM_001414687.1 (35,819 .. 46,231) | TGGGCCCCTACCTCCTGGACAGAGGCAGTCTCTATGTCAATGGTTTCACCCATCGGAACTTTGTGCCCAT | 5740 |
| Kuramochi | TGGGCCCCTACCTCCTGGACAGAGGCAGTCTCTATGTCAATGGTTTCACCCATCGGAACTTTGTGCCCAT | 5740 |
| OVCAR3 | TGGGCCCCTACCTCCTGGACAGAGGCAGTCTCTATGTCAATGGTTTCACCCATCGGAACTTTGTGCCCAT | 5740 |
| OVCAR5 | TGGGCCCCTACCTCCTGGACAGAGGCAGTCTCTATGTCAATGGTTTCACCCATCGGAACTTTGTGCCCAT | 5740 |
| OV1 | TGGGCCCCTACCTCCTGGACAGAGGCAGTCTCTATGTCAATGGTTTCACCCATCGGAACTTTGTGCCCAT | 5740 |
| OV2 | TGGGCCCCTACCTCCTGGACAGAGGCAGTCTCTATGTCAATGGTTTCACCCATCGGAACTTTGTGCCCAT | 5740 |
| OV3 | TGGGCCCCTACCTCCTGGACAGAGGCAGTCTCTATGTCAATGGTTTCACCCATCGGAACTTTGTGCCCAT | 5740 |

| | | |
|---|---|---|
| **Consensus** | **CACCAGCACTCCTGGGACCTCCACAGTACACCTAGGAACCTCTGAAACTCCATCCTCCCTACCTAGACCC** | |
| NM_001414687.1 (35,819 .. 46,231) | CACCAGCACTCCTGGGACCTCCACAGTACACCTAGGAACCTCTGAAACTCCATCCTCCCTACCTAGACCC | 5810 |
| Kuramochi | CACCAGCACTCCTGGGACCTCCACAGTACACCTAGGAACCTCTGAAACTCCATCCTCCCTACCTAGACCC | 5810 |
| OVCAR3 | CACCAGCACTCCTGGGACCTCCACAGTACACCTAGGAACCTCTGAAACTCCATCCTCCCTACCTAGACCC | 5810 |
| OVCAR5 | CACCAGCACTCCTGGGACCTCCACAGTACACCTAGGAACCTCTGAAACTCCATCCTCCCTACCTAGACCC | 5810 |
| OV1 | CACCAGCACTCCTGGGACCTCCACAGTACACCTAGGAACCTCTGAAACTCCATCCTCCCTACCTAGACCC | 5810 |
| OV2 | CACCAGCACTCCTGGGACCTCCACAGTACACCTAGGAACCTCTGAAACTCCATCCTCCCTACCTAGACCC | 5810 |
| OV3 | CACCAGCACTCCTGGGACCTCCACAGTACACCTAGGAACCTCTGAAACTCCATCCTCCCTACCTAGACCC | 5810 |

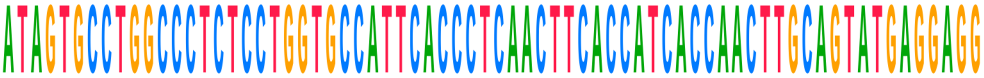


| | | |
|---|---|---|
| **Consensus** | **ATAGTGCCTGGCCCTCTCCTGGTGCCATTCACCCTCAACTTCACCATCACCAACTTGCAGTATGAGGAGG** | |
| NM_001414687.1 (35,819 .. 46,231) | ATAGTGCCTGGCCCTCTCCTGGTGCCATTCACCCTCAACTTCACCATCACCAACTTGCAGTATGAGGAGG | 5880 |
| Kuramochi | ATAGTGCCTGGCCCTCTCCTGGTGCCATTCACCCTCAACTTCACCATCACCAACTTGCAGTATGAGGAGG | 5880 |
| OVCAR3 | ATAGTGCCTGGCCCTCTCCTGGTGCCATTCACCCTCAACTTCACCATCACCAACTTGCAGTATGAGGAGG | 5880 |
| OVCAR5 | ATAGTGCCTGGCCCTCTCCTGGTGCCATTCACCCTCAACTTCACCATCACCAACTTGCAGTATGAGGAGG | 5880 |
| OV1 | ATAGTGCCTGGCCCTCTCCTGGTGCCATTCACCCTCAACTTCACCATCACCAACTTGCAGTATGAGGAGG | 5880 |
| OV2 | ATAGTGCCTGGCCCTCTCCTGGTGCCATTCACCCTCAACTTCACCATCACCAACTTGCAGTATGAGGAGG | 5880 |
| OV3 | ATAGTGCCTGGCCCTCTCCTGGTGCCATTCACCCTCAACTTCACCATCACCAACTTGCAGTATGAGGAGG | 5880 |

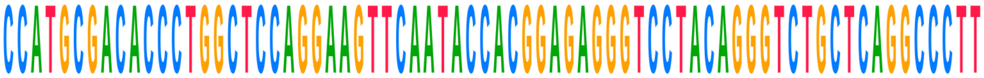


| | | |
|---|---|---|
| **Consensus** | **CCATGCGACACCCTGGCTCCAGGAAGTTCAATACCACGGAGAGGGTCCTACAGGGTCTGCTCAGGCCCTT** | |
| NM_001414687.1 (35,819 .. 46,231) | CCATGCGACACCCTGGCTCCAGGAAGTTCAATACCACGGAGAGGGTCCTACAGGGTCTGCTCAGGCCCTT | 5950 |
| Kuramochi | CCATGCGACACCCTGGCTCCAGGAAGTTCAATACCACGGAGAGGGTCCTACAGGGTCTGCTCAGGCCCTT | 5950 |
| OVCAR3 | CCATGCGACACCCTGGCTCCAGGAAGTTCAATACCACGGAGAGGGTCCTACAGGGTCTGCTCAGGCCCTT | 5950 |
| OVCAR5 | CCATGCGACACCCTGGCTCCAGGAAGTTCAATACCACGGAGAGGGTCCTACAGGGTCTGCTCAGGCCCTT | 5950 |
| OV1 | CCATGCGACACCCTGGCTCCAGGAAGTTCAATACCACGGAGAGGGTCCTACAGGGTCTGCTCAGGCCCTT | 5950 |
| OV2 | CCATGCGACACCCTGGCTCCAGGAAGTTCAATACCACGGAGAGGGTCCTACAGGGTCTGCTCAGGCCCTT | 5950 |
| OV3 | CCATGCGACACCCTGGCTCCAGGAAGTTCAATACCACGGAGAGGGTCCTACAGGGTCTGCTCAGGCCCTT | 5950 |

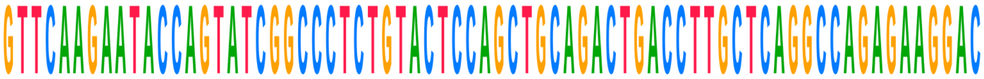


| | | |
|---|---|---|
| **Consensus** | **GTTCAAGAATACCAGTATCGGCCCTCTGTACTCCAGCTGCAGACTGACCTTGCTCAGGCCAGAGAAGGAC** | |
| NM_001414687.1 (35,819 .. 46,231) | GTTCAAGAATACCAGTATCGGCCCTCTGTACTCCAGCTGCAGACTGACCTTGCTCAGGCCAGAGAAGGAC | 6020 |
| Kuramochi | GTTCAAGAATACCAGTATCGGCCCTCTGTACTCCAGCTGCAGACTGACCTTGCTCAGGCCAGAGAAGGAC | 6020 |
| OVCAR3 | GTTCAAGAATACCAGTATCGGCCCTCTGTACTCCAGCTGCAGACTGACCTTGCTCAGGCCAGAGAAGGAC | 6020 |
| OVCAR5 | GTTCAAGAATACCAGTATCGGCCCTCTGTACTCCAGCTGCAGACTGACCTTGCTCAGGCCAGAGAAGGAC | 6020 |
| OV1 | GTTCAAGAATACCAGTATCGGCCCTCTGTACTCCAGCTGCAGACTGACCTTGCTCAGGCCAGAGAAGGAC | 6020 |
| OV2 | GTTCAAGAATACCAGTATCGGCCCTCTGTACTCCAGCTGCAGACTGACCTTGCTCAGGCCAGAGAAGGAC | 6020 |
| OV3 | GTTCAAGAATACCAGTATCGGCCCTCTGTACTCCAGCTGCAGACTGACCTTGCTCAGGCCAGAGAAGGAC | 6020 |

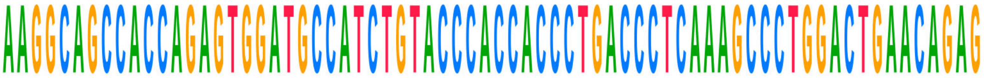


| | | |
|---|---|---|
| **Consensus** | **AAGGCAGCCACCAGAGTGGATGCCATCTGTACCCACCACCCTGACCCTCAAAGCCCTGGACTGAACAGAG** | |
| NM_001414687.1 (35,819 .. 46,231) | AAGGCAGCCACCAGAGTGGATGCCATCTGTACCCACCACCCTGACCCTCAAAGCCCTGGACTGAACAGAG | 6090 |
| Kuramochi | AAGGCAGCCACCAGAGTGGATGCCATCTGTACCCACCACCCTGACCCTCAAAGCCCTGGACTGAACAGAG | 6090 |
| OVCAR3 | AAGGCAGCCACCAGAGTGGATGCCATCTGTACCCACCACCCTGACCCTCAAAGCCCTGGACTGAACAGAG | 6090 |
| OVCAR5 | AAGGCAGCCACCAGAGTGGATGCCATCTGTACCCACCACCCTGACCCTCAAAGCCCTGGACTGAACAGAG | 6090 |
| OV1 | AAGGCAGCCACCAGAGTGGATGCCATCTGTACCCACCACCCTGACCCTCAAAGCCCTGGACTGAACAGAG | 6090 |
| OV2 | AAGGCAGCCACCAGAGTGGATGCCATCTGTACCCACCACCCTGACCCTCAAAGCCCTGGACTGAACAGAG | 6090 |
| OV3 | AAGGCAGCCACCAGAGTGGATGCCATCTGTACCCACCACCCTGACCCTCAAAGCCCTGGACTGAACAGAG | 6090 |

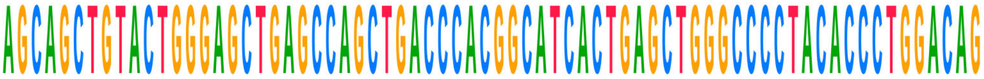


| | | |
|---|---|---|
| **Consensus** | **AGCAGCTGTACTGGGAGCTGAGCCAGCTGACCCACGGCATCACTGAGCTGGGCCCCTACACCCTGGACAG** | |
| NM_001414687.1 (35,819 .. 46,231) | AGCAGCTGTACTGGGAGCTGAGCCAGCTGACCCACGGCATCACTGAGCTGGGCCCCTACACCCTGGACAG | 6160 |
| Kuramochi | AGCAGCTGTACTGGGAGCTGAGCCAGCTGACCCACGGCATCACTGAGCTGGGCCCCTACACCCTGGACAG | 6160 |
| OVCAR3 | AGCAGCTGTACTGGGAGCTGAGCCAGCTGACCCACGGCATCACTGAGCTGGGCCCCTACACCCTGGACAG | 6160 |
| OVCAR5 | AGCAGCTGTACTGGGAGCTGAGCCAGCTGACCCACGGCATCACTGAGCTGGGCCCCTACACCCTGGACAG | 6160 |
| OV1 | AGCAGCTGTACTGGGAGCTGAGCCAGCTGACCCACGGCATCACTGAGCTGGGCCCCTACACCCTGGACAG | 6160 |
| OV2 | AGCAGCTGTACTGGGAGCTGAGCCAGCTGACCCACGGCATCACTGAGCTGGGCCCCTACACCCTGGACAG | 6160 |
| OV3 | AGCAGCTGTACTGGGAGCTGAGCCAGCTGACCCACGGCATCACTGAGCTGGGCCCCTACACCCTGGACAG | 6160 |

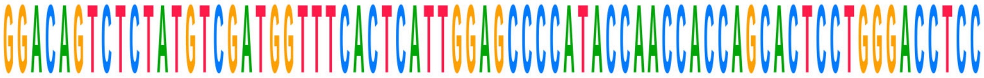


| | | |
|---|---|---|
| **Consensus** | **GGACAGTCTCTATGTCGATGGTTTCACTCATTGGAGCCCCATACCAACCACCAGCACTCCTGGGACCTCC** | |
| NM_001414687.1 (35,819 .. 46,231) | GGACAGTCTCTATGTCGATGGTTTCACTCATTGGAGCCCCATACCAACCACCAGCACTCCTGGGACCTCC | 6230 |
| Kuramochi | GGACAGTCTCTATGTCGATGGTTTCACTCATTGGAGCCCCATACCAACCACCAGCACTCCTGGGACCTCC | 6230 |
| OVCAR3 | GGACAGTCTCTATGTCGATGGTTTCACTCATTGGAGCCCCATACCAACCACCAGCACTCCTGGGACCTCC | 6230 |
| OVCAR5 | GGACAGTCTCTATGTCGATGGTTTCACTCATTGGAGCCCCATACCAACCACCAGCACTCCTGGGACCTCC | 6230 |
| OV1 | GGACAGTCTCTATGTCGATGGTTTCACTCATTGGAGCCCCATACCAACCACCAGCACTCCTGGGACCTCC | 6230 |
| OV2 | GGACAGTCTCTATGTCGATGGTTTCACTCATTGGAGCCCCATACCAACCACCAGCACTCCTGGGACCTCC | 6230 |
| OV3 | GGACAGTCTCTATGTCGATGGTTTCACTCATTGGAGCCCCATACCAACCACCAGCACTCCTGGGACCTCC | 6230 |

| | | |
|---|---|---|
| **Consensus** | **ATAGTGAACCTGGGAACCTCTGGGATCCCACCTTCCCTCCCTGAAACTACAGCCACCGGCCCTCTCCTGG** | |
| NM_001414687.1 (35,819 .. 46,231) | ATAGTGAACCTGGGAACCTCTGGGATCCCACCTTCCCTCCCTGAAACTACAGCCACCGGCCCTCTCCTGG | 6300 |
| Kuramochi | ATAGTGAACCTGGGAACCTCTGGGATCCCACCTTCCCTCCCTGAAACTACAGCCACCGGCCCTCTCCTGG | 6300 |
| OVCAR3 | ATAGTGAACCTGGGAACCTCTGGGATCCCACCTTCCCTCCCTGAAACTACAGCCACCGGCCCTCTCCTGG | 6300 |
| OVCAR5 | ATAGTGAACCTGGGAACCTCTGGGATCCCACCTTCCCTCCCTGAAACTACAGCCACCGGCCCTCTCCTGG | 6300 |
| OV1 | ATAGTGAACCTGGGAACCTCTGGGATCCCACCTTCCCTCCCTGAAACTACAGCCACCGGCCCTCTCCTGG | 6300 |
| OV2 | ATAGTGAACCTGGGAACCTCTGGGATCCCACCTTCCCTCCCTGAAACTACAGCCACCGGCCCTCTCCTGG | 6300 |
| OV3 | ATAGTGAACCTGGGAACCTCTGGGATCCCACCTTCCCTCCCTGAAACTACAGCCACCGGCCCTCTCCTGA | 6300 |

| | | |
|---|---|---|
| **Consensus** | **TGCCATTCACACTCAACTTCACCATCACTAACCTACAGTATGAGGAGAACATGGGTCACCCTGGCTCCAG** | |
| NM_001414687.1 (35,819 .. 46,231) | TGCCATTCACACTCAACTTCACCATCACTAACCTACAGTATGAGGAGAACATGGGTCACCCTGGCTCCAG | 6370 |
| Kuramochi | TGCCATTCACACTCAACTTCACCATCACTAACCTACAGTATGAGGAGAACATGGGTCACCCTGGCTCCAG | 6370 |
| OVCAR3 | TGCCATTCACACTCAACTTCACCATCACTAACCTACAGTATGAGGAGAACATGGGTCACCCTGGCTCCAG | 6370 |
| OVCAR5 | TGCCATTCACACTCAACTTCACCATCACTAACCTACAGTATGAGGAGAACATGGGTCACCCTGGCTCCAG | 6370 |
| OV1 | TGCCATTCACACTCAACTTCACCATCACTAACCTACAGTATGAGGAGAACATGGGTCACCCTGGCTCCAG | 6370 |
| OV2 | TGCCATTCACACTCAACTTCACCATCACTAACCTACAGTATGAGGAGAACATGGGTCACCCTGGCTCCAG | 6370 |
| OV3 | TGCCATTCACACTCAACTTCACCATCACTAACCTACAGTATGAGGAGAACATGGGTCACCCTGGCTCCAG | 6370 |

| | | |
|---|---|---|
| **Consensus** | **GAAGTTCAACATCACGGAGAGTGTTCTGCAGGGTCTGCTCAAGCCCTTGTTCAAGAGCACCAGTGTTGGC** | |
| NM_001414687.1 (35,819 .. 46,231) | GAAGTTCAACATCACGGAGAGTGTTCTGCAGGGTCTGCTCAAGCCCTTGTTCAAGAGCACCAGTGTTGGC | 6440 |
| Kuramochi | GAAGTTCAACATCACGGAGAGTGTTCTGCAGGGTCTGCTCAAGCCCTTGTTCAAGAGCACCAGTGTTGGC | 6440 |
| OVCAR3 | GAAGTTCAACATCACGGAGAGTGTTCTGCAGGGTCTGCTCAAGCCCTTGTTCAAGAGCACCAGTGTTGGC | 6440 |
| OVCAR5 | GAAGTTCAACATCACGGAGAGTGTTCTGCAGGGTCTGCTCAAGCCCTTGTTCAAGAGCACCAGTGTTGGC | 6440 |
| OV1 | GAAGTTCAACATCACGGAGAGTGTTCTGCAGGGTCTGCTCAAGCCCTTGTTCAAGAGCACCAGTGTTGGC | 6440 |
| OV2 | GAAGTTCAACATCACGGAGAGTGTTCTGCAGGGTCTGCTCAAGCCCTTGTTCAAGAGCACCAGTGTTGGC | 6440 |
| OV3 | GAAGTTCAACATCACGGAGAGTGTTCTGCAGGGTCTGCTCAAGCCCTTGTTCAAGAGCACCAGTGTTGGC | 6440 |

| | | |
|---|---|---|
| **Consensus** | **CCTCTGTATTCTGGCTGCAGACTGACCTTGCTCAGGCCTGAGAAGGACGGAGTAGCCACCAGAGTGGACG** | |
| NM_001414687.1 (35,819 .. 46,231) | CCTCTGTATTCTGGCTGCAGACTGACCTTGCTCAGGCCTGAGAAGGATGGAGTAGCCACCAGAGTGGACG | 6510 |
| Kuramochi | CCTCTGTATTCTGGCTGCAGACTGACCTTGCTCAGGCCTGAGAAGGACGGAGTAGCCACCAGAGTGGACG | 6510 |
| OVCAR3 | CCTCTGTATTCTGGCTGCAGACTGACCTTGCTCAGGCCTGAGAAGGACGGAGTAGCCACCAGAGTGGACG | 6510 |
| OVCAR5 | CCTCTGTATTCTGGCTGCAGACTGACCTTGCTCAGGCCTGAGAAGGACGGAGTAGCCACCAGAGTGGACG | 6510 |
| OV1 | CCTCTGTATTCTGGCTGCAGACTGACCTTGCTCAGGCCTGAGAAGGACGGAGTAGCCACCAGAGTGGACG | 6510 |
| OV2 | CCTCTGTATTCTGGCTGCAGACTGACCTTGCTCAGGCCTGAGAAGGACGGAGTAGCCACCAGAGTGGACG | 6510 |
| OV3 | CCTCTGTATTCTGGCTGCAGACTGACCTTGCTCAGGCCTGAGAAGGATGGAGTAGCCACCAGAGTGGACG | 6510 |

| | | |
|---|---|---|
| **Consensus** | **CCATCTGCACCCACCGCCCTGACCCCAAAATCCCTGGGCTAGACAGACAGCAGCTATACTGGGAGCTGAG** | |
| NM_001414687.1 (35,819 .. 46,231) | CCATCTGCACCCACCGCCCTGACCCCAAAATCCCTGGGCTAGACAGACAGCAGCTATACTGGGAGCTGAG | 6580 |
| Kuramochi | CCATCTGCACCCACCGCCCTGACCCCAAAATCCCTGGGCTAGACAGACAGCAGCTATACTGGGAGCTGAG | 6580 |
| OVCAR3 | CCATCTGCACCCACCGCCCTGACCCCAAAATCCCTGGGCTAGACAGACAGCAGCTATACTGGGAGCTGAG | 6580 |
| OVCAR5 | CCATCTGCACCCACCGCCCTGACCCCAAAATCCCTGGGCTAGACAGACAGCAGCTATACTGGGAGCTGAG | 6580 |
| OV1 | CCATCTGCACCCACCGCCCTGATCCCAAAATCCCTGGGCTAGACAGACAGCAGCTATACTGGGAGCTGAG | 6580 |
| OV2 | CCATCTGCACCCACCGCCCTGATCCCAAAATCCCTGGGCTAGACAGACAGCAGCTATACTGGGAGCTGAG | 6580 |
| OV3 | CCATCTGCACCCACCGCCCTGACCCCAAAATCCCTGGGCTAGACAGACAGCAGCTATACTGGGAGCTGAG | 6580 |

| | | |
|---|---|---|
| **Consensus** | **CCAGCTGACCCACAGCATCACTGAGCTGGGACCCTACACCCTGGATAGGGACAGTCTCTATGTCAATGGT** | |
| NM_001414687.1 (35,819 .. 46,231) | CCAGCTGACCCACAGCATCACTGAGCTGGGACCCTACACCCTGGATAGGGACAGTCTCTATGTCAATGGT | 6650 |
| Kuramochi | CCAGCTGACCCACAGCATCACTGAGCTGGGACCCTACACCCTGGATAGGGACAGTCTCTATGTCAATGGT | 6650 |
| OVCAR3 | CCAGCTGACCCACAGCATCACTGAGCTGGGACCCTACACCCTGGATAGGGACAGTCTCTATGTCAATGGT | 6650 |
| OVCAR5 | CCAGCTGACCCACAGCATCACTGAGCTGGGACCCTACACCCTGGATAGGGACAGTCTCTATGTCAATGGT | 6650 |
| OV1 | CCAGCTGACCCACAGCATCACTGAGCTGGGACCCTACACCCTGGATAGGGACAGTCTCTATGTCAATGGT | 6650 |
| OV2 | CCAGCTGACCCACAGCATCACTGAGCTGGGACCCTACACCCTGGATAGGGACAGTCTCTATGTCAATGGT | 6650 |
| OV3 | CCAGCTGACCCACAGCATCACTGAGCTGGGACCCTACACCCTGGATAGGGACAGTCTCTATGTCAATGGT | 6650 |

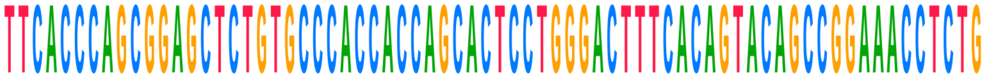

| | | |
|---|---|---|
| **Consensus** | **TTCACCCAGCGGAGCTCTGTGCCCACCACCAGCACTCCTGGGACTTTCACAGTACAGCCGGAAACCTCTG** | |
| NM_001414687.1 (35,819 .. 46,231) | TTCACCCAGCGGAGCTCTGTGCCCACCACCAGCACTCCTGGGACTTTCACAGTACAGCCGGAAACCTCTG | 6720 |
| Kuramochi | TTCACCCAGCGGAGCTCTGTGCCCACCACCAGCACTCCTGGGACTTTCACAGTACAGCCGGAAACCTCTG | 6720 |
| OVCAR3 | TTCACCCAGCGGAGCTCTGTGCCCACCACCAGCACTCCTGGGACTTTCACAGTACAGCCGGAAACCTCTG | 6720 |
| OVCAR5 | TTCACCCAGCGGAGCTCTGTGCCCACCACCAGCACTCCTGGGACTTTCACAGTACAGCCGGAAACCTCTG | 6720 |
| OV1 | TTCACCCAGCGGAGCTCTGTGCCCACCACCAGCACTCCTGGGACTTTCACAGTACAGCCGGAAACCTCTG | 6720 |
| OV2 | TTCACCCAGCGGAGCTCTGTGCCCACCACCAGCACTCCTGGGACTTTCACAGTACAGCCGGAAACCTCTG | 6720 |
| OV3 | TTCACCCAGCGGAGCTCTGTGCCCACCACCAGCACTCCTGGGACTTTCACAGTACAGCCGGAAACCTCTG | 6720 |

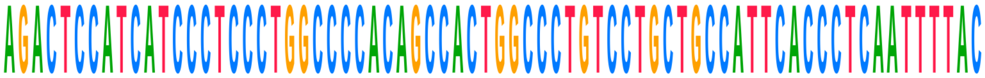

| | | |
|---|---|---|
| **Consensus** | **AGACTCCATCATCCCTCCCTGGCCCCACAGCCACTGGCCCTGTCCTGCTGCCATTCACCCTCAATTTTAC** | |
| NM_001414687.1 (35,819 .. 46,231) | AGACTCCATCATCCCTCCCTGGCCCCACAGCCACTGGCCCTGTCCTGCTGCCATTCACCCTCAATTTTAC | 6790 |
| Kuramochi | AGACTCCATCATCCCTCCCTGGCCCCACAGCCACTGGCCCTGTCCTGCTGCCATTCACCCTCAATTTTAC | 6790 |
| OVCAR3 | AGACTCCATCATCCCTCCCTGGCCCCACAGCCACTGGCCCTGTCCTGCTGCCATTCACCCTCAATTTTAC | 6790 |
| OVCAR5 | AGACTCCATCATCCCTCCCTGGCCCCACAGCCACTGGCCCTGTCCTGCTGCCATTCACCCTCAATTTTAC | 6790 |
| OV1 | AGACTCCATCATCCCTCCCTGGCCCCACAGCCACTGGCCCTGTCCTGCTGCCATTCACCCTCAATTTTAC | 6790 |
| OV2 | AGACTCCATCATCCCTCCCTGGCCCCACAGCCACTGGCCCTGTCCTGCTGCCATTCACCCTCAATTTTAC | 6790 |
| OV3 | AGACTCCATCATCCCTCCCTGGCCCCACAGCCACTGGCCCTGTCCTGCTGCCATTCACCCTCAATTTTAC | 6790 |

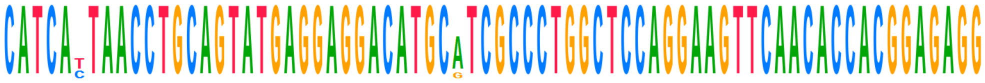

| | | |
|---|---|---|
| **Consensus** | **CATCATTAACCTGCAGTATGAGGAGGACATGCATCGCCCTGGCTCCAGGAAGTTCAACACCACGGAGAGG** | |
| NM_001414687.1 (35,819 .. 46,231) | CATCACTAACCTGCAGTATGAGGAGGACATGCGTCGCCCTGGCTCCAGGAAGTTCAACACCACGGAGAGG | 6860 |
| Kuramochi | CATCATTAACCTGCAGTATGAGGAGGACATGCATCGCCCTGGCTCCAGGAAGTTCAACACCACGGAGAGG | 6860 |
| OVCAR3 | CATCATTAACCTGCAGTATGAGGAGGACATGCATCGCCCTGGCTCCAGGAAGTTCAACACCACGGAGAGG | 6860 |
| OVCAR5 | CATCATTAACCTGCAGTATGAGGAGGACATGCATCGCCCTGGCTCCAGGAAGTTCAACACCACGGAGAGG | 6860 |
| OV1 | CATCATTAACCTGCAGTATGAGGAGGACATGCATCGCCCTGGCTCCAGGAAGTTCAACACCACGGAGAGG | 6860 |
| OV2 | CATCACTAACCTGCAGTATGAGGAGGACATGCATCGCCCTGGCTCCAGGAAGTTCAACACCACGGAGAGG | 6860 |
| OV3 | CATCACTAACCTGCAGTATGAGGAGGACATGCATCGCCCTGGCTCCAGGAAGTTCAACACCACGGAGAGG | 6860 |

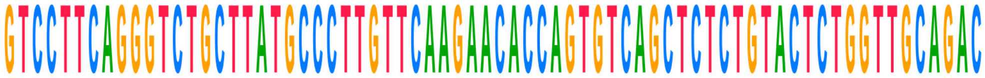

| | | |
|---|---|---|
| **Consensus** | **GTCCTTCAGGGTCTGCTTATGCCCTTGTTCAAGAACACCAGTGTCAGCTCTCTGTACTCTGGTTGCAGAC** | |
| NM_001414687.1 (35,819 .. 46,231) | GTCCTTCAGGGTCTGCTTATGCCCTTGTTCAAGAACACCAGTGTCAGCTCTCTGTACTCTGGTTGCAGAC | 6930 |
| Kuramochi | GTCCTTCAGGGTCTGCTTATGCCCTTGTTCAAGAACACCAGTGTCAGCTCTCTGTACTCTGGTTGCAGAC | 6930 |
| OVCAR3 | GTCCTTCAGGGTCTGCTTATGCCCTTGTTCAAGAACACCAGTGTCAGCTCTCTGTACTCTGGTTGCAGAC | 6930 |
| OVCAR5 | GTCCTTCAGGGTCTGCTTATGCCCTTGTTCAAGAACACCAGTGTCAGCTCTCTGTACTCTGGTTGCAGAC | 6930 |
| OV1 | GTCCTTCAGGGTCTGCTTATGCCCTTGTTCAAGAACACCAGTGTCAGCTCTCTGTACTCTGGTTGCAGAC | 6930 |
| OV2 | GTCCTTCAGGGTCTGCTTATGCCCTTGTTCAAGAACACCAGTGTCAGCTCTCTGTACTCTGGTTGCAGAC | 6930 |
| OV3 | GTCCTTCAGGGTCTGCTTATGCCCTTGTTCAAGAACACCAGTGTCAGCTCTCTGTACTCTGGTTGCAGAC | 6930 |

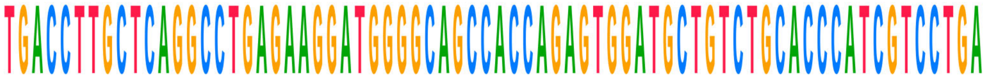

| | | |
|---|---|---|
| **Consensus** | **TGACCTTGCTCAGGCCTGAGAAGGATGGGGCAGCCACCAGAGTGGATGCTGTCTGCACCCATCGTCCTGA** | |
| NM_001414687.1 (35,819 .. 46,231) | TGACCTTGCTCAGGCCTGAGAAGGATGGGGCAGCCACCAGAGTGGATGCTGTCTGCACCCATCGTCCTGA | 7000 |
| Kuramochi | TGACCTTGCTCAGGCCTGAGAAGGATGGGGCAGCCACCAGAGTGGATGCTGTCTGCACCCATCGTCCTGA | 7000 |
| OVCAR3 | TGACCTTGCTCAGGCCTGAGAAGGATGGGGCAGCCACCAGAGTGGATGCTGTCTGCACCCATCGTCCTGA | 7000 |
| OVCAR5 | TGACCTTGCTCAGGCCTGAGAAGGATGGGGCAGCCACCAGAGTGGATGCTGTCTGCACCCATCGTCCTGA | 7000 |
| OV1 | TGACCTTGCTCAGGCCTGAGAAGGATGGGGCAGCCACCAGAGTGGATGCTGTCTGCACCCATCGTCCTGA | 7000 |
| OV2 | TGACCTTGCTCAGGCCTGAGAAGGATGGGGCAGCCACCAGAGTGGATGCTGTCTGCACCCATCGTCCTGA | 7000 |
| OV3 | TGACCTTGCTCAGGCCTGAGAAGGATGGGGCAGCCACCAGAGTGGATGCTGTCTGCACCCATCGTCCTGA | 7000 |

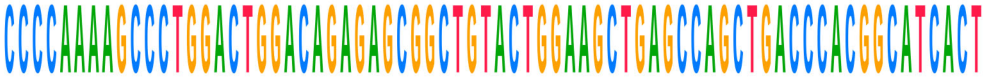

| | | |
|---|---|---|
| **Consensus** | **CCCCAAAAGCCCTGGACTGGACAGAGAGCGGCTGTACTGGAAGCTGAGCCAGCTGACCCACGGCATCACT** | |
| NM_001414687.1 (35,819 .. 46,231) | CCCCAAAAGCCCTGGACTGGACAGAGAGCGGCTGTACTGGAAGCTGAGCCAGCTGACCCACGGCATCACT | 7070 |
| Kuramochi | CCCCAAAAGCCCTGGACTGGACAGAGAGCGGCTGTACTGGAAGCTGAGCCAGCTGACCCACGGCATCACT | 7070 |
| OVCAR3 | CCCCAAAAGCCCTGGACTGGACAGAGAGCGGCTGTACTGGAAGCTGAGCCAGCTGACCCACGGCATCACT | 7070 |
| OVCAR5 | CCCCAAAAGCCCTGGACTGGACAGAGAGCGGCTGTACTGGAAGCTGAGCCAGCTGACCCACGGCATCACT | 7070 |
| OV1 | CCCCAAAAGCCCTGGACTGGACAGAGAGCGGCTGTACTGGAAGCTGAGCCAGCTGACCCACGGCATCACT | 7070 |
| OV2 | CCCCAAAAGCCCTGGACTGGACAGAGAGCGGCTGTACTGGAAGCTGAGCCAGCTGACCCACGGCATCACT | 7070 |
| OV3 | CCCCAAAAGCCCTGGACTGGACAGAGAGCGGCTGTACTGGAAGCTGAGCCAGCTGACCCACGGCATCACT | 7070 |

| | | |
|---|---|---|
| **Consensus** | **GAGCTGGGCCCCTACACCCTGGACAGGCACAGTCTCTATGTCAATGGTTTCACCCATCAGAGCTCTATGA** | |
| NM_001414687.1 (35,819 .. 46,231) | GAGCTGGGCCCCTACACCCTGGACAGGCACAGTCTCTATGTCAATGGTTTCACCCATCAGAGCTCTATGA | 7140 |
| Kuramochi | GAGCTGGGCCCCTACACCCTGGACAGGCACAGTCTCTATGTCAATGGTTTCACCCATCAGAGCTCTATGA | 7140 |
| OVCAR3 | GAGCTGGGCCCCTACACCCTGGACAGGCACAGTCTCTATGTCAATGGTTTCACCCATCAGAGCTCTATGA | 7140 |
| OVCAR5 | GAGCTGGGCCCCTACACCCTGGACAGGCACAGTCTCTATGTCAATGGTTTCACCCATCAGAGCTCTATGA | 7140 |
| OV1 | GAGCTGGGCCCCTACACCCTGGACAGGCACAGTCTCTATGTCAATGGTTTCACCCATCAGAGCTCTATGA | 7140 |
| OV2 | GAGCTGGGCCCCTACACCCTGGACAGGCACAGTCTCTATGTCAATGGTTTCACCCATCAGAGCTCTATGA | 7140 |
| OV3 | GAGCTGGGCCCCTACACCCTGGACAGGCACAGTCTCTATGTCAATGGTTTCACCCATCAGAGCTCTATGA | 7140 |

| | | |
|---|---|---|
| **Consensus** | **CGACCACCAGAACTCCTGATACCTCCACAATGCACCTGGCAACCTCGAGAACTCCAGCCTCCCTGTCTGG** | |
| NM_001414687.1 (35,819 .. 46,231) | CGACCACCAGAACTCCTGATACCTCCACAATGCACCTGGCAACCTCGAGAACTCCAGCCTCCCTGTCTGG | 7210 |
| Kuramochi | CGACCACCAGAACTCCTGATACCTCCACAATGCACCTGGCAACCTCGAGAACTCCAGCCTCCCTGTCTGG | 7210 |
| OVCAR3 | CGACCACCAGAACTCCTGATACCTCCACAATGCACCTGGCAACCTCGAGAACTCCAGCCTCCCTGTCTGG | 7210 |
| OVCAR5 | CGACCACCAGAACTCCTGATACCTCCACAATGCACCTGGCAACCTCGAGAACTCCAGCCTCCCTGTCTGG | 7210 |
| OV1 | CGACCACCAGAACTCCTGATACCTCCACAATGCACCTGGCAACCTCGAGAACTCCAGCCTCCCTGTCTGG | 7210 |
| OV2 | CGACCACCAGAACTCCTGATACCTCCACAATGCACCTGGCAACCTCGAGAACTCCAGCCTCCCTGTCTGG | 7210 |
| OV3 | CGACCACCAGAACTCCTGATACCTCCACAATGCACCTGGCAACCTCGAGAACTCCAGCCTCCCTGTCTGG | 7210 |

| | | |
|---|---|---|
| **Consensus** | **ACCTACGACCGCCAGCCCTCTCCTGGTGCTATTCACAATTAACTTCACCATCACTAACCTGCGGTATGAG** | |
| NM_001414687.1 (35,819 .. 46,231) | ACCCATGACCGCCAGCCCTCTCCTGGTGCTATTCACAATTAACTTCACCATCACTAACCTGCGGTATGAG | 7280 |
| Kuramochi | ACCTACGACCGCCAGCCCTCTCCTGGTGCTATTCACAATTAACTTCACCATCACTAACCTGCGGTATGAG | 7280 |
| OVCAR3 | ACCTACGACCGCCAGCCCTCTCCTGGTGCTATTCACAATTAACTTCACCATCACTAACCTGCGGTATGAG | 7280 |
| OVCAR5 | ACCTACGACCGCCAGCCCTCTCCTGGTGCTATTCACAATTAACTTCACCATCACTAACCTGCGGTATGAG | 7280 |
| OV1 | ACCTACGACCGCCAGCCCTCTCCTGGTGCTATTCACAATTAACTTCACCATCACTAACCTGCGGTATGAG | 7280 |
| OV2 | ACCTACGACCGCCAGCCCTCTCCTGGTGCTATTCACAATTAACTTCACCATCACTAACCTGCGGTATGAG | 7280 |
| OV3 | ACCTATGACCGCCAGCCCTCTCCTGGTGCTATTCACAATTAACTTCACCATCACTAACCTGCGGTATGAG | 7280 |

| | | |
|---|---|---|
| **Consensus** | **GAGAACATGCATCACCCTGGCTCTAGAAAGTTTAACACCACGGAGAGAGTCCTTCAGGGTCTGCTCAGGC** | |
| NM_001414687.1 (35,819 .. 46,231) | GAGAACATGCATCACCCTGGCTCTAGAAAGTTTAACACCACGGAGAGAGTCCTTCAGGGTCTGCTCAGGC | 7350 |
| Kuramochi | GAGAACATGCATCACCCTGGCTCTAGAAAGTTTAACACCACGGAGAGAGTCCTTCAGGGTCTGCTCAGGC | 7350 |
| OVCAR3 | GAGAACATGCATCACCCTGGCTCTAGAAAGTTTAACACCACGGAGAGAGTCCTTCAGGGTCTGCTCAGGC | 7350 |
| OVCAR5 | GAGAACATGCATCACCCTGGCTCTAGAAAGTTTAACACCACGGAGAGAGTCCTTCAGGGTCTGCTCAGGC | 7350 |
| OV1 | GAGAACATGCATCACCCTGGCTCTAGAAAGTTTAACACCACGGAGAGAGTCCTTCAGGGTCTGCTCAGGC | 7350 |
| OV2 | GAGAACATGCATCACCCTGGCTCTAGAAAGTTTAACACCACGGAGAGAGTCCTTCAGGGTCTGCTCAGGC | 7350 |
| OV3 | GAGAACATGCATCACCCTGGCTCTAGAAAGTTTAACACCACGGAGAGAGTCCTTCAGGGTCTGCTCAGGC | 7350 |

| | | |
|---|---|---|
| **Consensus** | **CTGTGTTCAAGAACACCAGTGTTGGCCCTCTGTACTCTGGCTGCAGACTGACCTTGCTCAGGCCCAAGAA** | |
| NM_001414687.1 (35,819 .. 46,231) | CTGTGTTCAAGAACACCAGTGTTGGCCCTCTGTACTCTGGCTGCAGACTGACCTTGCTCAGGCCCAAGAA | 7420 |
| Kuramochi | CTGTGTTCAAGAACACCAGTGTTGGCCCTCTGTACTCTGGCTGCAGACTGACCTTGCTCAGGCCCAAGAA | 7420 |
| OVCAR3 | CTGTGTTCAAGAACACCAGTGTTGGCCCTCTGTACTCTGGCTGCAGACTGACCTTGCTCAGGCCCAAGAA | 7420 |
| OVCAR5 | CTGTGTTCAAGAACACCAGTGTTGGCCCTCTGTACTCTGGCTGCAGACTGACCTTGCTCAGGCCCAAGAA | 7420 |
| OV1 | CTGTGTTCAAGAACACCAGTGTTGGCCCTCTGTACTCTGGCTGCAGACTGACCTTGCTCAGGCCCAAGAA | 7420 |
| OV2 | CTGTGTTCAAGAACACCAGTGTTGGCCCTCTGTACTCTGGCTGCAGACTGACCTTGCTCAGGCCCAAGAA | 7420 |
| OV3 | CTGTGTTCAAGAACACCAGTGTTGGCCCTCTGTACTCTGGCTGCAGACTGACCTTGCTCAGGCCCAAGAA | 7420 |

| | | |
|---|---|---|
| **Consensus** | **GGATGGGGCAGCCACCAAAGTGGATGCCATCTGCACCTACCGCCCTGATCCCAAAAGCCCTGGACTGGAC** | |
| NM_001414687.1 (35,819 .. 46,231) | GGATGGGGCAGCCACCAAAGTGGATGCCATCTGCACCTACCGCCCTGATCCCAAAAGCCCTGGACTGGAC | 7490 |
| Kuramochi | GGATGGGGCAGCCACCAAAGTGGATGCCATCTGCACCTACCGCCCTGATCCCAAAAGCCCTGGACTGGAC | 7490 |
| OVCAR3 | GGATGGGGCAGCCACCAAAGTGGATGCCATCTGCACCTACCGCCCTGATCCCAAAAGCCCTGGACTGGAC | 7490 |
| OVCAR5 | GGATGGGGCAGCCACCAAAGTGGATGCCATCTGCACCTACCGCCCTGATCCCAAAAGCCCTGGACTGGAC | 7490 |
| OV1 | GGATGGGGCAGCCACCAAAGTGGATGCCATCTGCACCTACCGCCCTGATCCCAAAAGCCCTGGACTGGAC | 7490 |
| OV2 | GGATGGGGCAGCCACCAAAGTGGATGCCATCTGCACCTACCGCCCTGATCCCAAAAGCCCTGGACTGGAC | 7490 |
| OV3 | GGATGGGGCAGCCACCAAAGTGGATGCCATCTGCACCTACCGCCCTGATCCCAAAAGCCCTGGACTGGAC | 7490 |

| | | |
|---|---|---|
| **Consensus** | **AGAGAGCAGCTATACTGGGAGCTGAGCCAGCTAACCCACAGCATCACTGAGCTGGGCCCCTACACCCTGG** | |
| NM_001414687.1 (35,819 .. 46,231) | AGAGAGCAGCTATACTGGGAGCTGAGCCAGCTGACCCACAGCATCACTGAGCTGGGCCCCTACACCCTGG | 7560 |
| Kuramochi | AGAGAGCAGCTATACTGGGAGCTGAGCCAGCTAACCCACAGCATCACTGAGCTGGGCCCCTACACCCTGG | 7560 |
| OVCAR3 | AGAGAGCAGCTATACTGGGAGCTGAGCCAGCTAACCCACAGCATCACTGAGCTGGGCCCCTACACCCTGG | 7560 |
| OVCAR5 | AGAGAGCAGCTATACTGGGAGCTGAGCCAGCTAACCCACAGCATCACTGAGCTGGGCCCCTACACCCTGG | 7560 |
| OV1 | AGAGAGCAGCTATACTGGGAGCTGAGCCAGCTAACCCACAGCATCACTGAGCTGGGCCCCTACACCCTGG | 7560 |
| OV2 | AGAGAGCAGCTATACTGGGAGCTGAGCCAGCTAACCCACAGCATCACTGAGCTGGGCCCCTACACCCTGG | 7560 |
| OV3 | AGAGAGCAGCTATACTGGGAGCTGAGCCAGCTGACCCACAGCATCACTGAGCTGGGCCCCTACACCCTGG | 7560 |

| | | |
|---|---|---|
| **Consensus** | **ACAGGGACAGTCTCTATGTCAATGGTTTCACACAGCGGAGCTCTGTGCCCACCACTAGCATTCCTGGGAC** | |
| NM_001414687.1 (35,819 .. 46,231) | ACAGGGACAGTCTCTATGTCAATGGTTTCACACAGCGGAGCTCTGTGCCCACCACTAGCATTCCTGGGAC | 7630 |
| Kuramochi | ACAGGGACAGTCTCTATGTCAATGGTTTCACACAGCGGAGCTCTGTGCCCACCACTAGCATTCCTGGGAC | 7630 |
| OVCAR3 | ACAGGGACAGTCTCTATGTCAATGGTTTCACACAGCGGAGCTCTGTGCCCACCACTAGCATTCCTGGGAC | 7630 |
| OVCAR5 | ACAGGGACAGTCTCTATGTCAATGGTTTCACACAGCGGAGCTCTGTGCCCACCACTAGCATTCCTGGGAC | 7630 |
| OV1 | ACAGGGACAGTCTCTATGTCAATGGTTTCACACAGCGGAGCTCTGTGCCCACCACTAGCATTCCTGGGAC | 7630 |
| OV2 | ACAGGGACAGTCTCTATGTCAATGGTTTCACACAGCGGAGCTCTGTGCCCACCACTAGCATTCCTGGGAC | 7630 |
| OV3 | ACAGGGACAGTCTCTATGTCAATGGTTTCACACAGCGGAGCTCTGTGCCCACCACTAGCATTCCTGGGAC | 7630 |

| | | |
|---|---|---|
| **Consensus** | **CCCCACAGTGGACCTGGGAACATCTGGGACTCCAGTTTCTAAACCTGGTCCCTCGGCTGCCAGCCCTCTC** | |
| NM_001414687.1 (35,819 .. 46,231) | CCCCACAGTGGACCTGGGAACATCTGGGACTCCAGTTTCTAAACCTGGTCCCTCGGCTGCCAGCCCTCTC | 7700 |
| Kuramochi | CCCCACAGTGGACCTGGGAACATCTGGGACTCCAGTTTCTAAACCTGGTCCCTCGGCTGCCAGCCCTCTC | 7700 |
| OVCAR3 | CCCCACAGTGGACCTGGGAACATCTGGGACTCCAGTTTCTAAACCTGGTCCCTCGGCTGCCAGCCCTCTC | 7700 |
| OVCAR5 | CCCCACAGTGGACCTGGGAACATCTGGGACTCCAGTTTCTAAACCTGGTCCCTCGGCTGCCAGCCCTCTC | 7700 |
| OV1 | CCCCACAGTGGACCTGGGAACATCTGGGACTCCAGTTTCTAAACCTGGTCCCTCGGCTGCCAGCCCTCTC | 7700 |
| OV2 | CCCCACAGTGGACCTGGGAACATCTGGGACTCCAGTTTCTAAACCTGGTCCCTCGGCTGCCAGCCCTCTC | 7700 |
| OV3 | CCCCACAGTGGACCTGGGAACATCTGGGACTCCAGTTTCTAAACCTGGTCCCTCGGCTGCCAGCCCTCTC | 7700 |

| | | |
|---|---|---|
| **Consensus** | **CTGGTGCTATTCACTCTCAACTTCACCATCACCAACCTGCGGTATGAGGAGAACATGCAGCACCCTGGCT** | |
| NM_001414687.1 (35,819 .. 46,231) | CTGGTGCTATTCACTCTCAACTTCACCATCACCAACCTGCGGTATGAGGAGAACATGCAGCACCCTGGCT | 7770 |
| Kuramochi | CTGGTGCTATTCACTCTCAACTTCACCATCACCAACCTGCGGTATGAGGAGAACATGCAGCACCCTGGCT | 7770 |
| OVCAR3 | CTGGTGCTATTCACTCTCAACTTCACCATCACCAACCTGCGGTATGAGGAGAACATGCAGCACCCTGGCT | 7770 |
| OVCAR5 | CTGGTGCTATTCACTCTCAACTTCACCATCACCAACCTGCGGTATGAGGAGAACATGCAGCACCCTGGCT | 7770 |
| OV1 | CTGGTGCTATTCACTCTCAACTTCACCATCACCAACCTGCGGTATGAGGAGAACATGCAGCACCCTGGCT | 7770 |
| OV2 | CTGGTGCTATTCACTCTCAACTTCACCATCACCAACCTGCGGTATGAGGAGAACATGCAGCACCCTGGCT | 7770 |
| OV3 | CTGGTGCTATTCACTCTCAACTTCACCATCACCAACCTGCGGTATGAGGAGAACATGCAGCACCCTGGCT | 7770 |

| | | |
|---|---|---|
| **Consensus** | **CCAGGAAGTTCAACACCACGGAGAGGGTCCTTCAGGGCCTGCTCAGGTCCCTGTTCAAGAGCACCAGTGT** | |
| NM_001414687.1 (35,819 .. 46,231) | CCAGGAAGTTCAACACCACGGAGAGGGTCCTTCAGGGCCTGCTCAGGTCCCTGTTCAAGAGCACCAGTGT | 7840 |
| Kuramochi | CCAGGAAGTTCAACACCACGGAGAGGGTCCTTCAGGGCCTGCTCAGGTCCCTGTTCAAGAGCACCAGTGT | 7840 |
| OVCAR3 | CCAGGAAGTTCAACACCACGGAGAGGGTCCTTCAGGGCCTGCTCAGGTCCCTGTTCAAGAGCACCAGTGT | 7840 |
| OVCAR5 | CCAGGAAGTTCAACACCACGGAGAGGGTCCTTCAGGGCCTGCTCAGGTCCCTGTTCAAGAGCACCAGTGT | 7840 |
| OV1 | CCAGGAAGTTCAACACCACGGAGAGGGTCCTTCAGGGCCTGCTCAGGTCCCTGTTCAAGAGCACCAGTGT | 7840 |
| OV2 | CCAGGAAGTTCAACACCACGGAGAGGGTCCTTCAGGGCCTGCTCAGGTCCCTGTTCAAGAGCACCAGTGT | 7840 |
| OV3 | CCAGGAAGTTCAACACCACGGAGAGGGTCCTTCAGGGCCTGCTCAGGTCCCTGTTCAAGAGCACCAGTGT | 7840 |

| | | |
|---|---|---|
| **Consensus** | **TGGCCCTCTGTACTCTGGCTGCAGACTGACTTTGCTCAGGCCTGAAAAGGATGGGACAGCCACTGGAGTG** | |
| NM_001414687.1 (35,819 .. 46,231) | TGGCCCTCTGTACTCTGGCTGCAGACTGACTTTGCTCAGGCCTGAAAAGGATGGGACAGCCACTGGAGTG | 7910 |
| Kuramochi | TGGCCCTCTGTACTCTGGCTGCAGACTGACTTTGCTCAGGCCTGAAAAGGATGGGACAGCCACTGGAGTG | 7910 |
| OVCAR3 | TGGCCCTCTGTACTCTGGCTGCAGACTGACTTTGCTCAGGCCTGAAAAGGATGGGACAGCCACTGGAGTG | 7910 |
| OVCAR5 | TGGCCCTCTGTACTCTGGCTGCAGACTGACTTTGCTCAGGCCTGAAAAGGATGGGACAGCCACTGGAGTG | 7910 |
| OV1 | TGGCCCTCTGTACTCTGGCTGCAGACTGACTTTGCTCAGGCCTGAAAAGGATGGGACAGCCACTGGAGTG | 7910 |
| OV2 | TGGCCCTCTGTACTCTGGCTGCAGACTGACTTTGCTCAGGCCTGAAAAGGATGGGACAGCCACTGGAGTG | 7910 |
| OV3 | TGGCCCTCTGTACTCTGGCTGCAGACTGACTTTGCTCAGGCCTGAAAAGGATGGGACAGCCACTGGAGTG | 7910 |

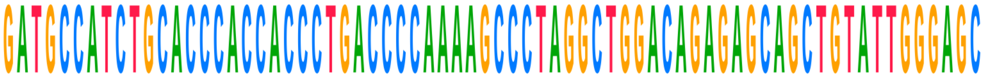

| | | |
|---|---|---|
| **Consensus** | **GATGCCATCTGCACCCACCACCCTGACCCCAAAAGCCCTAGGCTGGACAGAGAGCAGCTGTATTGGGAGC** | |
| NM_001414687.1 (35,819 .. 46,231) | GATGCCATCTGCACCCACCACCCTGACCCCAAAAGCCCTAGGCTGGACAGAGAGCAGCTGTATTGGGAGC | 7980 |
| Kuramochi | GATGCCATCTGCACCCACCACCCTGACCCCAAAAGCCCTAGGCTGGACAGAGAGCAGCTGTATTGGGAGC | 7980 |
| OVCAR3 | GATGCCATCTGCACCCACCACCCTGACCCCAAAAGCCCTAGGCTGGACAGAGAGCAGCTGTATTGGGAGC | 7980 |
| OVCAR5 | GATGCCATCTGCACCCACCACCCTGACCCCAAAAGCCCTAGGCTGGACAGAGAGCAGCTGTATTGGGAGC | 7980 |
| OV1 | GATGCCATCTGCACCCACCACCCTGACCCCAAAAGCCCTAGGCTGGACAGAGAGCAGCTGTATTGGGAGC | 7980 |
| OV2 | GATGCCATCTGCACCCACCACCCTGACCCCAAAAGCCCTAGGCTGGACAGAGAGCAGCTGTATTGGGAGC | 7980 |
| OV3 | GATGCCATCTGCACCCACCACCCTGACCCCAAAAGCCCTAGGCTGGACAGAGAGCAGCTGTATTGGGAGC | 7980 |

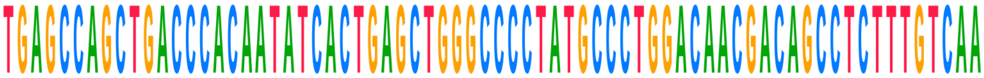

| | | |
|---|---|---|
| **Consensus** | **TGAGCCAGCTGACCCACAATATCACTGAGCTGGGCCCCTATGCCCTGGACAACGACAGCCTCTTTGTCAA** | |
| NM_001414687.1 (35,819 .. 46,231) | TGAGCCAGCTGACCCACAATATCACTGAGCTGGGCCCCTATGCCCTGGACAACGACAGCCTCTTTGTCAA | 8050 |
| Kuramochi | TGAGCCAGCTGACCCACAATATCACTGAGCTGGGCCCCTATGCCCTGGACAACGACAGCCTCTTTGTCAA | 8050 |
| OVCAR3 | TGAGCCAGCTGACCCACAATATCACTGAGCTGGGCCCCTATGCCCTGGACAACGACAGCCTCTTTGTCAA | 8050 |
| OVCAR5 | TGAGCCAGCTGACCCACAATATCACTGAGCTGGGCCCCTATGCCCTGGACAACGACAGCCTCTTTGTCAA | 8050 |
| OV1 | TGAGCCAGCTGACCCACAATATCACTGAGCTGGGCCCCTATGCCCTGGACAACGACAGCCTCTTTGTCAA | 8050 |
| OV2 | TGAGCCAGCTGACCCACAATATCACTGAGCTGGGCCCCTATGCCCTGGACAACGACAGCCTCTTTGTCAA | 8050 |
| OV3 | TGAGCCAGCTGACCCACAATATCACTGAGCTGGGCCCCTATGCCCTGGACAACGACAGCCTCTTTGTCAA | 8050 |

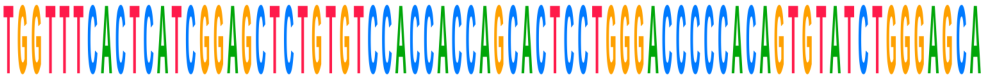

| | | |
|---|---|---|
| **Consensus** | **TGGTTTCACTCATCGGAGCTCTGTGTCCACCACCAGCACTCCTGGGACCCCCACAGTGTATCTGGGAGCA** | |
| NM_001414687.1 (35,819 .. 46,231) | TGGTTTCACTCATCGGAGCTCTGTGTCCACCACCAGCACTCCTGGGACCCCCACAGTGTATCTGGGAGCA | 8120 |
| Kuramochi | TGGTTTCACTCATCGGAGCTCTGTGTCCACCACCAGCACTCCTGGGACCCCCACAGTGTATCTGGGAGCA | 8120 |
| OVCAR3 | TGGTTTCACTCATCGGAGCTCTGTGTCCACCACCAGCACTCCTGGGACCCCCACAGTGTATCTGGGAGCA | 8120 |
| OVCAR5 | TGGTTTCACTCATCGGAGCTCTGTGTCCACCACCAGCACTCCTGGGACCCCCACAGTGTATCTGGGAGCA | 8120 |
| OV1 | TGGTTTCACTCATCGGAGCTCTGTGTCCACCACCAGCACTCCTGGGACCCCCACAGTGTATCTGGGAGCA | 8120 |
| OV2 | TGGTTTCACTCATCGGAGCTCTGTGTCCACCACCAGCACTCCTGGGACCCCCACAGTGTATCTGGGAGCA | 8120 |
| OV3 | TGGTTTCACTCATCGGAGCTCTGTGTCCACCACCAGCACTCCTGGGACCCCCACAGTGTATCTGGGAGCA | 8120 |

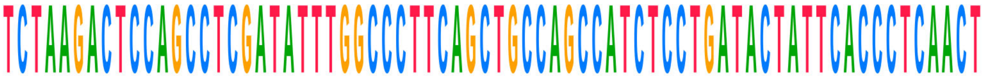

| | | |
|---|---|---|
| **Consensus** | **TCTAAGACTCCAGCCTCGATATTTGGCCCTTCAGCTGCCAGCCATCTCCTGATACTATTCACCCTCAACT** | |
| NM_001414687.1 (35,819 .. 46,231) | TCTAAGACTCCAGCCTCGATATTTGGCCCTTCAGCTGCCAGCCATCTCCTGATACTATTCACCCTCAACT | 8190 |
| Kuramochi | TCTAAGACTCCAGCCTCGATATTTGGCCCTTCAGCTGCCAGCCATCTCCTGATACTATTCACCCTCAACT | 8190 |
| OVCAR3 | TCTAAGACTCCAGCCTCGATATTTGGCCCTTCAGCTGCCAGCCATCTCCTGATACTATTCACCCTCAACT | 8190 |
| OVCAR5 | TCTAAGACTCCAGCCTCGATATTTGGCCCTTCAGCTGCCAGCCATCTCCTGATACTATTCACCCTCAACT | 8190 |
| OV1 | TCTAAGACTCCAGCCTCGATATTTGGCCCTTCAGCTGCCAGCCATCTCCTGATACTATTCACCCTCAACT | 8190 |
| OV2 | TCTAAGACTCCAGCCTCGATATTTGGCCCTTCAGCTGCCAGCCATCTCCTGATACTATTCACCCTCAACT | 8190 |
| OV3 | TCTAAGACTCCAGCCTCGATATTTGGCCCTTCAGCTGCCAGCCATCTCCTGATACTATTCACCCTCAACT | 8190 |

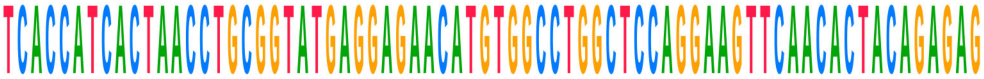

| | | |
|---|---|---|
| **Consensus** | **TCACCATCACTAACCTGCGGTATGAGGAGAACATGTGGCCTGGCTCCAGGAAGTTCAACACTACAGAGAG** | |
| NM_001414687.1 (35,819 .. 46,231) | TCACCATCACTAACCTGCGGTATGAGGAGAACATGTGGCCTGGCTCCAGGAAGTTCAACACTACAGAGAG | 8260 |
| Kuramochi | TCACCATCACTAACCTGCGGTATGAGGAGAACATGTGGCCTGGCTCCAGGAAGTTCAACACTACAGAGAG | 8260 |
| OVCAR3 | TCACCATCACTAACCTGCGGTATGAGGAGAACATGTGGCCTGGCTCCAGGAAGTTCAACACTACAGAGAG | 8260 |
| OVCAR5 | TCACCATCACTAACCTGCGGTATGAGGAGAACATGTGGCCTGGCTCCAGGAAGTTCAACACTACAGAGAG | 8260 |
| OV1 | TCACCATCACTAACCTGCGGTATGAGGAGAACATGTGGCCTGGCTCCAGGAAGTTCAACACTACAGAGAG | 8260 |
| OV2 | TCACCATCACTAACCTGCGGTATGAGGAGAACATGTGGCCTGGCTCCAGGAAGTTCAACACTACAGAGAG | 8260 |
| OV3 | TCACCATCACTAACCTGCGGTATGAGGAGAACATGTGGCCTGGCTCCAGGAAGTTCAACACTACAGAGAG | 8260 |

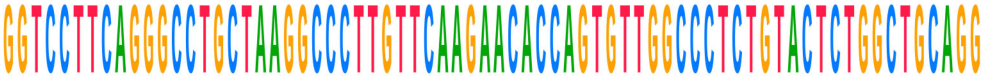

| | | |
|---|---|---|
| **Consensus** | **GGTCCTTCAGGGCCTGCTAAGGCCCTTGTTCAAGAACACCAGTGTTGGCCCTCTGTACTCTGGCTGCAGG** | |
| NM_001414687.1 (35,819 .. 46,231) | GGTCCTTCAGGGCCTGCTAAGGCCCTTGTTCAAGAACACCAGTGTTGGCCCTCTGTACTCTGGCTGCAGG | 8330 |
| Kuramochi | GGTCCTTCAGGGCCTGCTAAGGCCCTTGTTCAAGAACACCAGTGTTGGCCCTCTGTACTCTGGCTGCAGG | 8330 |
| OVCAR3 | GGTCCTTCAGGGCCTGCTAAGGCCCTTGTTCAAGAACACCAGTGTTGGCCCTCTGTACTCTGGCTGCAGG | 8330 |
| OVCAR5 | GGTCCTTCAGGGCCTGCTAAGGCCCTTGTTCAAGAACACCAGTGTTGGCCCTCTGTACTCTGGCTGCAGG | 8330 |
| OV1 | GGTCCTTCAGGGCCTGCTAAGGCCCTTGTTCAAGAACACCAGTGTTGGCCCTCTGTACTCTGGCTGCAGG | 8330 |
| OV2 | GGTCCTTCAGGGCCTGCTAAGGCCCTTGTTCAAGAACACCAGTGTTGGCCCTCTGTACTCTGGCTGCAGG | 8330 |
| OV3 | GGTCCTTCAGGGCCTGCTAAGGCCCTTGTTCAAGAACACCAGTGTTGGCCCTCTGTACTCTGGCTGCAGG | 8330 |

| | | |
|---|---|---|
| **Consensus** | **CTGACCTTGCTCAGGCCAGAGAAAGATGGGGAAGCCACCGGAGTGGATGCCATCTGCACCCACCGCCCTG** | |
| NM_001414687.1 (35,819 .. 46,231) | CTGACCTTGCTCAGGCCAGAGAAAGATGGGGAAGCCACCGGAGTGGATGCCATCTGCACCCACCGCCCTG | 8400 |
| Kuramochi | CTGACCTTGCTCAGGCCAGAGAAAGATGGGGAAGCCACCGGAGTGGATGCCATCTGCACCCACCGCCCTG | 8400 |
| OVCAR3 | CTGACCTTGCTCAGGCCAGAGAAAGATGGGGAAGCCACCGGAGTGGATGCCATCTGCACCCACCGCCCTG | 8400 |
| OVCAR5 | CTGACCTTGCTCAGGCCAGAGAAAGATGGGGAAGCCACCGGAGTGGATGCCATCTGCACCCACCGCCCTG | 8400 |
| OV1 | CTGACCTTGCTCAGGCCAGAGAAAGATGGGGAAGCCACCGGAGTGGATGCCATCTGCACCCACCGCCCTG | 8400 |
| OV2 | CTGACCTTGCTCAGGCCAGAGAAAGATGGGGAAGCCACCGGAGTGGATGCCATCTGCACCCACCGCACTG | 8400 |
| OV3 | CTGACCTTGCTCAGGCCAGAGAAAGATGGGGAAGCCACCGGAGTGGATGCCATCTGCACCCACCGCCCTG | 8400 |

| | | |
|---|---|---|
| **Consensus** | **ACCCCACAGGCCCTGGGCTGGACAGAGAGCAGCTGTATTTGGAGCTGAGCCAGCTGACCCACAGCATCAC** | |
| NM_001414687.1 (35,819 .. 46,231) | ACCCCACAGGCCCTGGGCTGGACAGAGAGCAGCTGTATTTGGAGCTGAGCCAGCTGACCCACAGCATCAC | 8470 |
| Kuramochi | ACCCCACAGGCCCTGGGCTGGACAGAGAGCAGCTGTATTTGGAGCTGAGCCAGCTGACCCACAGCATCAC | 8470 |
| OVCAR3 | ACCCCACAGGCCCTGGGCTGGACAGAGAGCAGCTGTATTTGGAGCTGAGCCAGCTGACCCACAGCATCAC | 8470 |
| OVCAR5 | ACCCCACAGGCCCTGGGCTGGACAGAGAGCAGCTGTATTTGGAGCTGAGCCAGCTGACCCACAGCATCAC | 8470 |
| OV1 | ACCCCACAGGCCCTGGGCTGGACAGAGAGCAGCTGTATTTGGAGCTGAGCCAGCTGACCCACAGCATCAC | 8470 |
| OV2 | ACCCCACAGGCCCTGGGCTGGACAGAGAGCAGCTGTATTTGGAGCTGAGCCAGCTGACCCACAGCATCAC | 8470 |
| OV3 | ACCCCACAGGCCCTGGGCTGGACAGAGAGCAGCTGTATTTGGAGCTGAGCCAGCTGACCCACAGCATCAC | 8470 |

| | | |
|---|---|---|
| **Consensus** | **TGAGCTGGGCCCCTACACACTGGACAGGGACAGTCTCTATGTCAATGGTTTCACCCATCGGAGCTCTGTA** | |
| NM_001414687.1 (35,819 .. 46,231) | TGAGCTGGGCCCCTACACACTGGACAGGGACAGTCTCTATGTCAATGGTTTCACCCATCGGAGCTCTGTA | 8540 |
| Kuramochi | TGAGCTGGGCCCCTACACACTGGACAGGGACAGTCTCTATGTCAATGGTTTCACCCATCGGAGCTCTGTA | 8540 |
| OVCAR3 | TGAGCTGGGCCCCTACACACTGGACAGGGACAGTCTCTATGTCAATGGTTTCACCCATCGGAGCTCTGTA | 8540 |
| OVCAR5 | TGAGCTGGGCCCCTACACACTGGACAGGGACAGTCTCTATGTCAATGGTTTCACCCATCGGAGCTCTGTA | 8540 |
| OV1 | TGAGCTGGGCCCCTACACACTGGACAGGGACAGTCTCTATGTCAATGGTTTCACCCATCGGAGCTCTGTA | 8540 |
| OV2 | TGAGCTGGGCCCCTACACACTGGACAGGGACAGTCTCTATGTCAATGGTTTCACCCATCGGAGCTCTGTA | 8540 |
| OV3 | TGAGCTGGGCCCCTACACACTGGACAGGGACAGTCTCTATGTCAATGGTTTCACCCATCGGAGCTCTGTA | 8540 |

| | | |
|---|---|---|
| **Consensus** | **CCCACCACCAGCACCGGGGTGGTCAGCGAGGAGCCATTCACACTGAACTTCACCATCAACAACCTGCGCT** | |
| NM_001414687.1 (35,819 .. 46,231) | CCCACCACCAGCACCGGGGTGGTCAGCGAGGAGCCATTCACACTGAACTTCACCATCAACAACCTGCGCT | 8610 |
| Kuramochi | CCCACCACCAGCACCGGGGTGGTCAGCGAGGAGCCATTCACACTGAACTTCACCATCAACAACCTGCGCT | 8610 |
| OVCAR3 | CCCACCACCAGCACCGGGGTGGTCAGCGAGGAGCCATTCACACTGAACTTCACCATCAACAACCTGCGCT | 8610 |
| OVCAR5 | CCCACCACCAGCACCGGGGTGGTCAGCGAGGAGCCATTCACACTGAACTTCACCATCAACAACCTGCGCT | 8610 |
| OV1 | CCCACCACCAGCACCGGGGTGGTCAGCGAGGAGCCATTCACACTGAACTTCACCATCAACAACCTGCGCT | 8610 |
| OV2 | CCCACCACCAGCACCGGGGTGGTCAGCGAGGAGCCATTCACACTGAACTTCACCATCAACAACCTGCGCT | 8610 |
| OV3 | CCCACCACCAGCACCGGGGTGGTCAGCGAGGAGCCATTCACACTGAACTTCACCATCAACAACCTGCGCT | 8610 |

| | | |
|---|---|---|
| **Consensus** | **ACATGGCGGACATGGGCCAACCCGGCTCCCTCAAGTTCAACATCACAGACAACGTCATGCAGCACCTGCT** | |
| NM_001414687.1 (35,819 .. 46,231) | ACATGGCGGACATGGGCCAACCCGGCTCCCTCAAGTTCAACATCACAGACAACGTCATGCAGCACCTGCT | 8680 |
| Kuramochi | ACATGGCGGACATGGGCCAACCCGGCTCCCTCAAGTTCAACATCACAGACAACGTCATGCAGCACCTGCT | 8680 |
| OVCAR3 | ACATGGCGGACATGGGCCAACCCGGCTCCCTCAAGTTCAACATCACAGACAACGTCATGCAGCACCTGCT | 8680 |
| OVCAR5 | ACATGGCGGACATGGGCCAACCCGGCTCCCTCAAGTTCAACATCACAGACAACGTCATGCAGCACCTGCT | 8680 |
| OV1 | ACATGGCGGACATGGGCCAACCCGGCTCCCTCAAGTTCAACATCACAGACAACGTCATGCAGCACCTGCT | 8680 |
| OV2 | ACATGGCGGACATGGGCCAACCCGGCTCCCTCAAGTTCAACATCACAGACAACGTCATGCAGCACCTGCT | 8680 |
| OV3 | ACATGGCGGACATGGGCCAACCCGGCTCCCTCAAGTTCAACATCACAGACAACGTCATGAAGCACCTGCT | 8680 |

| | | |
|---|---|---|
| **Consensus** | **CAGTCCTTTGTTCCAGAGGAGCAGCCTGGGTGCACGGTACACAGGCTGCAGGGTCATCGCACTAAGGTCT** | |
| NM_001414687.1 (35,819 .. 46,231) | CAGTCCTTTGTTCCAGAGGAGCAGCCTGGGTGCACGGTACACAGGCTGCAGGGTCATCGCACTAAGGTCT | 8750 |
| Kuramochi | CAGTCCTTTGTTCCAGAGGAGCAGCCTGGGTGCACGGTACACAGGCTGCAGGGTCATCGCACTAAGGTCT | 8750 |
| OVCAR3 | CAGTCCTTTGTTCCAGAGGAGCAGCCTGGGTGCACGGTACACAGGCTGCAGGGTCATCGCACTAAGGTCT | 8750 |
| OVCAR5 | CAGTCCTTTGTTCCAGAGGAGCAGCCTGGGTGCACGGTACACAGGCTGCAGGGTCATCGCACTAAGGTCT | 8750 |
| OV1 | CAGTCCTTTGTTCCAGAGGAGCAGCCTGGGTGCACGGTACACAGGCTGCAGGGTCATCGCACTAAGGTCT | 8750 |
| OV2 | CAGTCCTTTGTTCCAGAGGAGCAGCCTGGGTGCACGGTACACAGGCTGCAGGGTCATCGCACTAAGGTCT | 8750 |
| OV3 | CAGTCCTTTGTTCCAGAGGAGCAGCCTGGGTGCACGGTACACAGGCTGCAGGGTCATCGCACTAAGGTCT | 8750 |

| | | |
|---|---|---|
| **Consensus** | **GTGAAGAACGGTGCTGAGACACGGGTGGACCTCCTCTGCACCTACCTGCAGCCCCTCAGCGGCCCAGGTC** | |
| NM_001414687.1 (35,819 .. 46,231) | GTGAAGAACGGTGCTGAGACACGGGTGGACCTCCTCTGCACCTACCTGCAGCCCCTCAGCGGCCCAGGTC | 8820 |
| Kuramochi | GTGAAGAACGGTGCTGAGACACGGGTGGACCTCCTCTGCACCTACCTGCAGCCCCTCAGCGGCCCAGGTC | 8820 |
| OVCAR3 | ATGAAGAACGGTGCTGAGACACGGGTGGACCTCCTCTGCACCTACCTGCAGCCCCTCAGCGGCCCAGGTC | 8820 |
| OVCAR5 | GTGAAGAACGGTGCTGAGACACGGGTGGACCTCCTCTGCACCTACCTGCAGCCCCTCAGCGGCCCAGGTC | 8820 |
| OV1 | GTGAAGAACGGTGCTGAGACACGGGTGGACCTCCTCTGCACCTACCTGCAGCCCCTCAGCGGCCCAGGTC | 8820 |
| OV2 | GTGAAGAACGGTGCTGAGACACGGGTGGACCTCCTCTGCACCTACCTGCAGCCCCTCAGCGGCCCAGGTC | 8820 |
| OV3 | GTGAAGAACGGTGCTGAGACACGGGTGGACCTCCTCTGCACCTACCTGCAGCCCCTCAGCGGCCCAGGTC | 8820 |

| | | |
|---|---|---|
| **Consensus** | **TGCCTATCAAGCAGGTGTTCCATGAGCTGAGCCAGCAGACCCATGGCATCACCCGGCTGGGCCCCTACTC** | |
| NM_001414687.1 (35,819 .. 46,231) | TGCCTATCAAGCAGGTGTTCCATGAGCTGAGCCAGCAGACCCATGGCATCACCCGGCTGGGCCCCTACTC | 8890 |
| Kuramochi | TGCCTATCAAGCAGGTGTTCCATGAGCTGAGCCAGCAGACCCATGGCATCACCCGGCTGGGCCCCTACTC | 8890 |
| OVCAR3 | TGCCTATCAAGCAGGTGTTCCATGAGCTGAGCCAGCAGACCCATGGCATCACCCGGCTGGGCCCCTACTC | 8890 |
| OVCAR5 | TGCCTATCAAGCAGGTGTTCCATGAGCTGAGCCAGCAGACCCATGGCATCACCCGGCTGGGCCCCTACTC | 8890 |
| OV1 | TGCCTATCAAGCAGGTGTTCCATGAGCTGAGCCAGCAGACCCATGGCATCACCCGGCTGGGCCCCTACTC | 8890 |
| OV2 | TGCCTATCAAGCAGGTGTTCCATGAGCTGAGCCAGCAGACCCATGGCATCACCCGGCTGGGCCCCTACTC | 8890 |
| OV3 | TGCCTATCAAGCAGGTGTTCCATGAGCTGAGCCAGCAGACCCATGGCATCACCCGGCTGGGCCCCTACTC | 8890 |

| | | |
|---|---|---|
| **Consensus** | **TCTGGACAAAGACAGCCTCTACCTTAACGGTTACAATGAACCTGGTCCAGATGAGCCTCCTACAACTCCC** | |
| NM_001414687.1 (35,819 .. 46,231) | TCTGGACAAAGACAGCCTCTACCTTAACGGTTACAATGAACCTGGTCCAGATGAGCCTCCTACAACTCCC | 8960 |
| Kuramochi | TCTGGACAAAGACAGCCTCTACCTTAACGGTTACAATGAACCTGGTCCAGATGAGCCTCCTACAACTCCC | 8960 |
| OVCAR3 | TCTGGACAAAGACAGCCTCTACCTTAACGGTTACAATGAACCTGGTCCAGATGAGCCTCCTACAACTCCC | 8960 |
| OVCAR5 | TCTGGACAAAGACAGCCTCTACCTTAACGGTTACAATGAACCTGGTCCAGATGAGCCTCCTACAACTCCC | 8960 |
| OV1 | TCTGGACAAAGACAGCCTCTACCTTAACGGTTACAATGAACCTGGTCCAGATGAGCCTCCTACAACTCCC | 8960 |
| OV2 | TCTGGACAAAGACAGCCTCTACCTTAACGGTTACAATGAACCTGGTCCAGATGAGCCTCCTACAACTCCC | 8960 |
| OV3 | TCTGGACAAAGACAGCCTCTACCTTAACGGTTACAATGAACCTGGTCCAGATGAGCCTCCTACAACTCCC | 8960 |

| | | |
|---|---|---|
| **Consensus** | **AAGCCAGCCACCACATTCCTGCCTCCTCTGTCAGAAGCCACAACAGCCATGGGGTACCACCTGAAGACCC** | |
| NM_001414687.1 (35,819 .. 46,231) | AAGCCAGCCACCACATTCCTGCCTCCTCTGTCAGAAGCCACAACAGCCATGGGGTACCACCTGAAGACCC | 9030 |
| Kuramochi | AAGCCAGCCACCACATTCCTGCCTCCTCTGTCAGAAGCCACAACAGCCATGGGGTACCACCTGAAGACCC | 9030 |
| OVCAR3 | AAGCCAGCCACCACATTCCTGCCTCCTCTGTCAGAAGCCACAACAGCCATGGGGTACCACCTGAAGACCC | 9030 |
| OVCAR5 | AAGCCAGCCACCACATTCCTGCCTCCTCTGTCAGAAGCCACAACAGCCATGGGGTACCACCTGAAGACCC | 9030 |
| OV1 | AAGCCAGCCACCACATTCCTGCCTCCTCTGTCAGAAGCCACAACAGCCATGGGGTACCACCTGAAGACCC | 9030 |
| OV2 | AAGCCAGCCACCACATTCCTGCCTCCTCTGTCAGAAGCCACAACAGCCATGGGGTACCACCTGAAGACCC | 9030 |
| OV3 | AAGCCAGCCACCACATTCCTGCCTCCTCTGTCAGAAGCCACAACAGCCATGGGGTACCACCTGAAGACCC | 9030 |

| | | |
|---|---|---|
| **Consensus** | **TCACACTCAACTTCACCATCTCCAATCTCCAGTATTCACCAGATATGGGCAAGGGCTCAGCTACATTCAA** | |
| NM_001414687.1 (35,819 .. 46,231) | TCACACTCAACTTCACCATCTCCAATCTCCAGTATTCACCAGATATGGGCAAGGGCTCAGCTACATTCAA | 9100 |
| Kuramochi | TCACACTCAACTTCACCATCTCCAATCTCCAGTATTCACCAGATATGGGCAAGGGCTCAGCTACATTCAA | 9100 |
| OVCAR3 | TCACACTCAACTTCACCATCTCCAATCTCCAGTATTCACCAGATATGGGCAAGGGCTCAGCTACATTCAA | 9100 |
| OVCAR5 | TCACACTCAACTTCACCATCTCCAATCTCCAGTATTCACCAGATATGGGCAAGGGCTCAGCTACATTCAA | 9100 |
| OV1 | TCACACTCAACTTCACCATCTCCAATCTCCAGTATTCACCAGATATGGGCAAGGGCTCAGCTACATTCAA | 9100 |
| OV2 | TCACACTCAACTTCACCATCTCCAATCTCCAGTATTCACCAGATATGGGCAAGGGCTCAGCTACATTCAA | 9100 |
| OV3 | TCACACTCAACTTCACCATCTCCAATCTCCAGTATTCACCAGATATGGGCAAGGGCTCAGCTACATTCAA | 9100 |

| | | |
|---|---|---|
| **Consensus** | **CTCCACCGAGGGGGTCCTTCAGCACCTGCTCAGACCCTTGTTCCAGAAGAGCAGCATGGGCCCCTTCTAC** | |
| NM_001414687.1 (35,819 .. 46,231) | CTCCACCGAGGGGGTCCTTCAGCACCTGCTCAGACCCTTGTTCCAGAAGAGCAGCATGGGCCCCTTCTAC | 9170 |
| Kuramochi | CTCCACCGAGGGGGTCCTTCAGCACCTGCTCAGACCCTTGTTCCAGAAGAGCAGCATGGGCCCCTTCTAC | 9170 |
| OVCAR3 | CTCCACCGAGGGGGTCCTTCAGCACCTGCTCAGACCCTTGTTCCAGAAGAGCAGCATGGGCCCCTTCTAC | 9170 |
| OVCAR5 | CTCCACCGAGGGGGTCCTTCAGCACCTGCTCAGACCCTTGTTCCAGAAGAGCAGCATGGGCCCCTTCTAC | 9170 |
| OV1 | CTCCACCGAGGGGGTCCTTCAGCACCTGCTCAGACCCTTGTTCCAGAAGAGCAGCATGGGCCCCTTCTAC | 9170 |
| OV2 | CTCCACCGAGGGGGTCCTTCAGCACCTGCTCAGACCCTTGTTCCAGAAGAGCAGCATGGGCCCCTTCTAC | 9170 |
| OV3 | CTCCACCGAGGGGGTCCTTCAGCACCTGCTCAGACCCTTGTTCCAGAAGAGCAGCATGGGCCCCTTCTAC | 9170 |

| | | |
|---|---|---|
| **Consensus** | **TTGGGTTGCCAACTGATCTCCCTCAGGCCTGAGAAGGATGGGGCAGCCACTGGTGTGGACACCACCTGCA** | |
| NM_001414687.1 (35,819 .. 46,231) | TTGGGTTGCCAACTGATCTCCCTCAGGCCTGAGAAGGATGGGGCAGCCACTGGTGTGGACACCACCTGCA | 9240 |
| Kuramochi | TTGGGTTGCCAACTGATCTCCCTCAGGCCTGAGAAGGATGGGGCAGCCACTGGTGTGGACACCACCTGCA | 9240 |
| OVCAR3 | TTGGGTTGCCAACTGATCTCCCTCAGGCCTGAGAAGGATGGGGCAGCCACTGGTGTGGACACCACCTGCA | 9240 |
| OVCAR5 | TTGGGTTGCCAACTGATCTCCCTCAGGCCTGAGAAGGATGGGGCAGCCACTGGTGTGGACACCACCTGCA | 9240 |
| OV1 | TTGGGTTGCCAACTGATCTCCCTCAGGCCTGAGAAGGATGGGGCAGCCACTGGTGTGGACACCACCTGCA | 9240 |
| OV2 | TTGGGTTGCCAACTGATCTCCCTCAGGCCTGAGAAGGATGGGGCAGCCACTGGTGTGGACACCACCTGCA | 9240 |
| OV3 | TTGGGTTGCCAACTGATCTCCCTCAGGCCTGAGAAGGATGGGGCAGCCACTGGTGTGGACACCACCTGCA | 9240 |

| | | |
|---|---|---|
| **Consensus** | **CCTACCACCCTGACCCTGTGGGCCCCGGGCTGGACATACAGCAGCTTTACTGGGAGCTGAGTCAGCTGAC** | |
| NM_001414687.1 (35,819 .. 46,231) | CCTACCACCCTGACCCTGTGGGCCCCGGGCTGGACATACAGCAGCTTTACTGGGAGCTGAGTCAGCTGAC | 9310 |
| Kuramochi | CCTACCACCCTGACCCTGTGGGCCCCGGGCTGGACATACAGCAGCTTTACTGGGAGCTGAGTCAGCTGAC | 9310 |
| OVCAR3 | CCTACCACCCTGACCCTGTGGGCCCCGGGCTGGACATACAGCAGCTTTACTGGGAGCTGAGTCAGCTGAC | 9310 |
| OVCAR5 | CCTACCACCCTGACCCTGTGGGCCCCGGGCTGGACATACAGCAGCTTTACTGGGAGCTGAGTCAGCTGAC | 9310 |
| OV1 | CCTACCACCCTGACCCTGTGGGCCCCGGGCTGGACATACAGCAGCTTTACTGGGAGCTGAGTCAGCTGAC | 9310 |
| OV2 | CCTACCACCCTGACCCTGTGGGCCCCGGGCTGGACATACAGCAGCTTTACTGGGAGCTGAGTCAGCTGAC | 9310 |
| OV3 | CCTACCACCCTGACCCTGTGGGCCCCGGGCTGGACATACAGCAGCTTTACTGGGAGCTGAGTCAGCTGAC | 9310 |

| | | |
|---|---|---|
| **Consensus** | **CCATGGTGTCACCCAACTGGGCTTCTATGTCCTGGACAGGGATAGCCTCTTCATCAATGGCTATGCACCC** | |
| NM_001414687.1 (35,819 .. 46,231) | CCATGGTGTCACCCAACTGGGCTTCTATGTCCTGGACAGGGATAGCCTCTTCATCAATGGCTATGCACCC | 9380 |
| Kuramochi | CCATGGTGTCACCCAACTGGGCTTCTATGTCCTGGACAGGGATAGCCTCTTCATCAATGGCTATGCACCC | 9380 |
| OVCAR3 | CCATGGTGTCACCCAACTGGGCTTCTATGTCCTGGACAGGGATAGCCTCTTCATCAATGGCTATGCACCC | 9380 |
| OVCAR5 | CCATGGTGTCACCCAACTGGGCTTCTATGTCCTGGACAGGGATAGCCTCTTCATCAATGGCTATGCACCC | 9380 |
| OV1 | CCATGGTGTCACCCAACTGGGCTTCTATGTCCTGGACAGGGATAGCCTCTTCATCAATGGCTATGCACCC | 9380 |
| OV2 | CCATGGTGTCACCCAACTGGGCTTCTATGTCCTGGACAGGGATAGCCTCTTCATCAATGGCTATGCACCC | 9380 |
| OV3 | CCATGGTGTCACCCAACTGGGCTTCTATGTCCTGGACAGGGATAGCCTCTTCATCAATGGCTATGCACCC | 9380 |

| | | |
|---|---|---|
| **Consensus** | **CAGAATTTATCAATCCGGGGCGAGTACCAGATAAATTTCCACATTGTCAACTGGAACCTCAGTAATCCAG** | |
| NM_001414687.1 (35,819 .. 46,231) | CAGAATTTATCAATCCGGGGCGAGTACCAGATAAATTTCCACATTGTCAACTGGAACCTCAGTAATCCAG | 9450 |
| Kuramochi | CAGAATTTATCAATCCGGGGCGAGTACCAGATAAATTTCCACATTGTCAACTGGAACCTCAGTAATCCAG | 9450 |
| OVCAR3 | CAGAATTTATCAATCCGGGGCGAGTACCAGATAAATTTCCACATTGTCAACTGGAACCTCAGTAATCCAG | 9450 |
| OVCAR5 | CAGAATTTATCAATCCGGGGCGAGTACCAGATAAATTTCCACATTGTCAACTGGAACCTCAGTAATCCAG | 9450 |
| OV1 | CAGAATTTATCAATCCGGGGCGAGTACCAGATAAATTTCCACATTGTCAACTGGAACCTCAGTAATCCAG | 9450 |
| OV2 | CAGAATTTATCAATCCGGGGCGAGTACCAGATAAATTTCCACATTGTCAACTGGAACCTCAGTAATCCAG | 9450 |
| OV3 | CAGAATTTATCAATCCGGGGCGAGTACCAGATAAATTTCCACATTGTCAACTGGAACCTCAGTAATCCAG | 9450 |

| | | |
|---|---|---|
| **Consensus** | **ACCCCACATCCTCAGAGTACATCACCCTGCTGAGGGACATCCAGGACAAGGTCACCACACTCTACAAAGG** | |
| NM_001414687.1 (35,819 .. 46,231) | ACCCCACATCCTCAGAGTACATCACCCTGCTGAGGGACATCCAGGACAAGGTCACCACACTCTACAAAGG | 9520 |
| Kuramochi | ACCCCACATCCTCAGAGTACATCACCCTGCTGAGGGACATCCAGGACAAGGTCACCACACTCTACAAAGG | 9520 |
| OVCAR3 | ACCCCACATCCTCAGAGTACATCACCCTGCTGAGGGACATCCAGGACAAGGTCACCACACTCTACAAAGG | 9520 |
| OVCAR5 | ACCCCACATCCTCAGAGTACATCACCCTGCTGAGGGACATCCAGGACAAGGTCACCACACTCTACAAAGG | 9520 |
| OV1 | ACCCCACATCCTCAGAGTACATCACCCTGCTGAGGGACATCCAGGACAAGGTCACCACACTCTACAAAGG | 9520 |
| OV2 | ACCCCACATCCTCAGAGTACATCACCCTGCTGAGGGACATCCAGGACAAGGTCACCACACTCTACAAAGG | 9520 |
| OV3 | ACCCCACATCCTCAGAGTACATCACCCTGCTGAGGGACATCCAGGACAAGGTCACCACACTCTACAAAGG | 9520 |

| | | |
|---|---|---|
| **Consensus** | **CAGTCAACTACATGACACATTCCGCTTCTGCCTGGTCACCAACTTGACGATGGACTCCGTGTTGGTCACT** | |
| NM_001414687.1 (35,819 .. 46,231) | CAGTCAACTACATGACACATTCCGCTTCTGCCTGGTCACCAACTTGACGATGGACTCCGTGTTGGTCACT | 9590 |
| Kuramochi | CAGTCAACTACATGACACATTCCGCTTCTGCCTGGTCACCAACTTGACGATGGACTCCGTGTTGGTCACT | 9590 |
| OVCAR3 | CAGTCAACTACATGACACATTCCGCTTCTGCCTGGTCACCAACTTGACGATGGACTCCGTGTTGGTCACT | 9590 |
| OVCAR5 | CAGTCAACTACATGACACATTCCGCTTCTGCCTGGTCACCAACTTGACGATGGACTCCGTGTTGGTCACT | 9590 |
| OV1 | CAGTCAACTACATGACACATTCCGCTTCTGCCTGGTCACCAACTTGACGATGGACTCCGTGTTGGTCACT | 9590 |
| OV2 | CAGTCAACTACATGACACATTCCGCTTCTGCCTGGTCACCAACTTGACGATGGACTCCGTGTTGGTCACT | 9590 |
| OV3 | CAGTCAACTACATGACACATTCCGCTTCTGCCTGGTCACCAACTTGACGATGGACTCCGTGTTGGTCACT | 9590 |

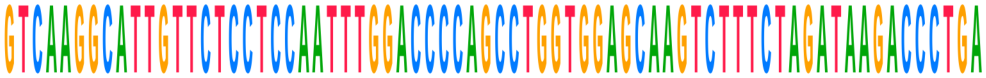

| | | |
|---|---|---|
| **Consensus** | **GTCAAGGCATTGTTCTCCTCCAATTTGGACCCCAGCCTGGTGGAGCAAGTCTTTCTAGATAAGACCCTGA** | |
| NM_001414687.1 (35,819 .. 46,231) | GTCAAGGCATTGTTCTCCTCCAATTTGGACCCCAGCCTGGTGGAGCAAGTCTTTCTAGATAAGACCCTGA | 9660 |
| Kuramochi | GTCAAGGCATTGTTCTCCTCCAATTTGGACCCCAGCCTGGTGGAGCAAGTCTTTCTAGATAAGACCCTGA | 9660 |
| OVCAR3 | GTCAAGGCATTGTTCTCCTCCAATTTGGACCCCAGCCTGGTGGAGCAAGTCTTTCTAGATAAGACCCTGA | 9660 |
| OVCAR5 | GTCAAGGCATTGTTCTCCTCCAATTTGGACCCCAGCCTGGTGGAGCAAGTCTTTCTAGATAAGACCCTGA | 9660 |
| OV1 | GTCAAGGCATTGTTCTCCTCCAATTTGGACCCCAGCCTGGTGGAGCAAGTCTTTCTAGATAAGACCCTGA | 9660 |
| OV2 | GTCAAGGCATTGTTCTCCTCCAATTTGGACCCCAGCCTGGTGGAGCAAGTCTTTCTAGATAAGACCCTGA | 9660 |
| OV3 | GTCAAGGCATTGTTCTCCTCCAATTTGGACCCCAGCCTGGTGGAGCAAGTCTTTCTAGATAAGACCCTGA | 9660 |

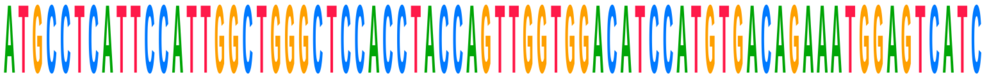

| | | |
|---|---|---|
| **Consensus** | **ATGCCTCATTCCATTGGCTGGGCTCCACCTACCAGTTGGTGGACATCCATGTGACAGAAATGGAGTCATC** | |
| NM_001414687.1 (35,819 .. 46,231) | ATGCCTCATTCCATTGGCTGGGCTCCACCTACCAGTTGGTGGACATCCATGTGACAGAAATGGAGTCATC | 9730 |
| Kuramochi | ATGCCTCATTCCATTGGCTGGGCTCCACCTACCAGTTGGTGGACATCCATGTGACAGAAATGGAGTCATC | 9730 |
| OVCAR3 | ATGCCTCATTCCATTGGCTGGGCTCCACCTACCAGTTGGTGGACATCCATGTGACAGAAATGGAGTCATC | 9730 |
| OVCAR5 | ATGCCTCATTCCATTGGCTGGGCTCCACCTACCAGTTGGTGGACATCCATGTGACAGAAATGGAGTCATC | 9730 |
| OV1 | ATGCCTCATTCCATTGGCTGGGCTCCACCTACCAGTTGGTGGACATCCATGTGACAGAAATGGAGTCATC | 9730 |
| OV2 | ATGCCTCATTCCATTGGCTGGGCTCCACCTACCAGTTGGTGGACATCCATGTGACAGAAATGGAGTCATC | 9730 |
| OV3 | ATGCCTCATTCCATTGGCTGGGCTCCACCTACCAGTTGGTGGACATCCATGTGACAGAAATGGAGTCATC | 9730 |

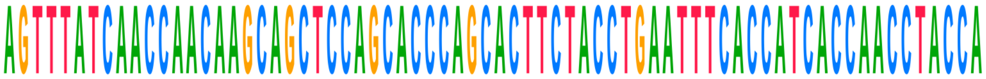

| | | |
|---|---|---|
| **Consensus** | **AGTTTATCAACCAACAAGCAGCTCCAGCACCCAGCACTTCTACCTGAATTTCACCATCACCAACCTACCA** | |
| NM_001414687.1 (35,819 .. 46,231) | AGTTTATCAACCAACAAGCAGCTCCAGCACCCAGCACTTCTACCTGAATTTCACCATCACCAACCTACCA | 9800 |
| Kuramochi | AGTTTATCAACCAACAAGCAGCTCCAGCACCCAGCACTTCTACCTGAATTTCACCATCACCAACCTACCA | 9800 |
| OVCAR3 | AGTTTATCAACCAACAAGCAGCTCCAGCACCCAGCACTTCTACCTGAATTTCACCATCACCAACCTACCA | 9800 |
| OVCAR5 | AGTTTATCAACCAACAAGCAGCTCCAGCACCCAGCACTTCTACCTGAATTTCACCATCACCAACCTACCA | 9800 |
| OV1 | AGTTTATCAACCAACAAGCAGCTCCAGCACCCAGCACTTCTACCTGAATTTCACCATCACCAACCTACCA | 9800 |
| OV2 | AGTTTATCAACCAACAAGCAGCTCCAGCACCCAGCACTTCTACCTGAATTTCACCATCACCAACCTACCA | 9800 |
| OV3 | AGTTTATCAACCAACAAGCAGCTCCAGCACCCAGCACTTCTACCTGAATTTCACCATCACCAACCTACCA | 9800 |

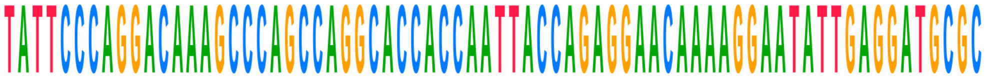

| | | |
|---|---|---|
| **Consensus** | **TATTCCCAGGACAAAGCCCAGCCAGGCACCACCAATTACCAGAGGAACAAAAGGAATATTGAGGATGCGC** | |
| NM_001414687.1 (35,819 .. 46,231) | TATTCCCAGGACAAAGCCCAGCCAGGCACCACCAATTACCAGAGGAACAAAAGGAATATTGAGGATGCGC | 9870 |
| Kuramochi | TATTCCCAGGACAAAGCCCAGCCAGGCACCACCAATTACCAGAGGAACAAAAGGAATATTGAGGATGCGC | 9870 |
| OVCAR3 | TATTCCCAGGACAAAGCCCAGCCAGGCACCACCAATTACCAGAGGAACAAAAGGAATATTGAGGATGCGC | 9870 |
| OVCAR5 | TATTCCCAGGACAAAGCCCAGCCAGGCACCACCAATTACCAGAGGAACAAAAGGAATATTGAGGATGCGC | 9870 |
| OV1 | TATTCCCAGGACAAAGCCCAGCCAGGCACCACCAATTACCAGAGGAACAAAAGGAATATTGAGGATGCGC | 9870 |
| OV2 | TATTCCCAGGACAAAGCCCAGCCAGGCACCACCAATTACCAGAGGAACAAAAGGAATATTGAGGATGCGC | 9870 |
| OV3 | TATTCCCAGGACAAAGCCCAGCCAGGCACCACCAATTACCAGAGGAACAAAAGGAATATTGAGGATGCGC | 9870 |

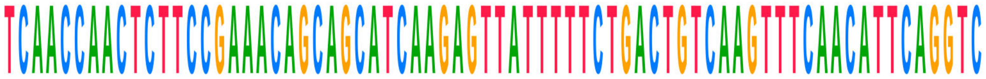

| | | |
|---|---|---|
| **Consensus** | **TCAACCAACTCTTCCGAAACAGCAGCATCAAGAGTTATTTTTCTGACTGTCAAGTTTCAACATTCAGGTC** | |
| NM_001414687.1 (35,819 .. 46,231) | TCAACCAACTCTTCCGAAACAGCAGCATCAAGAGTTATTTTTCTGACTGTCAAGTTTCAACATTCAGGTC | 9940 |
| Kuramochi | TCAACCAACTCTTCCGAAACAGCAGCATCAAGAGTTATTTTTCTGACTGTCAAGTTTCAACATTCAGGTC | 9940 |
| OVCAR3 | TCAACCAACTCTTCCGAAACAGCAGCATCAAGAGTTATTTTTCTGACTGTCAAGTTTCAACATTCAGGTC | 9940 |
| OVCAR5 | TCAACCAACTCTTCCGAAACAGCAGCATCAAGAGTTATTTTTCTGACTGTCAAGTTTCAACATTCAGGTC | 9940 |
| OV1 | TCAACCAACTCTTCCGAAACAGCAGCATCAAGAGTTATTTTTCTGACTGTCAAGTTTCAACATTCAGGTC | 9940 |
| OV2 | TCAACCAACTCTTCCGAAACAGCAGCATCAAGAGTTATTTTTCTGACTGTCAAGTTTCAACATTCAGGTC | 9940 |
| OV3 | TCAACCAACTCTTCCGAAACAGCAGCATCAAGAGTTATTTTTCTGACTGTCAAGTTTCAACATTCAGGTC | 9940 |

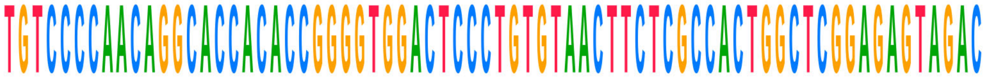

| | | |
|---|---|---|
| **Consensus** | **TGTCCCCAACAGGCACCACACCGGGGTGGACTCCCTGTGTAACTTCTCGCCACTGGCTCGGAGAGTAGAC** | |
| NM_001414687.1 (35,819 .. 46,231) | TGTCCCCAACAGGCACCACACCGGGGTGGACTCCCTGTGTAACTTCTCGCCACTGGCTCGGAGAGTAGAC | 10,010 |
| Kuramochi | TGTCCCCAACAGGCACCACACCGGGGTGGACTCCCTGTGTAACTTCTCGCCACTGGCTCGGAGAGTAGAC | 10,010 |
| OVCAR3 | TGTCCCCAACAGGCACCACACCGGGGTGGACTCCCTGTGTAACTTCTCGCCACTGGCTCGGAGAGTAGAC | 10,010 |
| OVCAR5 | TGTCCCCAACAGGCACCACACCGGGGTGGACTCCCTGTGTAACTTCTCGCCACTGGCTCGGAGAGTAGAC | 10,010 |
| OV1 | TGTCCCCAACAGGCACCACACCGGGGTGGACTCCCTGTGTAACTTCTCGCCACTGGCTCGGAGAGTAGAC | 10,010 |
| OV2 | TGTCCCCAACAGGCACCACACCGGGGTGGACTCCCTGTGTAACTTCTCGCCACTGGCTCGGAGAGTAGAC | 10,010 |
| OV3 | TGTCCCCAACAGGCACCACACCGGGGTGGACTCCCTGTGTAACTTCTCGCCACTGGCTCGGAGAGTAGAC | 10,010 |

```
Consensus                        AGAGTTGCCATCTATGAGGAATTTCTGCGGATGACCCGGAATGGTACCCAGCTGCAGAACTTCACCCTGG
NM_001414687.1 (35,819 .. 46,231) AGAGTTGCCATCTATGAGGAATTTCTGCGGATGACCCGGAATGGTACCCAGCTGCAGAACTTCACCCTGG 10,080
Kuramochi                        AGAGTTGCCATCTATGAGGAATTTCTGCGGATGACCCGGAATGGTACCCAGCTGCAGAACTTCACCCTGG 10,080
OVCAR3                           AGAGTTGCCATCTATGAGGAATTTCTGCGGATGACCCGGAATGGTACCCAGCTGCAGAACTTCACCCTGG 10,080
OVCAR5                           AGAGTTGCCATCTATGAGGAATTTCTGCGGATGACCCGGAATGGTACCCAGCTGCAGAACTTCACCCTGG 10,080
OV1                              AGAGTTGCCATCTATGAGGAATTTCTGCGGATGACCCGGAATGGTACCCAGCTGCAGAACTTCACCCTGG 10,080
OV2                              AGAGTTGCCATCTATGAGGAATTTCTGCGGATGACCCGGAATGGTACCCAGCTGCAGAACTTCACCCTGG 10,080
OV3                              AGAGTTGCCATCTATGAGGAATTTCTGCGGATGACCCGGAATGGTACCCAGCTGCAGAACTTCACCCTGG 10,080

Consensus                        ACAGGAGCAGTGTCCTTGTGGATGGGTATTCTCCCAACAGAAATGAGCCCTTAACTGGGAATTCTGACCT
NM_001414687.1 (35,819 .. 46,231) ACAGGAGCAGTGTCCTTGTGGATGGGTATTCTCCCAACAGAAATGAGCCCTTAACTGGGAATTCTGACCT 10,150
Kuramochi                        ACAGGAGCAGTGTCCTTGTGGATGGGTATTCTCCCAACAGAAATGAGCCCTTAACTGGGAATTCTGACCT 10,150
OVCAR3                           ACAGGAGCAGTGTCCTTGTGGATGGGTATTCTCCCAACAGAAATGAGCCCTTAACTGGGAATTCTGACCT 10,150
OVCAR5                           ACAGGAGCAGTGTCCTTGTGGATGGGTATTCTCCCAACAGAAATGAGCCCTTAACTGGGAATTCTGACCT 10,150
OV1                              ACAGGAGCAGTGTCCTTGTGGATGGGTATTCTCCCAACAGAAATGAGCCCTTAACTGGGAATTCTGACCT 10,150
OV2                              ACAGGAGCAGTGTCCTTGTGGATGGGTATTCTCCCAACAGAAATGAGCCCTTAACTGGGAATTCTGACCT 10,150
OV3                              ACAGGAGCAGTGTCCTTGTGGATGGGTATTCTCCCAACAGAAATGAGCCCTTAACTGGGAATTCTGACCT 10,150

Consensus                        TCCCTTCTGGGCTGTCATCCTCATCGGCTTGGCAGGACTCCTGGGACTCATCACATGCCTGATCTGCGGT
NM_001414687.1 (35,819 .. 46,231) TCCCTTCTGGGCTGTCATCCTCATCGGCTTGGCAGGACTCCTGGGAGTCATCACATGCCTGATCTGCGGT 10,220
Kuramochi                        TCCCTTCTGGGCTGTCATCCTCATCGGCTTGGCAGGACTCCTGGGACTCATCACATGCCTGATCTGCGGT 10,220
OVCAR3                           TCCCTTCTGGGCTGTCATCCTCATCGGCTTGGCAGGACTCCTGGGACTCATCACATGCCTGATCTGCGGT 10,220
OVCAR5                           TCCCTTCTGGGCTGTCATCCTCATCGGCTTGGCAGGACTCCTGGGACTCATCACATGCCTGATCTGCGGT 10,220
OV1                              TCCCTTCTGGGCTGTCATCCTCATCGGCTTGGCAGGACTCCTGGGACTCATCACATGCCTGATCTGCGGT 10,220
OV2                              TCCCTTCTGGGCTGTCATCCTCATCGGCTTGGCAGGACTCCTGGGACTCATCACATGCCTGATCTGCGGT 10,220
OV3                              TCCCTTCTGGGCTGTCATCCTCATCGGCTTGGCAGGACTCCTGGGACTCATCACATGCCTGATCTGCGGT 10,220

Consensus                        GTCCTGGTGACCACCCGCCGGCGGAAGAAGGAAGGAGAATACAACGTCCAGCAACAGTGCCCAGGCTACT
NM_001414687.1 (35,819 .. 46,231) GTCCTGGTGACCACCCGCCGGCGGAAGAAGGAAGGAGAATACAACGTCCAGCAACAGTGCCCAGGCTACT 10,290
Kuramochi                        GTCCTGGTGACCACCCGCCGGCGGAAGAAGGAAGGAGAATACAACGTCCAGCAACAGTGCCCAGGCTACT 10,290
OVCAR3                           GTCCTGGTGACCACCCGCCGGCGGAAGAAGGAAGGAGAATACAACGTCCAGCAACAGTGCCCAGGCTACT 10,290
OVCAR5                           GTCCTGGTGACCACCCGCCGGCGGAAGAAGGAAGGAGAATACAACGTCCAGCAACAGTGCCCAGGCTACT 10,290
OV1                              GTCCTGGTGACCACCCGCCGGCGGAAGAAGGAAGGAGAATACAACGTCCAGCAACAGTGCCCAGGCTACT 10,290
OV2                              GTCCTGGTGACCACCCGCCGGCGGAAGAAGGAAGGAGAATACAACGTCCAGCAACAGTGCCCAGGCTACT 10,290
OV3                              GTCCTGGTGACCACCCGCCGGCGGAAGAAGGAAGGAGAATACAACGTCCAGCAACAGTGCCCAGGCTACT 10,290

Consensus                        ACCAGTCACACCTAGACCTGGAGGATCTGCAA--------------------------------------
NM_001414687.1 (35,819 .. 46,231) ACCAGTCACACCTAGACCTGGAGGATCTGCAATGACTGGAACTTGCCGGTGCCTGGGGTGCCTTTCCCC 10,360
Kuramochi                        ACCAGTCACACCTAGACCTGGAGGATCTGCAA-------------------------------------- 10,322
OVCAR3                           ACCAGTCACACCTAGACCTGGAGGATCTGCAA-------------------------------------- 10,322
OVCAR5                           ACCAGTCACACCTAGACCTGGAGGATCTGCAA-------------------------------------- 10,322
OV1                              ACCAGTCACACCTAGACCTGGAGGATCTGCAA-------------------------------------- 10,322
OV2                              ACCAGTCACACCTAGACCTGGAGGATCTGCAA-------------------------------------- 10,322
OV3                              ACCAGTCACACCTAGACCTGGAGGATCTGCAA-------------------------------------- 10,322

Consensus                        -----------------------------------------------------
NM_001414687.1 (35,819 .. 46,231) AGCCAGGGTCCAAAGAAGCTTGGCTGGGGCAGAAATAAACCATATTGGTCGGA 10,413
Kuramochi                        ----------------------------------------------------- 10,322
OVCAR3                           ----------------------------------------------------- 10,322
OVCAR5                           ----------------------------------------------------- 10,322
OV1                              ----------------------------------------------------- 10,322
OV2                              ----------------------------------------------------- 10,322
OV3                              ----------------------------------------------------- 10,322
```
