# Supplementary material for: A Revised Molecular Model of Ovarian Cancer Biomarker CA125 (MUC16) Enabled by Long-read Sequencing
Source: Cancer Res Commun. 2024 Jan 31;4(1):253–63. doi: 10.1158/2767-9764.CRC-23-0327 (PMC10829539; doi:10.1158/2767-9764.CRC-23-0327)
Supplement: Supplementary Document 3 — Protein alignment [file crc-23-0327-s06.pdf]

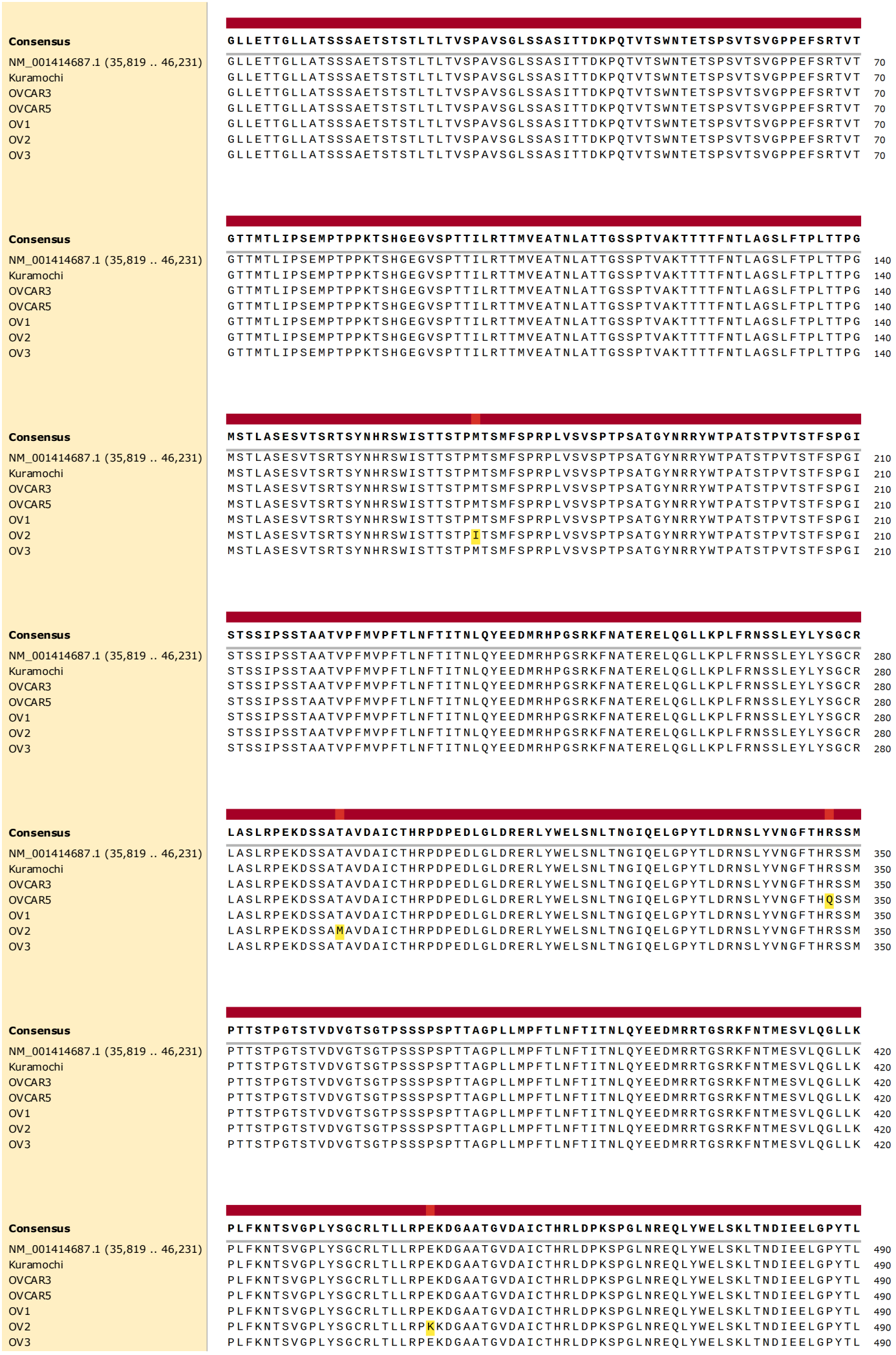
Consensus
GLLETTGLLATSSSAETSTSTLTLTVSPAVSGLSSASITTDKPQTVTSWNTETSPSVTSVGPPEFSRTVT
NM_001414687.1 (35,819 .. 46,231) GLLETTGLLATSSSAETSTSTLTLTVSPAVSGLSSASITTDKPQTVTSWNTETSPSVTSVGPPEFSRTVT 70
Kuramochi GLLETTGLLATSSSAETSTSTLTLTVSPAVSGLSSASITTDKPQTVTSWNTETSPSVTSVGPPEFSRTVT 70
OVCAR3 GLLETTGLLATSSSAETSTSTLTLTVSPAVSGLSSASITTDKPQTVTSWNTETSPSVTSVGPPEFSRTVT 70
OVCAR5 GLLETTGLLATSSSAETSTSTLTLTVSPAVSGLSSASITTDKPQTVTSWNTETSPSVTSVGPPEFSRTVT 70
OV1 GLLETTGLLATSSSAETSTSTLTLTVSPAVSGLSSASITTDKPQTVTSWNTETSPSVTSVGPPEFSRTVT 70
OV2 GLLETTGLLATSSSAETSTSTLTLTVSPAVSGLSSASITTDKPQTVTSWNTETSPSVTSVGPPEFSRTVT 70
OV3 GLLETTGLLATSSSAETSTSTLTLTVSPAVSGLSSASITTDKPQTVTSWNTETSPSVTSVGPPEFSRTVT 70
Consensus
GTTMTLIPSEMPTPPKTSHGEGVSPTTILRTTMVEATNLATTGSSPTVAKTTTTFNTLAGSLFTPLTTPG
NM_001414687.1 (35,819 .. 46,231) GTTMTLIPSEMPTPPKTSHGEGVSPTTILRTTMVEATNLATTGSSPTVAKTTTTFNTLAGSLFTPLTTPG 140
Kuramochi GTTMTLIPSEMPTPPKTSHGEGVSPTTILRTTMVEATNLATTGSSPTVAKTTTTFNTLAGSLFTPLTTPG 140
OVCAR3 GTTMTLIPSEMPTPPKTSHGEGVSPTTILRTTMVEATNLATTGSSPTVAKTTTTFNTLAGSLFTPLTTPG 140
OVCAR5 GTTMTLIPSEMPTPPKTSHGEGVSPTTILRTTMVEATNLATTGSSPTVAKTTTTFNTLAGSLFTPLTTPG 140
OV1 GTTMTLIPSEMPTPPKTSHGEGVSPTTILRTTMVEATNLATTGSSPTVAKTTTTFNTLAGSLFTPLTTPG 140
OV2 GTTMTLIPSEMPTPPKTSHGEGVSPTTILRTTMVEATNLATTGSSPTVAKTTTTFNTLAGSLFTPLTTPG 140
OV3 GTTMTLIPSEMPTPPKTSHGEGVSPTTILRTTMVEATNLATTGSSPTVAKTTTTFNTLAGSLFTPLTTPG 140
Consensus
MSTLASESVTSRTSYNHRSWISTTSTPMTSMFSPRPLVSVSPTPSATGYNRRYWTPATSTPVTSTFSPGI
NM_001414687.1 (35,819 .. 46,231) MSTLASESVTSRTSYNHRSWISTTSTPMTSMFSPRPLVSVSPTPSATGYNRRYWTPATSTPVTSTFSPGI 210
Kuramochi MSTLASESVTSRTSYNHRSWISTTSTPMTSMFSPRPLVSVSPTPSATGYNRRYWTPATSTPVTSTFSPGI 210
OVCAR3 MSTLASESVTSRTSYNHRSWISTTSTPMTSMFSPRPLVSVSPTPSATGYNRRYWTPATSTPVTSTFSPGI 210
OVCAR5 MSTLASESVTSRTSYNHRSWISTTSTPMTSMFSPRPLVSVSPTPSATGYNRRYWTPATSTPVTSTFSPGI 210
OV1 MSTLASESVTSRTSYNHRSWISTTSTPMTSMFSPRPLVSVSPTPSATGYNRRYWTPATSTPVTSTFSPGI 210
OV2 MSTLASESVTSRTSYNHRSWISTTSTPITSMFSPRPLVSVSPTPSATGYNRRYWTPATSTPVTSTFSPGI 210
OV3 MSTLASESVTSRTSYNHRSWISTTSTPMTSMFSPRPLVSVSPTPSATGYNRRYWTPATSTPVTSTFSPGI 210
Consensus
STSSIPSSTAATVPFMVPFTLNFTITNLQYEEDMRHPGSRKFNATERELQGLLKPLFRNSSLEYLYSGCR
NM_001414687.1 (35,819 .. 46,231) STSSIPSSTAATVPFMVPFTLNFTITNLQYEEDMRHPGSRKFNATERELQGLLKPLFRNSSLEYLYSGCR 280
Kuramochi STSSIPSSTAATVPFMVPFTLNFTITNLQYEEDMRHPGSRKFNATERELQGLLKPLFRNSSLEYLYSGCR 280
OVCAR3 STSSIPSSTAATVPFMVPFTLNFTITNLQYEEDMRHPGSRKFNATERELQGLLKPLFRNSSLEYLYSGCR 280
OVCAR5 STSSIPSSTAATVPFMVPFTLNFTITNLQYEEDMRHPGSRKFNATERELQGLLKPLFRNSSLEYLYSGCR 280
OV1 STSSIPSSTAATVPFMVPFTLNFTITNLQYEEDMRHPGSRKFNATERELQGLLKPLFRNSSLEYLYSGCR 280
OV2 STSSIPSSTAATVPFMVPFTLNFTITNLQYEEDMRHPGSRKFNATERELQGLLKPLFRNSSLEYLYSGCR 280
OV3 STSSIPSSTAATVPFMVPFTLNFTITNLQYEEDMRHPGSRKFNATERELQGLLKPLFRNSSLEYLYSGCR 280
Consensus
LASLRPEKDSSATAVDAICTHRPDPEDLGLDRERLYWELSNLTNGIQELGPYTLDRNSLYVNGFTHRSSM
NM_001414687.1 (35,819 .. 46,231) LASLRPEKDSSATAVDAICTHRPDPEDLGLDRERLYWELSNLTNGIQELGPYTLDRNSLYVNGFTHRSSM 350
Kuramochi LASLRPEKDSSATAVDAICTHRPDPEDLGLDRERLYWELSNLTNGIQELGPYTLDRNSLYVNGFTHRSSM 350
OVCAR3 LASLRPEKDSSATAVDAICTHRPDPEDLGLDRERLYWELSNLTNGIQELGPYTLDRNSLYVNGFTHRSSM 350
OVCAR5 LASLRPEKDSSATAVDAICTHRPDPEDLGLDRERLYWELSNLTNGIQELGPYTLDRNSLYVNGFTHQSSM 350
OV1 LASLRPEKDSSATAVDAICTHRPDPEDLGLDRERLYWELSNLTNGIQELGPYTLDRNSLYVNGFTHRSSM 350
OV2 LASLRPEKDSSAMAVDAICTHRPDPEDLGLDRERLYWELSNLTNGIQELGPYTLDRNSLYVNGFTHRSSM 350
OV3 LASLRPEKDSSATAVDAICTHRPDPEDLGLDRERLYWELSNLTNGIQELGPYTLDRNSLYVNGFTHRSSM 350
Consensus
PTTSTPGTSTVDVGTSGTPSSSPSPTTAGPLLMPFTLNFTITNLQYEEDMRRTGSRKFNTMESVLQGLLK
NM_001414687.1 (35,819 .. 46,231) PTTSTPGTSTVDVGTSGTPSSSPSPTTAGPLLMPFTLNFTITNLQYEEDMRRTGSRKFNTMESVLQGLLK 420
Kuramochi PTTSTPGTSTVDVGTSGTPSSSPSPTTAGPLLMPFTLNFTITNLQYEEDMRRTGSRKFNTMESVLQGLLK 420
OVCAR3 PTTSTPGTSTVDVGTSGTPSSSPSPTTAGPLLMPFTLNFTITNLQYEEDMRRTGSRKFNTMESVLQGLLK 420
OVCAR5 PTTSTPGTSTVDVGTSGTPSSSPSPTTAGPLLMPFTLNFTITNLQYEEDMRRTGSRKFNTMESVLQGLLK 420
OV1 PTTSTPGTSTVDVGTSGTPSSSPSPTTAGPLLMPFTLNFTITNLQYEEDMRRTGSRKFNTMESVLQGLLK 420
OV2 PTTSTPGTSTVDVGTSGTPSSSPSPTTAGPLLMPFTLNFTITNLQYEEDMRRTGSRKFNTMESVLQGLLK 420
OV3 PTTSTPGTSTVDVGTSGTPSSSPSPTTAGPLLMPFTLNFTITNLQYEEDMRRTGSRKFNTMESVLQGLLK 420
Consensus
PLFKNTSVGPLYSGCRLTLLRPEKDGAATGVDAICTHRLDPKSPGLNREQLYWELSKLTNDIEELGPYTL
NM_001414687.1 (35,819 .. 46,231) PLFKNTSVGPLYSGCRLTLLRPEKDGAATGVDAICTHRLDPKSPGLNREQLYWELSKLTNDIEELGPYTL 490
Kuramochi PLFKNTSVGPLYSGCRLTLLRPEKDGAATGVDAICTHRLDPKSPGLNREQLYWELSKLTNDIEELGPYTL 490
OVCAR3 PLFKNTSVGPLYSGCRLTLLRPEKDGAATGVDAICTHRLDPKSPGLNREQLYWELSKLTNDIEELGPYTL 490
OVCAR5 PLFKNTSVGPLYSGCRLTLLRPEKDGAATGVDAICTHRLDPKSPGLNREQLYWELSKLTNDIEELGPYTL 490
OV1 PLFKNTSVGPLYSGCRLTLLRPEKDGAATGVDAICTHRLDPKSPGLNREQLYWELSKLTNDIEELGPYTL 490
OV2 PLFKNTSVGPLYSGCRLTLLRPKKDGAATGVDAICTHRLDPKSPGLNREQLYWELSKLTNDIEELGPYTL 490
OV3 PLFKNTSVGPLYSGCRLTLLRPEKDGAATGVDAICTHRLDPKSPGLNREQLYWELSKLTNDIEELGPYTL 490

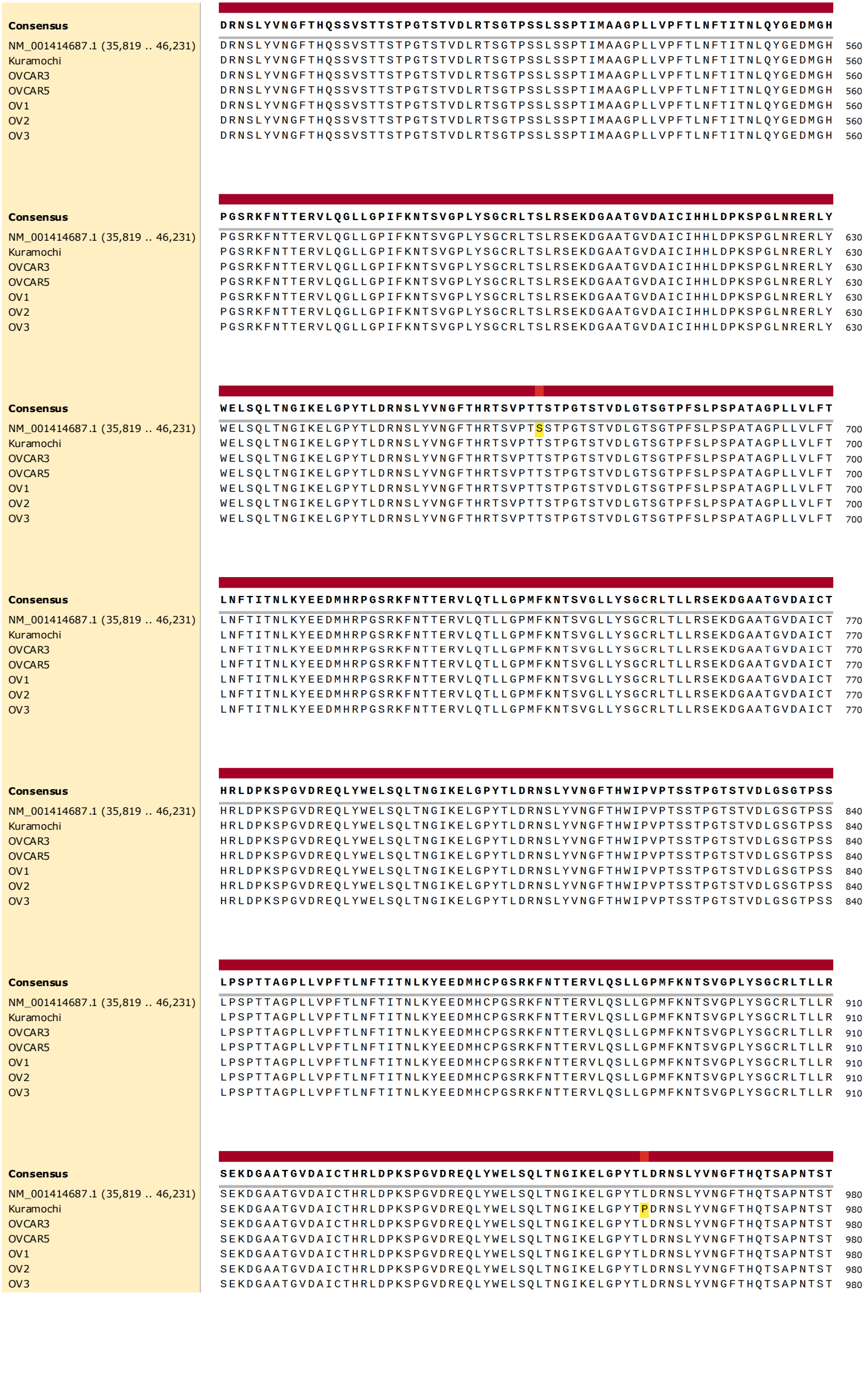
Consensus
DRNSLYVNGFTHQSSVSTTSTPGTSTVDLRTSGTPSSLSSPTIMAAGPLLVPFTLNFTITNLQYGEDMGH
NM_001414687.1 (35,819 .. 46,231) DRNSLYVNGFTHQSSVSTTSTPGTSTVDLRTSGTPSSLSSPTIMAAGPLLVPFTLNFTITNLQYGEDMGH 560
Kuramochi DRNSLYVNGFTHQSSVSTTSTPGTSTVDLRTSGTPSSLSSPTIMAAGPLLVPFTLNFTITNLQYGEDMGH 560
OVCAR3 DRNSLYVNGFTHQSSVSTTSTPGTSTVDLRTSGTPSSLSSPTIMAAGPLLVPFTLNFTITNLQYGEDMGH 560
OVCAR5 DRNSLYVNGFTHQSSVSTTSTPGTSTVDLRTSGTPSSLSSPTIMAAGPLLVPFTLNFTITNLQYGEDMGH 560
OV1 DRNSLYVNGFTHQSSVSTTSTPGTSTVDLRTSGTPSSLSSPTIMAAGPLLVPFTLNFTITNLQYGEDMGH 560
OV2 DRNSLYVNGFTHQSSVSTTSTPGTSTVDLRTSGTPSSLSSPTIMAAGPLLVPFTLNFTITNLQYGEDMGH 560
OV3 DRNSLYVNGFTHQSSVSTTSTPGTSTVDLRTSGTPSSLSSPTIMAAGPLLVPFTLNFTITNLQYGEDMGH 560
Consensus
PGSRKFNTTERVLQGLLGPIFKNTSVGPLYSGCRLTSLRSEKDGAATGVDAICIHHLDPKSPGLNRERLY
NM_001414687.1 (35,819 .. 46,231) PGSRKFNTTERVLQGLLGPIFKNTSVGPLYSGCRLTSLRSEKDGAATGVDAICIHHLDPKSPGLNRERLY 630
Kuramochi PGSRKFNTTERVLQGLLGPIFKNTSVGPLYSGCRLTSLRSEKDGAATGVDAICIHHLDPKSPGLNRERLY 630
OVCAR3 PGSRKFNTTERVLQGLLGPIFKNTSVGPLYSGCRLTSLRSEKDGAATGVDAICIHHLDPKSPGLNRERLY 630
OVCAR5 PGSRKFNTTERVLQGLLGPIFKNTSVGPLYSGCRLTSLRSEKDGAATGVDAICIHHLDPKSPGLNRERLY 630
OV1 PGSRKFNTTERVLQGLLGPIFKNTSVGPLYSGCRLTSLRSEKDGAATGVDAICIHHLDPKSPGLNRERLY 630
OV2 PGSRKFNTTERVLQGLLGPIFKNTSVGPLYSGCRLTSLRSEKDGAATGVDAICIHHLDPKSPGLNRERLY 630
OV3 PGSRKFNTTERVLQGLLGPIFKNTSVGPLYSGCRLTSLRSEKDGAATGVDAICIHHLDPKSPGLNRERLY 630
Consensus
WELSQLTNGIKELGPYTLDRNSLYVNGFTHRTSVPTTSTPGTSTVDLGTSGTPFSLPSPATAGPLLVLFT
NM_001414687.1 (35,819 .. 46,231) WELSQLTNGIKELGPYTLDRNSLYVNGFTHRTSVPTSSTPGTSTVDLGTSGTPFSLPSPATAGPLLVLFT 700
Kuramochi WELSQLTNGIKELGPYTLDRNSLYVNGFTHRTSVPTTSTPGTSTVDLGTSGTPFSLPSPATAGPLLVLFT 700
OVCAR3 WELSQLTNGIKELGPYTLDRNSLYVNGFTHRTSVPTTSTPGTSTVDLGTSGTPFSLPSPATAGPLLVLFT 700
OVCAR5 WELSQLTNGIKELGPYTLDRNSLYVNGFTHRTSVPTTSTPGTSTVDLGTSGTPFSLPSPATAGPLLVLFT 700
OV1 WELSQLTNGIKELGPYTLDRNSLYVNGFTHRTSVPTTSTPGTSTVDLGTSGTPFSLPSPATAGPLLVLFT 700
OV2 WELSQLTNGIKELGPYTLDRNSLYVNGFTHRTSVPTTSTPGTSTVDLGTSGTPFSLPSPATAGPLLVLFT 700
OV3 WELSQLTNGIKELGPYTLDRNSLYVNGFTHRTSVPTTSTPGTSTVDLGTSGTPFSLPSPATAGPLLVLFT 700
Consensus
LNFTITNLKYEEDMHRPGSRKFNTTERVLQTLLGPMFKNTSVGLLYSGCRLTLLRSEKDGAATGVDAICT
NM_001414687.1 (35,819 .. 46,231) LNFTITNLKYEEDMHRPGSRKFNTTERVLQTLLGPMFKNTSVGLLYSGCRLTLLRSEKDGAATGVDAICT 770
Kuramochi LNFTITNLKYEEDMHRPGSRKFNTTERVLQTLLGPMFKNTSVGLLYSGCRLTLLRSEKDGAATGVDAICT 770
OVCAR3 LNFTITNLKYEEDMHRPGSRKFNTTERVLQTLLGPMFKNTSVGLLYSGCRLTLLRSEKDGAATGVDAICT 770
OVCAR5 LNFTITNLKYEEDMHRPGSRKFNTTERVLQTLLGPMFKNTSVGLLYSGCRLTLLRSEKDGAATGVDAICT 770
OV1 LNFTITNLKYEEDMHRPGSRKFNTTERVLQTLLGPMFKNTSVGLLYSGCRLTLLRSEKDGAATGVDAICT 770
OV2 LNFTITNLKYEEDMHRPGSRKFNTTERVLQTLLGPMFKNTSVGLLYSGCRLTLLRSEKDGAATGVDAICT 770
OV3 LNFTITNLKYEEDMHRPGSRKFNTTERVLQTLLGPMFKNTSVGLLYSGCRLTLLRSEKDGAATGVDAICT 770
Consensus
HRLDPKSPGVDREQLYWELSQLTNGIKELGPYTLDRNSLYVNGFTHWIPVPTSSTPGTSTVDLGSGTPSS
NM_001414687.1 (35,819 .. 46,231) HRLDPKSPGVDREQLYWELSQLTNGIKELGPYTLDRNSLYVNGFTHWIPVPTSSTPGTSTVDLGSGTPSS 840
Kuramochi HRLDPKSPGVDREQLYWELSQLTNGIKELGPYTLDRNSLYVNGFTHWIPVPTSSTPGTSTVDLGSGTPSS 840
OVCAR3 HRLDPKSPGVDREQLYWELSQLTNGIKELGPYTLDRNSLYVNGFTHWIPVPTSSTPGTSTVDLGSGTPSS 840
OVCAR5 HRLDPKSPGVDREQLYWELSQLTNGIKELGPYTLDRNSLYVNGFTHWIPVPTSSTPGTSTVDLGSGTPSS 840
OV1 HRLDPKSPGVDREQLYWELSQLTNGIKELGPYTLDRNSLYVNGFTHWIPVPTSSTPGTSTVDLGSGTPSS 840
OV2 HRLDPKSPGVDREQLYWELSQLTNGIKELGPYTLDRNSLYVNGFTHWIPVPTSSTPGTSTVDLGSGTPSS 840
OV3 HRLDPKSPGVDREQLYWELSQLTNGIKELGPYTLDRNSLYVNGFTHWIPVPTSSTPGTSTVDLGSGTPSS 840
Consensus
LPSPTTAGPLLVPFTLNFTITNLKYEEDMHCPGSRKFNTTERVLQSLLGPMFKNTSVGPLYSGCRLTLLR
NM_001414687.1 (35,819 .. 46,231) LPSPTTAGPLLVPFTLNFTITNLKYEEDMHCPGSRKFNTTERVLQSLLGPMFKNTSVGPLYSGCRLTLLR 910
Kuramochi LPSPTTAGPLLVPFTLNFTITNLKYEEDMHCPGSRKFNTTERVLQSLLGPMFKNTSVGPLYSGCRLTLLR 910
OVCAR3 LPSPTTAGPLLVPFTLNFTITNLKYEEDMHCPGSRKFNTTERVLQSLLGPMFKNTSVGPLYSGCRLTLLR 910
OVCAR5 LPSPTTAGPLLVPFTLNFTITNLKYEEDMHCPGSRKFNTTERVLQSLLGPMFKNTSVGPLYSGCRLTLLR 910
OV1 LPSPTTAGPLLVPFTLNFTITNLKYEEDMHCPGSRKFNTTERVLQSLLGPMFKNTSVGPLYSGCRLTLLR 910
OV2 LPSPTTAGPLLVPFTLNFTITNLKYEEDMHCPGSRKFNTTERVLQSLLGPMFKNTSVGPLYSGCRLTLLR 910
OV3 LPSPTTAGPLLVPFTLNFTITNLKYEEDMHCPGSRKFNTTERVLQSLLGPMFKNTSVGPLYSGCRLTLLR 910
Consensus
SEKDGAATGVDAICTHRLDPKSPGVDREQLYWELSQLTNGIKELGPYTLDRNSLYVNGFTHQTSAPNTST
NM_001414687.1 (35,819 .. 46,231) SEKDGAATGVDAICTHRLDPKSPGVDREQLYWELSQLTNGIKELGPYTLDRNSLYVNGFTHQTSAPNTST 980
Kuramochi SEKDGAATGVDAICTHRLDPKSPGVDREQLYWELSQLTNGIKELGPYTPDRNSLYVNGFTHQTSAPNTST 980
OVCAR3 SEKDGAATGVDAICTHRLDPKSPGVDREQLYWELSQLTNGIKELGPYTLDRNSLYVNGFTHQTSAPNTST 980
OVCAR5 SEKDGAATGVDAICTHRLDPKSPGVDREQLYWELSQLTNGIKELGPYTLDRNSLYVNGFTHQTSAPNTST 980
OV1 SEKDGAATGVDAICTHRLDPKSPGVDREQLYWELSQLTNGIKELGPYTLDRNSLYVNGFTHQTSAPNTST 980
OV2 SEKDGAATGVDAICTHRLDPKSPGVDREQLYWELSQLTNGIKELGPYTLDRNSLYVNGFTHQTSAPNTST 980
OV3 SEKDGAATGVDAICTHRLDPKSPGVDREQLYWELSQLTNGIKELGPYTLDRNSLYVNGFTHQTSAPNTST 980

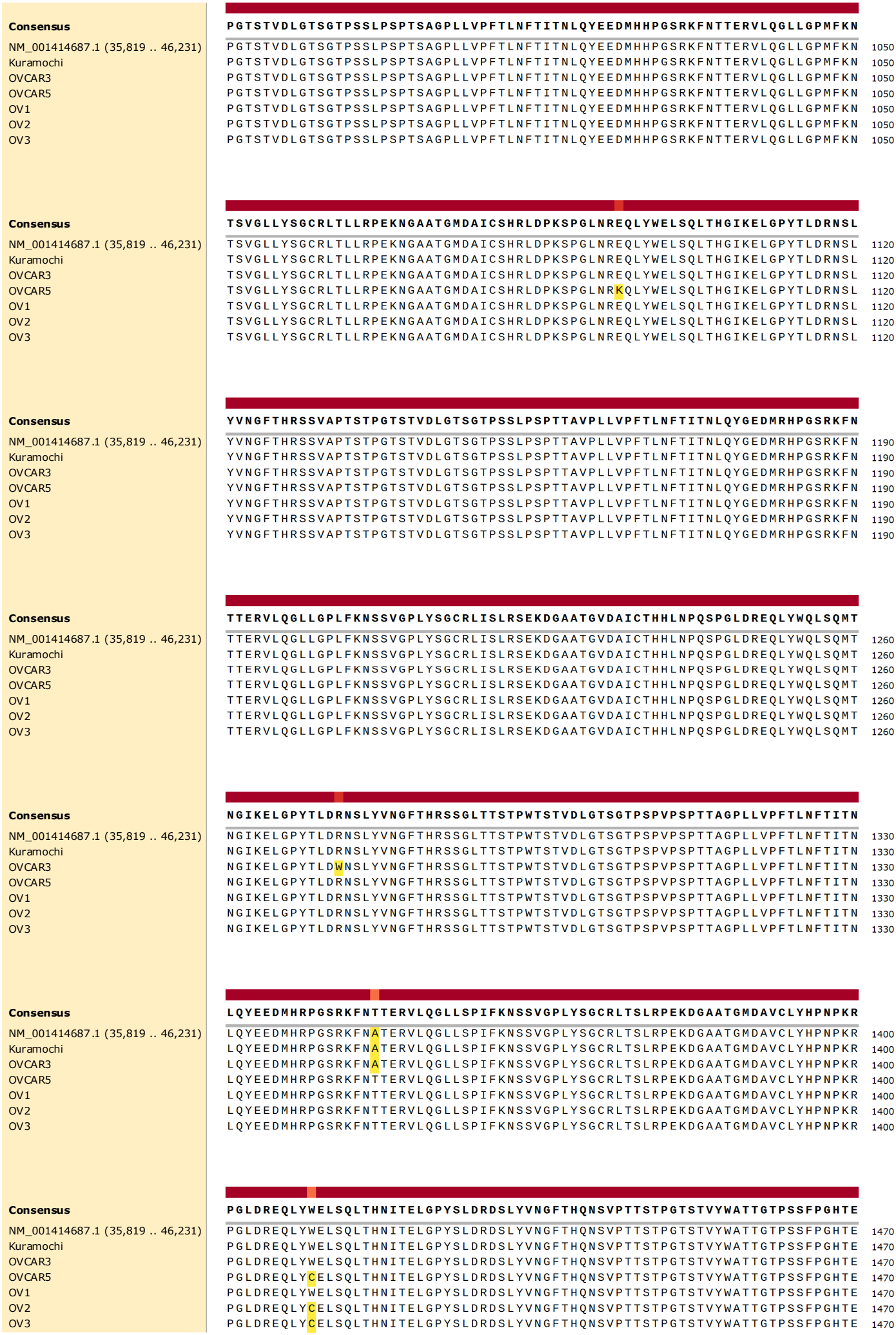
Consensus
PGTSTVDLGTSGTPSSLPSPTSAGPLLVPFTLNFTITNLQYEEDMHHPGSRKFNTTERVLQGLLGPMFKN
NM_001414687.1 (35,819 .. 46,231) PGTSTVDLGTSGTPSSLPSPTSAGPLLVPFTLNFTITNLQYEEDMHHPGSRKFNTTERVLQGLLGPMFKN 1050
Kuramochi PGTSTVDLGTSGTPSSLPSPTSAGPLLVPFTLNFTITNLQYEEDMHHPGSRKFNTTERVLQGLLGPMFKN 1050
OVCAR3 PGTSTVDLGTSGTPSSLPSPTSAGPLLVPFTLNFTITNLQYEEDMHHPGSRKFNTTERVLQGLLGPMFKN 1050
OVCAR5 PGTSTVDLGTSGTPSSLPSPTSAGPLLVPFTLNFTITNLQYEEDMHHPGSRKFNTTERVLQGLLGPMFKN 1050
OV1 PGTSTVDLGTSGTPSSLPSPTSAGPLLVPFTLNFTITNLQYEEDMHHPGSRKFNTTERVLQGLLGPMFKN 1050
OV2 PGTSTVDLGTSGTPSSLPSPTSAGPLLVPFTLNFTITNLQYEEDMHHPGSRKFNTTERVLQGLLGPMFKN 1050
OV3 PGTSTVDLGTSGTPSSLPSPTSAGPLLVPFTLNFTITNLQYEEDMHHPGSRKFNTTERVLQGLLGPMFKN 1050
Consensus
TSVGLLYSGCRLTLLRPEKNGAATGMDAICSHRLDPKSPGLNREQLYWELSQLTHGIKELGPYTLDRNSL
NM_001414687.1 (35,819 .. 46,231) TSVGLLYSGCRLTLLRPEKNGAATGMDAICSHRLDPKSPGLNREQLYWELSQLTHGIKELGPYTLDRNSL 1120
Kuramochi TSVGLLYSGCRLTLLRPEKNGAATGMDAICSHRLDPKSPGLNREQLYWELSQLTHGIKELGPYTLDRNSL 1120
OVCAR3 TSVGLLYSGCRLTLLRPEKNGAATGMDAICSHRLDPKSPGLNREQLYWELSQLTHGIKELGPYTLDRNSL 1120
OVCAR5 TSVGLLYSGCRLTLLRPEKNGAATGMDAICSHRLDPKSPGLNRKQLYWELSQLTHGIKELGPYTLDRNSL 1120
OV1 TSVGLLYSGCRLTLLRPEKNGAATGMDAICSHRLDPKSPGLNREQLYWELSQLTHGIKELGPYTLDRNSL 1120
OV2 TSVGLLYSGCRLTLLRPEKNGAATGMDAICSHRLDPKSPGLNREQLYWELSQLTHGIKELGPYTLDRNSL 1120
OV3 TSVGLLYSGCRLTLLRPEKNGAATGMDAICSHRLDPKSPGLNREQLYWELSQLTHGIKELGPYTLDRNSL 1120
Consensus
YVNGFTHRSSVAPTSTPGTSTVDLGTSGTPSSLPSPTTAVPLLVPFTLNFTITNLQYGEDMRHPGSRKFN
NM_001414687.1 (35,819 .. 46,231) YVNGFTHRSSVAPTSTPGTSTVDLGTSGTPSSLPSPTTAVPLLVPFTLNFTITNLQYGEDMRHPGSRKFN 1190
Kuramochi YVNGFTHRSSVAPTSTPGTSTVDLGTSGTPSSLPSPTTAVPLLVPFTLNFTITNLQYGEDMRHPGSRKFN 1190
OVCAR3 YVNGFTHRSSVAPTSTPGTSTVDLGTSGTPSSLPSPTTAVPLLVPFTLNFTITNLQYGEDMRHPGSRKFN 1190
OVCAR5 YVNGFTHRSSVAPTSTPGTSTVDLGTSGTPSSLPSPTTAVPLLVPFTLNFTITNLQYGEDMRHPGSRKFN 1190
OV1 YVNGFTHRSSVAPTSTPGTSTVDLGTSGTPSSLPSPTTAVPLLVPFTLNFTITNLQYGEDMRHPGSRKFN 1190
OV2 YVNGFTHRSSVAPTSTPGTSTVDLGTSGTPSSLPSPTTAVPLLVPFTLNFTITNLQYGEDMRHPGSRKFN 1190
OV3 YVNGFTHRSSVAPTSTPGTSTVDLGTSGTPSSLPSPTTAVPLLVPFTLNFTITNLQYGEDMRHPGSRKFN 1190
Consensus
TTERVLQGLLGPLFKNSSVGPLYSGCRLISLRSEKDGAATGVDAICTHHLNPQSPGLDREQLYWQLSQMT
NM_001414687.1 (35,819 .. 46,231) TTERVLQGLLGPLFKNSSVGPLYSGCRLISLRSEKDGAATGVDAICTHHLNPQSPGLDREQLYWQLSQMT 1260
Kuramochi TTERVLQGLLGPLFKNSSVGPLYSGCRLISLRSEKDGAATGVDAICTHHLNPQSPGLDREQLYWQLSQMT 1260
OVCAR3 TTERVLQGLLGPLFKNSSVGPLYSGCRLISLRSEKDGAATGVDAICTHHLNPQSPGLDREQLYWQLSQMT 1260
OVCAR5 TTERVLQGLLGPLFKNSSVGPLYSGCRLISLRSEKDGAATGVDAICTHHLNPQSPGLDREQLYWQLSQMT 1260
OV1 TTERVLQGLLGPLFKNSSVGPLYSGCRLISLRSEKDGAATGVDAICTHHLNPQSPGLDREQLYWQLSQMT 1260
OV2 TTERVLQGLLGPLFKNSSVGPLYSGCRLISLRSEKDGAATGVDAICTHHLNPQSPGLDREQLYWQLSQMT 1260
OV3 TTERVLQGLLGPLFKNSSVGPLYSGCRLISLRSEKDGAATGVDAICTHHLNPQSPGLDREQLYWQLSQMT 1260
Consensus
NGIKELGPYTLDRNSLYVNGFTHRSSGLTTSTPWTSTVDLGTSGTPSPVPSPTTAGPLLVPFTLNFTITN
NM_001414687.1 (35,819 .. 46,231) NGIKELGPYTLDRNSLYVNGFTHRSSGLTTSTPWTSTVDLGTSGTPSPVPSPTTAGPLLVPFTLNFTITN 1330
Kuramochi NGIKELGPYTLDRNSLYVNGFTHRSSGLTTSTPWTSTVDLGTSGTPSPVPSPTTAGPLLVPFTLNFTITN 1330
OVCAR3 NGIKELGPYTLDWNSLYVNGFTHRSSGLTTSTPWTSTVDLGTSGTPSPVPSPTTAGPLLVPFTLNFTITN 1330
OVCAR5 NGIKELGPYTLDRNSLYVNGFTHRSSGLTTSTPWTSTVDLGTSGTPSPVPSPTTAGPLLVPFTLNFTITN 1330
OV1 NGIKELGPYTLDRNSLYVNGFTHRSSGLTTSTPWTSTVDLGTSGTPSPVPSPTTAGPLLVPFTLNFTITN 1330
OV2 NGIKELGPYTLDRNSLYVNGFTHRSSGLTTSTPWTSTVDLGTSGTPSPVPSPTTAGPLLVPFTLNFTITN 1330
OV3 NGIKELGPYTLDRNSLYVNGFTHRSSGLTTSTPWTSTVDLGTSGTPSPVPSPTTAGPLLVPFTLNFTITN 1330
Consensus
LQYEEDMHRPGSRKFNTTERVLQGLLSPIFKNSSVGPLYSGCRLTSLRPEKDGAATGMDAVCLYHPNPKR
NM_001414687.1 (35,819 .. 46,231) LQYEEDMHRPGSRKFNATERVLQGLLSPIFKNSSVGPLYSGCRLTSLRPEKDGAATGMDAVCLYHPNPKR 1400
Kuramochi LQYEEDMHRPGSRKFNATERVLQGLLSPIFKNSSVGPLYSGCRLTSLRPEKDGAATGMDAVCLYHPNPKR 1400
OVCAR3 LQYEEDMHRPGSRKFNATERVLQGLLSPIFKNSSVGPLYSGCRLTSLRPEKDGAATGMDAVCLYHPNPKR 1400
OVCAR5 LQYEEDMHRPGSRKFNTTERVLQGLLSPIFKNSSVGPLYSGCRLTSLRPEKDGAATGMDAVCLYHPNPKR 1400
OV1 LQYEEDMHRPGSRKFNTTERVLQGLLSPIFKNSSVGPLYSGCRLTSLRPEKDGAATGMDAVCLYHPNPKR 1400
OV2 LQYEEDMHRPGSRKFNTTERVLQGLLSPIFKNSSVGPLYSGCRLTSLRPEKDGAATGMDAVCLYHPNPKR 1400
OV3 LQYEEDMHRPGSRKFNTTERVLQGLLSPIFKNSSVGPLYSGCRLTSLRPEKDGAATGMDAVCLYHPNPKR 1400
Consensus
PGLDREQLYWELSQLTHNITELGPYSLDRDSLYVNGFTHQNSVPTTSTPGTSTVYWATTGTPSSFPGHTE
NM_001414687.1 (35,819 .. 46,231) PGLDREQLYWELSQLTHNITELGPYSLDRDSLYVNGFTHQNSVPTTSTPGTSTVYWATTGTPSSFPGHTE 1470
Kuramochi PGLDREQLYWELSQLTHNITELGPYSLDRDSLYVNGFTHQNSVPTTSTPGTSTVYWATTGTPSSFPGHTE 1470
OVCAR3 PGLDREQLYWELSQLTHNITELGPYSLDRDSLYVNGFTHQNSVPTTSTPGTSTVYWATTGTPSSFPGHTE 1470
OVCAR5 PGLDREQLYCELSQLTHNITELGPYSLDRDSLYVNGFTHQNSVPTTSTPGTSTVYWATTGTPSSFPGHTE 1470
OV1 PGLDREQLYWELSQLTHNITELGPYSLDRDSLYVNGFTHQNSVPTTSTPGTSTVYWATTGTPSSFPGHTE 1470
OV2 PGLDREQLYCELSQLTHNITELGPYSLDRDSLYVNGFTHQNSVPTTSTPGTSTVYWATTGTPSSFPGHTE 1470
OV3 PGLDREQLYCELSQLTHNITELGPYSLDRDSLYVNGFTHQNSVPTTSTPGTSTVYWATTGTPSSFPGHTE 1470

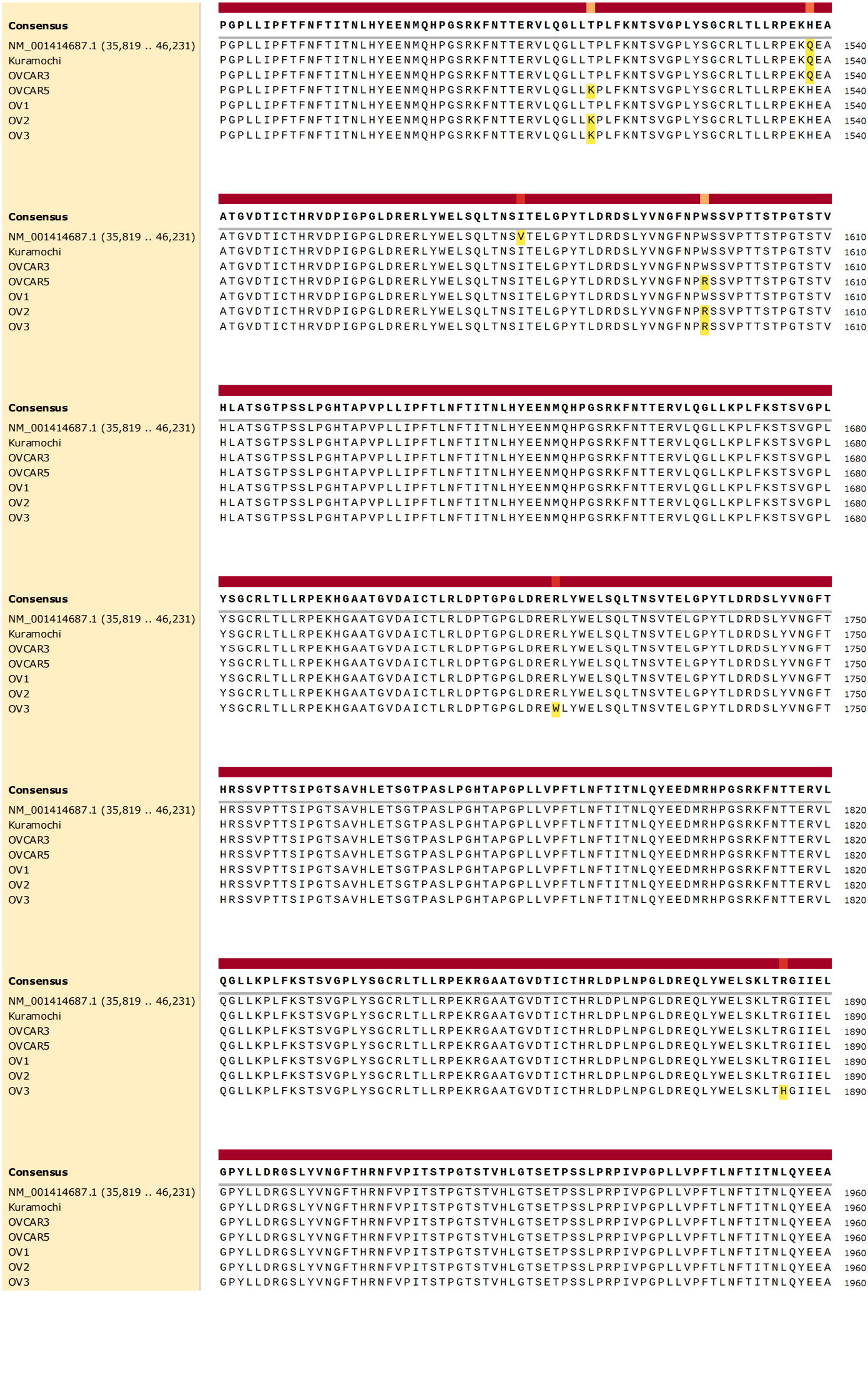
Consensus
PGPLLIPFTFNFTITNLHYEENMQHPGSRKFNTTERVLQGLLTPLFKNTSVGPLYSGCRLTLLRPEKHEA
NM_001414687.1 (35,819 .. 46,231) PGPLLIPFTFNFTITNLHYEENMQHPGSRKFNTTERVLQGLLTPLFKNTSVGPLYSGCRLTLLRPEKQEA 1540
Kuramochi PGPLLIPFTFNFTITNLHYEENMQHPGSRKFNTTERVLQGLLTPLFKNTSVGPLYSGCRLTLLRPEKQEA 1540
OVCAR3 PGPLLIPFTFNFTITNLHYEENMQHPGSRKFNTTERVLQGLLTPLFKNTSVGPLYSGCRLTLLRPEKQEA 1540
OVCAR5 PGPLLIPFTFNFTITNLHYEENMQHPGSRKFNTTERVLQGLLKPLFKNTSVGPLYSGCRLTLLRPEKHEA 1540
OV1 PGPLLIPFTFNFTITNLHYEENMQHPGSRKFNTTERVLQGLLTPLFKNTSVGPLYSGCRLTLLRPEKHEA 1540
OV2 PGPLLIPFTFNFTITNLHYEENMQHPGSRKFNTTERVLQGLLKPLFKNTSVGPLYSGCRLTLLRPEKHEA 1540
OV3 PGPLLIPFTFNFTITNLHYEENMQHPGSRKFNTTERVLQGLLKPLFKNTSVGPLYSGCRLTLLRPEKHEA 1540
Consensus
ATGVDTICTHRVDPIGPGLDRERLYWELSQLTNSITELGPYTLDRDSLYVNGFNPWSSVPTTSTPGTSTV
NM_001414687.1 (35,819 .. 46,231) ATGVDTICTHRVDPIGPGLDRERLYWELSQLTNSVTELGPYTLDRDSLYVNGFNPWSSVPTTSTPGTSTV 1610
Kuramochi ATGVDTICTHRVDPIGPGLDRERLYWELSQLTNSITELGPYTLDRDSLYVNGFNPWSSVPTTSTPGTSTV 1610
OVCAR3 ATGVDTICTHRVDPIGPGLDRERLYWELSQLTNSITELGPYTLDRDSLYVNGFNPWSSVPTTSTPGTSTV 1610
OVCAR5 ATGVDTICTHRVDPIGPGLDRERLYWELSQLTNSITELGPYTLDRDSLYVNGFNPRSSVPTTSTPGTSTV 1610
OV1 ATGVDTICTHRVDPIGPGLDRERLYWELSQLTNSITELGPYTLDRDSLYVNGFNPWSSVPTTSTPGTSTV 1610
OV2 ATGVDTICTHRVDPIGPGLDRERLYWELSQLTNSITELGPYTLDRDSLYVNGFNPRSSVPTTSTPGTSTV 1610
OV3 ATGVDTICTHRVDPIGPGLDRERLYWELSQLTNSITELGPYTLDRDSLYVNGFNPRSSVPTTSTPGTSTV 1610
Consensus
HLATSGTPSSLPGHTAPVPLLIPFTLNFTITNLHYEENMQHPGSRKFNTTERVLQGLLKPLFKSTSVGPL
NM_001414687.1 (35,819 .. 46,231) HLATSGTPSSLPGHTAPVPLLIPFTLNFTITNLHYEENMQHPGSRKFNTTERVLQGLLKPLFKSTSVGPL 1680
Kuramochi HLATSGTPSSLPGHTAPVPLLIPFTLNFTITNLHYEENMQHPGSRKFNTTERVLQGLLKPLFKSTSVGPL 1680
OVCAR3 HLATSGTPSSLPGHTAPVPLLIPFTLNFTITNLHYEENMQHPGSRKFNTTERVLQGLLKPLFKSTSVGPL 1680
OVCAR5 HLATSGTPSSLPGHTAPVPLLIPFTLNFTITNLHYEENMQHPGSRKFNTTERVLQGLLKPLFKSTSVGPL 1680
OV1 HLATSGTPSSLPGHTAPVPLLIPFTLNFTITNLHYEENMQHPGSRKFNTTERVLQGLLKPLFKSTSVGPL 1680
OV2 HLATSGTPSSLPGHTAPVPLLIPFTLNFTITNLHYEENMQHPGSRKFNTTERVLQGLLKPLFKSTSVGPL 1680
OV3 HLATSGTPSSLPGHTAPVPLLIPFTLNFTITNLHYEENMQHPGSRKFNTTERVLQGLLKPLFKSTSVGPL 1680
Consensus
YSGCRLTLLRPEKHGAATGVDAICTLRLDPTGPGLDRERLYWELSQLTNSVTELGPYTLDRDSLYVNGFT
NM_001414687.1 (35,819 .. 46,231) YSGCRLTLLRPEKHGAATGVDAICTLRLDPTGPGLDRERLYWELSQLTNSVTELGPYTLDRDSLYVNGFT 1750
Kuramochi YSGCRLTLLRPEKHGAATGVDAICTLRLDPTGPGLDRERLYWELSQLTNSVTELGPYTLDRDSLYVNGFT 1750
OVCAR3 YSGCRLTLLRPEKHGAATGVDAICTLRLDPTGPGLDRERLYWELSQLTNSVTELGPYTLDRDSLYVNGFT 1750
OVCAR5 YSGCRLTLLRPEKHGAATGVDAICTLRLDPTGPGLDRERLYWELSQLTNSVTELGPYTLDRDSLYVNGFT 1750
OV1 YSGCRLTLLRPEKHGAATGVDAICTLRLDPTGPGLDRERLYWELSQLTNSVTELGPYTLDRDSLYVNGFT 1750
OV2 YSGCRLTLLRPEKHGAATGVDAICTLRLDPTGPGLDRERLYWELSQLTNSVTELGPYTLDRDSLYVNGFT 1750
OV3 YSGCRLTLLRPEKHGAATGVDAICTLRLDPTGPGLDREWLYWELSQLTNSVTELGPYTLDRDSLYVNGFT 1750
Consensus
HRSSVPTTSIPGTSAVHLETSGTPASLPGHTAPGPLLVPFTLNFTITNLQYEEDMRHPGSRKFNTTERVL
NM_001414687.1 (35,819 .. 46,231) HRSSVPTTSIPGTSAVHLETSGTPASLPGHTAPGPLLVPFTLNFTITNLQYEEDMRHPGSRKFNTTERVL 1820
Kuramochi HRSSVPTTSIPGTSAVHLETSGTPASLPGHTAPGPLLVPFTLNFTITNLQYEEDMRHPGSRKFNTTERVL 1820
OVCAR3 HRSSVPTTSIPGTSAVHLETSGTPASLPGHTAPGPLLVPFTLNFTITNLQYEEDMRHPGSRKFNTTERVL 1820
OVCAR5 HRSSVPTTSIPGTSAVHLETSGTPASLPGHTAPGPLLVPFTLNFTITNLQYEEDMRHPGSRKFNTTERVL 1820
OV1 HRSSVPTTSIPGTSAVHLETSGTPASLPGHTAPGPLLVPFTLNFTITNLQYEEDMRHPGSRKFNTTERVL 1820
OV2 HRSSVPTTSIPGTSAVHLETSGTPASLPGHTAPGPLLVPFTLNFTITNLQYEEDMRHPGSRKFNTTERVL 1820
OV3 HRSSVPTTSIPGTSAVHLETSGTPASLPGHTAPGPLLVPFTLNFTITNLQYEEDMRHPGSRKFNTTERVL 1820
Consensus
QGLLKPLFKSTSVGPLYSGCRLTLLRPEKRGAATGVDTICTHRLDPLNPGLDREQLYWELSKLTRGIIEL
NM_001414687.1 (35,819 .. 46,231) QGLLKPLFKSTSVGPLYSGCRLTLLRPEKRGAATGVDTICTHRLDPLNPGLDREQLYWELSKLTRGIIEL 1890
Kuramochi QGLLKPLFKSTSVGPLYSGCRLTLLRPEKRGAATGVDTICTHRLDPLNPGLDREQLYWELSKLTRGIIEL 1890
OVCAR3 QGLLKPLFKSTSVGPLYSGCRLTLLRPEKRGAATGVDTICTHRLDPLNPGLDREQLYWELSKLTRGIIEL 1890
OVCAR5 QGLLKPLFKSTSVGPLYSGCRLTLLRPEKRGAATGVDTICTHRLDPLNPGLDREQLYWELSKLTRGIIEL 1890
OV1 QGLLKPLFKSTSVGPLYSGCRLTLLRPEKRGAATGVDTICTHRLDPLNPGLDREQLYWELSKLTRGIIEL 1890
OV2 QGLLKPLFKSTSVGPLYSGCRLTLLRPEKRGAATGVDTICTHRLDPLNPGLDREQLYWELSKLTRGIIEL 1890
OV3 QGLLKPLFKSTSVGPLYSGCRLTLLRPEKRGAATGVDTICTHRLDPLNPGLDREQLYWELSKLTHGIIEL 1890
Consensus
GPYLLDRGSLYVNGFTHRNFVPITSTPGTSTVHLGTSETPSSLPRPIVPGPLLVPFTLNFTITNLQYEEA
NM_001414687.1 (35,819 .. 46,231) GPYLLDRGSLYVNGFTHRNFVPITSTPGTSTVHLGTSETPSSLPRPIVPGPLLVPFTLNFTITNLQYEEA 1960
Kuramochi GPYLLDRGSLYVNGFTHRNFVPITSTPGTSTVHLGTSETPSSLPRPIVPGPLLVPFTLNFTITNLQYEEA 1960
OVCAR3 GPYLLDRGSLYVNGFTHRNFVPITSTPGTSTVHLGTSETPSSLPRPIVPGPLLVPFTLNFTITNLQYEEA 1960
OVCAR5 GPYLLDRGSLYVNGFTHRNFVPITSTPGTSTVHLGTSETPSSLPRPIVPGPLLVPFTLNFTITNLQYEEA 1960
OV1 GPYLLDRGSLYVNGFTHRNFVPITSTPGTSTVHLGTSETPSSLPRPIVPGPLLVPFTLNFTITNLQYEEA 1960
OV2 GPYLLDRGSLYVNGFTHRNFVPITSTPGTSTVHLGTSETPSSLPRPIVPGPLLVPFTLNFTITNLQYEEA 1960
OV3 GPYLLDRGSLYVNGFTHRNFVPITSTPGTSTVHLGTSETPSSLPRPIVPGPLLVPFTLNFTITNLQYEEA 1960

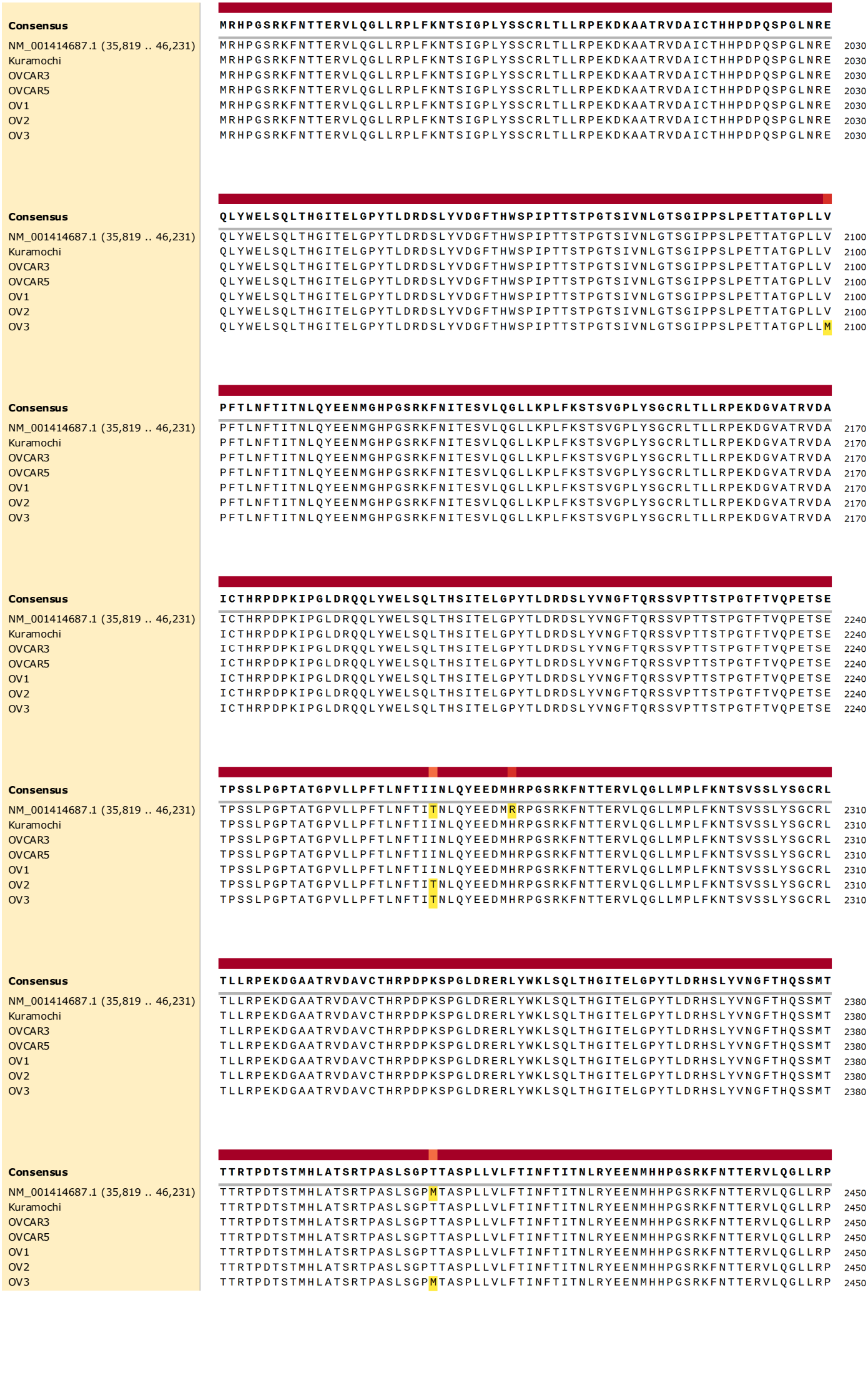
Consensus
MRHPGSRKFNTTERVLQGLLRPLFKNTSIGPLYSSCRLTLLRPEKDKAATRVDAICTHHPDPQSPGLNRE
NM_001414687.1 (35,819 .. 46,231) MRHPGSRKFNTTERVLQGLLRPLFKNTSIGPLYSSCRLTLLRPEKDKAATRVDAICTHHPDPQSPGLNRE 2030
Kuramochi MRHPGSRKFNTTERVLQGLLRPLFKNTSIGPLYSSCRLTLLRPEKDKAATRVDAICTHHPDPQSPGLNRE 2030
OVCAR3 MRHPGSRKFNTTERVLQGLLRPLFKNTSIGPLYSSCRLTLLRPEKDKAATRVDAICTHHPDPQSPGLNRE 2030
OVCAR5 MRHPGSRKFNTTERVLQGLLRPLFKNTSIGPLYSSCRLTLLRPEKDKAATRVDAICTHHPDPQSPGLNRE 2030
OV1 MRHPGSRKFNTTERVLQGLLRPLFKNTSIGPLYSSCRLTLLRPEKDKAATRVDAICTHHPDPQSPGLNRE 2030
OV2 MRHPGSRKFNTTERVLQGLLRPLFKNTSIGPLYSSCRLTLLRPEKDKAATRVDAICTHHPDPQSPGLNRE 2030
OV3 MRHPGSRKFNTTERVLQGLLRPLFKNTSIGPLYSSCRLTLLRPEKDKAATRVDAICTHHPDPQSPGLNRE 2030
Consensus
QLYWELSQLTHGITELGPYTLDRDSLYVDGFTHWSPIPTTSTPGTSIVNLGTSGIPPSLPETTATGPLLV
NM_001414687.1 (35,819 .. 46,231) QLYWELSQLTHGITELGPYTLDRDSLYVDGFTHWSPIPTTSTPGTSIVNLGTSGIPPSLPETTATGPLLV 2100
Kuramochi QLYWELSQLTHGITELGPYTLDRDSLYVDGFTHWSPIPTTSTPGTSIVNLGTSGIPPSLPETTATGPLLV 2100
OVCAR3 QLYWELSQLTHGITELGPYTLDRDSLYVDGFTHWSPIPTTSTPGTSIVNLGTSGIPPSLPETTATGPLLV 2100
OVCAR5 QLYWELSQLTHGITELGPYTLDRDSLYVDGFTHWSPIPTTSTPGTSIVNLGTSGIPPSLPETTATGPLLV 2100
OV1 QLYWELSQLTHGITELGPYTLDRDSLYVDGFTHWSPIPTTSTPGTSIVNLGTSGIPPSLPETTATGPLLV 2100
OV2 QLYWELSQLTHGITELGPYTLDRDSLYVDGFTHWSPIPTTSTPGTSIVNLGTSGIPPSLPETTATGPLLV 2100
OV3 QLYWELSQLTHGITELGPYTLDRDSLYVDGFTHWSPIPTTSTPGTSIVNLGTSGIPPSLPETTATGPLLM 2100
Consensus
PFTLNFTITNLQYEENMGHPGSRKFNITESVLQGLLKPLFKSTSVGPLYSGCRLTLLRPEKDGVATRVDA
NM_001414687.1 (35,819 .. 46,231) PFTLNFTITNLQYEENMGHPGSRKFNITESVLQGLLKPLFKSTSVGPLYSGCRLTLLRPEKDGVATRVDA 2170
Kuramochi PFTLNFTITNLQYEENMGHPGSRKFNITESVLQGLLKPLFKSTSVGPLYSGCRLTLLRPEKDGVATRVDA 2170
OVCAR3 PFTLNFTITNLQYEENMGHPGSRKFNITESVLQGLLKPLFKSTSVGPLYSGCRLTLLRPEKDGVATRVDA 2170
OVCAR5 PFTLNFTITNLQYEENMGHPGSRKFNITESVLQGLLKPLFKSTSVGPLYSGCRLTLLRPEKDGVATRVDA 2170
OV1 PFTLNFTITNLQYEENMGHPGSRKFNITESVLQGLLKPLFKSTSVGPLYSGCRLTLLRPEKDGVATRVDA 2170
OV2 PFTLNFTITNLQYEENMGHPGSRKFNITESVLQGLLKPLFKSTSVGPLYSGCRLTLLRPEKDGVATRVDA 2170
OV3 PFTLNFTITNLQYEENMGHPGSRKFNITESVLQGLLKPLFKSTSVGPLYSGCRLTLLRPEKDGVATRVDA 2170
Consensus
ICTHRPDPKIPGLDRQQLYWELSQLTHSITELGPYTLDRDSLYVNGFTQRSSVPTTSTPGTFTVQPETSE
NM_001414687.1 (35,819 .. 46,231) ICTHRPDPKIPGLDRQQLYWELSQLTHSITELGPYTLDRDSLYVNGFTQRSSVPTTSTPGTFTVQPETSE 2240
Kuramochi ICTHRPDPKIPGLDRQQLYWELSQLTHSITELGPYTLDRDSLYVNGFTQRSSVPTTSTPGTFTVQPETSE 2240
OVCAR3 ICTHRPDPKIPGLDRQQLYWELSQLTHSITELGPYTLDRDSLYVNGFTQRSSVPTTSTPGTFTVQPETSE 2240
OVCAR5 ICTHRPDPKIPGLDRQQLYWELSQLTHSITELGPYTLDRDSLYVNGFTQRSSVPTTSTPGTFTVQPETSE 2240
OV1 ICTHRPDPKIPGLDRQQLYWELSQLTHSITELGPYTLDRDSLYVNGFTQRSSVPTTSTPGTFTVQPETSE 2240
OV2 ICTHRPDPKIPGLDRQQLYWELSQLTHSITELGPYTLDRDSLYVNGFTQRSSVPTTSTPGTFTVQPETSE 2240
OV3 ICTHRPDPKIPGLDRQQLYWELSQLTHSITELGPYTLDRDSLYVNGFTQRSSVPTTSTPGTFTVQPETSE 2240
Consensus
TPSSLPGPTATGPVLLPFTLNFTIINLQYEEDMHRPGSRKFNTTERVLQGLLMPLFKNTSVSSLYSGCRL
NM_001414687.1 (35,819 .. 46,231) TPSSLPGPTATGPVLLPFTLNFTITNLQYEEDMRRPGSRKFNTTERVLQGLLMPLFKNTSVSSLYSGCRL 2310
Kuramochi TPSSLPGPTATGPVLLPFTLNFTIINLQYEEDMHRPGSRKFNTTERVLQGLLMPLFKNTSVSSLYSGCRL 2310
OVCAR3 TPSSLPGPTATGPVLLPFTLNFTIINLQYEEDMHRPGSRKFNTTERVLQGLLMPLFKNTSVSSLYSGCRL 2310
OVCAR5 TPSSLPGPTATGPVLLPFTLNFTIINLQYEEDMHRPGSRKFNTTERVLQGLLMPLFKNTSVSSLYSGCRL 2310
OV1 TPSSLPGPTATGPVLLPFTLNFTIINLQYEEDMHRPGSRKFNTTERVLQGLLMPLFKNTSVSSLYSGCRL 2310
OV2 TPSSLPGPTATGPVLLPFTLNFTITNLQYEEDMHRPGSRKFNTTERVLQGLLMPLFKNTSVSSLYSGCRL 2310
OV3 TPSSLPGPTATGPVLLPFTLNFTITNLQYEEDMHRPGSRKFNTTERVLQGLLMPLFKNTSVSSLYSGCRL 2310
Consensus
TLLRPEKDGAATRVDAVCTHRPDPKSPGLDRERLYWKLSQLTHGITELGPYTLDRHSLYVNGFTHQSSMT
NM_001414687.1 (35,819 .. 46,231) TLLRPEKDGAATRVDAVCTHRPDPKSPGLDRERLYWKLSQLTHGITELGPYTLDRHSLYVNGFTHQSSMT 2380
Kuramochi TLLRPEKDGAATRVDAVCTHRPDPKSPGLDRERLYWKLSQLTHGITELGPYTLDRHSLYVNGFTHQSSMT 2380
OVCAR3 TLLRPEKDGAATRVDAVCTHRPDPKSPGLDRERLYWKLSQLTHGITELGPYTLDRHSLYVNGFTHQSSMT 2380
OVCAR5 TLLRPEKDGAATRVDAVCTHRPDPKSPGLDRERLYWKLSQLTHGITELGPYTLDRHSLYVNGFTHQSSMT 2380
OV1 TLLRPEKDGAATRVDAVCTHRPDPKSPGLDRERLYWKLSQLTHGITELGPYTLDRHSLYVNGFTHQSSMT 2380
OV2 TLLRPEKDGAATRVDAVCTHRPDPKSPGLDRERLYWKLSQLTHGITELGPYTLDRHSLYVNGFTHQSSMT 2380
OV3 TLLRPEKDGAATRVDAVCTHRPDPKSPGLDRERLYWKLSQLTHGITELGPYTLDRHSLYVNGFTHQSSMT 2380
Consensus
TTRTPDTSTMHLATSRTPASLSGPTTASPLLVLFTINFTITNLRYEENMHHPGSRKFNTTERVLQGLLRP
NM_001414687.1 (35,819 .. 46,231) TTRTPDTSTMHLATSRTPASLSGPMTASPLLVLFTINFTITNLRYEENMHHPGSRKFNTTERVLQGLLRP 2450
Kuramochi TTRTPDTSTMHLATSRTPASLSGPTTASPLLVLFTINFTITNLRYEENMHHPGSRKFNTTERVLQGLLRP 2450
OVCAR3 TTRTPDTSTMHLATSRTPASLSGPTTASPLLVLFTINFTITNLRYEENMHHPGSRKFNTTERVLQGLLRP 2450
OVCAR5 TTRTPDTSTMHLATSRTPASLSGPTTASPLLVLFTINFTITNLRYEENMHHPGSRKFNTTERVLQGLLRP 2450
OV1 TTRTPDTSTMHLATSRTPASLSGPTTASPLLVLFTINFTITNLRYEENMHHPGSRKFNTTERVLQGLLRP 2450
OV2 TTRTPDTSTMHLATSRTPASLSGPTTASPLLVLFTINFTITNLRYEENMHHPGSRKFNTTERVLQGLLRP 2450
OV3 TTRTPDTSTMHLATSRTPASLSGPMTASPLLVLFTINFTITNLRYEENMHHPGSRKFNTTERVLQGLLRP 2450

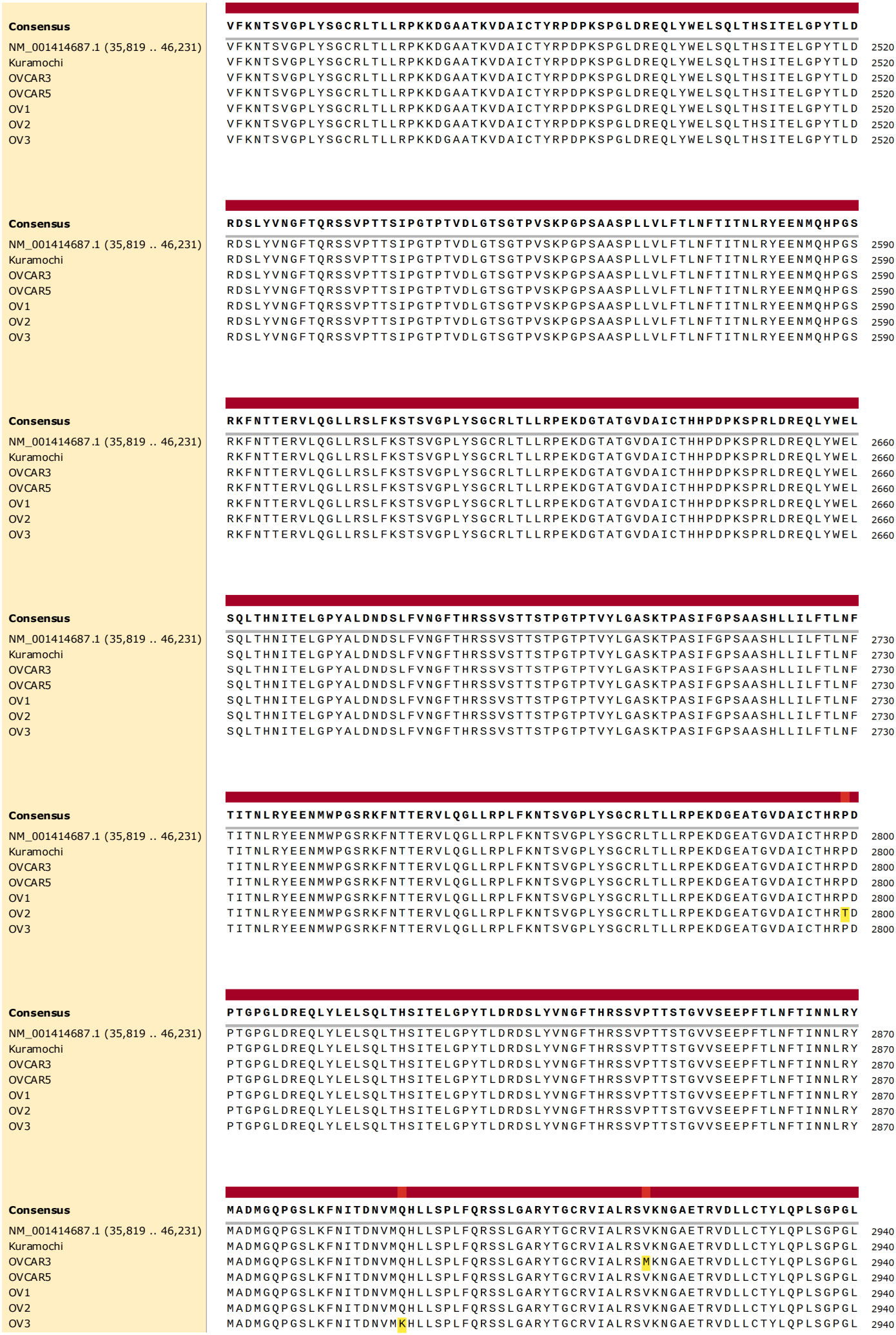
Consensus
VFKNTSVGPLYSGCRLTLLRPKKDGAATKVDAICTYRPDPKSPGLDREQLYWELSQLTHSITELGPYTLD
NM_001414687.1 (35,819 .. 46,231)
Kuramochi
OVCAR3
OVCAR5
OV1
OV2
OV3
VFKNTSVGPLYSGCRLTLLRPKKDGAATKVDAICTYRPDPKSPGLDREQLYWELSQLTHSITELGPYTLD 2520
Consensus
RDSLYVNGFTQRSSVPTTSIPGTPTVDLGTSGTPVSKPGPSAASPLLVLFTLNFTITNLRYEENMQHPGS
NM_001414687.1 (35,819 .. 46,231)
Kuramochi
OVCAR3
OVCAR5
OV1
OV2
OV3
RDSLYVNGFTQRSSVPTTSIPGTPTVDLGTSGTPVSKPGPSAASPLLVLFTLNFTITNLRYEENMQHPGS 2590
Consensus
RKFNTTERVLQGLLRSLFKSTSVGPLYSGCRLTLLRPEKDGTATGVDAICTHHPDPKSPRLDREQLYWEL
NM_001414687.1 (35,819 .. 46,231)
Kuramochi
OVCAR3
OVCAR5
OV1
OV2
OV3
RKFNTTERVLQGLLRSLFKSTSVGPLYSGCRLTLLRPEKDGTATGVDAICTHHPDPKSPRLDREQLYWEL 2660
Consensus
SQLTHNITELGPYALDNDSLFVNGFTHRSSVSTTSTPGTPTVYLGASKTPASIFGPSAASHLLILFTLNF
NM_001414687.1 (35,819 .. 46,231)
Kuramochi
OVCAR3
OVCAR5
OV1
OV2
OV3
SQLTHNITELGPYALDNDSLFVNGFTHRSSVSTTSTPGTPTVYLGASKTPASIFGPSAASHLLILFTLNF 2730
Consensus
TITNLRYEENMWPGSRKFNTTERVLQGLLRPLFKNTSVGPLYSGCRLTLLRPEKDGEATGVDAICTHRPD
NM_001414687.1 (35,819 .. 46,231)
Kuramochi
OVCAR3
OVCAR5
OV1
OV2
OV3
TITNLRYEENMWPGSRKFNTTERVLQGLLRPLFKNTSVGPLYSGCRLTLLRPEKDGEATGVDAICTHRPD 2800
Consensus
PTGPGLDREQLYLELSQLTHSITELGPYTLDRDSLYVNGFTHRSSVPTTSTGVVSEEPFTLNFTINNLRY
NM_001414687.1 (35,819 .. 46,231)
Kuramochi
OVCAR3
OVCAR5
OV1
OV2
OV3
PTGPGLDREQLYLELSQLTHSITELGPYTLDRDSLYVNGFTHRSSVPTTSTGVVSEEPFTLNFTINNLRY 2870
Consensus
MADMGQPGSLKFNITDNVMQHLLSPLFQRSSLGARYTGCRVIALRSVKNGAETRVDLLCTYLQPLSGPGL
NM_001414687.1 (35,819 .. 46,231)
Kuramochi
OVCAR3
OVCAR5
OV1
OV2
OV3
MADMGQPGSLKFNITDNVMQHLLSPLFQRSSLGARYTGCRVIALRSVKNGAETRVDLLCTYLQPLSGPGL 2940

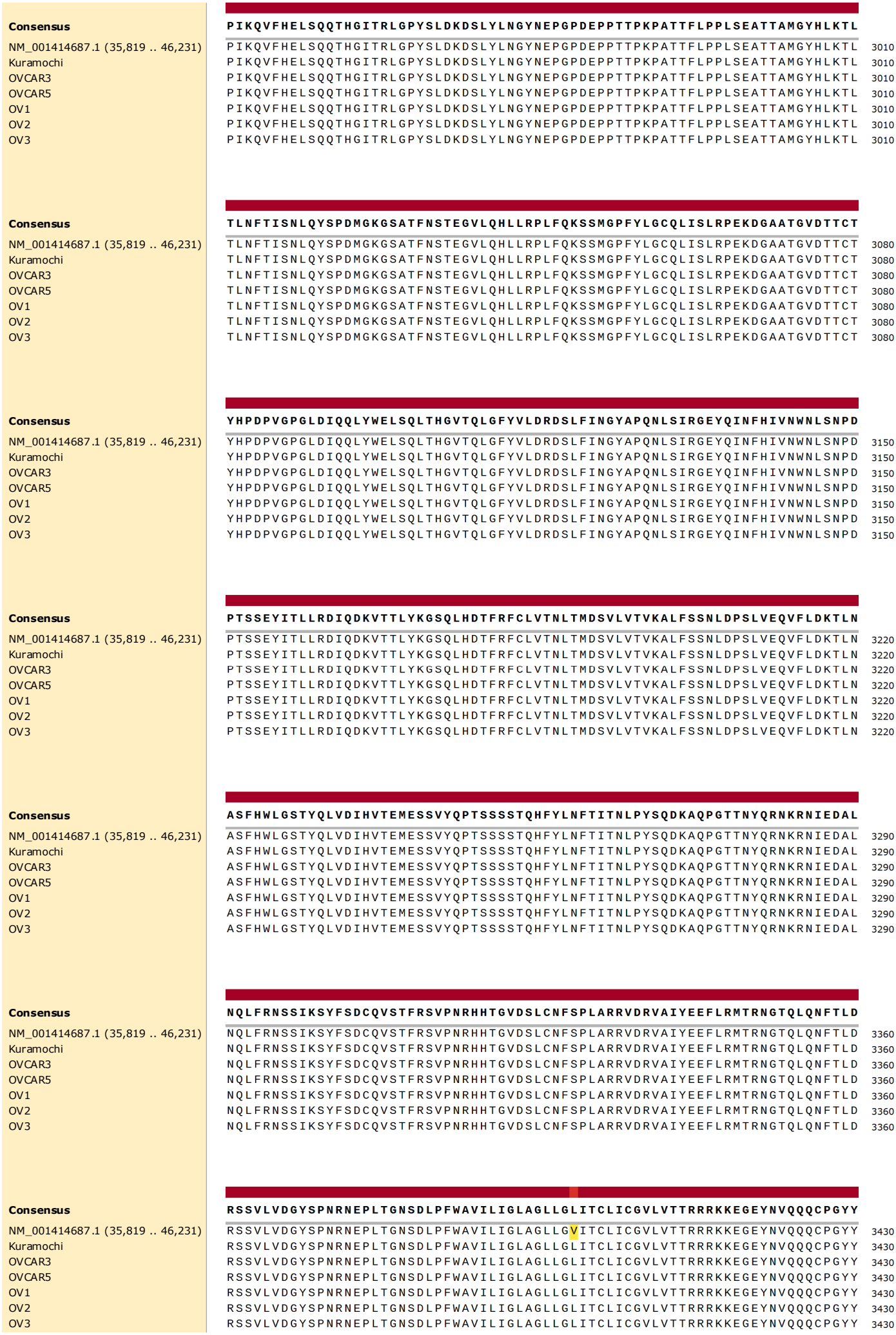
Consensus
PIKQVFHELSQQTHGITRLGPYSLDKDSLYLNGYNEPGPDEPPTTPKPATTFLPPLSEATTAMGYHLKTL
NM_001414687.1 (35,819 .. 46,231) PIKQVFHELSQQTHGITRLGPYSLDKDSLYLNGYNEPGPDEPPTTPKPATTFLPPLSEATTAMGYHLKTL 3010
Kuramochi PIKQVFHELSQQTHGITRLGPYSLDKDSLYLNGYNEPGPDEPPTTPKPATTFLPPLSEATTAMGYHLKTL 3010
OVCAR3 PIKQVFHELSQQTHGITRLGPYSLDKDSLYLNGYNEPGPDEPPTTPKPATTFLPPLSEATTAMGYHLKTL 3010
OVCAR5 PIKQVFHELSQQTHGITRLGPYSLDKDSLYLNGYNEPGPDEPPTTPKPATTFLPPLSEATTAMGYHLKTL 3010
OV1 PIKQVFHELSQQTHGITRLGPYSLDKDSLYLNGYNEPGPDEPPTTPKPATTFLPPLSEATTAMGYHLKTL 3010
OV2 PIKQVFHELSQQTHGITRLGPYSLDKDSLYLNGYNEPGPDEPPTTPKPATTFLPPLSEATTAMGYHLKTL 3010
OV3 PIKQVFHELSQQTHGITRLGPYSLDKDSLYLNGYNEPGPDEPPTTPKPATTFLPPLSEATTAMGYHLKTL 3010
Consensus
TLNFTISNLQYSPDMGKGSATFNSTEGVLQHLLRPLFQKSSMGPFYLGCQLISLRPEKDGAATGVDTTCT
NM_001414687.1 (35,819 .. 46,231) TLNFTISNLQYSPDMGKGSATFNSTEGVLQHLLRPLFQKSSMGPFYLGCQLISLRPEKDGAATGVDTTCT 3080
Kuramochi TLNFTISNLQYSPDMGKGSATFNSTEGVLQHLLRPLFQKSSMGPFYLGCQLISLRPEKDGAATGVDTTCT 3080
OVCAR3 TLNFTISNLQYSPDMGKGSATFNSTEGVLQHLLRPLFQKSSMGPFYLGCQLISLRPEKDGAATGVDTTCT 3080
OVCAR5 TLNFTISNLQYSPDMGKGSATFNSTEGVLQHLLRPLFQKSSMGPFYLGCQLISLRPEKDGAATGVDTTCT 3080
OV1 TLNFTISNLQYSPDMGKGSATFNSTEGVLQHLLRPLFQKSSMGPFYLGCQLISLRPEKDGAATGVDTTCT 3080
OV2 TLNFTISNLQYSPDMGKGSATFNSTEGVLQHLLRPLFQKSSMGPFYLGCQLISLRPEKDGAATGVDTTCT 3080
OV3 TLNFTISNLQYSPDMGKGSATFNSTEGVLQHLLRPLFQKSSMGPFYLGCQLISLRPEKDGAATGVDTTCT 3080
Consensus
YHPDPVGPGLDIQQLYWELSQLTHGVTQLGFYVLDRDSLFINGYAPQNLSIRGEYQINFHIVNWNLSNPD
NM_001414687.1 (35,819 .. 46,231) YHPDPVGPGLDIQQLYWELSQLTHGVTQLGFYVLDRDSLFINGYAPQNLSIRGEYQINFHIVNWNLSNPD 3150
Kuramochi YHPDPVGPGLDIQQLYWELSQLTHGVTQLGFYVLDRDSLFINGYAPQNLSIRGEYQINFHIVNWNLSNPD 3150
OVCAR3 YHPDPVGPGLDIQQLYWELSQLTHGVTQLGFYVLDRDSLFINGYAPQNLSIRGEYQINFHIVNWNLSNPD 3150
OVCAR5 YHPDPVGPGLDIQQLYWELSQLTHGVTQLGFYVLDRDSLFINGYAPQNLSIRGEYQINFHIVNWNLSNPD 3150
OV1 YHPDPVGPGLDIQQLYWELSQLTHGVTQLGFYVLDRDSLFINGYAPQNLSIRGEYQINFHIVNWNLSNPD 3150
OV2 YHPDPVGPGLDIQQLYWELSQLTHGVTQLGFYVLDRDSLFINGYAPQNLSIRGEYQINFHIVNWNLSNPD 3150
OV3 YHPDPVGPGLDIQQLYWELSQLTHGVTQLGFYVLDRDSLFINGYAPQNLSIRGEYQINFHIVNWNLSNPD 3150
Consensus
PTSSEYITLLRDIQDKVTTLYKGSQLHDTFRFCLVTNLTMDSVLVTVKALFSSNLDPSLVEQVFLDKTLN
NM_001414687.1 (35,819 .. 46,231) PTSSEYITLLRDIQDKVTTLYKGSQLHDTFRFCLVTNLTMDSVLVTVKALFSSNLDPSLVEQVFLDKTLN 3220
Kuramochi PTSSEYITLLRDIQDKVTTLYKGSQLHDTFRFCLVTNLTMDSVLVTVKALFSSNLDPSLVEQVFLDKTLN 3220
OVCAR3 PTSSEYITLLRDIQDKVTTLYKGSQLHDTFRFCLVTNLTMDSVLVTVKALFSSNLDPSLVEQVFLDKTLN 3220
OVCAR5 PTSSEYITLLRDIQDKVTTLYKGSQLHDTFRFCLVTNLTMDSVLVTVKALFSSNLDPSLVEQVFLDKTLN 3220
OV1 PTSSEYITLLRDIQDKVTTLYKGSQLHDTFRFCLVTNLTMDSVLVTVKALFSSNLDPSLVEQVFLDKTLN 3220
OV2 PTSSEYITLLRDIQDKVTTLYKGSQLHDTFRFCLVTNLTMDSVLVTVKALFSSNLDPSLVEQVFLDKTLN 3220
OV3 PTSSEYITLLRDIQDKVTTLYKGSQLHDTFRFCLVTNLTMDSVLVTVKALFSSNLDPSLVEQVFLDKTLN 3220
Consensus
ASFHWLGSTYQLVDIHVTEMESSVYQPTSSSSTQHFYLNFTITNLPYSQDKAQPGTTNYQRNKRNIEDAL
NM_001414687.1 (35,819 .. 46,231) ASFHWLGSTYQLVDIHVTEMESSVYQPTSSSSTQHFYLNFTITNLPYSQDKAQPGTTNYQRNKRNIEDAL 3290
Kuramochi ASFHWLGSTYQLVDIHVTEMESSVYQPTSSSSTQHFYLNFTITNLPYSQDKAQPGTTNYQRNKRNIEDAL 3290
OVCAR3 ASFHWLGSTYQLVDIHVTEMESSVYQPTSSSSTQHFYLNFTITNLPYSQDKAQPGTTNYQRNKRNIEDAL 3290
OVCAR5 ASFHWLGSTYQLVDIHVTEMESSVYQPTSSSSTQHFYLNFTITNLPYSQDKAQPGTTNYQRNKRNIEDAL 3290
OV1 ASFHWLGSTYQLVDIHVTEMESSVYQPTSSSSTQHFYLNFTITNLPYSQDKAQPGTTNYQRNKRNIEDAL 3290
OV2 ASFHWLGSTYQLVDIHVTEMESSVYQPTSSSSTQHFYLNFTITNLPYSQDKAQPGTTNYQRNKRNIEDAL 3290
OV3 ASFHWLGSTYQLVDIHVTEMESSVYQPTSSSSTQHFYLNFTITNLPYSQDKAQPGTTNYQRNKRNIEDAL 3290
Consensus
NQLFRNSSIKSYFSDCQVSTFRSVPNRHHTGVDSLCNFSPLARRVDRVAIYEEFLRMTRNGTQLQNFTLD
NM_001414687.1 (35,819 .. 46,231) NQLFRNSSIKSYFSDCQVSTFRSVPNRHHTGVDSLCNFSPLARRVDRVAIYEEFLRMTRNGTQLQNFTLD 3360
Kuramochi NQLFRNSSIKSYFSDCQVSTFRSVPNRHHTGVDSLCNFSPLARRVDRVAIYEEFLRMTRNGTQLQNFTLD 3360
OVCAR3 NQLFRNSSIKSYFSDCQVSTFRSVPNRHHTGVDSLCNFSPLARRVDRVAIYEEFLRMTRNGTQLQNFTLD 3360
OVCAR5 NQLFRNSSIKSYFSDCQVSTFRSVPNRHHTGVDSLCNFSPLARRVDRVAIYEEFLRMTRNGTQLQNFTLD 3360
OV1 NQLFRNSSIKSYFSDCQVSTFRSVPNRHHTGVDSLCNFSPLARRVDRVAIYEEFLRMTRNGTQLQNFTLD 3360
OV2 NQLFRNSSIKSYFSDCQVSTFRSVPNRHHTGVDSLCNFSPLARRVDRVAIYEEFLRMTRNGTQLQNFTLD 3360
OV3 NQLFRNSSIKSYFSDCQVSTFRSVPNRHHTGVDSLCNFSPLARRVDRVAIYEEFLRMTRNGTQLQNFTLD 3360
Consensus
RSSVLVDGYSPNRNEPLTGNSDLPFWAVILIGLAGLLGLITCLICGVLVTTRRRKKEGEYNVQQQCPGYY
NM_001414687.1 (35,819 .. 46,231) RSSVLVDGYSPNRNEPLTGNSDLPFWAVILIGLAGLLGVITCLICGVLVTTRRRKKEGEYNVQQQCPGYY 3430
Kuramochi RSSVLVDGYSPNRNEPLTGNSDLPFWAVILIGLAGLLGLITCLICGVLVTTRRRKKEGEYNVQQQCPGYY 3430
OVCAR3 RSSVLVDGYSPNRNEPLTGNSDLPFWAVILIGLAGLLGLITCLICGVLVTTRRRKKEGEYNVQQQCPGYY 3430
OVCAR5 RSSVLVDGYSPNRNEPLTGNSDLPFWAVILIGLAGLLGLITCLICGVLVTTRRRKKEGEYNVQQQCPGYY 3430
OV1 RSSVLVDGYSPNRNEPLTGNSDLPFWAVILIGLAGLLGLITCLICGVLVTTRRRKKEGEYNVQQQCPGYY 3430
OV2 RSSVLVDGYSPNRNEPLTGNSDLPFWAVILIGLAGLLGLITCLICGVLVTTRRRKKEGEYNVQQQCPGYY 3430
OV3 RSSVLVDGYSPNRNEPLTGNSDLPFWAVILIGLAGLLGLITCLICGVLVTTRRRKKEGEYNVQQQCPGYY 3430

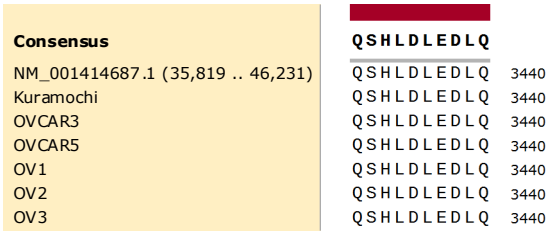
Consensus
NM_001414687.1 (35,819 .. 46,231)
Kuramochi
OVCAR3
OVCAR5
OV1
OV2
OV3
Q S H L D L E D L Q
Q S H L D L E D L Q 3440
Q S H L D L E D L Q 3440
Q S H L D L E D L Q 3440
Q S H L D L E D L Q 3440
Q S H L D L E D L Q 3440
Q S H L D L E D L Q 3440
Q S H L D L E D L Q 3440
