# Supplementary material for: A Revised Molecular Model of Ovarian Cancer Biomarker CA125 (MUC16) Enabled by Long-read Sequencing
Source: Cancer Res Commun. 2024 Jan 31;4(1):253–63. doi: 10.1158/2767-9764.CRC-23-0327 (PMC10829539; doi:10.1158/2767-9764.CRC-23-0327)
Supplement: Supplementary Document 4 — Mutation table [file crc-23-0327-s08.pdf]

Sample	Amino acid number in mucin-16 isoform 3 (NP_001401616.1)	Substitution amino acid	Change in N-glycosilation site	Repeat number	Position in the repeat
OV1	S12536	T	N	3	133
	A13216	T	N	8	34
	Q13407	H	N	9	69
	V13444	I	N	9	106
	T14134	I	N	14	16
	R14143	H	N	14	25
	M14274	T	N	14	156
OV2	S12536	T	N	3	133
	T12162	M	N	1	73
	E12312	K	N	2	67
	A13216	T	N	8	34
	W13279	C	N	8	97
	T13382	K	N	9	44
	Q13407	H	N	9	69
	V13444	I	N	9	106
	W13465	R	N	9	127
	R14143	H	N	14	25
	M14274	T	N	14	156
	P14668	T	N	17	82
OV3	S12536	T	N	3	133
	A13216	T	N	8	34
	W13279	C	N	8	97
	T13382	K	N	9	44
	Q13407	H	N	9	69
	V13444	I	N	9	106
	W13465	R	N	9	127
	R12588	W	N	4	29
	R13754	H	N	11	104
	V13969	M	N	13	7
	R14143	H	N	14	25
	Q14759	K	N	18	17

Kuramochi	S12536	T	N	3	133
	L12828	P	N	5	114
	V13444	I	N	9	106
	T14134	I	N	14	16
	R14143	H	N	14	25
	M14274	T	N	14	156
OVCAR3	S12536	T	N	3	133
	R13142	W	N	7	116
	V13444	I	N	9	106
	T14134	I	N	14	16
	R14143	H	N	14	25
	M14274	T	N	14	156
	V14786	M	N	18	44
OVCAR5	R12216	Q	N	1	127
	S12536	T	N	3	133
	E12963	K	N	6	93
	A13216	T	N	8	34
	W13279	C	N	8	97
	T13382	K	N	9	44
	Q13407	H	N	9	69
	V13444	I	N	9	106
	W13465	R	N	9	127
	T14134	I	N	14	16
	R14143	H	N	14	25
	M14274	T	N	14	156
