# Supplementary material for: A Revised Molecular Model of Ovarian Cancer Biomarker CA125 (MUC16) Enabled by Long-read Sequencing
Source: Cancer Res Commun. 2024 Jan 31;4(1):253–63. doi: 10.1158/2767-9764.CRC-23-0327 (PMC10829539; doi:10.1158/2767-9764.CRC-23-0327)
Supplement: Figure S7 — AlphaFold predicted models of individual tandem repeats. (A)-(S) Repeats 1-19. [file crc-23-0327-s11.pdf]

Figure S7 (A) - (S)

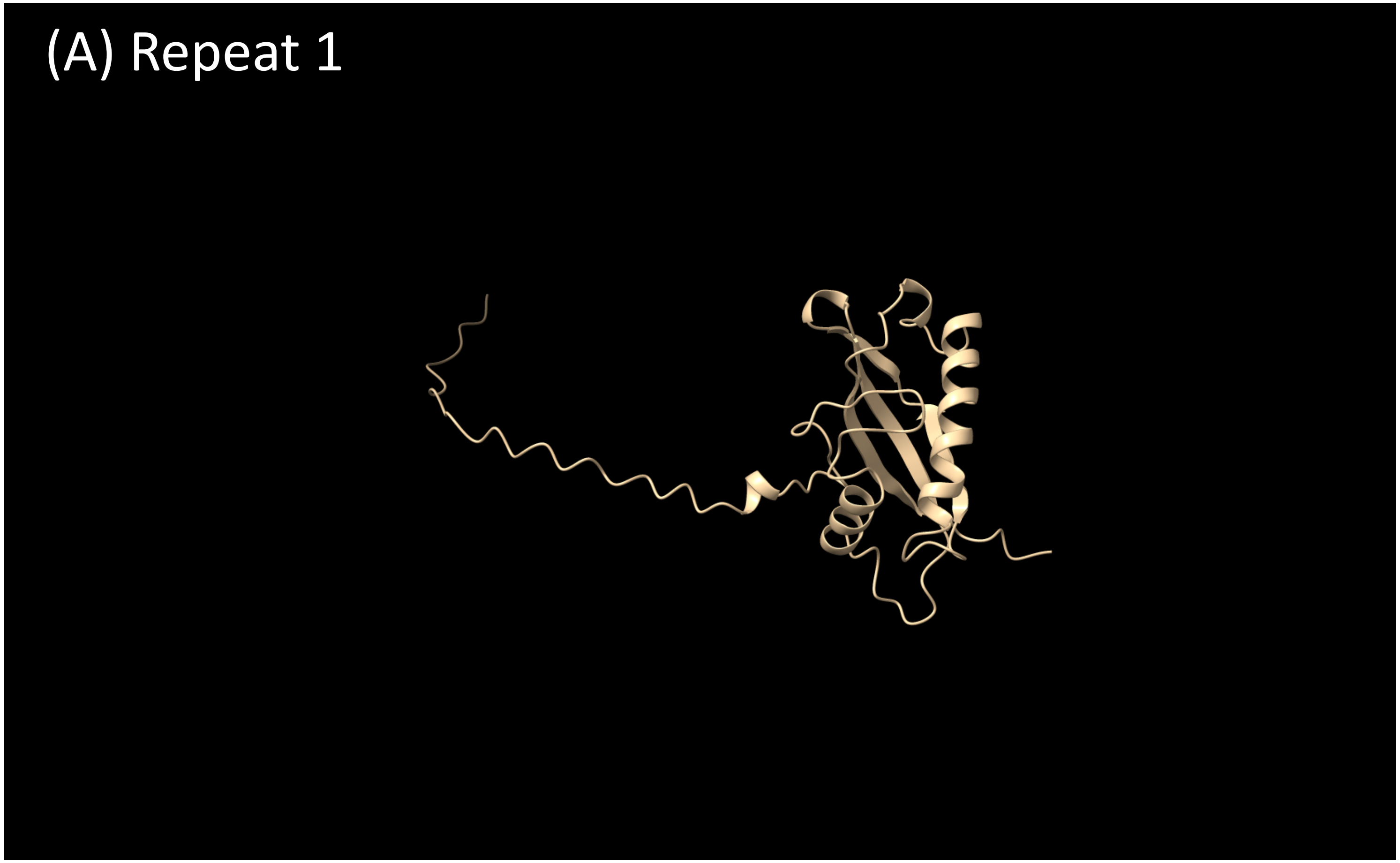

(B) Repeat 2

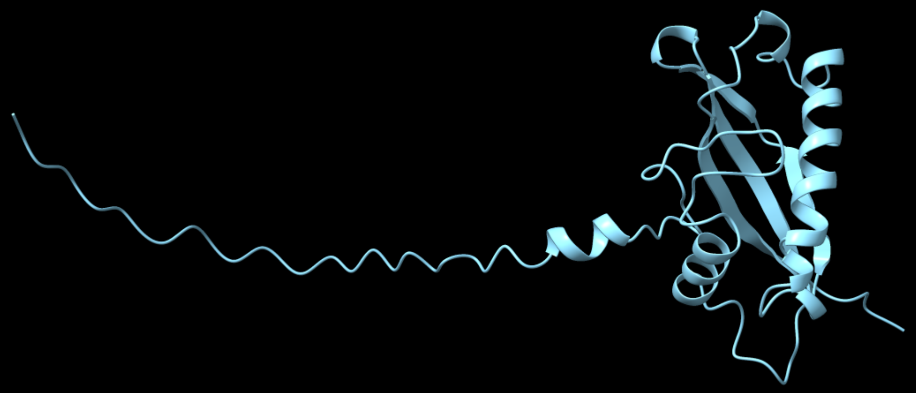

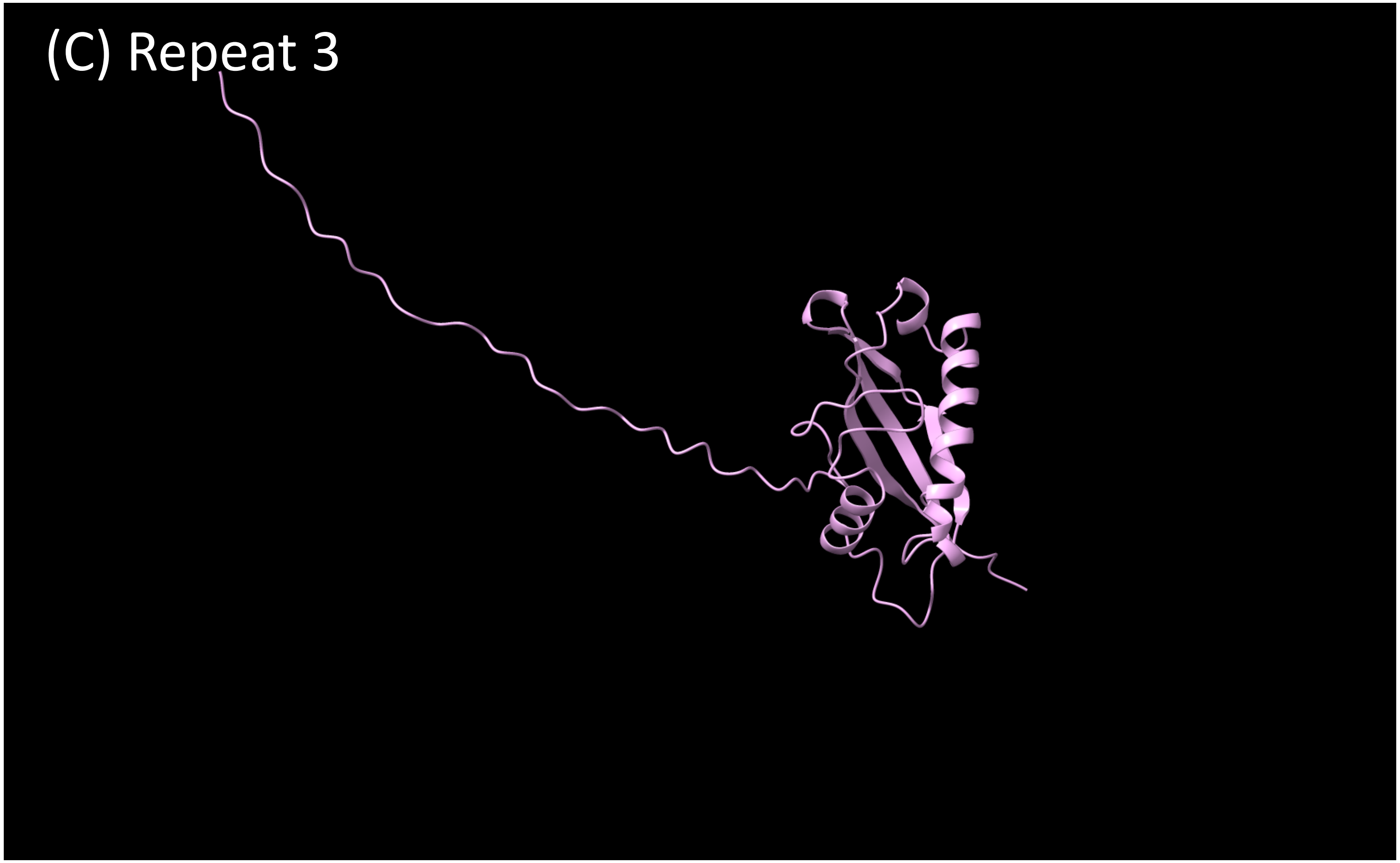
(C) Repeat 3

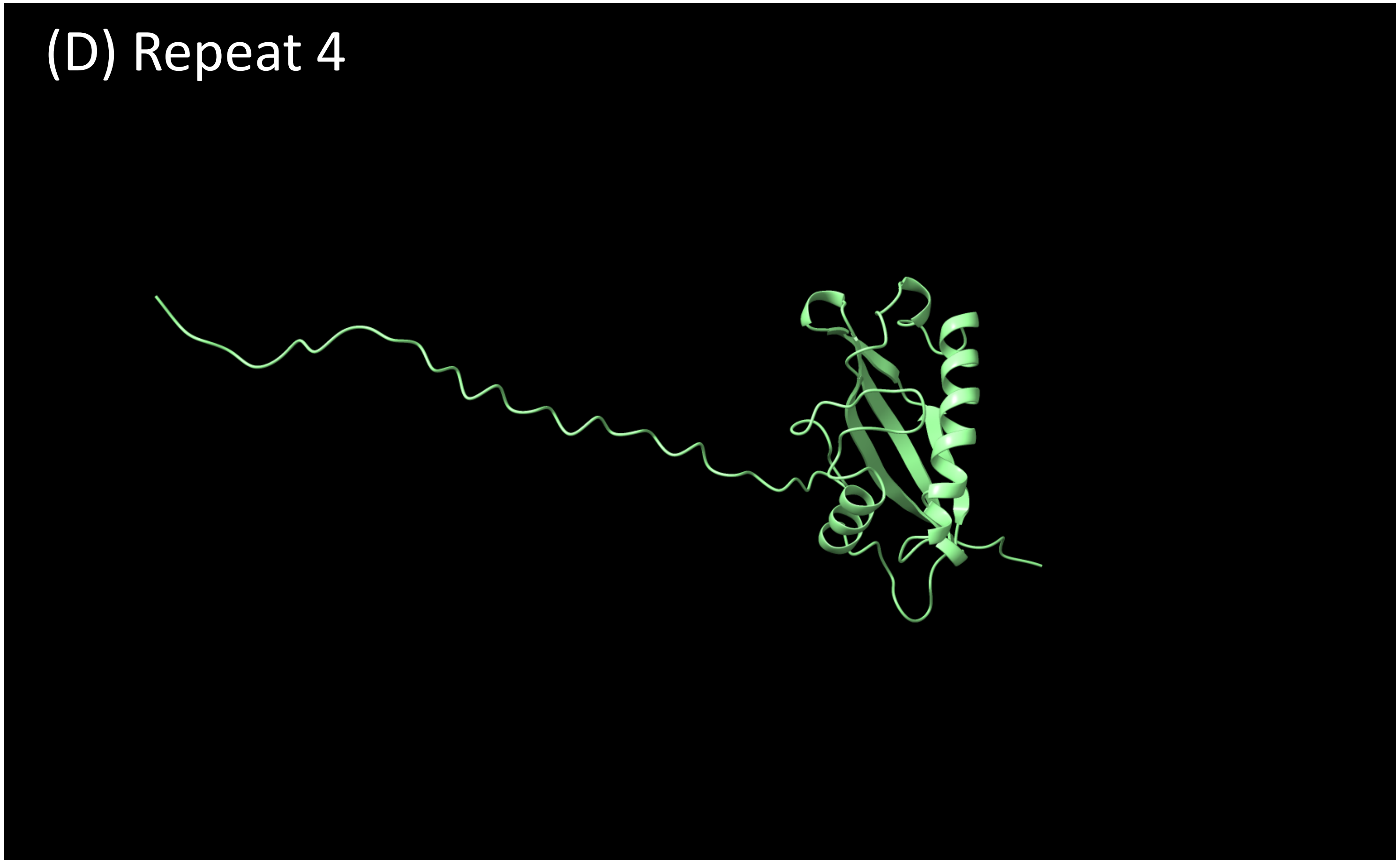
(D) Repeat 4

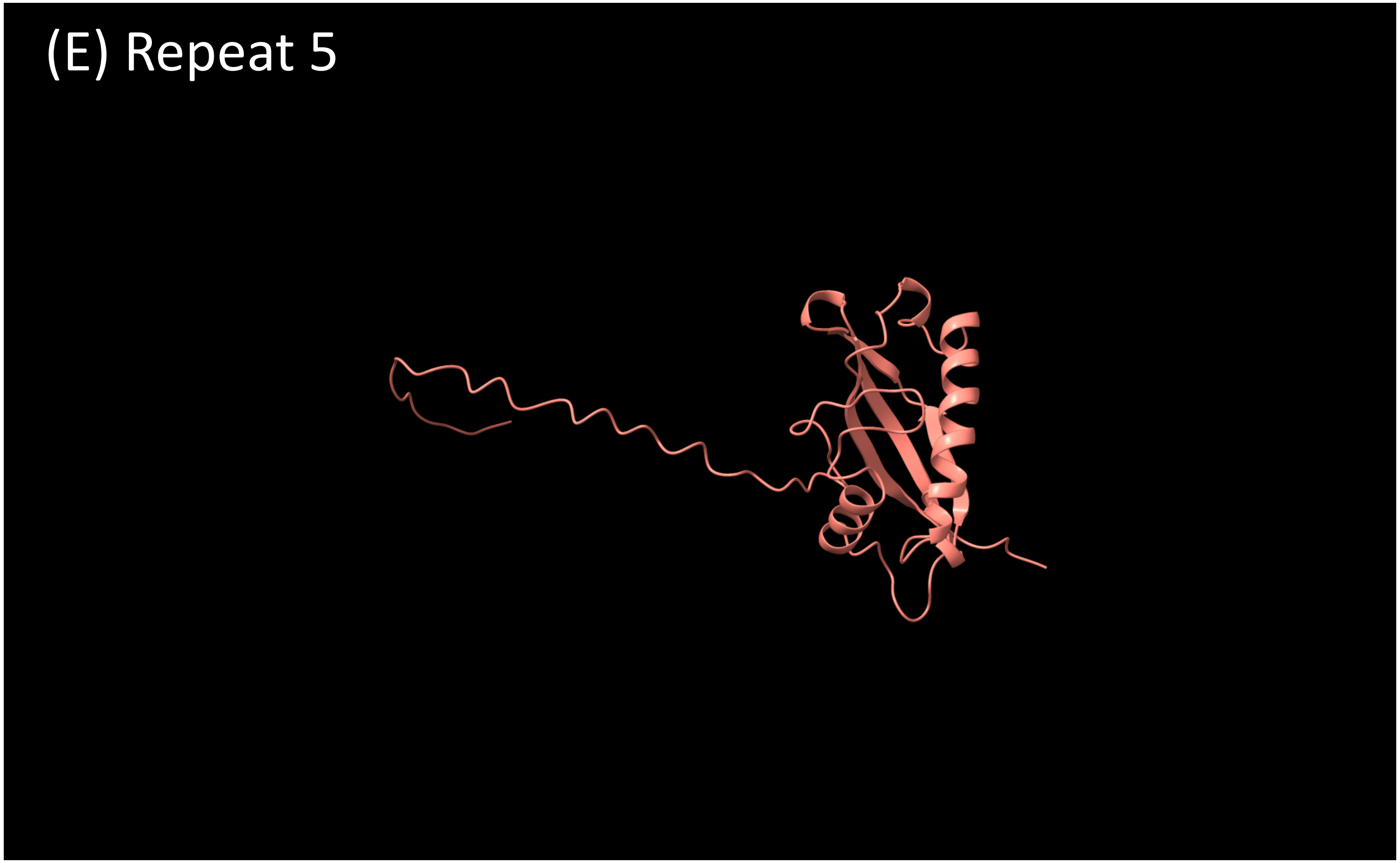
(E) Repeat 5

(F) Repeat 6

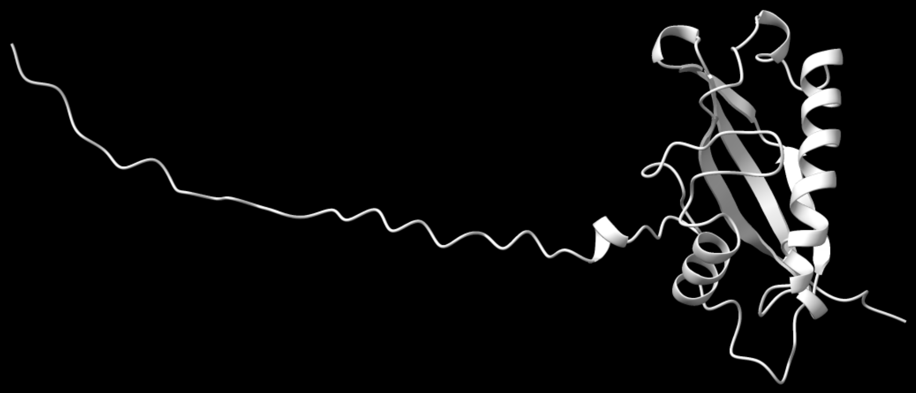

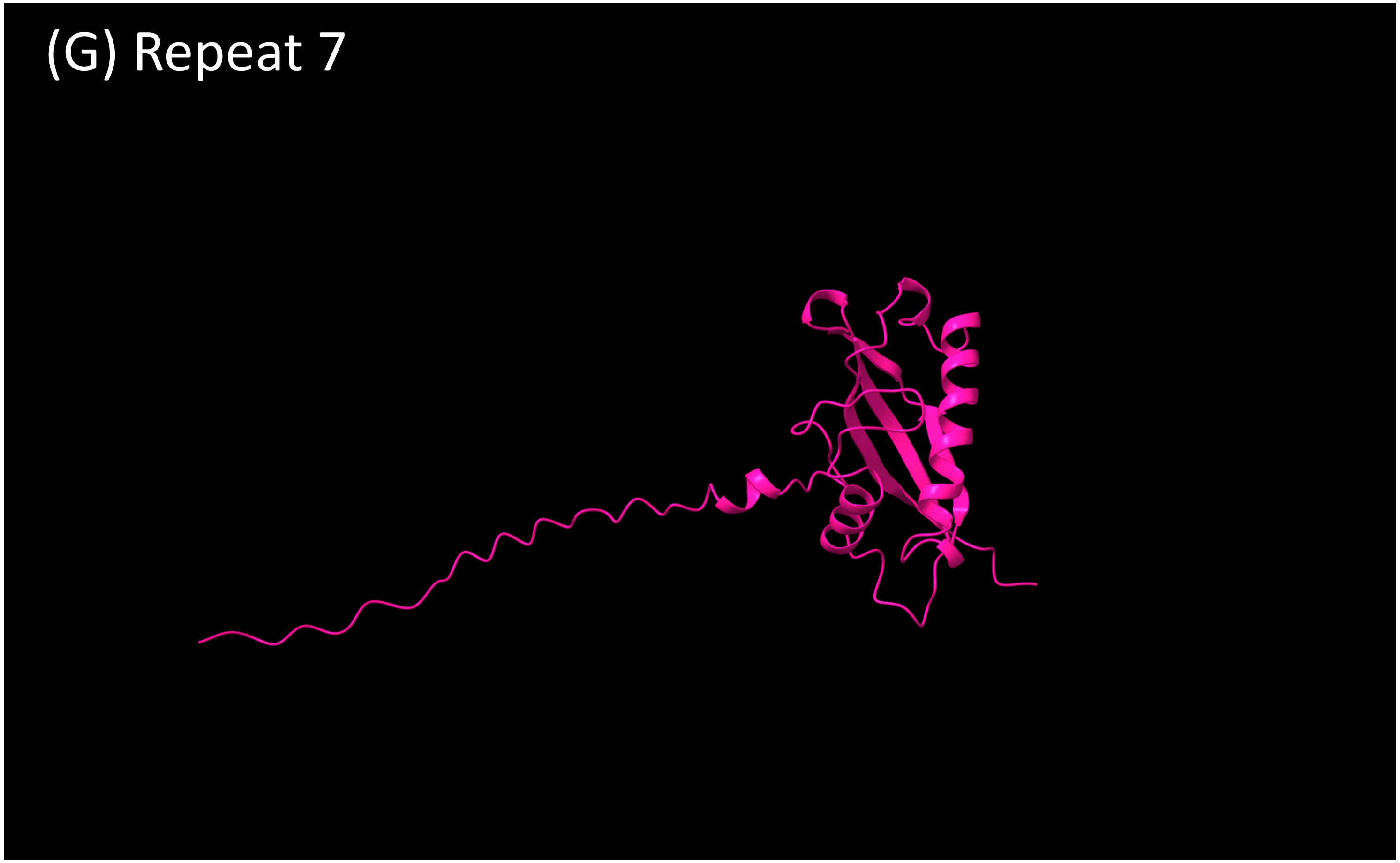
(G) Repeat 7

(H) Repeat 8

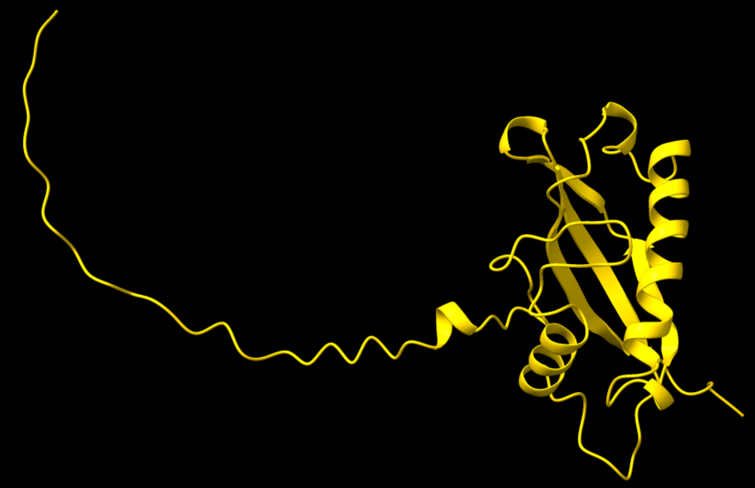

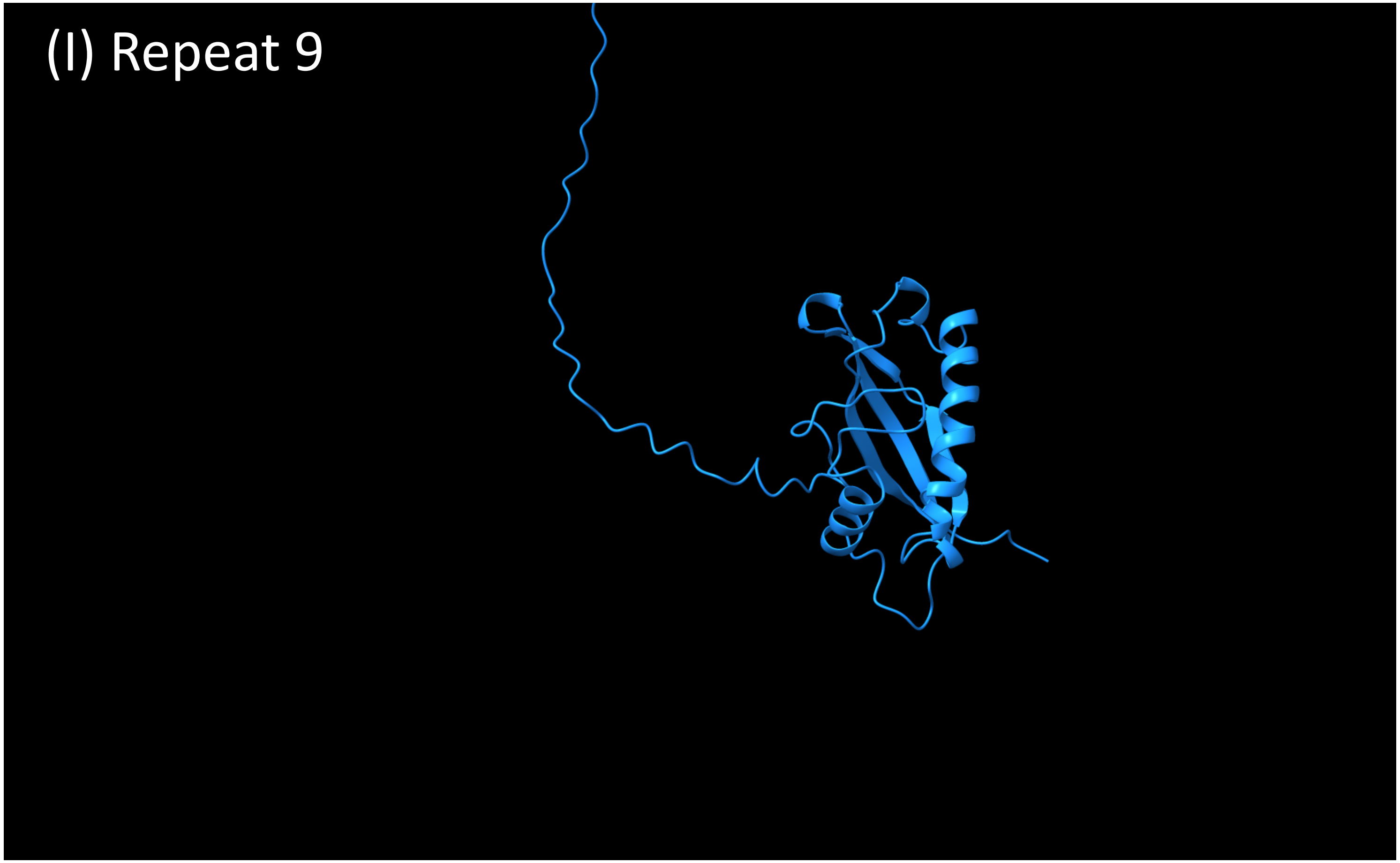
(I) Repeat 9

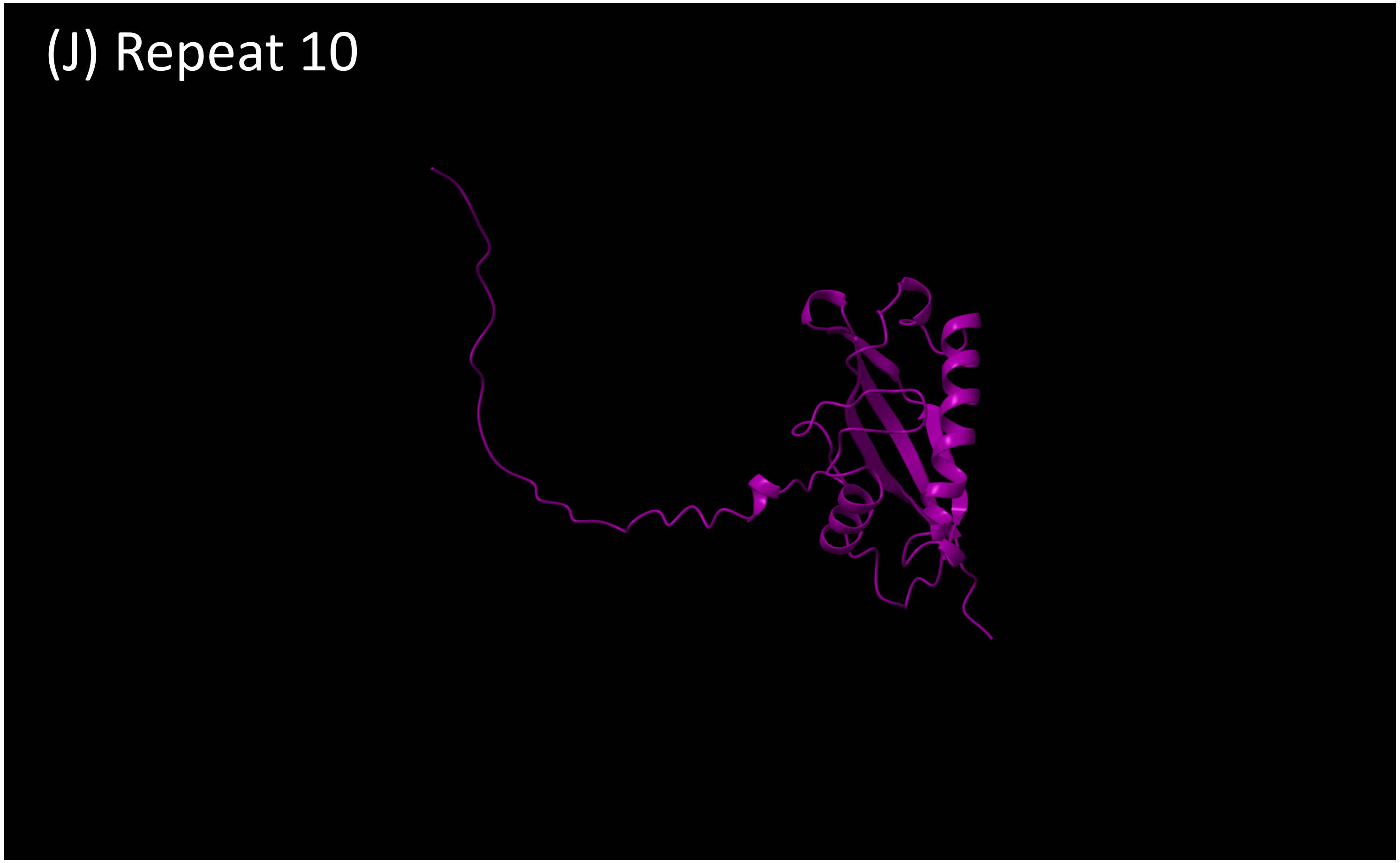

(J) Repeat 10

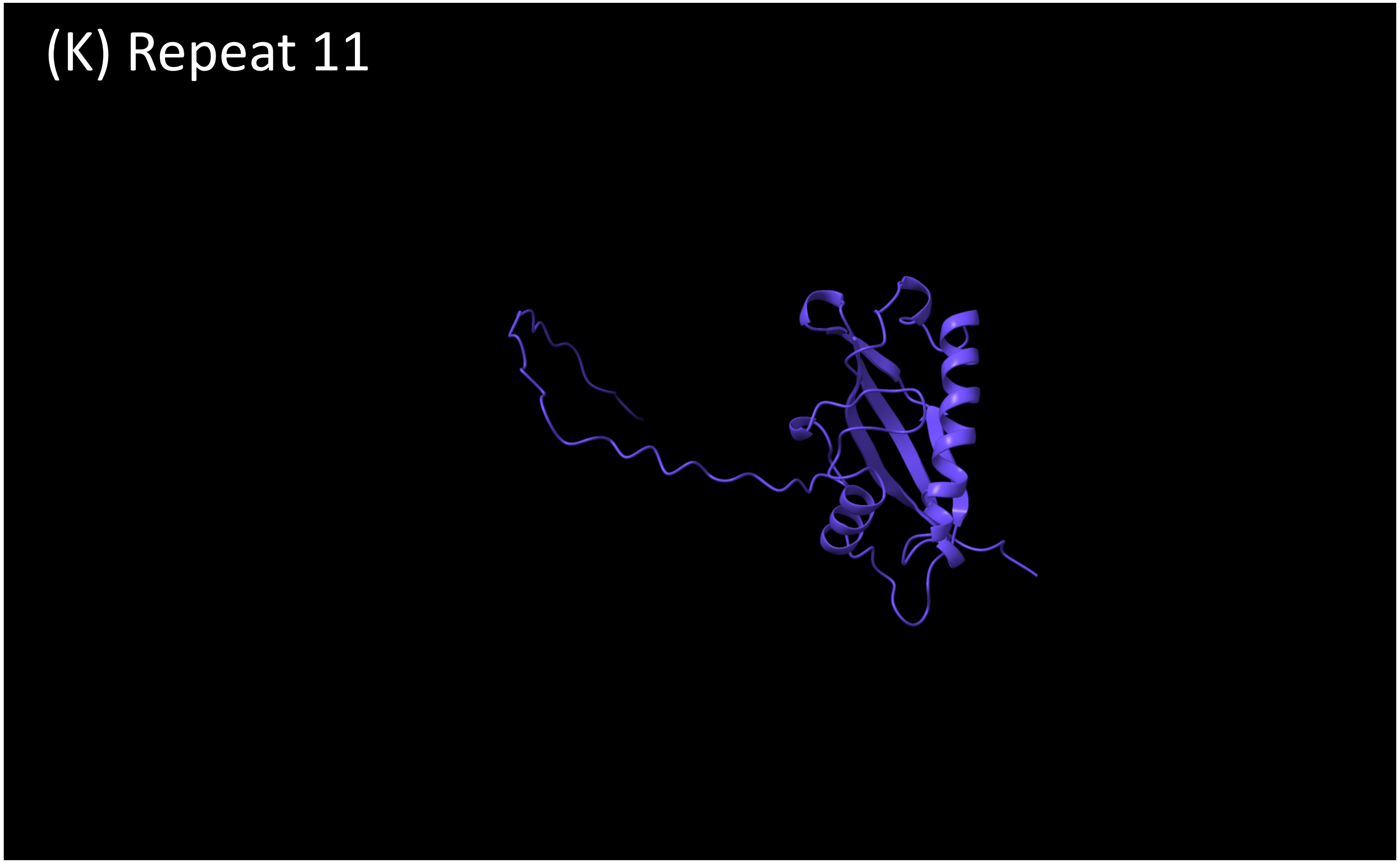
(K) Repeat 11

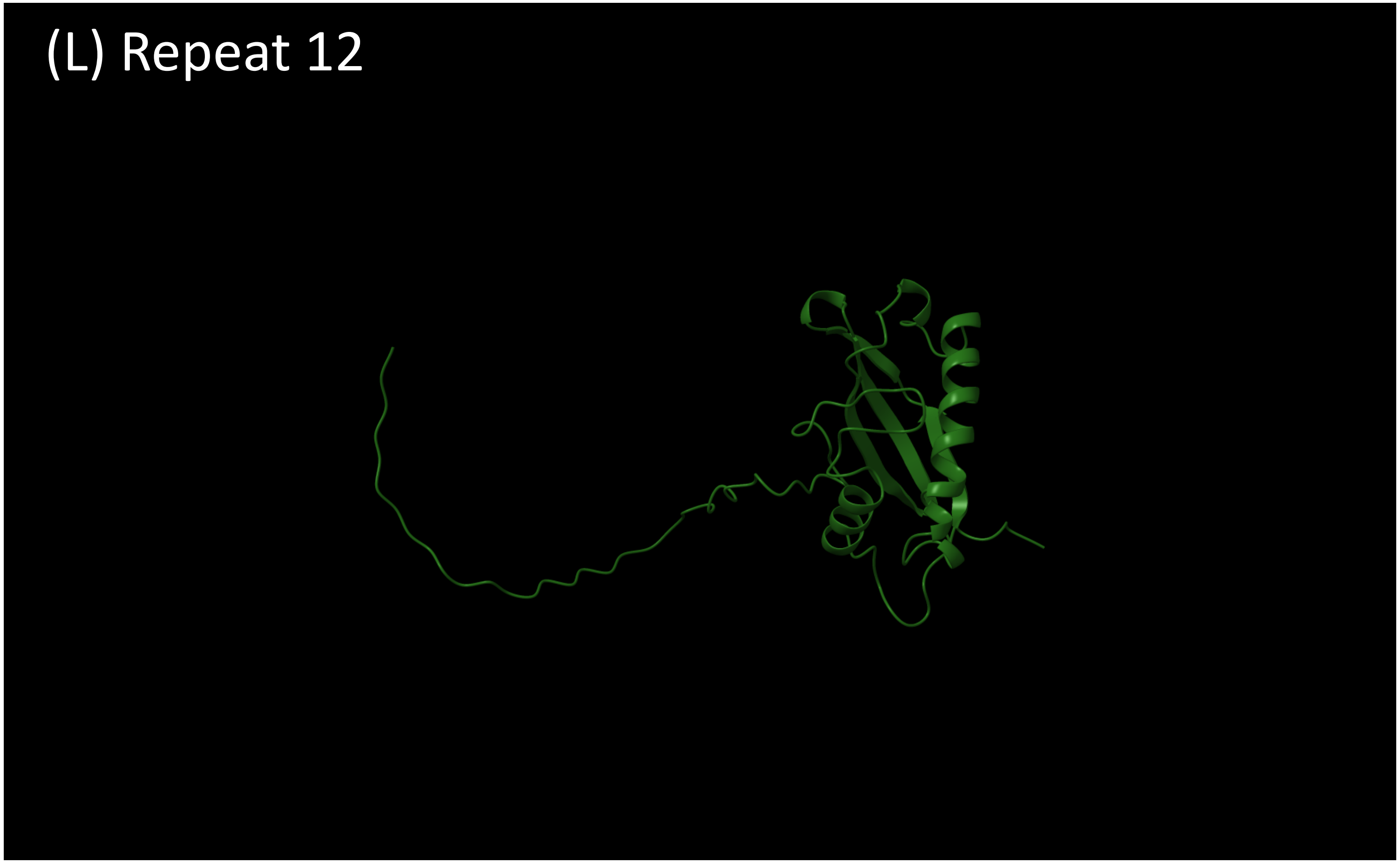
(L) Repeat 12

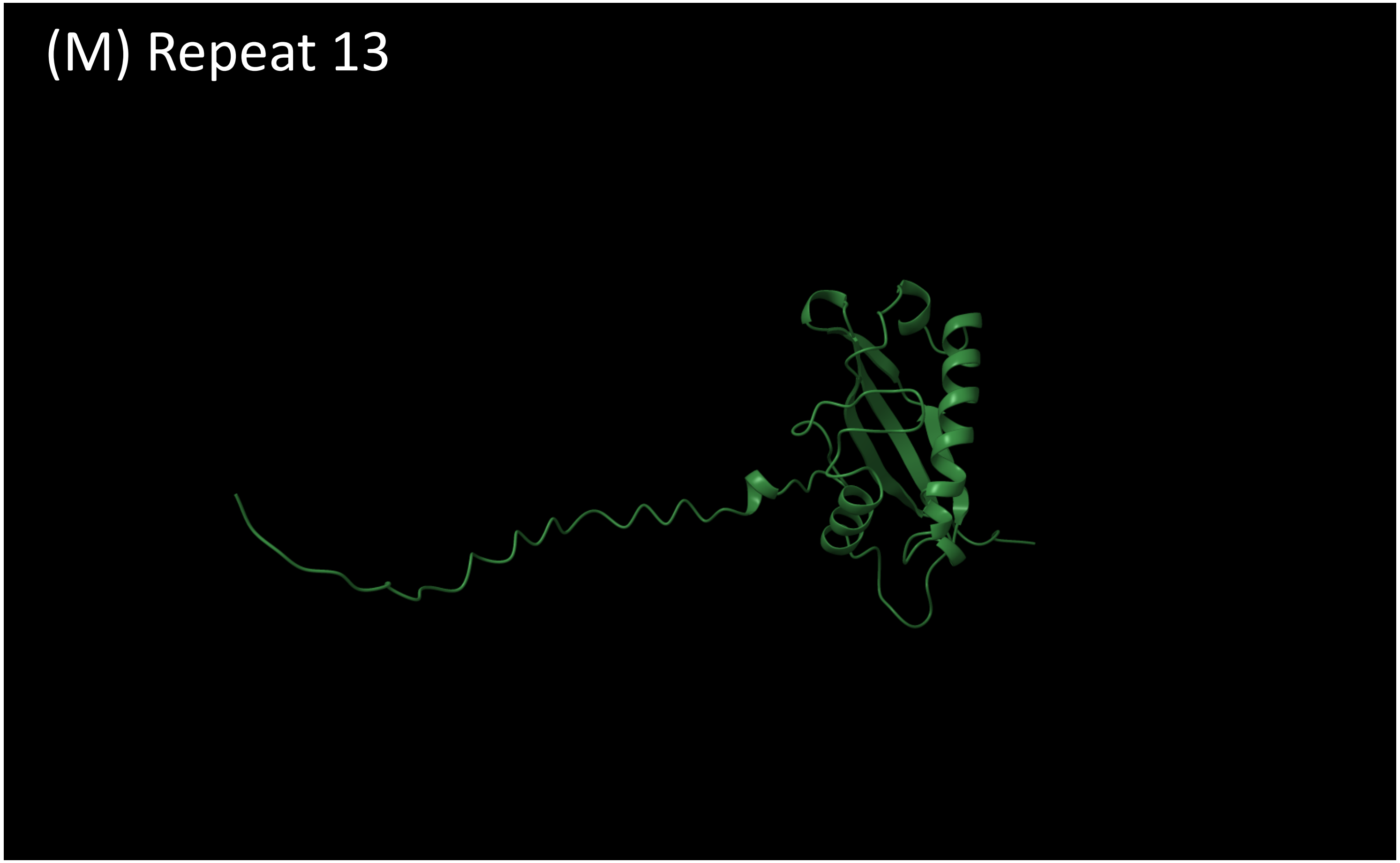
(M) Repeat 13

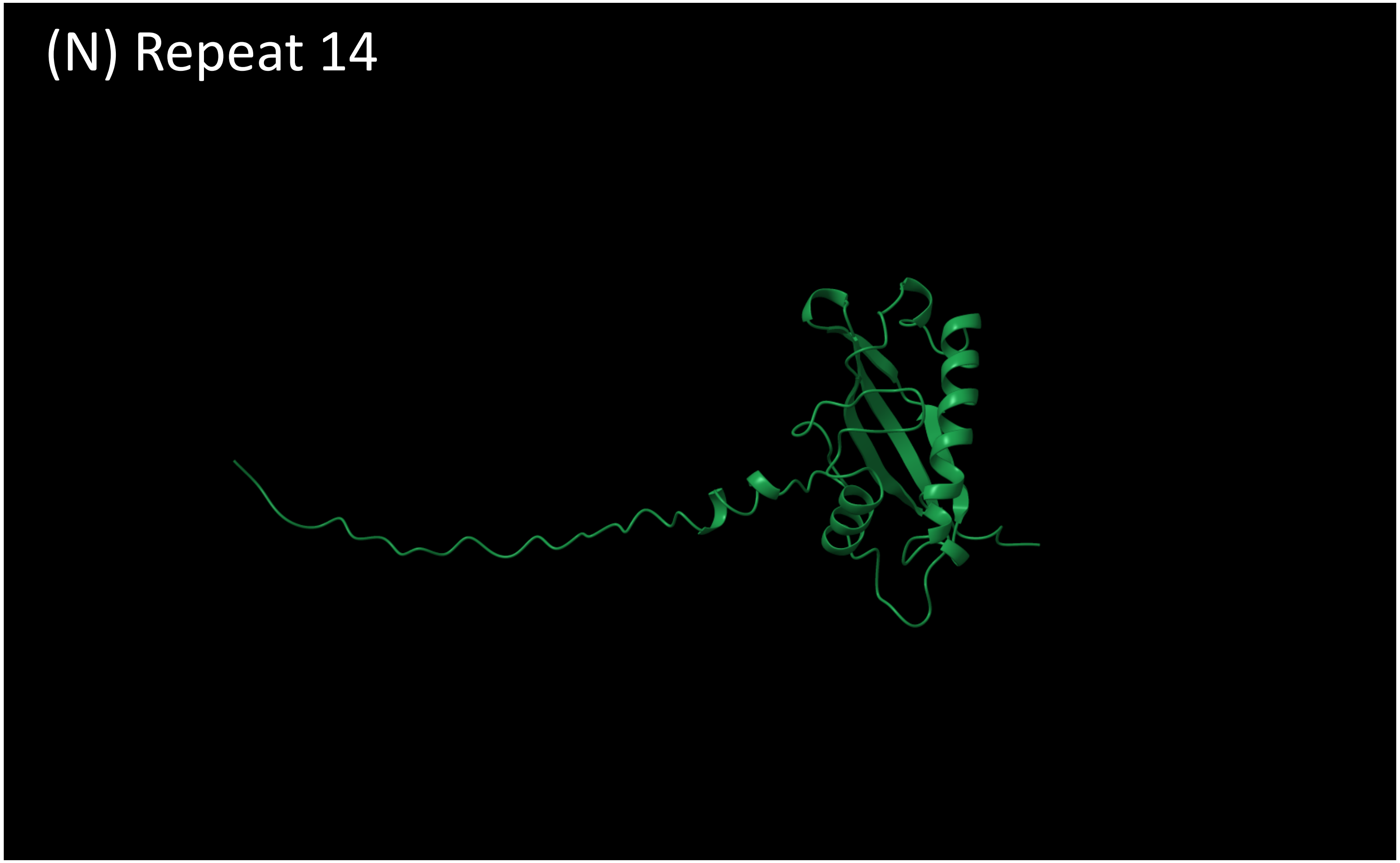
(N) Repeat 14

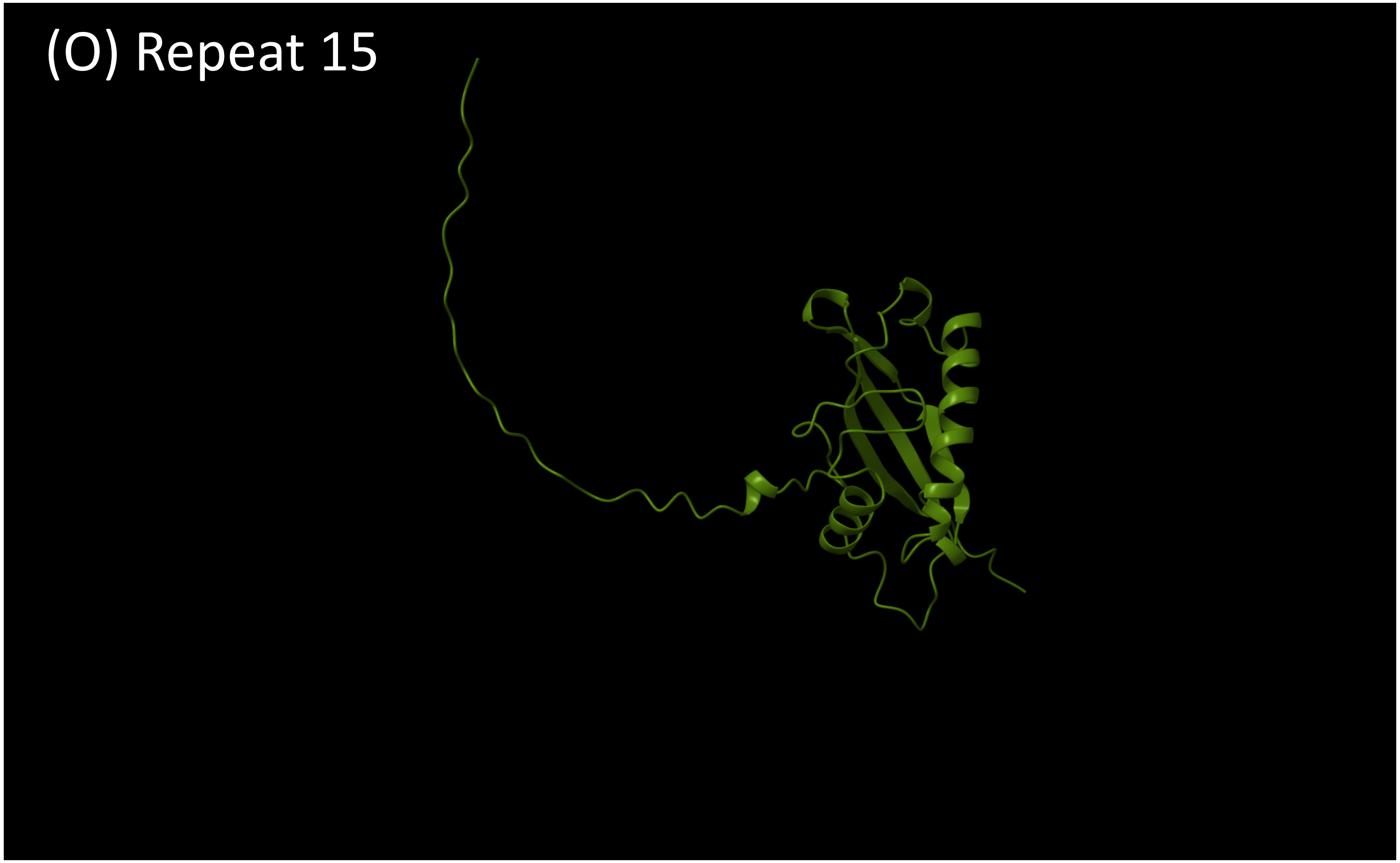
(O) Repeat 15

(P) Repeat 16

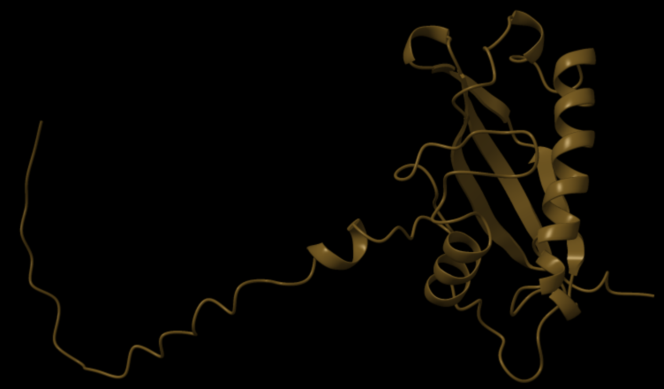

(Q) Repeat 17

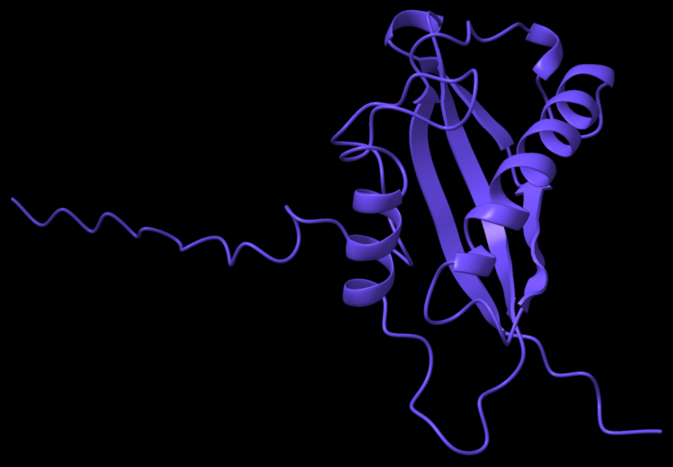

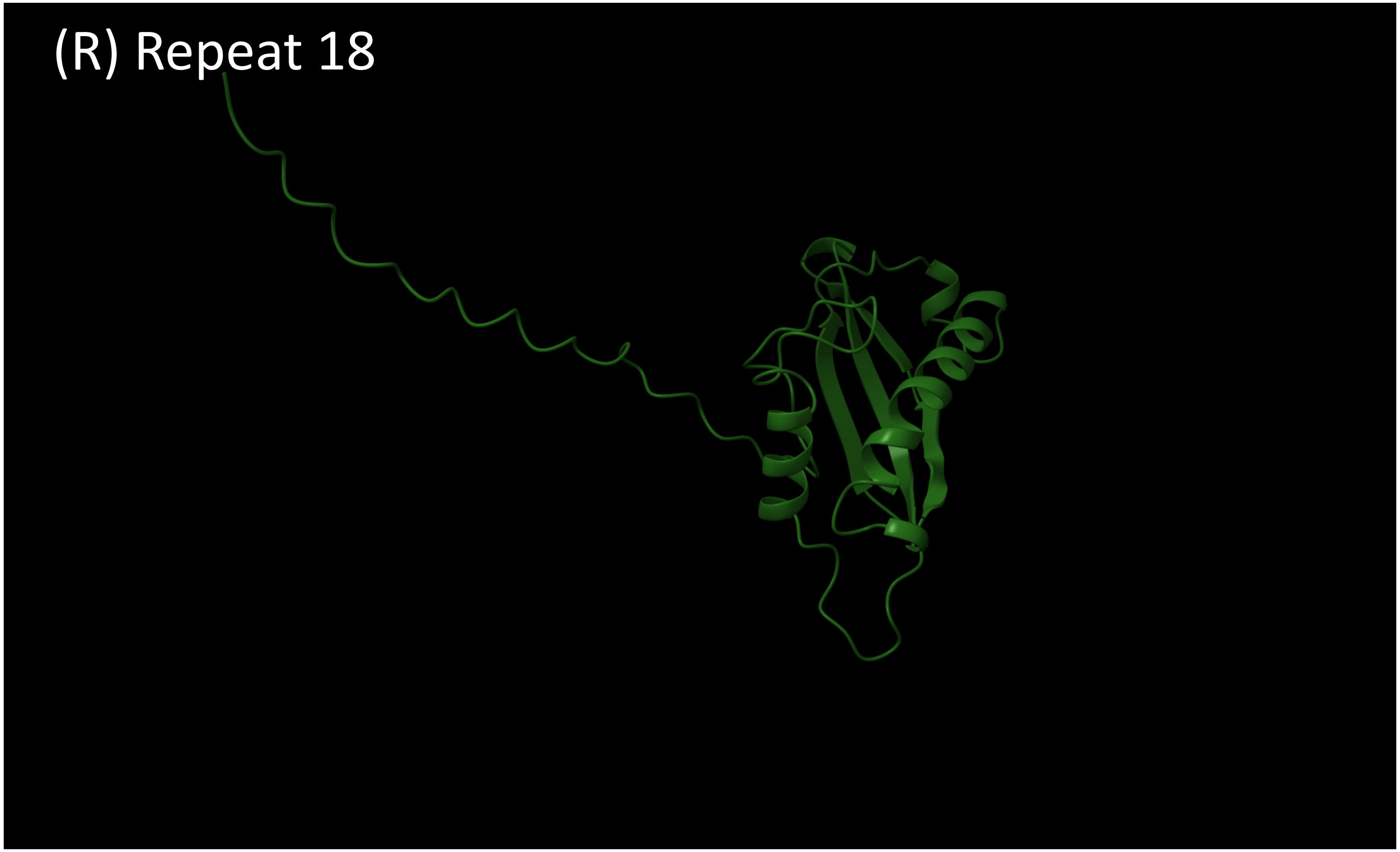
(R) Repeat 18

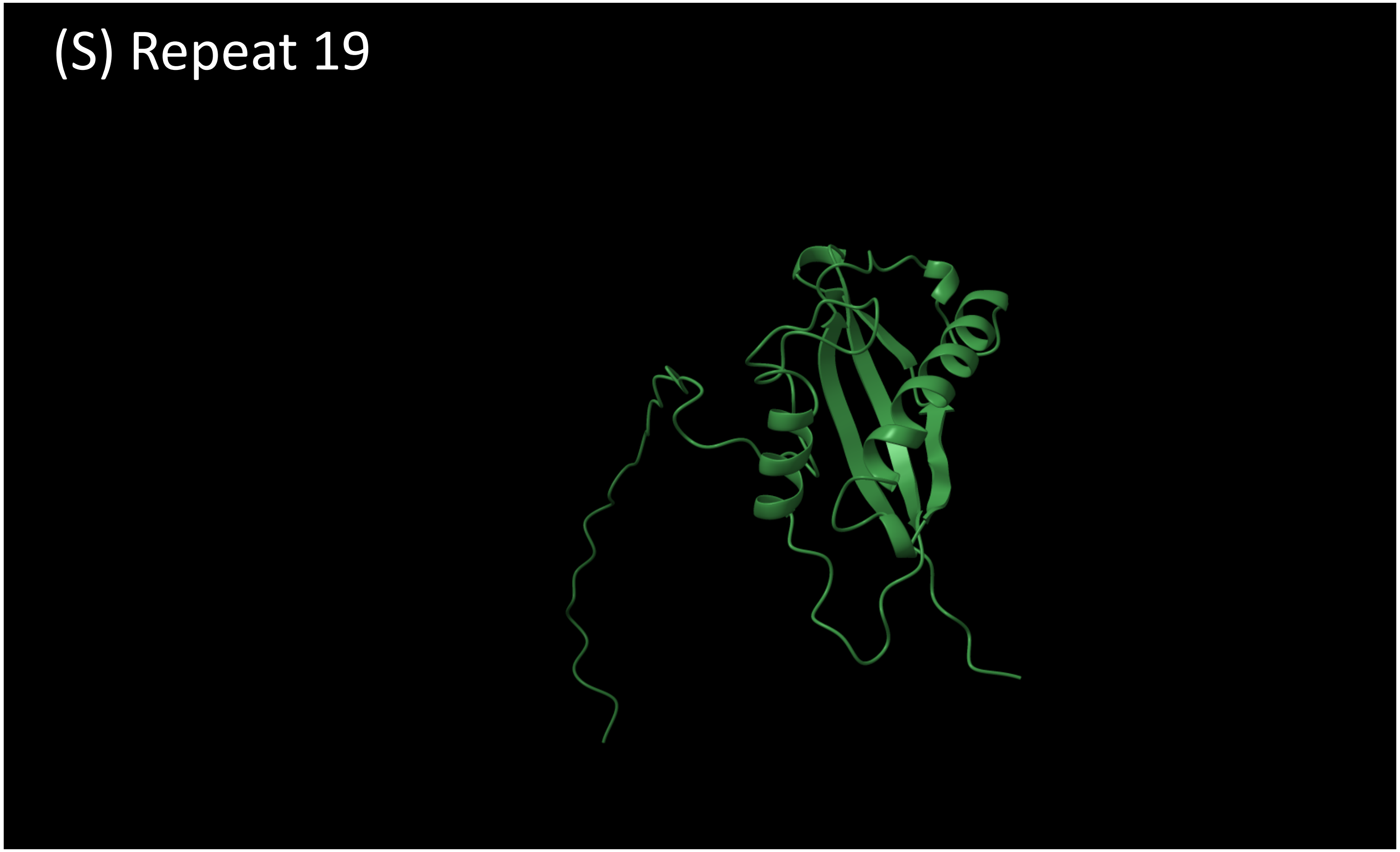

Figure S7. AlphaFold predicted models of individual tandem repeats. (A)-(S) Repeats 1-19.
